# Identification of Chemosensory Genes, Including Candidate Pheromone Receptors, in *Phauda flammans* (Walker) (Lepidoptera: Phaudidae) Through Transcriptomic Analyses

**DOI:** 10.3389/fphys.2022.907694

**Published:** 2022-07-01

**Authors:** Jin Hu, Xiao-Yun Wang, Liu-Su Tan, Wen Lu, Xia-Lin Zheng

**Affiliations:** Guangxi Key Laboratory of Agric-Environment and Agric-Products Safety, College of Agriculture, Guangxi University, Nanning, China

**Keywords:** transcriptome, chemosensory genes, *Phauda flammans*, gene expression, pheromone receptors

## Abstract

Olfactory and gustatory systems play an irreplaceable role in all cycles of growth of insects, such as host location, mating, and oviposition. Many chemosensory genes in many nocturnal moths have been identified *via* omics technology, but knowledge of these genes in diurnal moths is lacking. In our recent studies, we reported two sex pheromone compounds and three host plant volatiles that play a vital role in attracting the diurnal moth, *Phauda flammans*. The antennal full-length transcriptome sequence of *P. flammans* was obtained using the Pacbio sequencing to further explore the process of sex pheromone and host plant volatile recognition in *P. flammans*. Transcriptome analysis identified 166 candidate olfactory and gustatory genes, including 58 odorant-binding proteins (*OBPs*), 19 chemosensory proteins (*CSPs*), 59 olfactory receptors (*ORs*), 16 ionotropic receptors (*IRs*), 14 gustatory receptors (*GRs*), and 2 sensory neuron membrane proteins (*SNMPs*). Subsequently, a phylogenetic tree was established using *P. flammans* and other lepidopteran species to investigate orthologs. Among the 17 candidate pheromone receptor (*PR*) genes, the expression levels of *PflaOR21*, *PflaOR25*, *PflaOR35*, *PflaOR40*, *PflaOR41*, *PflaOR42*, *PflaOR44*, *PflaOR49*, *PflaOR51*, *PflaOR61*, and *PflaOR63* in the antennae were significantly higher than those in other non-antennae tissues. Among these *PR* genes, *PflaOR21*, *PflaOR27*, *PflaOR29*, *PflaOR35*, *PflaOR37*, *PflaOR40*, *PflaOR42*, *PflaOR44*, *PflaOR60*, and *PflaOR62* showed male-biased expression, whereas *PflaOR49*, *PflaOR61*, and *PflaOR63* revealed female-biased expression. The functions of related *OR* genes were also discussed. This research filled the gap of the chemosensory genes of *P. flammans* and provided basic data for future functional molecular mechanisms studies on *P. flammans* olfaction.

## Introduction

In long evolutionary histories, lepidopteran herbivores with abundant species have developed highly sensitive olfactory and gustatory systems, which are essential for survival and reproduction (Song et al., 2020). Host plant volatiles and sex pheromones are dominating odor compounds that usually function together and efficiently guide herbivores to locate mates, hosts, and oviposition sites and avoid natural enemies (Prestwich, 1996; Bruce et al., 2005; Xu and Turlings, 2018). The ability to identify intricate odors in lepidopteran herbivores specifically and extensively depends on their sophisticated sensilla and sensory genes. Among all the main chemosensory organs of adult insects, the antennae typically contain various types of sensilla that play important roles in a number of behaviors (Zacharuk and Shields, 1991; Hildebrand and Shepherd, 1997; Hallem et al., 2006; Gu et al., 2015; Liu et al., 2022). In the sensilla, many sensory genes, including chemosensory proteins, olfactory receptors, ionotropic receptors, gustatory receptors, odorant-degrading enzymes (ODEs), and sensory neuron membrane proteins, are supposed to be involved in host volatile and sex pheromone recognition (Vogt and Riddiford, 1981; Wu et al., 2022). The gene families’ odorant-binding proteins and chemosensory proteins are in charge of binding and transporting externally lipophilic odorant molecules through the sensilla lymph, which is the first step to identifying odorant molecules (Zhou et al., 2021). The first OBP and CSP in lepidopteron are identified from *Antheraea polyphemus* and *Cactoblastis cactorum*, respectively (Vogt and Riddiford, 1981; Maleszka and Stange, 1997). Extensive OBPs and CSPs of lepidopteran herbivores, such as *Chilo suppressalis* (Cao et al., 2014), *Loxostege sticticalis* (Wei et al., 2017), and *Sesamia inferens* (Zhang et al., 2013), have been identified, classified, and analyzed using transcriptomes. Normally, pheromone-binding proteins (PBPs) and general odorant–binding proteins (GOBPs) are the primary subfamilies in OBPs of lepidopteron and can recognize sex pheromone constituents and volatiles from host plants (Zhou et al., 2008; Xing et al., 2021). However, a recent study showed that CpinPBP2 and CpinGOBP1 have similar functions in the identification and transportation of sex pheromones and host plant volatiles in *Conogethes pinicolalis* (Jing et al., 2020).

Receptor protein families, that is, ORs, IRs, and GRs, are used to recognize semiochemicals in the fields (Clyne et al., 1999, 2000; Benton, 2009). The OBP–odorant complex or the OBP-released individual odorant in proximity are recognized by relevant receptor proteins on the dendritic membrane, resulting in chemical signals transforming into electrical signals and then guiding the central nervous system (CNS) to make corresponding commands about the ethological response of lepidopteran insects (Leal, 2013; Wicher and Miazzi, 2021; Wu et al., 2022). During the process of the odorant identification, ORs with odorant receptor co-receptor (Orco) form tetramer as the channels of odorant-gated ions to respond to odors and pheromones (Liu N et al., 2018; Sun et al., 2019). Orco is a unique and highly conserved ortholog considered as the necessary factor for the localization, stability, and correct protein folding of ORs (Larsson et al., 2004; Neuhaus et al., 2005; Martin and Alcorta, 2011). The knockout or mutation of the Orco gene can lead to the loss of odorant function in lepidopterons (Koutroumpa et al., 2016). PRs are highly conserved orthologs, and nearly all PR sequences in moth species are clustered into PR clades including the classical type I, novel, type II, and type 0 PR clades, which are separated from other odorant receptor clades (Zhang and Löfstedt, 2015; Yuvaraj et al., 2022). As a member of the ionotropic glutamate receptor (iGluR) gene family, IRs have a wide range of functions besides odor identification and can take part in the process of sensation of temperature, humidity, and taste (Chen et al., 2015; Zhang et al., 2019; Sun D. et al., 2020). IRs can be divided into antennal IRs (A-IRs), divergent IRs (D-IRs), and Lepidoptera-specific IRs (LS-IRs) (Croset et al., 2010; Zhou et al., 2021). GRs are necessary to sense gustatory substances (Knecht et al., 2016; Xing et al., 2021). GRs can also be classified as CO_2_, sugars, fructose, and bitter receptors, which share relatively high conservation (Ning et al., 2016; Xu, 2020). The ORs, IRs, and GRs of lepidopteran herbivores are first reported on *Heliothis virescens* (Krieger et al., 2002), *Bombyx mori* (Wanner and Robertson, 2008), and *Spodoptera littoralis* (Olivier et al., 2011). To date, a number of receptor protein genes, including *Oraesia emarginata* (Feng et al., 2017), *Semiothisa cinerearia* (Liu et al., 2020), *Achelura yunnanensis* (Li G.-C. et al., 2021), and *Dioryctria abietella* (Wang et al., 2021), have been identified. Among the receptor proteins, PRs are a special type of OR that participate in pheromone reception (Wu et al., 2022). Therefore, extensive studies have been conducted on its identification and function in insect pests for potential control (Tian et al., 2021), including BmOR1 and BmOR2 of *B. mori* (Sakurai et al., 2004; Nakagawa et al., 2005); HR13, HR14, and HR16 of *H. virescens* (Grosse-Wilde et al., 2007); and PxOR1, MsOR1, and DiOR1 of *Plutella xylostella*, *Mythimna separata*, and *Diaphania indica*, respectively (Mitsuno et al., 2008).

SNMP, a member of the CD36 protein family, plays an important role in the process of accepting sex pheromones, degrading the breakdown products of odors, and immunity (Pregitzer et al., 2014; Zhang et al., 2020; Li G.-C. et al., 2021; Cassau and Krieger, 2021). The latest studies indicated that SNMPs can be classified as SNMP1, SNMP2, and SNMP3 in lepidopterons (Liu J et al., 2015; Zhang et al., 2020). The SNMPs of lepidopteron insects are first identified in *A. polyphemus* (Rogers et al., 1997). Most SNMPs of moths, such as *Carposina sasakii* Matsumura (Tian et al., 2018), *Galleria mellonella* (Jiang et al., 2021), *S. litura* (Zhang et al., 2015), and *S. exigua* (Liu N et al., 2015), have been identified until now.

*Phauda flammans* (Walker) (Lepidoptera: Phaudidae) is a diurnal moth and oligophagous pest widely distributed in Southeast Asia and Southern China (Nageshchandra et al., 1972; Verma and Dogra, 1982; FOF, 2015; Liu J et al., 2015; Liu et al., 2016; Robinson et al., 2019). Larvae primarily consume leaves, and additionally, some of the phloem in *Ficus*, including *Ficus microcarpa* (Miq.) and *F. benjamina* L., resulting in leaf necrosis, and only the bare trunk remains (Liu N et al., 2015; Liu et al., 2016). Early studies found two sex pheromone components from *P. flammans* females, including Z-9-hexadecenal and (Z,Z,Z)-9,12,15-octadecatrienal, and their application in fields gain success (Zheng et al., 2019). In addition, β-rolene, β-caryophylene, and D-limonene in host plant volatiles show remarkable attraction to *P. flammans* adults (Guan et al., 2020). Two PflaPBPs are demonstrated to have high affinity with sex pheromones (unpublished data), as identified from the antennal unigene transcriptome. As the crucial next step of sex pheromone recognition, the identification and functional test of PflaPRs become urgent by comprehensive transcriptome sequencing tools.

Knowledge about olfactory- and gustatory-related genes that participate in the identification of sex pheromone and plant volatiles in *P. flammans* remains lacking. Therefore, the first antennal full-length transcriptome of *P. flammans* is generated using single-molecule real-time (SMRT) sequencing technology. Furthermore, 166 candidate olfactory and gustatory genes, including 58 OBPs, 19 CSPs, 59 ORs, 16 IRs, 14GRs, and 2 SNMPs, are identified by analyzing the transcriptome, constructing evolutionary trees of chemosensory genes, and evaluating the expression profiles of 17 candidate PflaPRs. The results on these chemosensory genes may provide fundamental data for understanding their roles in discerning odorants and identifying specific molecular targets in *P. flammans*.

## Materials and Methods

### Insect Rearing and Tissue Collection

Mature *P. flammans* larvae were collected from Lingli town (108° 81′ E, 22° 85′ N), Qingxiu District, Nanning City, Guangxi Zhuang Autonomous Region, P.R. China from June to July in 2020. Every 10 larvae were reared in a barrel-shaped transparent plastic box (2000 ml in volume) with enough pinholes (1 mm in diameter) under constant conditions of 25 ± 1°C, 80% ± 5% relative humidity, and a photoperiod of 14 L:10 D (light:dark). The larvae were fed with fresh leaves of *F. benjamina* until pupation. The pupae were sorted by sex and placed in different barrel-shaped transparent plastic boxes until eclosion. For real-time quantitative PCR (qRT-PCR), 45 male antennae, 45 female antennae; 30 heads (♀:♂ = 1:1), whose antennae were cut off; 30 legs (♀:♂ = 1:1), 30 wings (♀:♂ = 1:1), 30 thoraxes (♀:♂ = 1:1), and 30 abdomens (♀:♂ = 1:1) were collected in three replications from 1-day-old virgin adults. After the collection, samples were preserved in liquid nitrogen and stored at −80°C for later use.

### RNA Extraction, cDNA Library Construction, Sequencing, and Functional Annotation of the Two Transcriptomes

Total RNA was extracted from one mixture sample including 50 male antennae and 50 female antennae of *P. flammans* using the RNAiso Plus (Takara, Japan) and in accordance with specifications. The degradation and integrity of the total RNA were tracked by 1% agarose gels. RNA concentration and quality were assessed using the NanoPhotometer® N60 spectrophotometer (Implen, Germany).

The cDNA library construction and SMRT (Single-Molecular, Real-Time) sequencing were conducted by the Gene Denovo Biotechnology Co., (Guangzhou, China) to acquire the antennal full-length transcriptome. The purification of mRNA from 3 μg total RNA was performed to produce cDNA libraries, mRNA was enriched by Oligo (dT) beads and reverse transcribed into cDNA using the Clontech SMARTer PCR cDNA Synthesis Kit, and the second-strand cDNA was synthesized. Fragments with size >4 kb were selected using the BluePippin™ Size-Selection System. The SMRTbell library was constructed with the large-scale PCR. DNA was damage repaired, end-repaired, and ligated to sequencing adapters. cDNAs were sequenced on the PacBio Sequel platform. High-quality cyclic consensus sequences were acquired from subreads BAM files. Full-length nonchimeric (FLNC) reads, including 5′ and 3′ primer and polyA structures, were obtained via removing primers, barcodes, polyA tail trimming, and concatemer of full passes. Similar FLNC reads were clustered using minimap2, and the consistency sequence was corrected by performing the quiver algorithm. High-quality isoforms (prediction accuracy ≥0.99) were collected. The final transcriptome isoform sequence was constructed after removing redundant sequences using CD-hit software (similarity threshold reached 0.99) (Fu et al., 2012). The quality and integrity of final transcriptome isoforms were evaluated by assembly quality statistics and BUSCO assessment. The BUSCO assessment of isoforms was performed using the BUSCO v5 ortholog search pipeline with “Insecta” as an ortholog set in the gVolante (Nishimura et al., 2017) (https://gvolante.riken.jp/).

The mRNA was enriched by Oligo (dT) beads and interrupted by ultrasound to acquire the antennal unigene transcriptome. The first strand of cDNA was synthesized in the M-MuLV reverse transcriptase system by using mRNAs as a template. Then, the second-strand cDNA was synthesized and purified. After the end repair, add a tail, and ligation of adapters, PCR was performed to amplify the cDNA. The cDNA was sequenced on the Illumina novaseq 6000 (Illumina, California, United States). After filtering the low-quality data and base quality analysis, clean reads were obtained. The final transcriptome unigene sequence was assembled and obtained using Trinity v2.8.4. The quality and integrity of final transcriptome unigenes were evaluated by assembly quality statistics and BUSCO assessment, respectively.

Isoforms and unigenes were annotated into different databases, including EuKaryotic Orthologous Groups/Clusters of Orthologous Groups (KOG/COG), NCBI nonredundant protein (Nr), Kyoto Encyclopedia of Genes and Genomes (KEGG), and Swiss-Prot database, to predict the assumed functional roles of isoforms and unigenes. Gene Ontology (GO) annotation was performed using Blast2GO software with Nr annotation results of isoforms (Conesa et al., 2005). The functional classification of isoforms and unigenes were analyzed using WEGO software (Ye et al., 2006).

### Sequence Analysis and Evolutionary Tree Construction of *P. flammans*

Two antennal transcriptomes, that is, full-length transcriptome and unigene transcriptome, were used to identify candidate olfactory genes in *P. flammans*. “OBP and odorant-binding protein,” “CSP and chemosensory protein,” “OR and odorant receptor,” “IR and ionotropic receptor,” “GR and gustatory receptor,” and “SNMP and sensory neuron membrane protein” were used as keywords to screen the annotated chemosensory genes by the results of Nr annotation from two antennal transcriptome datasets. The similarity in candidate olfactory sequence was analyzed using the Blastx search in the NCBI database (Altschul et al., 1997) (https://blast.ncbi.nlm.nih.gov/Blast.cgi?PROGRAM=blastx&PAGE_TYPE=BlastSearch&LINK_LOC=blasthome). The open reading frames (ORFs) of candidate olfactory genes were estimated using the NCBI ORF finder (https://www.ncbi.nlm.nih.gov/orffinder/). The putative N-terminal signal peptides and transmembrane domains (TMDs) of candidate olfactory genes were predicted by running the SignalP 5.0 Server (Almagro Armenteros et al., 2019) (https://services.healthtech.dtu.dk/service.php?SignalP-5.0) and TOPCONS (Tsirigos et al., 2015) (https://topcons.net/pred/result/rst_651nhxzl/). The amino acid (aa) sequence alignment of candidate olfactory genes in *P. flammans* and the homologous sequence obtained from different lepidopteran families (Supplementary Tables S4, S6, S8, S10, S12, and S14) were analyzed using the Clustal W method (Ye et al., 2006) in the MEGA (version 7.0, Mega Limited, Auckland, New Zealand) software (Kumar et al., 2016). Then, a phylogenetic tree was constructed using the neighbor-joining method in the IQ-TREE web server (Trifinopoulos et al., 2016) (http://iqtree.cibiv.univie.ac.at/). The reliability of the tree structure was evaluated using the 1000-fold bootstrap replication. All phylogenetic trees were established utilizing the FigTree (version 1.4.3, Andrew Rambaut Institute of Evolutionary Biology, England) and Adobe Illustrator (CC 2019; Adobe, America). The olfactory sequence identified from an antennal unigene transcriptome in *P. flammans* (unpublished data) was also included in the phylogenetic analysis to achieve a comprehensive analysis.

### Tissue Expression Profiles of *PflaORs*

The total RNA was extracted from 12 different tissues of *P. flammans* as mentioned earlier. The primers for *PflaORs* were designed using the Primer-BLAST (http://www.ncbi.nlm.nih.gov/tools/primer-blast). *TUB1* (accession number: MN852477) and *GADPH* (accession number: MN852481) were used as reference genes in accordance with a previous study (Chen L et al., 2021). The primers used in this tissue expression are listed in Supplementary Table S1. The amplification efficiencies of primers for target genes were determined using the fivefold dilutions of male antennal cDNA. The specificity of products was evaluated by a melting curve analysis. Melting curves and primer amplification efficiencies are shown in Supplementary Presentation S1 and Supplementary Table S2. On the basis of the phylogeny of PflaORs, 17 candidate PflaPRs in *P. flammans* were selected, and their expression profiles in different tissues were further detected using qRT-PCR with the LightCycler 96 System Real-Time PCR System (Roche, Switzerland). qRT-PCR reactions were executed in 10 µl reaction mixtures containing 5 μl TB Green Premix Ex Taq II (Tli RNaseH Plus, Takara, China), 0.4 µl of each primer (forward and reverse primers, 10 µM), 2 µl sample cDNA, and 2.2 µl sterilized H_2_O. Thermocycling conditions are as follows: predenaturation (95°C for 30 s, one cycle), PCR reaction (95°C for 5 s and 60°C for 30 s, 40 cycles in total), and melt curve (95°C for 15 s, 60°C for 1 min, and 95°C for 15 s). Each qRT-PCR reaction was carried out with 3 technical and 3 biological replicates. The relative expression levels of *PflaORs* were measured using the 2^−∆∆CT^ method, where the heads were used as the internal control (Livak and Schmittgen, 2001). All data were analyzed using SPSS Statistics 26.0 (IBM, Armonk, United States). The expression of *PflaORs* in different tissues was analyzed using a one-way analysis of variance followed by the Tukey’s honest significant difference test. In the same tissue, the expression of *PflaORs* in different sexes was analyzed using the independent sample t-test. Graphs were drawn using GraphPad Prism 5 (GraphPad Software, San Diego, California, United States).

## Results

### Identification and Phylogenetic Analysis of Candidate *OBPs*

The antennal full-length transcriptome of *P. flammans* was created using the PacBio Sequel platform. A total of 20,332 isoforms with a total length of 52,425,403 bp were acquired. The average length of isoforms was 2578.47 bp. The N50 value of isoforms was 2909 bp (Supplementary Figure S1). In the result of BUSCO assessment, the complete, fragmented, and missing BUSCOs accounted for 49.09%, 51.50%, and 48.50%, respectively (Supplementary Figure S2). The unigene transcriptome of *P. flammans* was also created using the Illumina novaseq 6000 platform. A total of 99 386 unigenes with a total length of 90 588 857 bp were acquired. The average length of unigenes was 911 bp. The N50 value of unigenes was 1862 bp (Supplementary Figure S3). The result of BUSCO assessment showed that the complete, fragmented, and missing BUSCOs accounted for 93.30%, 3.67%, and 1.94%, respectively (Supplementary Figures S4, S5).

**FIGURE 1:**
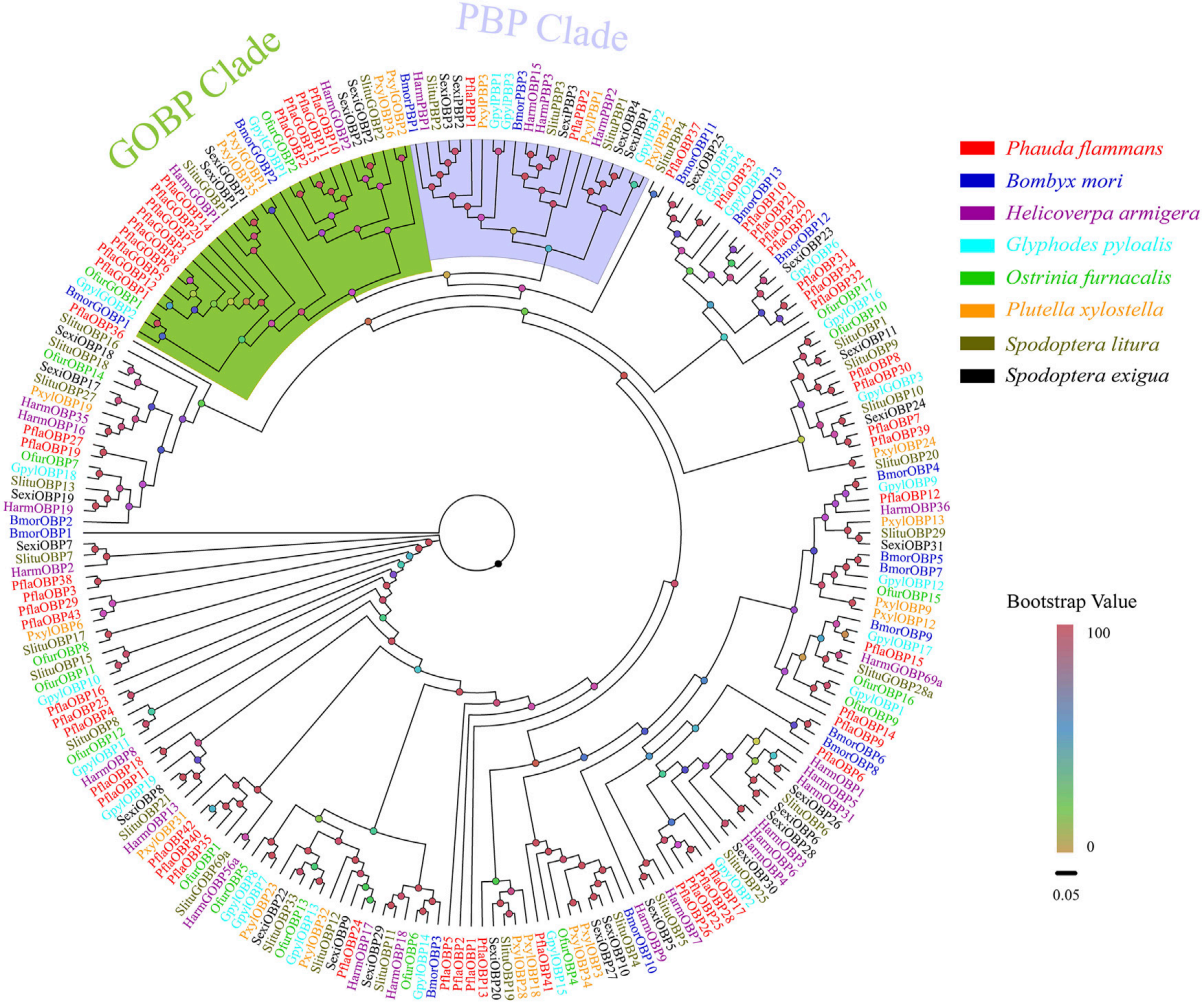
Phylogenetic tree of candidate odorant–binding proteins (OBPs) of *P. flammans* with other lepidopteran insects, including *B. mori*, *H. armigera*, *Glyphodes pyloalis*, *O. furnacalis*, *P. xylostella*, *S. litura*, and *S. exigua*. Bootstrap values are indicated by colors from yellow (0) to red (100). The protein names and gene accession of OBPs used in the phylogenetic tree are recorded in Supplementary Table S4.

A total of 58 candidate *OBPs*, including 43 *PflaOBPs*, 13 *PflaGOBPs*, and 2 *PflaPBPs* from the antennal full-length transcriptome and unigene transcriptome dataset of *P. flammans* were identified*.* This study showed that all candidate *PflaOBPs* had 312–999 bp, which could code 103–332 aa sequences. Among the candidate *OBP* genes, 44 *PflaOBPs* had complete ORFs, and 45 PflaOBPs were predicted that had signal peptides. In total, 16 *PflaOBPs* (i.e., *PflaOBP1*, *PflaOBP3*, *PflaOBP4*, *PflaOBP6*, *PflaOBP12*, *PflaOBP17*, *PflaOBP28*, *PflaOBP35*, *PflaOBP41*, *PflaOBP42*, *PflaGOBP5*, *PflaGOBP6*, *PflaGOBP8*, *PflaGOBP15*, *PflaPflaPBP1*, and *PflaPBP2*) contained six conserved cysteine residues, and this finding was the same as the classic structure of OBPs in other insects (Supplementary Table S3). The GOBP clade was next to the PBP clade, and both clades were phylogenetically far from other OBPs in accordance with phylogenetic analysis. In total, 13 PflaGOBPs could phylogenetically cluster into the GOBP1 and GOBP2 subtribe of other lepidopterans. PflaPBP1 and PflaPBP2 were also closely assembled with other PBPs. Most PflaOBPs were dispersedly distributed in the phylogenetic tree, building orthologous with other lepidopteran OBPs. PflaOBP8 and PflaOBP30 were clustered with SlituOBP10 in the same branch. PflaOBP3 and PflaOBP38 were clustered into an independent branch, as well as PflaOBP4, and PflaOBP23. PflaOBP16 formed separate branches by itself. All of them were considered to have a special function in *P. flammans* (Figure 1).

### Identification and Phylogenetic Analysis of Candidate *CSPs*

**FIGURE 2:**
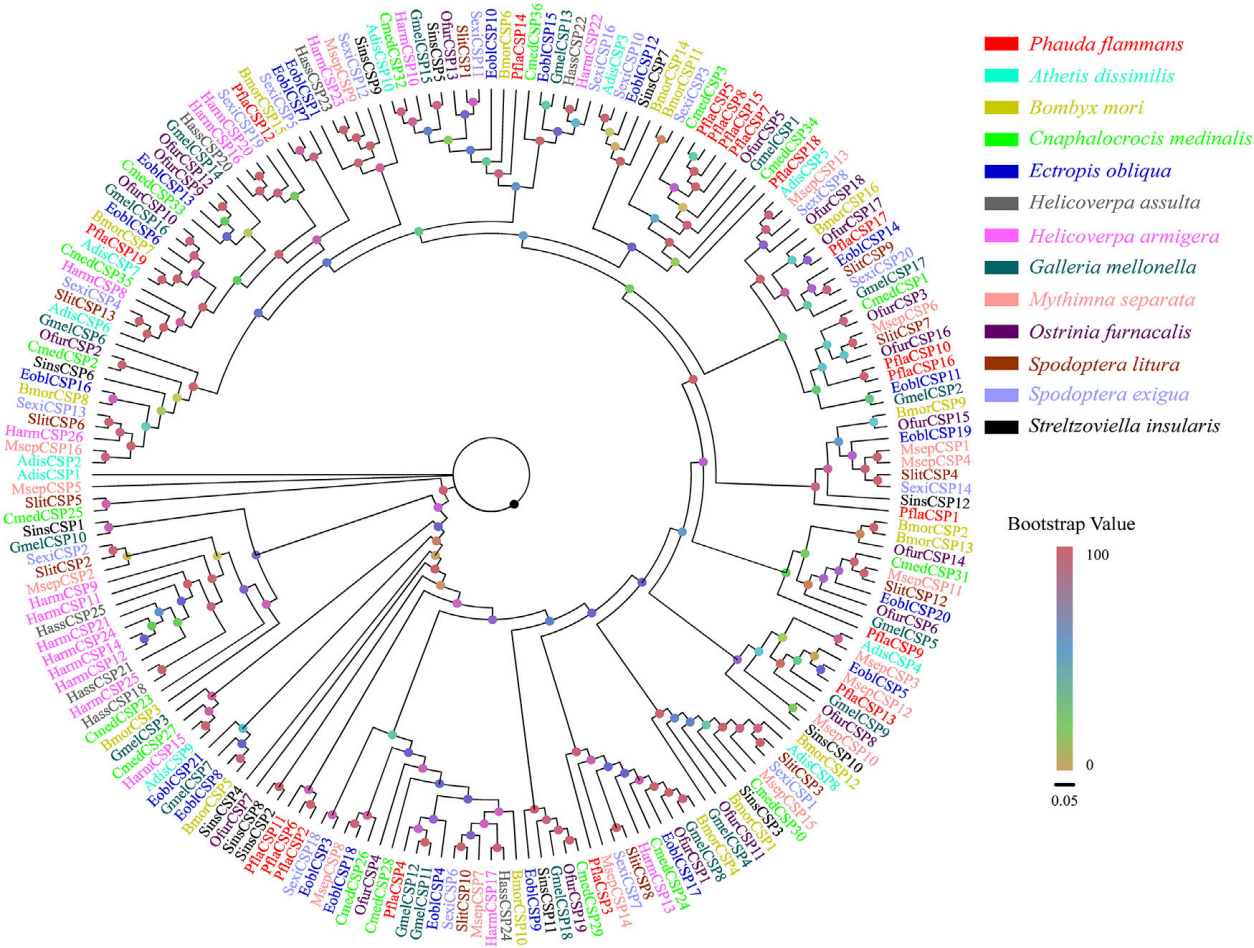
Phylogenetic tree of candidate chemosensory proteins (CSPs) of *P. flammans* with other lepidopteran insects, including *Athetis dissimilis*, *B. mori*, *Cnaphalocrocis medinalis*, *Ectropis obliqua*, *H. assulta*, *H. armigera*, *G. mellonella*, *M. separata*, *O. furnacalis*, *S. litura*, *S. exigua*, and *Streltzoviella insularis*. Bootstrap values are indicated by colors from yellow (0) to red (100). The protein names and gene accession of CSPs used in the phylogenetic tree are recorded in Supplementary Table S6.

A total of 19 candidate *CSPs*, which had 312–546 bp, were also identified. Among these *CSPs*, 18 *PflaCSPs* were predicted to have complete ORF to code 103–181 aa sequences. All PflaCSPs had 15–19 aa signal peptides (Supplementary Table S5). The phylogeny of CSPs indicated that the amount of PflaCSPs except PflaCSP5, PflaCSP8, PflaCSP15, and PflaCSP17 was dispersed widespread in varied branches. In addition, PflaCSP2, PflaCSP6, and PflaCSP11 were clustered together with the homologous clade (Figure 2).

### Identification and Phylogenetic Analysis of Candidate *ORs*

**FIGURE 3:**
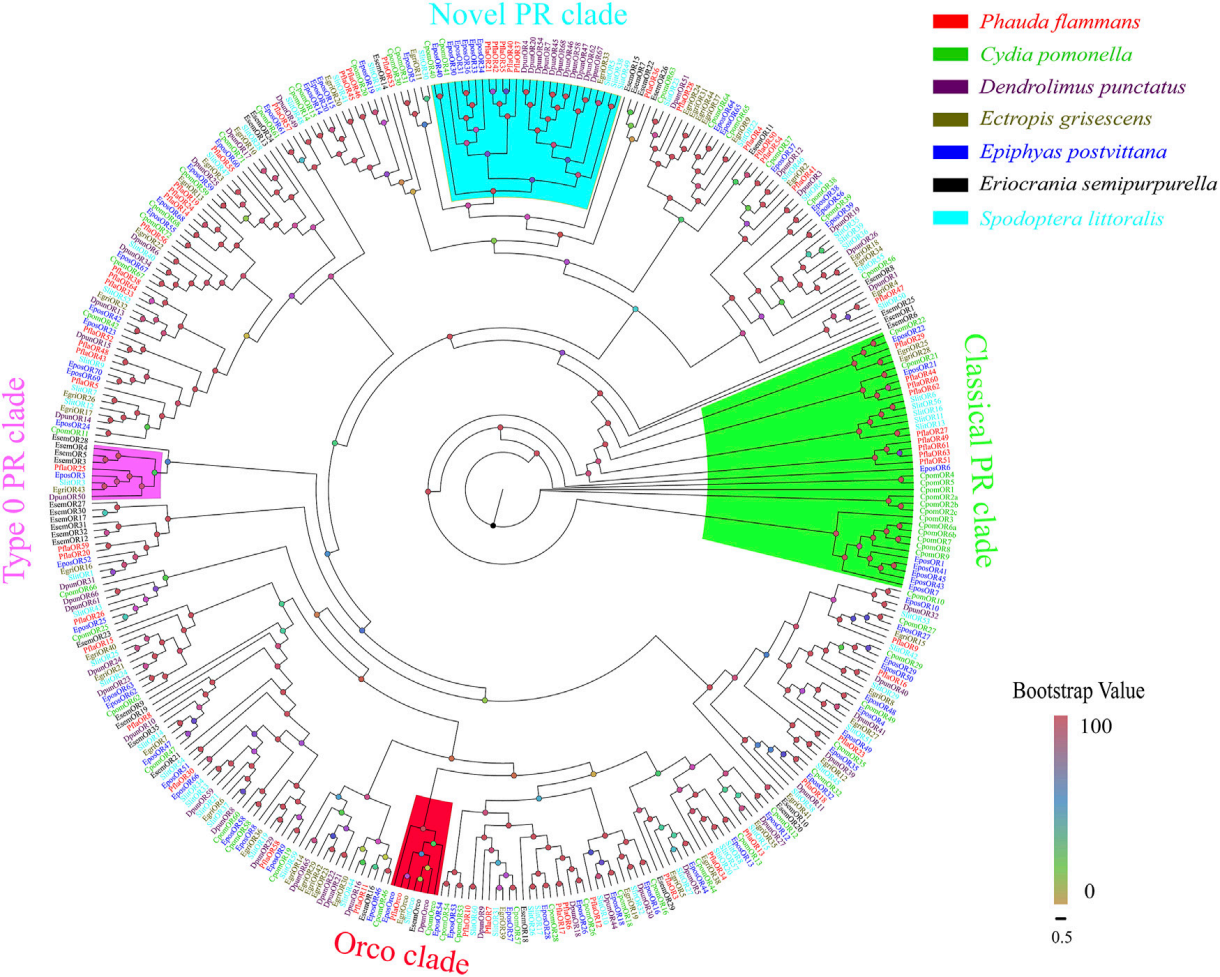
Phylogenetic tree of candidate olfactory receptors (ORs) of *P. flammans* with other lepidopteran insects, including *Cydia pomonella*, *Dendrolimus punctatus*, *E. grisescens*, *E. postvittana*, *Eriocrania semipurpurella*, and *S. littoralis*. Bootstrap values are indicated by colors from yellow (0) to red (100). The protein names and gene accession of ORs used in the phylogenetic tree are recorded in Supplementary Table S8.

A total of 59 candidate *PflaORs*, which were identified by bioinformatics analysis from the two transcriptomes of *P. flammans*, consisted of 687–1440 bp nucleotide sequences and could code 228–479 aa sequences. Among these *PflaORs*, 51 were forecasted to own full-length ORFs containing 3–7 TMDs (Supplementary Table S7). The phylogenetic tree of ORs clearly showed that PflaORs exhibited high differentiation. Most PflaORs split into different clades. By contrast, the Orco clade and three PR clades were comparatively highly conserved. One PflaOR (PflaOrco) was clustered in the Orco clade. A total of 9 PflaORs (i.e., PflaOR27, PflaOR29, PflaOR44, PflaOR49, PflaOR51, and PflaOR60-63), 5 PflaORs (i.e., PflaOR21, PflaOR35, PflaOR37, PflaOR40, and PflaOR42), and 1 PflaOR (i.e., PflaOR25) were clustered in the classical PR clade, novel clade of type I PR clade, and type 0 PR clade, respectively. Moreover, compared with the ORs from the six other lepidopteran insects, two expansions in the number of ORs in *P. flammans* were found. These expansions were branches of PflaOR14, PflaOR19, and PflaOR24 and branches of PflaOR33, PflaOR38, and PflaOR64. A unique gene, namely, PflaOR53, which had no homologous gene in other species, was also found (Figure 3).

### Identification and Phylogenetic Analysis of Candidate *IRs*

**FIGURE 4:**
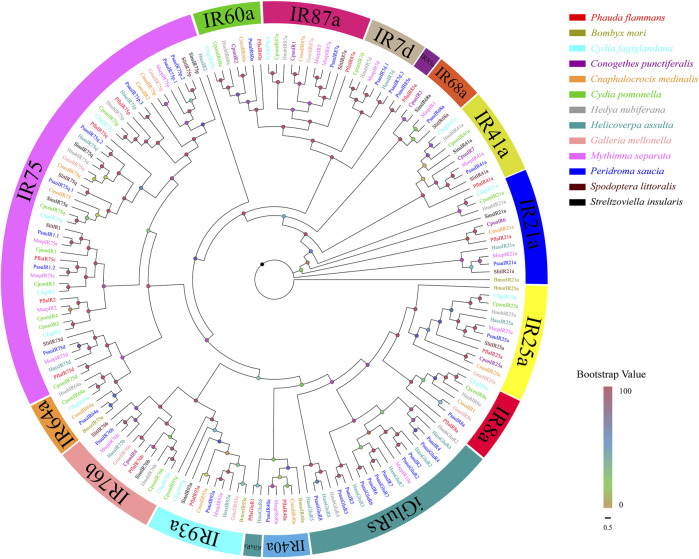
Phylogenetic tree of candidate ionotropic receptors (IRs) of P. flammans with other lepidopteran insects, including *B. mori*, *Cydia fagiglandana*, *Conogethes punctiferalis*, *C. medinalis*, *C. pomonella*, *H. nubiferana*, *H. assulta*, *G. mellonella*, *M. separata*, *Peridroma saucia*, *S. littoralis*, and *S. insularis*. Bootstrap values are indicated by colors from yellow (0) to red (100). The protein names and gene accession of IRs used in the phylogenetic tree are recorded in Supplementary Table S10.

A total of 16 isoforms were recognized as candidate *PflaIRs*. The lengths of isoforms were all 1116–2700 bp, which could code 371–899 aa sequences. Then, 16 *PflaIRs* were predicted to possess complete ORFs containing 1–5 TMDs (Supplementary Table S9). The phylogenetic tree of IRs showed that these PflaIRs were grouped into 15 highly conserved IR subtribes, including IR7d, IR8a, IR21a, IR25a, IR40a, IR41a, IR60a, IR64a, IR68a, IR75, IR76b, IR85a, IR87a, IR93a, and iGluRs (Figure 4). Interestingly, the aa of PflaIR25a was found to share 90.55% identity with HnubIR25a using the best blast-x match. Equally, the aa of PflaIR8a shared 74.6% similarity with EhipIR8a (Supplementary Table S9). In the phylogenetic tree, PflaIR25a and PflaIR8a, which belonged to co-receptor groups IR25a and IR8a, respectively, were clustered with conserved branches. Several PflaIRs (i.e., PflaIR75d, PflaIR75c, PflaIR75p, PflaIR75q, and PflaIR2) were clustered into the IR75 subfamily. In addition, PflaIR76b showed homology with SinsIR76b in the evolutionary tree. PflaIR85a was clustered with PsauIR85a in the same branch (Figure 4).

### Identification and Phylogenetic Analysis of Candidate *GRs*

**FIGURE 5:**
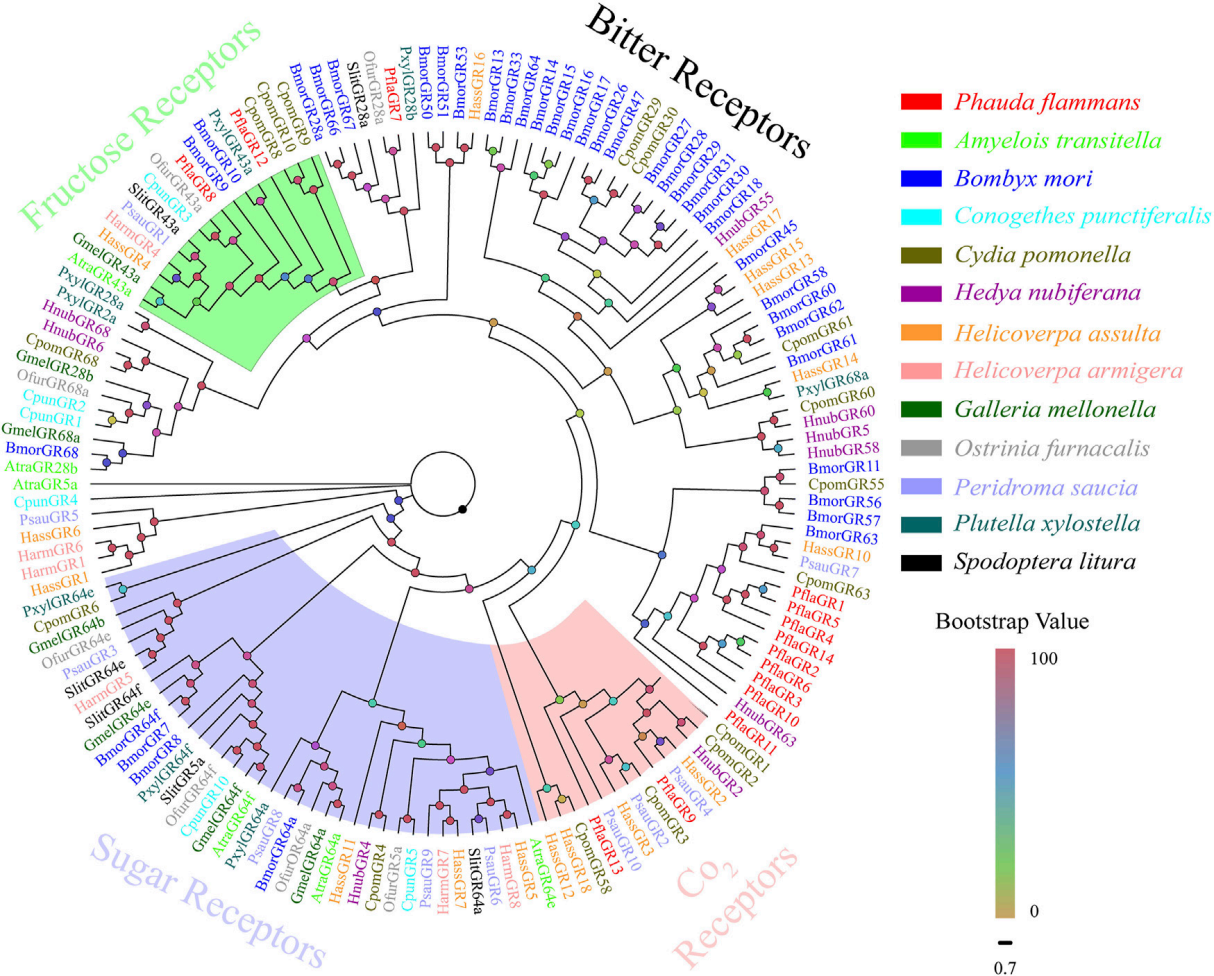
Phylogenetic tree of candidate gustatory receptors (GRs) of *P. flammans* with other lepidopteran insects, including *A. transitella*, *B. mori*, *C. punctiferalis*, *C. pomonella*, *H. nubiferana*, *H. assulta*, *H. armigera*, *G. mellonella*, *O. furnacalis*, *P. saucia*, *P. xylostella*, and *S. litura*. Bootstrap values are indicated by colors from yellow (0) to red (100). The protein names and gene accession of GRs used in the phylogenetic tree are recorded in Supplementary Table S12.

A total of 14 candidate *PflaGRs* were singled out from the antennal transcriptomes of *P. flammans.* They had 621–1275 bp and could code 206–424 aa sequences. Subsequently, five *PflaGRs* were predicted to retain complete ORFs containing 6–7 TMDs (Supplementary Table S11). The phylogenetic tree of GRs manifested that PflaGRs were classified as four GR subfamilies of lepidopteran, including CO_2_, sugar, fructose, and bitter receptors. Among these PflaGRs, 2 (PflaGR8 and PflaGR12) showed homology and were clustered in the fructose receptor branch, and 2 (i.e., PflaGR9 and PflaGR13) showed homology and were clustered in the CO_2_ receptor branch (Figure 5).

### 3.6 Identification and Phylogenetic Analysis of Candidate *SNMPs*

**FIGURE 6:**
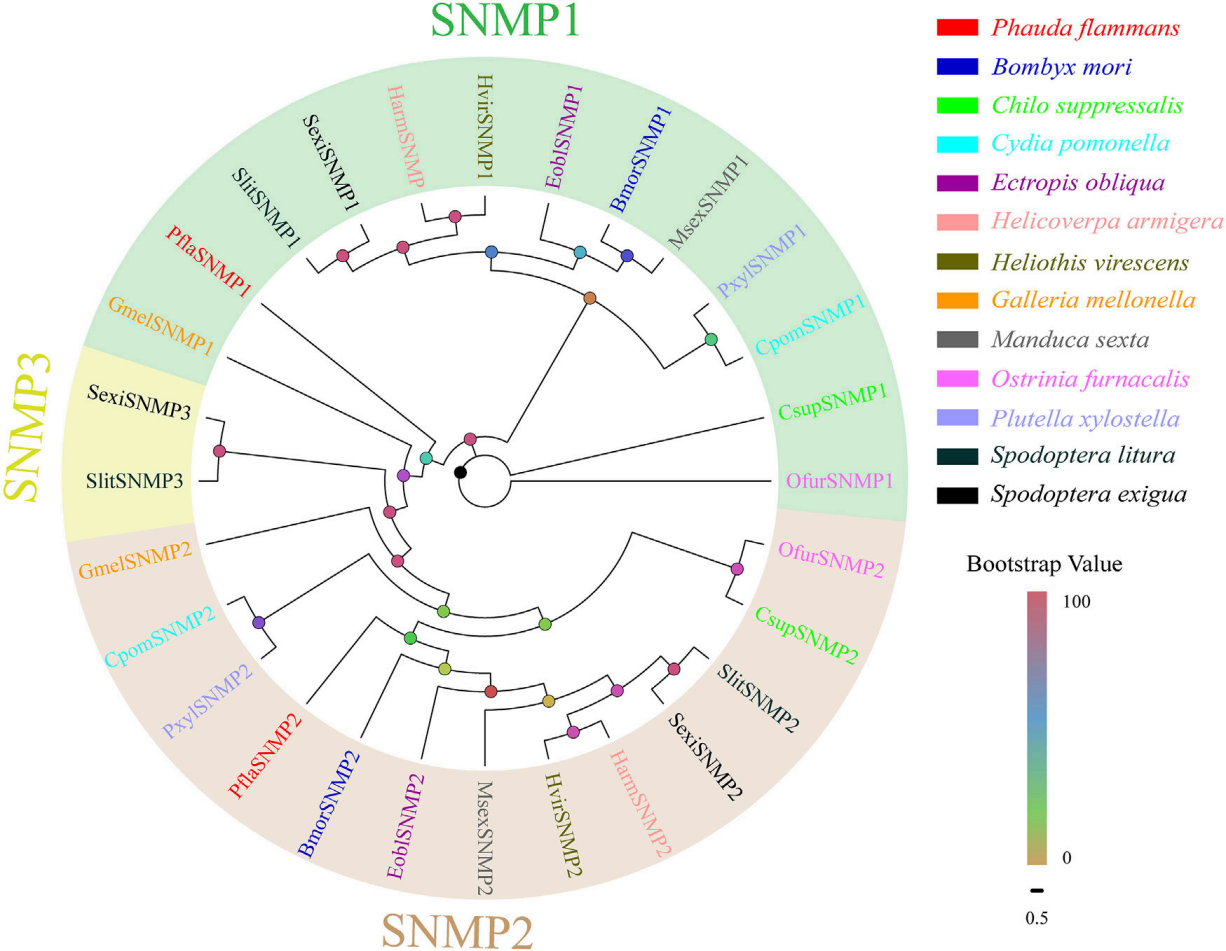
Phylogenetic tree of candidate sensory neuron membrane proteins (SNMPs) of *P. flammans* with other lepidopteran insects, including *B. mori*, *C. suppressalis*, *C. pomonella*, *E. obliqua*, *H. armigera*, *H. virescens*, *G. mellonella*, *M. sexta*, *O. furnacalis*, *P. xylostella*, *S. litura*, and *S. exigua*. Bootstrap values are indicated by colors from yellow (0) to red (100). The protein names and gene accession of SNMPs used in the phylogenetic tree are recorded in Supplementary Table S14.

Two *PflaSNMPs* containing complete ORFs were recognized in the two transcriptomes of *P. flammans.* Both of their nucleotide sequences were 1563 bp and could code 520 aa sequences. PflaSNMPs (i.e., PflaSNMP1 and PflaSNMP2) were predicted to have two conserved TMDs, belonging to the CD36 protein family. PflaSNMP1 shared 77.78% aa identity with SNMP1 in *H. kahamanoa*, whereas PflaSNMP2 was more analogous with OnubSNMP2 (identity: 66.48%, Supplementary Table S13). Phylogenetic analysis showed that PflaSNMP1 and PflaSNMP2 were closely clustered into lepidopteran SNMP1 and SNMP2 branches, respectively (Figure 6).

### Expression Profile Analysis of Candidate *PRs* of *P. flammans*

**FIGURE 7:**
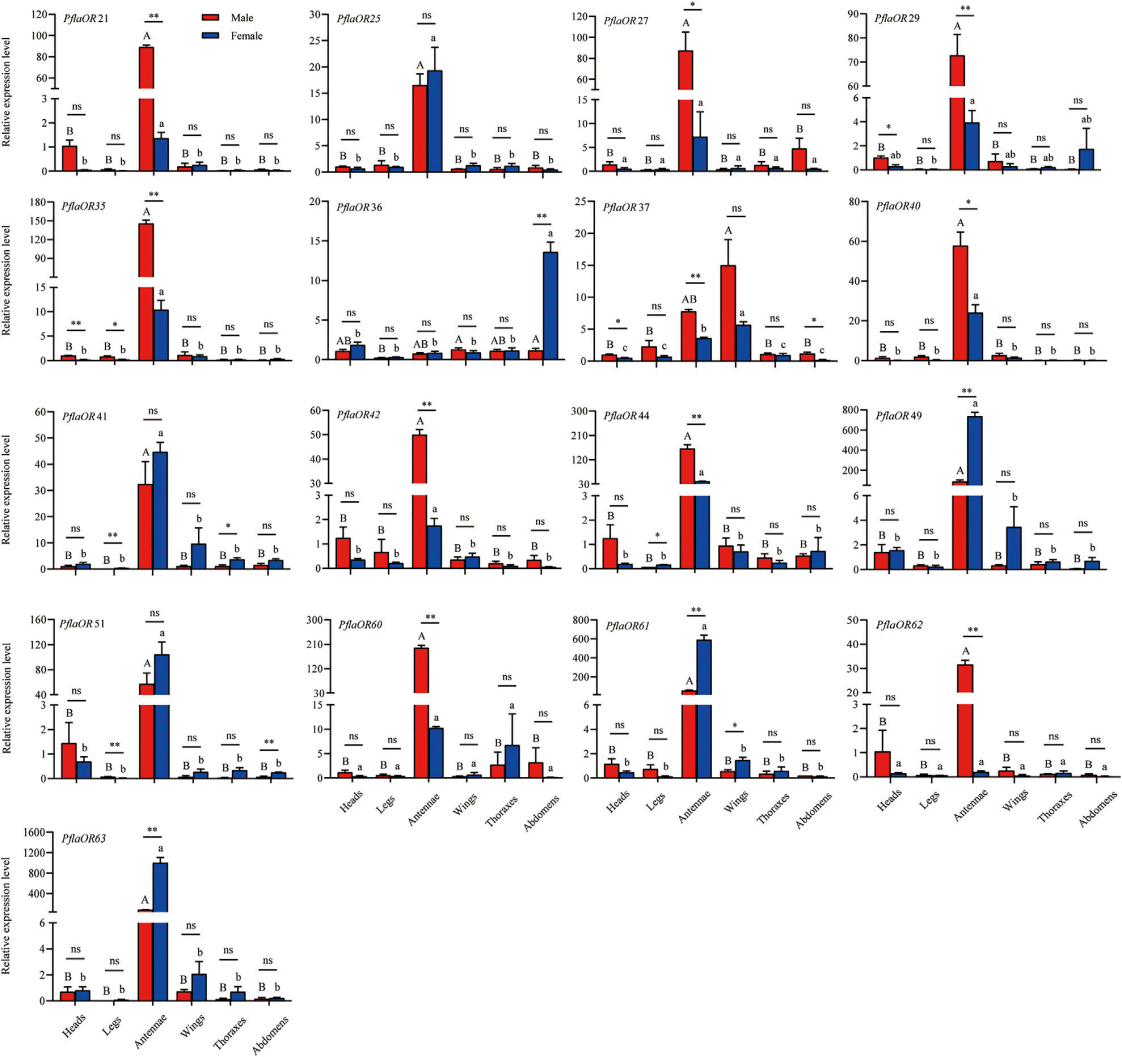
Expression profile of candidate pheromone receptors (*PRs*) in different tissues of male and female adults of *P. flammans*. Note: Data are presented as mean ± SE. Different uppercase and lowercase letters on the column indicated significant differences in the OR expression levels among different tissues of male and female, respectively (ANOVA and Tukey tests*, p < 0.05*). ns, *, and ** on the column indicated no significant difference (*p > 0.05*), significant difference (*p < 0.05*), and highly significant difference (*p < 0.01*), respectively, in the gene expression between different sexes in the same tissue (independent sample *t*-test).

A total of 17 candidate *PR* genes (i.e., *PflaOR21*, *PflaOR25*, *PflaOR27*, *PflaOR29*, *PflaOR35*, *PflaOR36*, *PflaOR37*, *PflaOR40*, *PflaOR41*, *PflaOR42*, *PflaOR44*, *PflaOR49*, *PflaOR51*, *PflaOR60*, *PflaOR61*, *PflaOR62*, and *PflaOR63*) were used to compare tissue- and sex-specific expression in *P. flammans*. RT-qPCR results discovered that the expression levels of 11 candidate *PRs* (i.e., *PflaOR21*, *PflaOR25*, *PflaOR35*, *PflaOR40*, *PflaOR41*, *PflaOR42*, *PflaOR44*, *PflaOR49*, *PflaOR51*, *PflaOR61*, and *PflaOR63*) in the antennae were significantly higher than those in five other tissues of males and females. However, *PflaOR27*, *PflaOR29*, *PflaOR60*, and *PflaOR62* were only significantly expressed in the antennae of males. *PflaOR36* was significantly expressed in the abdomens of females. *PflaOR37* was significantly expressed in the wings of females. Clearly, 13 candidate *PRs* (i.e., *PflaOR21*, *PflaOR27*, *PflaOR29*, *PflaOR35*, *PflaOR37*, *PflaOR40*, *PflaOR42*, *PflaOR44*, *PflaOR49*, *PflaOR60*, *PflaOR61*, *PflaOR62*, and *PflaOR63*) exhibited significantly sex-biased expression in the antennae. Among them, *PflaOR27* and *PflaOR40* showed sex-biased expression in the male antennae, whereas *PflaOR21*, *PflaOR29*, *PflaOR35*, *PflaOR37*, *PflaOR42*, *PflaOR44*, *PflaOR60*, and *PflaOR62* showed highly expressed significant differences in the male antennae compared with those in their female counterpart. By contrast, *PflaOR49*, *PflaOR61*, and *PflaOR63* revealed the female-biased expression in the antennae. *PflaOR36* also displayed the female-biased expression in the abdomens. Other candidate *PRs* (i.e., *PflaOR25*, *PflaOR41*, and *PflaOR51*) had almost similar expression between female and male antennae (Figure 7).

## Discussion

*Phauda flammans* is a serious pest of *Ficus* trees (Liu J et al., 2015; Liu et al., 2016). Although the sex pheromone components of *P. flammans* females and the main components of host plant volatiles have been identified (Zheng et al., 2019; Guan et al., 2020), the mechanisms of how *P. flammans* recognizes these odors through their olfactory and gustatory sensory systems remain unclear. To date, many chemosensory receptor genes of lepidopteran herbivores, e.g., at least 11 families of nocturnal moths (such as Noctuidae, Crambidae, and Cossidae) and some diurnal moths (such as Zygaenidae) (Hu et al., 2016; Jia et al., 2016; Sun et al., 2016; Yang et al., 2020), have been identified by increasingly mature transcriptome sequencing technologies. Most of these studies are focused on nocturnal moths that larvae can cause loss to the agricultural economy, and only a few diurnal moths are paid attention. Therefore, the identification and analysis of candidate olfactory and gustatory genes of *P. flammans* can provide essential information to further understand the chemosensory mechanism of mate and host location in diurnal moths.

In this research, 166 candidate olfactory and gustatory genes, including 58 *OBPs*, 19 *CSPs*, 59 *ORs*, 16 *IRs*, 14 *GRs*, and 2 *SNMPs*, are identified by analyzing the antennal full-length transcriptome and RNA-seq transcriptome of *P. flammans* (the sequences of the 166 genes are provided in Supplementary Material S1)*.* This number is less than that in *D. abietella* (42 *OBPs*, 23 *CSPs*, 75 *ORs*, 30 *IRs*, 62 *GRs*, and 3 *SNMPs*), an oligophagous pest (Wang et al., 2021), and more than that in *O. emarginata* (41 *OBPs*, 20 *CSPs*, 35 *ORs*, six *IRs*, and 2 *SNMPs*); *M. separata* (50 *OBPs*, 20 *CSPs*, 35 *ORs*, six *IRs*, and 2 *SNMPs*); and *C. sasakii* (29 *OBPs*, 13 *CSPs*, 52 *ORs*, eight *IRs*, 11 *GRs*, and 1 *SNMP*) (Feng et al., 2017; Liu et al., 2017; Tian et al., 2018), an omnivorous pest; and *P. xyllostella* (24 *OBPs*, 15 *CSPs*, 54 *ORs*, 16 *IRs*, 7 *GRs*, and 2 *SNMPs*), an oligophagous pest (Yang, et al., 2017). These findings show that the transcriptome database owns high-quality gene numbers belonging to a normal range of moth species.

Oligophagous pests generally require relatively few binding proteins to sense external compounds due to their specificity to host plants (Gouin et al., 2017). In the current study, 58 candidates *OBPs* (43 *PflaOBPs*, 13 *PflaGOBPs*, and 2 *PflaPBPs*) are found from the transcriptome of *P. flammans*. This number is more than most of the identified *OBPs* from transcriptomes in lepidopteran herbivores, such as *C. punctiferalis* (29 *OBPs*), *M. separata* (50 *OBPs*), *P. xyllostella* (24 *OBPs*), and *Athetis lepigone* (28 *OBPs*) (Xiao et al., 2016; Liu et al., 2017; Yang et al., 2017; Zhang et al., 2017). This finding may be because two transcriptome databases are integrated, and a mixture of male and female antennae is used as samples. The phylogenetic tree indicates that PflaPBP1 and PflaPBP2 can cluster in the PBP branch of lepidopterons. However, a third PflaPBP is not found. Three or more PBPs are commonly found in moth, such as *C. punctiferalis* (5 PBPs), *O. emarginata,* and *Clostera restitura* (3 PBPs) (Xiao et al., 2016; Feng et al., 2017; Gu et al., 2019). However, the result of the present study is similar to that of another study, which reports that only two PBPs are found in the diurnal moth *Histia rhodope*. A total of 13 PflaGOBPs are identified in the transcriptome of *P. flammans*. This number is substantially more than those of other moths, such as *S. littoralis* (2 GOBPs) and *G. mellonella* (2 GOBPs) (Jiang et al., 2021; Koutroumpa et al., 2021). PflaGOBP and PflaPBP subfamilies cluster together adjacently in the phylogenetic tree, indicating that they may evolve from the same ancestral gene and produce differentiation along with the evolution of species. Many studies showed that some GOBPs have similar functions with PBPs, which cannot only bind plant volatile but also sex pheromones. For example, AtraGOBPs have high affinities with their sex pheromone components in *A. transitella* (Liu et al., 2010). Han et al. (2022) found that SlitGOBP2 also takes part in sensing sex pheromone components using *in vivo* CRISPR/Cas9 technique. *P. flammans* is an oligophagous moth with a narrow host range. Thus, males are likely to find females on host plants. Therefore, sex pheromones and host plant volatiles may co-regulate the mate finding of *P. flammans* males. In this process, PflaGOBPs and PflaPBPs are possibly sensing sex pheromones and host plant volatiles at the same time.

As a binding protein family, CSPs are considered to have similar binding capacities with OBPs, same with host plant volatiles and sex pheromones (Pelosi et al., 2014; Li et al., 2020). However, increasing studies suggested that CSPs have a wider range of functions than OBPs, including non-olfactory functions (Benton et al., 2007; Yang et al., 2019). For example, in *S. exigua*, SexiCSP3 is demonstrated to be involved in the survival and reproduction by the RNA interference technology (Gong et al., 2012). PrapCSPs are conjectured to be related to the reproduction of *Pieris rapae* because PrapCSP7, PrapCSP18, and PrapCSP20 are evidently expressed in the reproductive organs (Li et al., 2020). Thus, CSPs are considered to take part in releasing some chemical substances in male glands, such as secreting vaccenyl acetate (Dyanov and Dzitoeva, 1995). Furthermore, CSPs may be associated with detecting nonvolatile chemicals. For example, CSPs are detected to have a high expression in the legs of *C. sacchariphagus* (Liu et al., 2021). In the present study, PflaCSPs are widely distributed in the whole branches of the CSP phylogenetic tree, and this finding is consistent with the hypothesis that CSP has a broad function in chemical communication (Benton et al., 2007; Yang et al., 2019). Among PflaCSPs, PflaCSP5/7/8/15/18, PflaCSP10/16/17, and PflaCSP2/6/11 are clustered in the same branch, indicating that they have recent gene differentiation.

A total of 16 candidate *PflaIRs* are recognized through the antennal transcriptome of *P. flammans* and are divided into 13 A-IRs (i.e., PflaIR2, PflaIR8a, PflaIR21a, PflaIR25a, PflaIR40a, PflaIR41a, PflaIR60a, PflaIR75c, PflaIR75d, PflaIR75p, PflaIR75q, PflaIR76b, and PflaIR93a), 1 D-IR (i.e., PflaIR85a), 1 LS-IR (i.e., PflaIR87a), and 1 iGluR (i.e., PflaGluR1). A-IRs and LS-IRs ordinarily belong to a conservative subfamily among different lepidopteran species. By contrast, D-IRs have variable gene numbers that contribute to the difference in IR numbers (Liu Y et al., 2018; Zhu et al., 2018; Wang et al., 2021; Yin et al., 2021). The phylogenetic tree analysis shows that all PflaIRs can be separated into 15 IR orthologous clades, including IR7d, IR8a, IR21a, IR25a, IR40a, IR41a, IR60a, IR64a, IR68a, IR75, IR76b, IR85a, IR87a, IR93a, and iGluRs. PflaIR8a and PflaIR25a are clustered into the IR8a/25a subfamily, and PflaIR76b is clustered into the IR76b subfamily, which is composed of IR co-receptors. Previous studies showed that IR co-receptors are highly expressed in antennae and other tissues and can sense carboxylic acids, that is, 3-methylpentanoicand hexanoic acids, and temperature variation (Ni et al., 2016; Zhang et al., 2019; Koutroumpa et al., 2021). Moreover, the *IR75* gene family exhibits the substantially female-biased expression in the antennae of *A. yunnanensis*, and the expression is upregulated extensively after mating in *H. armigera.* The IR75 may be related to mating and spawning behavior (Liu N et al., 2018; Li H. et al., 2021). Therefore, PflaIR75d, PflaIR75c, PflaIR75p, PflaIR75q, and PflaIR2 may have the same function, but validation is needed.

A total of 14 candidate *PflaGRs* are identified in the *P. flammans* transcriptome. GRs can be categorized into fructose, sugar, CO_2_, and bitter receptors by differentiation in function (Tian et al., 2018). Some studies found that sugars and sugar alcohols can influence the host plant search and the oviposition behavior of female *C. pomonella* (Lombarkia and Derridj, 2008). In the present study, PflaGR8 and PflaGR12 are clustered into fructose receptors that hint the two PflaGRs may have a reaction for fructose the same as BmorGR9 in *B. mori* (Sato et al., 2011) and SlitGR9 in *S. littoralis* (Walker et al., 2019) on fructose detection. Two PflaGRs could mediate internal nutrient sensing in the brain, such as DmelGR43a of *Drosophila melanogaster* (Miyamoto et al., 2012). Moths usually use CO_2_ as cues to detect the floral food source, and CO_2_ receptors play an important role in this process (Thom et al., 2004). Most moths have 2–3 CO_2_ receptors, such as *A. yunnanensis* (Li G.-C. et al., 2021), *S. littoralis* (Koutroumpa et al., 2021), and *Plodia interpunctella* (Jia et al., 2018). PflaGR9 and PflaGR13 are clustered into the branch of the CO_2_ receptor subfamily. CO_2_ receptors are usually highly expressed in gustatory organs, including proboscis, tarsi, mouthparts, ovipositors, and legs but have a low expression in the antenna (Guerenstein and Hildebrand, 2008; Koutroumpa et al., 2021). The low number of GRs identified in the current study may be explained in this respect.

In *S. insularis*, *H. assulta*, and *D. abietella*, *SNMP1* and *SNMP2* are highly expressed in the antenna of male and female adults and are speculated to participate in pheromone detection (Yang et al., 2019; Li H. et al., 2021; Wang et al., 2021). In *P. flammans*, PflaSNMP1 and PflaSNMP2 are closely clustered into lepidopteran SNMP1 and SNMP2 branches by the evolutionary tree. Many expression studies showed that SNMP1 has a wide association with pheromone sensing (Nichols and Vogt, 2008; Gomez-Diaz et al., 2016; Liu et al., 2021). However, some studies considered that SNMP1 is not necessary for pheromone sensing (Cassau and Krieger, 2021). Therefore, the function of PflaSNMP1 in the sexual communication of *P. flammans* needs to be further studied. In addition, two PflaSNMPs are recognized in *P. flammans*, whereas SNMPs are phylogenetically partitioned into three groups in most lepidopteran insects in a recent study (Zhang et al., 2020). Therefore, subsequent verification is needed to prove whether SNMP3 exists in *P. flammans*.

ORs are the most essential part of insect olfactory recognition (Dani et al., 2011; Leal, 2013). The phylogenetic tree analysis revealed that PflaOrco is clustered into the conservative Orco branch, indicating that it may be Orco in *P. flammans*. PflaOrco may also become the most highly expressed *OR* in *P. flammans* antennae, such as in *P. saucia* (Sun Y.-L. et al., 2020), *S. litura* (Wu et al., 2013), and *Lymantria dispar* (Lin et al., 2015), in which Orco is highly expressed in male antennae. By contrast, the *Orco* of some moths, such as *M. sexta* (Howlett et al., 2012), *S. cinerearia* (Liu et al., 2020), and *E. grisescens* (Li et al., 2017), shows high expression in female antennae. The olfactory function of *Orco* can be further confirmed using the RNAi technology and EAG response assays. For example, the expression quantity of *GmolOrco* and responses to sex pheromones show significant reduction in male *Grapholita molesta* after injecting dsRNA (Chen X et al., 2021). Fandino et al. (2019) used CRISPR-Cas9-targeted mutagenesis to knockout *MsexOrco* and showed that the foraging and oviposition behavior of *M. sexta* are affected. The aforementioned research can provide a reference for the functional verification of *PflaOrco*.

In the phylogenetic tree, 15 PflaORs (i.e., PflaOR21, PflaOR25, PflaOR27, PflaOR29, PflaOR35, PflaOR37, PflaOR40, PflaOR42, PflaOR44, PflaOR49, PflaOR51, PflaOR60, PflaOR61, PflaOR62, and PflaOR63) are found to be clustered together with three PR clades. Thus, these 15 PflaORs are screened, and two PflaORs (i.e., PflaOR36 and PflaOR41), which are close to the PR clade, are added as candidate PRs to conduct the tissue expression. The results of RTq-PCR analysis show that *PflaOR21*, *PflaOR27*, *PflaOR29*, *PflaOR35*, *PflaOR37*, *PflaOR40*, *PflaOR42*, *PflaOR44*, *PflaOR60*, and *PflaOR62* have sex-biased expression in male antennae and may be involved as specificity-sensing sex pheromone compound. These results are similar to those of *CpomPRs* (i.e., *CpomOR1*, *CpomOR2a*, *CpomOR5*, and *CpomOR7*) in *C. pomonella*, in which *CpomOR2a* and *CpomOR5* respond to an important component of *C. pomonella* sex pheromone (Tian et al., 2021). Classical (i.e., *PflaOR27*, *PflaOR29*, *PflaOR44*, *PflaOR60*, and *PflaOR62*) and novel (i.e., *PflaOR21*, *PflaOR35*, *PflaOR37*, *PflaOR40*, and *PflaOR42*) PR clades show male sex-biased expression in the antennae. Yuvaraj et al. (2022) found that the PRs of both clades of light brown apple moth show no difference in acetates and both isomers, behavioral attractants, and behavioral antagonists. Two clades have evolved convergently on the ability to detect similar compounds. By contrast, *PflaOR36*, *PflaOR49*, *PflaOR61*, and *PflaOR63* reveal female-biased expression in the abdomens and antennae, and this finding is similar to *HillOR32*, *HillOR44*, *HillOR56*, *HillOR57*, *HillOR60*, and *HillOR66* in *Hermetia illucens* (Xu et al., 2020). This finding suggested that the four *PflaORs* act on the male-released pheromone detection, localization of oviposition sites, and oviposition for females. *PflaOR25*, *PflaOR41*, and *PflaOR51* have almost similar expressions between female and male antennae, such as in *G. mellonella* (*GmelOR13* and *GmelOR50*) (Wang et al., 2021) and *P. saucia* (e.g., *PsauOR43* and *PsauOR54*) (Sun Y.-L. et al., 2020), and are likely to function in food source odor perception. Some *PRs*, such as *BmorOR9* of *B. mori* and *HvirOR6* of *H. virescens* (Krieger et al., 2004; Wanner et al., 2007), also display the no sex-biased expression pattern. *PflaOR25*, *PflaOR41*, and *PflaOR51* may be similar to these PRs. *PflaOR29*, *PflaOR36*, and *PflaOR37* can be expressed in nonantennal tissue, indicating wide functions not limited to odor recognition, and odor recognition may be happening in other tissues besides the antennae such as proboscis, palpae, and ovipositor.

In recent years, many sex pheromones of lepidopteron insects show practical application in the monitoring of pest populations, mass trapping, mating disruption, and push–pull strategies (Rizvi et al., 2021). In accordance with the response spectrum of PR and combined with behavior experiments, many sexual behavior antagonists can be screened to interfere with the courtship behavior of target lepidopteran pests, including *S. litura* (Zhang et al., 2014), *Helicoverpa armigera* (Chang et al., 2017), and *Epiphyas postvittana* (Yuvaraj et al., 2022), and have potential applications in developing new control methods (You and Zhang, 2021).

## Conclusion

In this study, a full-length transcriptome and an antennal unigene transcriptome of *P. flammans* antennae were formed. A total of 166 candidate olfactory and gustatory genes, including 58 *OBPs*, 19 *CSPs*, 59 *ORs*, 16 *IRs*, 14 *GRs*, and 2 *SNMPs*, were identified. Their evolution and phylogenetic relationships with those of other lepidopteran insects were elaborated. The expression profile analysis of candidate PflaPRs revealed tissue-specific and sex-biased expression in *P. flammans*, indicating their probable important role in olfactory recognition processes, such as sexual communication and host recognition. This research filled the gap in olfactory and gustatory genes of *P. flammans* and provided basic data for future functional molecular mechanism studies on *P. flammans* olfaction.

## Data Availability

The datasets presented in this study can be found in online repositories. The data presented in the study are deposited in the repository: https://www.ncbi.nlm.nih.gov/, accession number:PRJNA806455.
