# Supplementary material for: Identification of Chemosensory Genes, Including Candidate Pheromone Receptors, in Phauda flammans (Walker) (Lepidoptera: Phaudidae) Through Transcriptomic Analyses
Source: Front Physiol. 2022 Jul 1;13:907694. doi: 10.3389/fphys.2022.907694 (PMC9283972; doi:10.3389/fphys.2022.907694)
Supplement: Supplementary file 1 [file Table1.DOCX]

## >PflaOBP1

ATGAAGATAATTATTGTCTTATGCGCGTTCTTCATCACATATGGTACATGCGAACAGATGCGTCTTAAATTGAGACCAGATTTAGAAGCAAAAATGGAAGAATCATTCAAAAGTTGTGTTGAAAAAACAGGCGTCGACCCGAAATTGATTGAATACATCAGGCATCTCTACTTCCCTGATACTATTGGAATGGAAGAAATAGTGACATGTGTTACTCAGTATGAAGGCATTATGGATTCTAATGGCCATTACAATGTTGATGCCTTGCTTAGTTACTATCCTGATAATGTTAACTTAGAGCCAGTTAGAGAAGCTTTGACGGAGTGCAACAAGCTTATGGGTCGAACTGTTGGATCGACTGCAGTAATGACTATCGGTTGCTATTATCAGAAATCTCCTGTGGTCATGATGCCCTAA

## >PflaOBP2

ATGAAACATCTTCTCGTTCTGGTTCTATGTGTTGTATTAACAATTTCTGGTGTTTTTGGTAAACAGGCAATTGTTAACTTATCTGGAGCTTCACAAGAAAAAGTGAGAACAAATGCCAATTTATGTGTCAAAGAAACGGGAAATAATGAAGATGTACTTGAAGTATTTAAAACTTTAGATTTGTCAAATGAAAGTCCCGGCTCTAAGTCATTTATACACTGTCTAGTATCAAAAATAAATGTAGTCAAAAGCGATGGCACTATGAATATAGATTTCATACTAAGATTCTTCCCAGATGAATTCAAGAAAAGTGTTAAAAATGTTATCGAAATATGTAACAATGAAAATAAAAGCAATAGTCCGGTCGACACAAGCTTTAATGTTATGAAATGTTATCTTAAGAACTCACCAGCTGTTGTGACAATTTGA

## >PflaOBP3

ATGACGAGACAACAACTGAAAAACTCATCAAAAATGCTGAAAAAGAATTGCATGGCCAAAAATGACGTCACTGAAGATCTGATTGGAGACATAGAGAAAGGAAAATTCATCGAGGACAGGAAGGTGATGTGCTACGTGGCCTGCATCTACCAGATGTCGCAGATCGTAAAAAACAACAAACTGAACTACGAGGCGTCAATAAAGCAAGTGGACATCATGTACCCGCCGGAGCTGAAGGATGTGGCGAAGAAAGCTATCGAGCACTGTAAAGATATCAGTAAAAAGTACAAGGACTTGTGTGAGGCGTCGTACTGGTCGGCCAAGTGCATGTACGAGTTTGATCCCAAAAGTTTCATATTTGCGTAA

## >PflaOBP4

ATGCGACTGCTGGACGTGCTGGCCGTGCTGCTCCTGGAGTTGCTGACGGCGCTGTCGCTGTGTGACGCGATGACCATGAAGCAAATAAAGAACACCGGCAAGATGATGAGGAAATCGTGTCAACCGAAAAATAACGTTGAAACCGAAAAAATCGACGCAATTCAAAACGGCGTGTTCATCGAGGAGAGGGAGGTGATGTGCTACATGGCCTGCGTCATGAAGATGACCAACACGGTGCGTGTCGAGATGCGCGTGACGCGGGACCAACTCAACAATAACTCAAGTCTTGTCTTTCTCAACCACTTTAAAGCTTCTTTTCTTTATCTTCATATTGTTCACGTTGGCACCTCCCCGCACCGCGCCGCCCCGCGCCGCCCCGCGCCGCCCCAGCTCGACACACATCTCAACCCAATGTTCAACATATCTATGACGTATTTATTACCTTTGGTAATGAGGTCTTTCAAGTGTGCATGTTACATTTTTATTTGA

## >PflaOBP5

ATGAAGTATATTCTTTTGATAAATTTTTTAACGCTAATTGTACAGTGTTACAGTTTGAAACAGGTGAAAGTCCATACAGATTCCAAGCACATTGAAATAATGAAATCTGTTATACTTCAATGTGCAAATAATAATGTACTAGACGTAATGGAACAGTTCCGCGAAGGGTACCTAATCGACACTCCTGACATGAAGGACTTTATGTACTGTTTCGATCTAAAAACCGGGGCTGTAAATGAAGATGGATTTTATGATATTGAAAAAATAGTCGACTATTACACATCAGAATCTGATAAAGAACAAGTTAGAGCTGCTATGAAGGAGTGCGTCAAAAAGGAAGGAGAAGATATCCCTTCTAGAGTGTTTGAAATTGAGAAGTGTTATTATGCAACAGTTCCCGTAATTGTCGTATTATAA

## >PflaOBP6

ATGGCACCACTGCGCACGTCGTCCCTGCTCGCGCTGCTCATCCTGGCCGTCACGTGGCGCAGCGTGCTCGCGGACCCGGCGTTCTCTCCGGAGCAACTCCAGAAACTGAGTATCCAGTACTTGCCGGTCATCACCGAGTGCCTCACGAAGTACCACACCAACCTTGATACTGCCGTCTCTGGAGTTAAGGACTCAAAATCCTTTCTGGGTATCAACCCATGCGCTTTCGCTTGTTTCCTGAAGAAAACTGACATGATGGATGAGAACGGTCTGCTGAGGAAAGGGAAGACATTAGAGAAGATCAAGGAAACTGTCAAAAGTGAAGAAGATTATGCTGCTATGAAAAATTTGTTGGAAACTTGTGAACCAGTGAACTCCAAGCCCGTGAGCGACGGCGCCGCCGGCTGCGAGCGCGCCGGCCTCGCCGTGCGCTGCTTCATCGACAACAAGGGATTCGACATATTCCGATTCTGA

## >PflaOBP7

ATGATTCGAAAAATTAGTGCACTGTTATTTTGTTTTTATATGTTCGGCATTTCCCTGAGTGACAGCGCCATTTCAAATGATAGTGAGACAAGATGCAAAAGTCCTCCGACAGCTCCACAGAAAATAGAAAAAGTCATCACCTTGTGTCAAGATGAAATCAAAATATCCATATTGAGAGAAGCCTTAGACGTGATTAAGGAGGAGCACACAATGCCAGCACAAAGGAGACGCAACAAAAGAGAGGTGCCGTTCACACACGACGAAAAAAGGATCGCAGGATGCCTGCTACAGTGCGTGTACAGAAAAGTGAAAGCTGTTGACAGCTTTGGTTTCCCCACTCTGGAAGGTCTGGTGGGCTTGTACTCCGATGGAGTAAACGAACGCGGCTACTTCATGGCGGTATTAGAAGCTTCCAGGGAATGTCTTATGAGAAACCATGAAAAATTCTCCAGGACCGTACCCATGGATAACGGCCGCAATTGCGATGTTTCTTTCGATATCTTCGAATGCATCTCTGACCGTATCGGCGAGTACTGCGGCTCCAACGGACTATGA

## >PflaOBP8

ATGTGTTCGATGAAATTTTATATTTTATTTCTTTTTCTAACTCTGTTTGATATTCATGCCCTGAATTGTAAATCAGAAGGAGGTCCACAAGAATCAGAACTTAAATCTATATATTCATCATGTTTAAAGAAGCAAGAGGGCAGAAATTCGAGTAACGATGAACATTTTGGAAGCCATTCCGATGATGAAGACACTCAGTCCCACTCTAAAGAACCTAGAGGACGGCACCGACCTTGGGACAGAAGCGGTATTAGAGACGAATACGACAGAGAAGATACTAAGCAAAACAGACGAAATAGAAATGAACGAATCGGAGAAGGCAAAATGAAAACAGACGGCAGAATGCGAAGTCGAGAAGATGATATTGGAAGTGAGGATATGCGAAACCAGTGGAACAATCACAATCAAAATAATGGTATGGGTTTTAACGATAGAATGGGCAGTGGAAGTGGTAGGTTTGGAAGCAACGGAGACGATTTCATGCAAAATGATCAATTTAGAAATCAAGACCAGCAGTACAACGCCTACGGTTCAAATAACAGAGATAGTAATAACTATAACCATCGTTACAAACGAGAAAAACGCTTAGAGATGAATTCAGGACACCGAAGTCAATATAACCCAAACTCGCCCTCGTCGAGATCACCTTGGCGTGAAGATTCCCAGAACAGCGGTGAAAGAAATTCGTCTAATAGAGAGAATAGCATGAACAGAGATGTTGATAGCAAAGCTTGCGCGCTTCATTGTTTCCTAGAGGAGCTGGAAATGGTAGGAGATAACGGCTTACCTGATCGTTACCTAGTTACCCATATAATAACCAAAGATGTCGATAATGAGGATTTGAAAGATTTTCTTCAAGAATCGATTGAAGATTGTTTTCAAGTACTGTATAATGAAAATACAGAGGATAGATGTAAATTTTCCAAAGACTTGATGATGTGTCTGTCAGAGAAAGGAAGAGCAAATTGCGACGATTGGAAAGACGATATACAATTTTAG

## >PflaOBP9

ATGATTCTAAACTGGTGGCTGTGCTGCGTGCTTATGTTGCCTCCCCTACTGGGTGCAGCTGCGGGTGACGAGGCCCCGGTCCCTGAGAGCCGCCAGAAGACTCTGGCAAGTCAGTTCGTCACGTACGGAGCAGAATGCCTCTCCGACAACACTTTGTCCTTGGAAGATATAAGAGCCCTACAATCATATAAGGCGCCCAAAAGTCACGAGGCTTCCTGTTTTTTTGCATGCATTTTGAAGAGATCGGAAATGATGGACGCAGACGGCAGCGTGTCTCCTAACAAAGTAGTGGAAGTCGTGCGAGCTGTGTGTCGCGGAGACGAGGAGCTGGAGGCCGCGCAGCGCGCGCTGCGACTGTGTGTCCGCGTGAACGAAGTAGAAGTGAGTGACGGTGTTGCTGGGTGCGAGCGGGCCCGCCTGCTTCTGGGCTGCGTGTTGGAGCTGGCGGACCCCGGCACCAGCGCCGTCGGCACCAGCCAGCGCCTGACCGCCACCTCGTCACCGGCGCAGGTAGAGACGGGCTTCAGTCTCAGGAACATAGTTCAGCGAGTCATCCAGCAAGTGATACGTCTGGTTTGTTTTGGTTTGAGCAAAGACTTCCTGCCCGGAAGAATCATCTCCAGGATCATTCCTGGGCTCGACACTATCTCTGCCTTATGCTCCTTTATAGTGTAA

## >PflaOBP10

ATGTTTATTAAAAACATATTATTATTTACTATTAGTTATTTGGTTTTGGTGGAATCTGCTTTTGTAAATAACCTACAAAAATGCAGCATCCACGATGGAGAATGTCACAGGGACTTAACGCAATCTATTATTACCGACATAGGGAAAACAGGTATTCGTGAGTTGGGCATCCCACCAATAGACCCAATCCATTTGACGAATATAACCGTGTCAGTGGCTGGCCTTTTGGATATAACTCTTGTCGATGGATATGCCAAAGGTGTCAAGGATTGTGTCATTGAGCGATTCGATACTCACATCGAAAACGAGCGAGCTACAATGGAACTAACTTGCGATATACTCATCAAAGGACATTACAGGCTGTTCTCAGACAGTCCCCTCGTCAAAGGAATTCTTGGATCTGATTCAGTTCACGGCGATGGAAACGGCAAAGTTAAGATTGAAAAACTACACGTCAAATTCGAATTCTTCTACTATGTGACCAGGAGAGACGGAGAGGTCTACATTAAATGCAAAAACGATGTTCTTAGTTACACTTACAAAATCAATGGTAAAACTACTTTCGGGGCTAACAATATATATCTTGGAAAAGAAGAAGCTAGTACCCAGGTAGTGGATTTGTTGAACACCAACTCAAAAATGGTCATGGATCTAGTCGGTAAGATATTCATGGACAAAGCCATGGAAGTCGTGTATGTTTATCTCCACAAATTCTTTGATCTCGTGCCTACAAAATACTACATCACTGAAGATCTATCTCAATATATAAAATCGTAA

## >PflaOBP11

ATGTCCTTAGCAACGGTTCCGCTTCTTCTCATATTCACAATCTTCTGCACCGGTCAGAAAGAACAACCTGTGTTCAGCGACGAAATCAAAGAAATAATCGAACTTGTTCACAACGAATGCGTCGGCAAGACCGGCGTCGCTGAGGAGGACATAAAGAATTGCGAAAACGGCATATTTAAGGAGGACGAGAAATTGAAGTGCTATATGTATTGCCTTCTCGAGGAAGCCAGTATCGCGGATGAGGAGGGCGTAGTGGATTACGAGATGCTGGTCAGCTTGATACCTGAAAAATACTATGATCGTGTCACCAAGATGATATTTGCATGCAGACATTTAGACACGCCTGATAAGAACAAATGTCAACGAGCTTTTGATGTCCATAAATGTTCGTATGAAAAGGACCCCAATGTAAGTAATTAA

## >PflaOBP12

ATGGCAAAAGCAATCGAGTGCAACCGTGACATCCCAGTACCGATGGCAGAGCTCGCCATGCTGGAGAAGCACAAGATGCCGGACAGCTCGGCTGCTAAATGCTTGATGGCATGTGTTTATAGGAAAGTCCAATGGATGGACGACAAAGGCATGTTTGATGAAGTCTCGGCCAACAATATGGTAGCGAAGGAGCACACCGACGACGCTGTAAAACTGGAAAACTCTAAAAAGTTATTTGCGTTGTGCAGAAAAGTGAACGAGGAGGCCGTGAGCGACGGCGACAAGGGCTGCGACAGGTCTGCGCACTTGTTCCAGTGCCTGACCGACAACGCTCCTAAGATGGGATTCCAGCTGTAG

## >PflaOBP13

ATGGCTATGTTTGTGTGGTTGAAAGTTTTGTGCGTTTTGAATTTAATTTACTTCGCGTCAGCTGAAACTGACAAGGATGCATCGCTTAGCTCCATCGTTCACAAAAGCCTTATTTTAACAGCCAAAACGTGTATGAAACGTGTAAATGCGTCCGAGACAGACTTGGAGCATTTGCGCAAGAGTCCACCATTTCCTGAGAGAGGTGCTTGCATAGTAAAATGTTTATTGGAGAAAATCGGAATCGTAAAAAACAATAAATATTCGAAGAACGGCTTCCTGACTGCAATAACGCCTCTCGTGTTCACCCACAAGAAGAAATTAGAACATATGAAGAATGTGGCTGAAACCTGTGACAAAGAGATAAGCCACGACGTATCGCCCTGTGAGCTCGGGAATGAAGTAACCACAT GCATTTTGAAATACGCT

## >PflaOBP14

ATGTCATCTACCAAAATGTTCGTCGATATAAACCGTCCCGCCGCGACGTCGCCGTCAGTAGCGTCGCCACGCAGCCGTCGCCCGCCCGCAGCCTGCACCACGCACCACGCACCACGCACCATGTTGTGGCTCCTTGCACTAGCCGCCGGTTCGTTTGTCGTCATACCAAACTTGACTCACGCTATGACGGCGGAACAGAAGGCAGCCATACACGAGCACTTCGAGACGATAGGCGCCGAGTGTGTGGGCGAGAACCCGATCACAGAGGACGACATCGCGAGCCTCCGAGCGCGCGAGCTGCCGGCCGGCGAGCACGCGGCCTGCTTCCTGGCCTGCGTGTTCAAACGGATCGGAGTGATGGACGACAAGGGCATGCTGCAAAAGGAGTCCGTATTAGAACTTGCAAAGAAGGTATTCGATGACGCAGAAGAACTGAAAGCGATCGAAAATTACCTTCACTCGTGTTCACACATCAACTCTGCGCCGGTGAGCGACGGCGCCGCCGGGTGCGAGCGCGCGCTGCTCTCCTACACCTGCATGACGGAGCACGCGGCGCAGTTTGGCTTCGAAGTTTGA

## >PflaOBP15

ATGGTTTATATTTTTTCATGTTTTCAGGCCATCACTGAAGAGCAAAAGAGCCACATACAGTCCAAGTTTCTAGCAGCGGGAGCCGGGTGCATAGCTGATCATCCACTCACTTCTGATGACATCAGTAGTTTCAAGGAAAAGAAACTGCCAGACGGCGACAACGCGGCGTGCTTCGCCGCCTGCCTGTTCCGGAGGATCGGTCTCGTGAGTAACGTTGTCGCGATAAGGCCGGTGTCCACGACGGTCACAGAGCACCAACTAGCAGACACCCACGCACCCACGCACCCACTGATGCAACATCTACATACATGA

## >PflaOBP16

ATGTCTTACTCTAGAACGTTACTTAGTTTTCTCCTTATATTAACATTGCTTGACAAAGCATTTGTTTCTTCAATGACTAGGCAGCAAATAAAAAACTCGGGGAAAATATTGAAGAAGACTTGTATGCCCAAGAACGAAGTCACAGAAGAACAAGTTGGTGCAATCGATCAGGGGAAGTTCTTGGAAGAGAGGAATGTGATGTGCTACGTCGCCTGCATATACTCGATGTCGCAGGCGGTGAGTGCTGCTGCCGGGACTCGGTCTGTGCTCGCAGCCGCAGTCACACACTCTACTACACGGAATATTATACGTGAGGCGGCCTTACGAACGCCACCGCCTCGTATCTTCCCATGGGTGTTGTAA

## >PflaOBP17

ATGGTCGAGCTGTTACGGACATTGTGCTGCGTCTTCAGCGTGCTCGCGGTCCTCAGTTTGAGTCACGTCCAGAGTTTAAGTAACGAAGAAATTGAAAAACTCAAGGGTGAGTTCTTGCCGATACTGGAGGAGTGTTCCAAGCAGGGCGGCATCACGGTCGAGGCGCTGAAGGAGGCCAAGCAGACGGGCCACGCGGACGCCGGCGTGCACTGCGTCATGAGCTGCGTCTTTAAGAAAACGGGCCTCATAAAAGAAGACGGAACGTTCGACAAGGAGGCGGCGCTGGGCAACGTCAAGCACTTGCTGTCCGACGCAGACGACATCGCCAGAACCAGCGAGCTGATGAGCGCCTGCGAGTCCGGTACGTACGGAGCGTACGGAGCGTACGGAGCGTACGGAGCACCGCCAGCCCTTGCCGGGCATTGGGCAACACATTAG

## >PflaOBP18

ATGTCCTTAGCAACGGTTCCGCTTCTTCTCATATTCACAATCTTCTGCACCGGTCAGAAAGAACAACCTGTGTTCAGCGACGAAATCAAAGAAATAATCGAACTTGTTCACAACGAATGCGTCGGCAAGACCGGCGTCGCTGAGGAGGACATAAAGAATTGCGAAAACGGCATATTTAAGGAGGACGAGAAATTGAAGTGCTATATGTATTGCCTTCTCGAGGAAGCCAGTATCGCGGATGAGGAGGGCGTAGTGGATTACGAGATGCTGGTCAGCTTGATACCTGAAAAATACTATGATCGTGTCACCAAGATGATATTTGCATGCAGACATTTAGACACGCCTGATAAGAACAAATGTCAACGAGCTTTTGATGTCCATAAATGTTCGTATGAAAAGGACCCCAATATATACTTCCTGTTCTAA

## >PflaOBP19

ATGACACAACGCCGAAGTATGACGGGCTACACTTGGCTATTTGTTTTCGCGGCATTATTTCAGAACATCCACTGCCAAAATGCCAATGTGCCCGAAAAATGCAGAGGGCCACCAGAAGGTGTGAAATCTGGTCCTCACGAATGCTGTCAGAAGAAACCATTCTTTGAAGAAGACGCTCTAAAAGAGTGCGGATTCGAAAAGCCAGATACATCTGGACCTCCACCACCGCCTCCAAGAGGACCACCAGACTGCGTAAAAGAGACTTGCTTACTGAAAAAGTATGGTCTGATGAAAGATGACACCAACGTTGACAAGGACGCCGTAGCGAAGCATTTGGACAAATGGGGCGAGGACAACACGGAATATGGTGATCTGGTGCCAGCACTTAAGGAGAAATGCGTTTCTGACAATCTACCCGGCCCTCCGAAAATGTGCAGCGCCGTCAAATTCATGCATTGCATTGGTCACTTCGTATTCGAGAACTGCAAATGGAATGACACGGAAGACTGCAACAAAATAAAGAGTCACCTTGAAGAGTGCAAGCAGTATTTTCCTAAACATCCATAA

## >PflaOBP20

ATGTTTATTAAAAAAATATTATTATTTACAATTAGTTATTTGGTTTTGGTGGAATCTGCTTTTGTTAATAACCTACAAAAATGCAGCATCCACGATGGAGAATGTCACAGGGACTTAACGCAATCTATTATTACCGACATAGGGAAAACAGGTATTCGTGAGTTGGGTATCCCACCAATAGACCCAATCCATTTGACGAATATAACCGTGTCTGTGGCTGGCCTTTTGGATATAACTCTTGTCGATGGATATGCCAAAGGTGTTAAGGATTGTGTCATTGAGCGATTCGATACCCACATCGAAAACGAGCGAGCTACTATGGAACTAACTTGCGATGTACTCATCAAAGGACATTACAGGCTGTTCTCAGACAGTCCTCTCGTCAAAGGAATTCTTGGATCCGATTCAGTTCACGGCGATGGAAACGGCAAAGTCAAGATTGAAAAGTTACACGTCAAATTCGAATTCTTCTACTACGTAACCAGGAGAGACGGAGAGGTCTACATCAAATGCAAAAACGATGTTCTTAGTTACTCTTACAAAATCAATGGTAAAACTACTTTCGGAGCTAACAATATATATCTTGGAAAAGAAGAAGCTAGTACCCAGGTAGTGGATTTGTTGAACACTAACTCAAAAATGGTCATGGATCTTGTCGGTAAGATATTCATGGACAAAGCCATGGAAGTCGTGTATGTTTATCTCCATAAATTCTTTGATCTCGTGCCAACAAAATACTACATCACTGAAGATCTGTCTCAATACATAAAATCGTGA

## >PflaOBP21

ATGTTTATTAAAAACATATTATTATTTACTATTAGTTATTTGGTTTTGGTGGAATCTGCTTTTGTAAATAACCTACAAAAATGCAGCATCCACGATGGAGAATGTCACAGGGACTTAACGCAATCTATTATTACCGACATAGGGAAAACAGGTATTCGTGAGTTGGGCATCCCACCCATAGACCCAATCCATTTGACGAATATAACCGTGTCAGTGGCTGGCCTTTTGGATATAACTCTTGTCGATGGATATGCCAAAGGTGTCAAGGATTGTGTCATTGAGCGATTCGATACTCACATCGAAAACGAGCGAGCTACAATGGAACTAACTTGCGATATACTCATCAAAGGACATTACAGGCTGTTCTCAGACAGTCCCCTCGTCAAAGGAATTCTTGGATCTGATTCAGTTCACGGCGATGGAAACGGCAAAGTTAAGATTGAAAAACTACACGTCAAATTCGAATTCTTCTACTATGTGACCAGGAGAGACGGAGAGGTCTACATTAAATGCAAAAACGATGTTCTTAGTTACACTTACAAAATCAATGGTAAAACTACTTTCGGGGCTAACAATATATATCTTGGAAAAGAAGAAGCTAGTACCCAGGTAGTGGATTTGTTGAACACCAACTCAAAAATGGTCATGGATCTAGTCGGTAAGATATTCATGGACAAAGCCATGGAAGTCGTGTATGTTTATCTCCACAAATTCTTTGATCTCGTGCCTACAAAATACTACATCACTGAAGATCTATCTCAATATATAAAATCGTAA

## >PflaOBP22

ATGGAACTAACTTGCGATATACTCATCAAAGGACATTACAGGCTGTTCTCGGACAGTCCCCTCGTCAAAGGAATTCTTGGATCTGATTCAGTTCACGGCGATGGAAACGGCAAAGTTAAGATTGAAAAACTACACGTCAAATTCGAATTCTTCTACTATGTGACCAGGAGAGACGGAGAGGTCTACATTAAATGCAAAAACGATGTTCTTAGTTACACTTACAAAATCAATGGTAAAACTACTTTCGGGGCTAACAATATATATCTTGGAAAAGAAGAAGCTAGTACCCAGGTAGTGGATTTGTTGAACACCAACTCAAAAATGGTCATGGATCTAGTCGGTAAGATATTCATGGACAAAGCCATGGAAGTCGTGTATGTTTATCTCCACAAATTCTTTGATCTCGTGCCTACAAAATACTACATCACTGAAGATCTATCTCAATATATAAAATCGTAA

## >PflaOBP23

ATGCGACTGCTGGACGTGCTGGCCGTGCTGCTCCTGGAGTTGCTGACGGCGCTGTCGCTGTGTGACGCGATGACCATGAAGCAAATAAAGAACACCGGCAAGATGATGAGGAAATCGTGTCAACCGAAAAATAACGTTGAAACCGAAAAAATCGACGCAATTCAAAACGGCGTGTTCATCGAGGAGAGGGAGGTGATGTGCTACATGGCCTGCGTCATGAAGATGACCAACACGATGAAAAATGGTAAGTTAAACTACGAAGCGGCGATGAAGCAGATCGACCTGGTGCTGCCTGAGGAGTTGAAGGAGCCCGCCAAAGCGGCGGTGACGGCGTGCAGGCGGGTCGCGGAGGCGCACAAGGACATCTGTGAGTCGTCGTTCCACGTCACCAAGTGTATCCAAAGACACAACCCAGACATATTTTATTTCCCGTAA

## >PflaOBP24

ATGAAGACTTACATCGTTCTCGCCGTCTGCCTCGTCATGGCGCAGGCCCTCACTGATGAGCAGAAAGAGAAGCTCAAGAAGCACAGGACCGACTGTCTGTCGGAGACTAAGGCTGACGTTCAACTTGTCGACAAACTGAAGGCGGGTGACTTCAAGACTGAGAATGAACCATTGAAGAAGTATGCCCTGTGCATGTTGAATAAGTCCGAGCTGATGACCAAGGACGGCAAATTCAAGAAAGATGTTGCCTTGGCTAAAGTCCTTAATGCAGCTGACAAGGCCGCCGTTGAGAAGCTGATCGACGCCTGCCTGGCCAACAAGGGCAACACCCCCCACCAGACCGCGTGGAATTACGTCAAGTGCTACCACGAGAAAGACCCCAAGCACCCAATCCTAGTATAA

## >PflaOBP25

ATGGTCGAGCTGTTACGGACATTGTGCTGCGTCTTCAGCGTGCTCGCGGTCCTCAGTTTGAGTCACGTCCAGAGTTTAAGTAACGAAGAAATTGAAAAACTCAAGGGTGAGTTCTTGCCGATACTGGAGGAGTGTTCCAAGCAGGGCGGCATCACGGTCGAGGCGCTGAAGGAGGCCAAGCAGACGGGCCACGCGGACGCCGGCGTGCACTGCGTCATGAGCTGCGTCTTTAAGAAAACGGGCCTCATAAAAGAAGACGGAACGTTCGACAAGGAGGCGGCGCTGGGCAACGTCAAGCACTTGCTGTCCGACGCAGACGACATCGCCAGAACCAGCGAGCTGATGAGCGCCTGCGAGTCCGTGAACGAGCAGCAAGTGGACGCGGACGACGAGGCCTGCACCCGCGCGAAGCTGCTTCTGGAGTGCTTCATGCAGCACAAAGACCTGAGCATTTTCCCGCATTCTTAG

## >PflaOBP26

ATGGCCGAGCTGTTACGGACATTGTGCTGCGTCTTCAGCGTGCTCGCGGTCCTCAGTTTGGGTCACGTCCAGAGTTTAAGTAACGAAGAAATTGATAAACTCAAGGGTGAGTTCTTGCCGATAGTGGAGGAGTGTTCTAAGCAGGGCGGCACCACGGTCGAGGCGCTGAAGGAGGCCAAGCAGACGGGCCACGTGGACGCCGGCGTGCACTGCGTCATGAGCTGCGTCTTTAAGAAAACGGGCCTCATAAAAGAAGACGGAACGTTCGACAAGGAGGCGGCGCTGGGCAACGTCAAGCACTTGCTGTCCGACGCAGACGACATCGCCAAAACCAGCGAGCTGATGAGCGCCTGCGAGTCCGTGAACGAGCAGCAAGTGGACGCGGACGACGAGGCCTGCACCCGCGCCAAGCTGCTTCTGGAGTGCTTCATGCAGCACAAAGACCTGAGCATTTTCCCGCATTCTTAG

## >PflaOBP27

ATGACACAACGCCGAAGTATGACGGGCTACACTTGGCTATTTGTTTTCGCGGCATTATTTCAGAACATCCACTGCCAAAATGCCAATGTGCCCGAAAAATGCAGAGGGCCACCAGAAGGTGTGAAATCTGGTCCTCACGAATGCTGTCAGAAGAAACCATTCTTTGAAGAAGACGCTCTAAAAGAGTGCGGATTCGAAAAGCCAGATGCATCTGGACCTCCACCACCGCCTCCAAGAGGACCACCAGACTGCGTAAAAGAGACTTGCTTACTGAAAAAGTATGGTCTGATGAAAGATGACACCAACGTTGACAAGGACGCCGTAGCGAAGCATTTGGACAAATGGGGCGAGGACAACACGGAATATGGTGATCTGGTGCCAGCACTTAAGGAGAAATGCGTTTCTGACAATCTACCCGGCCCTCCGAAAATGTGCAGCGCCGTCAAATTCATGCATTGCATTGGTCACTTCGTATTCGAGAACTGCAAATGGAATGACACGGAAGACTGCAACAAAATAAAGAGTCACCTTGAAGAGTGCAAGCAGTATTTTCCTAAACATCCATAA

## >PflaOBP28

ATGGTCGAGCTGTTACGGACATTGTGCTGCGTCTTCAGCGTGCTCGCGGTCCTCAGTTTGAGTCACGTCCAGAGTTTAAGTAACGAAGAAATTGAAAAACTCAAGGGTGAGTTCTTGCCGATACTGGAGGAGTGTTCCAAGCAGGGCGGCATCACGGTCGAGGCGCTGAAGGAGGCCAAGCAGACGGGCCACGCGGACGCCGGCGTGCACTGCGTCATGAGCTGCGTCTTTAAGAAAACGGGCCTCATAAAAGAAGACGGAACGTTCGACAAGGAGGCGGCGCTGGGCAACGTCAAGCACTTGCTGTCCGACGCAGACGACATCGCCAGAACCAGCGAGCTGATGAGCGCCTGCGAGTCCGAGCATTTTCCCGCATTCTTAGTGACACTCCTAGCTGCTACCCGGACCACGACGACCACAGGGACCTCGAGCACCACGAGGACGACAGGACGCTCCGCCGCGTCGCACCCTCAGCAATGA

## >PflaOBP29

ATGGACACGTTCAAGATGTGTGCGTCAGGCTTCGTTTTGTTGTGTATTGTAGACCTCCTGGTCGCCATGACAAGACAACAATTAAAAAATTCCGGGAAGATAATGAAGAAATCTTGTCTGGCCAAACACGACGTAACCGAAGATCAAGTCGGGGAAATAGAACAAGGAAAGTTTATTGAAGACAAGAACGTGATGTGCTACATCGGTTGCATCTATACGATGACGCAAGTGGTTAAAAATAACAAGCTTAATTATGAAGCAGTACTCAAACAAGTAGACGCGATGTTTCCCGCAGAACTCAAAGAACCGGTGAAGGCCGCTGCAAAACATTGCAAACATATCGCTAAGAAGTACGAAGACTTGTGTGAAGCATCGTATCATACAGCTAAGTGCATGTACGATTTTGATCCAAAAAGTTTTGTTTTTCCATGA

## >PflaOBP30

ATGTGTTCGATGAAATTTTATATTTTATTTCTTTTTCTAACTCTGTTTGATATTCATGCCCTGAATTGTAAATCAGAAGGAGGTCCACAAGAATCAGAACTTAAATCTATATATTCATCATGTTTAAAGAAGCAAGAGGGCAGAAATTCGAGTAACGATGAACATTTTGGAAGCCATTCCGATGATGAAGACACTCAGTCCCACTCTAAAGAACCTAGAGGACGGCACCGACCTTGGGACAGAAGCGGTATTAGAGACGAATACGACAGAGAAGATACTAAGCAAAACAGACGAAATAGAAATGAACGAATCGGAGAAGGCAAAATGAAAACAGACGGCAGAATGCGAAGTCGAGAAGATGATATTGGAAGTGAGGATATGCGAAACCAGTGGAACAATCACAATCAAAATAATGGTATGGGTTTTAACGATAGAATGGGCAGTGGAAGTGGTAGGTTTGGAAGCAACGGAGACGATTTCATGCAAAATGATCAATTTAGAAATCAAGACCAGCAGTACAACGCCTACGGTTCAAATAACAGAGATAGTAATAACTATAACCATCGTTACAAACGAGAAAAACGCTTAGAGATGAATTCAGGACACCGAAGTCAATATAACCCAAACTCGCCCTCGTCGAGATCACCTTGGCGTGAAGATTCCCAGAACAGCGGTGAAAGAAATTCGTCTAATAGAGAGAATAGCATGAACAGAGATGTTGATAGCAAAGCTTGCGCGCTTCATTGTTTCCTAGAGGAGCTGGAAATGGTAGGAGATAACGGCTTACCTGATCGTTACCTAGTTACCCATATAATAACCAAAGATGTCGATAATGAGGATTTAAAAGATTTTCTTCAAGAATCGATTGAAGATTGTTTTCAAATACTGTATAATGAAAATACAGAGGATAGATGTAAATTTTCCAAAGACTTGATGATGTGTCTGTCAGAGAAAGGAAGAGCAAATTGCGACGATTGGAAAGACGATATACAATTTTAG

## >PflaOBP31

ATGTTTAAAAGATATTTGCTATTTTTGTTTCTATTTCAAGTTCTGACTGTTTCCATTGGGACTCTTCAGATAAATAACTATGTGCAGACCTGTGACAGACTCTCTCCAGAAACAAATGAATGTCTAAAAGAAGCGGTGATAAATGGCCTCGCTGAGTTATGCAAAGGTATTCCGGAGCTGGACATCCCACCAATTGATCCACATCATCAAGACGAAATCAAATTTAATTATAATGCCAATCAAATAATGCTTTCGGCGTTCCTGAGAGATGTCAACGTTTATGGCTTGAAAACTGCGAAGATACACAACGTAAGGCTACGAGCTGATGACGACTCTTTTCACATGGAGGTAGACGCGACTCAACCGAAGGTGTTGGTAAAGGGGAAATATAGCGCCAGTGGAGTCTACAACTCATTGAAATTGAAAGCAATTGGCAATTTCAGTGTTACCATGAAGAATTTAACATTCACTTGGAAACTCGACGGAAAACCGGAGGAAAGGGACGGCGAAACCTTTATGAAAATCACATCCTTTTACATGCGACCTCAAATAGAAAAGTTGATTTCCGCGGCGACAAACGATATTCAAGAAAGCAGGGCACTCACTGACCTAGCGGTGTCCGTCGCAAATCAAAACTGGCCTCCAATCTACAAGGAGTTATTACCACTGGCTCAGGACAACTGGAATACTGTAGGTACAGGGATTGCCAACAAGATTTTCCTTAAAGTGCCTTACAATCAATTGTTTCCCGTAGAATAA

## >PflaOBP32

ATGTTCAAAACATCATTACTTCTTTTGTTCGTATTAAAAGTTTCAACTGTTTGCTACGGGCGTATACAGATAAATAAATATATAAAAACATGTAACAGAATCTCGCCCGATGTAAATGAATGTCTTAAAGAAGCTATAAAACTCGGTATTGACACACTGTGCAAGGGTATACCGGAGCTGGACATCCCACCAATTGACCCCTTTCATGAAGACGTGATTAAACATGTGTATAATAAGAATCAAATTAGCCTCACTTCGTCCGTTACAGATGTCAACGCCTACGGATTCCAAAATGCGAAAGTTCACAACGTAAGGTTACGAGCTGATGATGACTCTTTCCACTTGGAGATAGACATGACAACACCAAAGGTGTTAGTGACGGGTAATTATGACTCCAGCGGTGACTTCAACTCATTGAAAATAAAAGCGATTGGCACCTTCAACTTTACCATGAAGGAATTAACGTATACGTGGAAACTCGACGGAAAACCAGTAGAAAGGGACGGTGAGACCTATATAAAAATCGCATCCTTTTACATGCACCCTCGCGTAGAAGGTTTGTCTATTGAGGCGACCAATGAAAATCCAGAGAGTAAACCTTTCACTGAGTTGGCCCTCTCCTTCGTGAATAACAACTGGGTCCCAGTCTACAAGGAGATATTGCCGTTTGCCCAGGAGAACTGGAATTCTGTCGGAGTCGAGATTGCTAATAAAATTTTCCTCAAAGTGCCGTACGACGAACTATTCCCCGTAGAATAA

## >PflaOBP33

ATGATATTTCTAATATCGTTATTGAGTATCATCTCTGTCGCATTTTGCAAAGAAGTGCCCTTTATCACAAAATGCCATCTGGACGATGAAGCCTGTATACTTAAGTCCGCGCAAACAGCCCTGCCTTTCTTCGTAAAGGGTTTGCCGGAATACAAAGTGAAAACCTTGGACCCGTTGAGCTTCAAAGTTATCGATGCCAGCACGGACAATCTCAAGTTCATCATGAAGAACCCAGTTTTTTATGGCACAACGAACAGCGTCATTAAGAAAATGAAAATGGAGGGTCGTAAGATGCAAAGTATCGTTGAATGTGACTTACTGAGGGGTGAGAGCGAGTACGAGGTGGATGGAAAGCTGCTTGTGTTGACCTTGAGCTCGAAGGGAATTGTGAAGACAACAATCCCAAAACTGATAATGACGATAGAAGCGGACTACGCAGACGTGGTGAAGAACGGTGAGACCTACTGGAAGATCAAGAGCTTTAAACACAAATACGAGATTCAGGGCAAGTGTACGACAGAGTTTGAAGACAAAGTGAGCAGTGAAGCCGTTGTGCGTACTGTGAAAGAGTTGGTAAAGACAAGTGGAAATGAGTTGCTTCTCGAACTGCTTCCTCCGATTATGGACTCAATCGTCAAGGAAATGGTGTCACAGATCAATCATTTCTTCTCGAGCGTACCCGCCAAAGACCTCAGTCTGGACTACTAA

## >PflaOBP34

ATGGAGGTAGACGCGACTCAACCGAAGGTGTTGGTAAAGGGGAAATATAGCGCCAGTGGAGTCTACAACTCATTGAAATTGAAAGCAATTGGCAATTTCAGTGTTACCATGAAGAATTTAACATTCACTTGGAAACTCGACGGAAAACCGGAGGAAAGGGACGGCGAAACCTTTATGAAAATCACATCCTTTTACATGCGACCTCAAATAGAAAAGTTGATTTCCGCGGCGACAAACGATATTCAAGAAAGCAGGGCACTCACTGACCTAGCGGTGTCCGTCGCAAATCAAAACTGGCCTCCAATCTACAAGGAGTTATTACCACTGGCTCAGGACAACTGGAATACTGTAGGTACAGGGATTGCCAACAAGATTTTCCTTAAAGTGCCTTACAATCAATTGTTTCCCGTAGAATAA

## >PflaOBP35

ATGGCAGAGTTAGCTCGCATGCTACGTGAGAATTGCGGTGAAGAAACTGGAGTCGATTTGTCACTTGTGGATAAAGTAAACGGCGGAGCTGACTTGATGCCCGACGCATCGCTGAAATGTTACATCAAATGCATTATGGAGACGGCGGGCATGCTGAGCGAAGGTGAAGTGGATGTGGAAGCAGTTATAGCTTTGATGCCCGACGCTATACGAGAGAAGAATGACGCCAATATCAGGAGCTGCGGAACTAAGAAGGGAGCAGACGATTGTGACACAGCATACCTCACACAGGTTTGCTGGCAGAAGGCCAACAAAGCTGACTACTTTTTAGTGTGA

## >PflaOBP36

ATGGTGCCCTACGGCAAGAGCACACATGACAAAGTGAACGACAAGTGGTCCTTCATCTGCCACCACGGCCCTGACGAGTGCTACGGTAACAAGATACAGGCGTGCATCCTGAAAGACAAGCATCTGTTGGACACGGACAAGATGGACCTCGTCATATGTCTGATGAGCCAAGCCGAACCTGACAAGTCTCTTGATACGTGTCTAGCCCAACACAACAAGCAGTCGGAGAGTGTGAAACTGAAGTCCTGTGCGTCAGGAGTGCAGGGCGACAACCTGCTCGCTGCCTACGGCGACAAGTCTGATGCCGTGCAGAAACCGTTCACCTTCGTGCCGACCGTTGTCATCAATGAAAAATTTGACTTCGCTGTTCAAGATGAAGCCGTCAAAGATCTGAAATCAGTCGTCTGTCGTGTGTCGCCAACAAAGCCTGCTGTCTGTGCATAA

## >PflaOBP37

ATGGTGGAAGACGAGGCGCGAAGTGGGAGACCAGTCGAGGTGGAGACTGACGCGAATGCGCAGCGTGTTCGCGCCCACAAAGAATTCGTTCCACCGGGGAAAACTGTGAATCAAGTCTACTATCGCCAAGTACTCGAAAGATTGCGAAAACGAGTCCGCAGGGTGTGCCCAGACATCGCTCATAACTGGATCCTTCATCACGACAATGCGCCGTGCCACACCGCCCTCAGCGTGTCCCGGTATTTGGCCTCTAAAGGGATCGTCGTGTTGCAACAGCCGCCTTATCCGCCCGACATGTCGCCCTGTGACTTTTTTGTTTCCTAG

## >PflaOBP38

ATGAAACTATTATTGTTGATGTTCATTGTGCTTTGTGCCAGTGTTGATACTATGACGAGACAACAACTGAAAAACTCATCAAAAATGCTGAAAAAGAATTGCATGGCCAAAAATGACGTCACTGAAGATCTGATTGGAGACATAGAGAAAGGAAAATTCATCGAGGACAGGAAGGTGATGTGCTACGTGGCCTGCATCTACCAGATGTCGCAGATCGTAAAAAACAACAAACTGAACTACGAGGCGTCAATAAAGCAAGTGGACATCATGTACCCGCCGGAGCTGAAGGATGTGGCGAAGAAAGCTATCGAGCACTGTAAAGATATCAGTAAAAAGTACAAGGACTTGTGTGAGGCGTCGTACTGGTCGGCCAAGTGCATGTACGAGTTTGATCCCAAAAGTTTCATATTTGCGTAA

## >PflaOBP39

ATGATTCGAAAAATTAGTGCACTGTTATTTTGTTTTTATATGTTCGGCATTTCCCTGAGTGACAGCGCCATTTCAAATGATAGTGAGACAAGATGCAAAAGTCCTCCGACAGCTCCACAGAAAATAGAAAAAGTCATCACCTTGTGTCAAGATGAAATCAAAATATCCATATTGAGAGAAGCCTTAGACGTGATTAAGGAGGAGCACACAATGCCAGCACAAAGGAGACGCAACAAAAGAGAGGTGCCGTTCACACACGACGAAAAAAGGATCGCAGGATGCCTGCTACAGTGCGTGTACAGAAAAGTGAAAGCTGTTGACAGCTTTGGTTTCCCCACTCTGGAAGGTCTGGTGGGCTTGTACTCCGATGGAGTAAACGAACGCGGCTACTTCATGGCGGTATTAGAAGCTTCCAGGGAATGTCTTATGAGAAACCATGAAAAATTCTCCAGGACCGTACCCATGGATAACGGCCGCAATTGCGATGTCTCTTTCGATATCTTCGAATGCATCTCTGACCGTATCGGCGAGTACTGCGGCTCCAACGGACTATAA

## >PflaOBP40

ATGGAAAATAAAATAATTTGTTGCGTACTATTTATTATTAGTTCATCATATAACGTTGTAGGAATGGATGATGAGATGGCAGAGTTAGCTCGCATGCTACGTGAGAATTGCGGTGAAGAAACTGGAGTCGATTTGTCACTTGTGGATAAAGTAAACGGCGGAGCTGACTTGATGCCCGACGCATCGCTGAAATGTTACATCAAATGCATTATGGAGACGGCGGGCATGCTGAGCGAAGGTGAAGTGGATGTGGAAGCAGTTATAGCTTTGATGCCCGACGCTATACGAGAGAAGAATGACGCCAATATCAGGAGCTGCGGAACTAAGAAGGGAGCAGACGATTGTGACACAGCATACCTCACACAGGTTTGCTGGCAGAAGGCCAACAAAGCTGACTACTTTTTAGTGTGA

## >PflaOBP41

ATGGACACCTTCAAGATGGGTGCGTCAGGGTTCGTTTTGTTGTTGACTGTAGACCTCCTGCTCGCCATGACAAGACAACAATTAAAAAATTCCGGGAAGATAATGAAGAAATCTTGTCTGGCTAAACACGACGTTACCGAAGATCAAGTTGGGGAAATAGAACAAGGAAAGTTCGTTGAAGACAAGAACGTGATGTGCTACATCGGTTGCATCTATACGATGACGCAAGTGATGACTCGGGCTCAGTTGAAGAAAACTTTAACGGTGGTGAAGAACCAGTGCATGGCGAAATACGGAGTGGGCGACAACAAAGTGGGTAACATCGAACAAGGACAATTTATAGAGGAGCACAGCGTGATGTGCTACGTCGCCTGCATCTACAAGAACATCCAGGTGGTGAAGGAGGACAAGTTGAATAAAGATATGGTGGTACGGCAGATAGACATCCTCTACCCGCAGGAGATGAGGGCCGGCGTCAAACGCTCCGTGGACCGGTGCATCCACGTTCAGGATAAATATGAAGAAATGTGTCAACGAGTTTTTTACGCCGTCAAATGTTTGTATGAAGACGACCCACCAAACTTCGTTTTCCCATGA

## >PflaOBP42

ATGAAGATTATGATATTGCTACTGGTAATACCCTCAATATTGCTAACAACGTTTCTAACGGAAATAGCAGAATCAAGGGCATTTCTGGATACTGATGGTTTTGCTAACTTACTATCTTCAATGGACCACATGAACTCGAGTACGGCCAACGCAGATCTTGTTACAGCAATGGGTGACTGCAATGAAACTTTTCGCATTGAAATGTCATACTTGGAGTCATTAAATAAAAGCGGCAGTTTTCCTGACGAAACTGATTCAACCCCAAAATGCTACCTACATTGCGTATTGAAATCACTGGAGATAGCATCTGGAGATGGAATATACAACCCTGAGAAGTTAACTGAACTATTCATGGAAAACAGGATAGGACCAAACATGGAGCAAAATGATATAGACGAGTTTGCTAACACATGCTCTCGGCGATTGGAAATGAACATATGCGAAAAATCCTACCAGTTTATGAAGTGCGTCCTCGAATCAGAGATAAAAATGAGTTCGATGAATAGAAATGAAACTTCGATGAGATCTGAGAGTACTATCAAGAAAACTTGA

## >PflaOBP43

ATGGATGATGAGATGGCAGAGTTAGCTCGCATGCTACGTGAGAATTGCGGTGAAGAAACTGGAGTCGATTTGTCACTTGTGGATAAAGTAAACGGCGGAGCTGACTTGATGCCCGACGCATCGCTGAAATGTTACATCAAATGCATTATGGAGACGGCGGGCATGCTGAGCGAAGGTGAAGTGGATGTGGAAGCAGTTATAGCTTTGATGCCCGACGCTATACGAGAGAAGAATGACGCCAATATCAGGAGCTGCGGAACTAAGAAGGGAGCAGACGATTGTGACACAGCATACCTCACACAGGTTTGCTGGCAGAAGGCCAACAAAGCTGACTACTTTTTAGTGTGA

## >PflaGOBP1

ATGGAGGGAAAATCTCGAAGGATATTAAGCGTTTTAATGATGGTTAGTCTATTTTCAGATATAAGAACTGATATGGTCGTGATGAAAGATGTTACTCTTGGATTTGGAGAGGCTTTGAAAGTTTGCAGAGAGCAGAGCCAGTTATCTGAAGAGCAGATGGAGGAGTTCTTCCACTTCTGGCGTGATGATTTCAAATTCGAACATCGCGAGATCGGTTGTGCGATCCAATGCATGAGCCGCCACTTTGACCTACTCACTGACACGCATCGTATGCACCACGAAAATACTGATAAATTCATCAAATCATTCCCAAATGGTGAGATTCTATCAAAGCAGATGATCGAATTGATTCACATGTGTGAGAAGAAATTCGACTTCGAAGAGGACCATTGTTGGCGGATACTCCACATCGCCGAATGCTTCAAGTTGTCGTGTCAAGAAACGGGTGTAGCACCGACAATGGAAATGCTTATGGCCGAGTTTATTATGGAATCGGAGCCATGA

## >PflaGOBP2

ATGGGTTCGATTTTGTTCTTATTAGTCACAATTGCAATTTTCATCGCCATTCCTGAAAATGTTGAAGGAACTGCTGAGGTAATGAGTCATGTAGCTGCTCATTTCGGGAAAACTCTGGAGGAATGTCGCGAAGAGTCCGGTTTGACCAATGAAGTGATGGAAGAGTTCAAACATTTCTGGAGCGATGATTTTGAAATAGTTCATCGTGAGTTGGGATGTGCGATCATCTGTATGTCTAAAAAGTTTTCTTTACTCAAAGACGACACCAGGATACACCATATCAACATGCACGATTATGTTAAACAATTTCCAAACGGTGAATTGCTGTCGGCAAAAATGGTCGAACTAATACACAACTGTGAGAAACAATTCGATGATATGAAAGATGATTGCGATCGTGTTGTTAAGGTGGCTGCGTGTTTCAGGGAAGATTGTAAGAAATCTGGTATAGCACCTGAAGTTGCCATGATAGAGGCAGTTATGGAGCAATATTAA

## >PflaGOBP3

ATGGAGGGAAAATCTCGAAGGATATTAAGCGTTTTAATGATGGTTAGTCTATTTTCAGATATAAGAACTGATATGGTCGTGATGAAAGATGTTACTCTTGGATTTGGAGAGGCTTTGAAAGTTTGCAGAGAGCAGAGCCAGTTATCTGAAGAGCAGATGGAGGAGTTCTTCCACTTCTGGCGTGATGATTTCAAATTCGAACATCGCGAGATCGGTTGTGCGATCCAATGCATGAGCCGCCACTTTGACCTACTCACTGACACGCATCGTATGCACCACGAAAATACTGATAAATTCATCAAATCATTCCCAAATGGTTAG

## >PflaGOBP5

ATGGAGGGAAAATCTCGAAGGATATTAAGCGTTTTAATGATGGTTAGTCTATTTTCAGATATAAGAACTGATATGGTCGTGATGAAAGATGTTACTCTTGGATTTGGAGAGGCTTTGAAAGTTTGCAGAGAGCAGAGCCAGTTATCTGAAGAGCAGATGGAGGAGTTCTTCCACTTCTGGCGTGACTCATTTCACGCATTCGTAACATCGCGAAATACTGATAAATTCGATCCAATGCATGAGCCGCCAATGGTGACCTACTCACTGACAAGCATCGTATGCACCACGAAAATACTGATTCACATGTGTGAGAAGAAATTCGACTTCGAAGAGGACCATTGTTGGCGGATACTCCACATCGCCGAATGCTTCAAGTTGTCGTGTCAAGAAACGGGTGTAGCACCGACAATGGAAATGCTTATGGCCGAGTTTATTATGGAATCGGAGCCATGA

## >PflaGOBP6

ATGGAGGAGTTCTTCCACTTCTGGCGTGATGATTTCAAATTCGAACATCGCGAGATCGGTTGTGCGATCCAATGCATGAGCCGCCACTTTGACCTACTCACTGACACGCATCGTATGCACCACGAAAATACTGATAAATTCATCAAATCATTCCCAAATGGTGAGATTCTATCAAAGCAGATGATCGAATTGATTCACATGTGTGAGAAGAAATTCGACTTCGAAGAGGACCATTGTTGGCGGATACTCCACATCGCCGAATGCTTCAAGTTGTCGTGTCAAGAAACGGGTGTAGCACCGACAATGGAAATGCTTATGGCCGAGTTTATTATGGAATCGGAGCCATGA

## >PflaGOBP7

ATGGAGGGAAAATCTCGAAGGATATTAAGCGTTTTAATGATGGTTAGTCTATTTTCAGATATAAGAACTGATATGGTCGTGATGAAAGATGTTACTCTTGGATTTGGAGAGGCTTTGAAAGTTTGCAGAGAGCAGAGCCAGTTATCTGAAGAGCAGATGGAGGAGTTCTTCCACTTCTGGCGTGATGATTTCAAATTCGAACATCGCGAGATCGGTTGTGCGATCCAATGCATGAGCCGCCACTTTGACCTACTCACTGACACGCATCGTATGCACCACGAAAATACTGATAAATTCATCAAATCATTCCCAAATGCTTATGGCCGAGTTTATTATGGAATCGGAGCCATGATCAACATTTGGAAATGA

## >PflaGOBP8

ATGGAGGGAAAATCTCGAAGGATATTAAGCGTTTTAATGATGAGCCAGTTATCTGAAGAGCAGATGGAGGAGTTCTTCCACTTCTGGCGTGATGATTTCAAATTCGAACATCGCGAGATCGGTTGTGCGATCCAATGCATGAGCCGCCACTTTGACCTACTCACTGACACGCATCGTATGCACCACGAAAATACTGATAAATTCATCAAATCATTCCCAAATGGTGAGATTCTATCAAAGCAGATGATCGAATTGATTCACATGTGTGAGAAGAAATTCGACTTCGAAGAGGACCATTGTTGGCGGATACTCCACATCGCCGAATGCTTCAAGTTGTCGTGTCAAGAAACGGGTGTAGCACCGACAATGGAAATGCTTATGGCCGAGTTTATTATGGAATCGGAGCCATGA

## >PflaGOBP10

ATGGGTTCGATTTTGTTCTTATTAGTCACAATTGCAATTTTCATCGCCATTCCTGAAAATGTTGAAGGAACTGCTGAGGTAATGAGTCATGTAGCTGCTCATTTCGGGAAAACTCTGGAGGAATGTCGCGAAGAGTCCGGCTTGACCAATGAAGTGATGGAAGAGTTCAAACATTTCTGGAGCGACGATTTTGAAATAGTTCATCGTGAGTTGGGATGTGCGATCATCTGTATGTCTAAAAAGTTCTCTTTACTCAAAGACGACACCAGGATACACCATGTCAACATGCATGACTATGTTAAACAATTTCCAAACGGTTAG

## >PflaGOBP11

ATGGGTTCGATTTTGTTCTTATTAGTCACAATTGCAATTTTCATCGCCATTCCTGAAAATGTTGAAGGAACTGCTGAGGTAATGAGTCATGTAGCTGCTCATTTCGGGAAAACTCTGGAGGAATGTCGCGAAGAGTCCGGTTTGACCAATGAAGTGATGGAAGAGTTCAAACATTTCTGGAGCGATGATTTTGAAATAGTTCATCGTGAGTTGGGATGTGCGATCATCTGTATGTCTAAAAAGTTTTCTTTACTCAAAGACGACACCAGGATACACCATATCAACATGCACGATTATGTTAAACAATTTCCAAACGGTTAG

## >PflaGOBP12

ATGGAGGGAAAATCTCGAAGGATATTAAGCGTTTTAATGATGGTTAGTCTATTTTCAGATATAAGAACTGATATGGTCGTGATGAAAGATGTTACTCTTGGATTTGGAGAGGCTTTGAAAGTTTGCAGAGAGCAGAGCCAGTTATCTGAAGAGCAGATGGAGGAGTTCTTCCACTTCTGGCGTGATGATTTCAAATTCGAACATCGCGAGATCGGTTGTGCGATCCAATGCATGAGCCGCCACTTTGACCTACTCACTGACACGCATCGTATGCACCACGAAAATACTGATAAATTCATCAAATCATTCCCAAATGATATAAGAACTGATATGGTCGTGATGAAAGATGTTACTCTTGGATTTGGAGAGGCTTTGAAAGTTTGCAGAGAGCAGAGCCAGTTATCTGAAGAGCAGATGGAGGAGTTCTTCCACTTCTGGCGTGATGATTTCAAATTCGAACATCGCGAGATCGGTTGTGCGATCCAATGCATGAGCCGCCACTTTGACCTACTCACTGACACGCATCGTATGCACCACGAAAATACTGATAAATTCATCAAATCATTCCCAAATGGTGAGATTCTATCAAAGCAGATGATCGAATTGATTCACATGTGTGAGAAGAAATTCGACTTCGAAGAGGACCATTGTTGGCGGATACTCCACATCGCCGAATGCTTCAAGTTGTCGTGTCAAGAAACGGGTGTAGCACCGACAATGGAAATGCTTATGGCCGAGTTTATTATGGAATCGGAGCCATGA

## >PflaGOBP14

ATGGAGGGAAAATCTCGAAGGATATTAAGCGTTTTAATGATGGTTAGTCTATTTTCAGATATAAGAACTGATATGGTCGTGATGAAAGATGTTACTCTTGGATTTGGAGAGGCTTTGAAAGTTTGCAGAGAGCAGAGCCAGTTATCTGAAGAGCAGATGGAGGAGTTCTTCCACTTCTGGCGTGATGATTTCAAATTCGAACATCGCGAGATCGGTTGTGCGATCCAATGCATGAGCCGCCACTTTGACCTACTCACTGACACGCATCGTATGCACCACGAAAATACTGATAAATTCATCAAATCATTCCCAAATGGTGAGATTCTATCAAAGCAGATGATCGAATTGATTCACATGTGTGAGAAGAAATTCGACTTCGAAGAGGACCATTGTTGGCGGATACTCCACATCGCCGAATGCTTATGGCCGAGTTTATTATGGAATCGGAGCCATGATCAACATTTGGAAATGACAATTTTACTAGCCCTACATAAATGTGCTTAG

## >PflaGOBP15

ATGGAAGAGTTCAAACATTTCTGGAGCGATGATTTTGAAATAGTTCATCGTGAGTTGGGATGTGCGATCATCTGTATGTCTAAAAAGTTTTCTTTACTCAAAGACGACACCAGGATACACCATATCAACATGCACGATTATGTTAAACAATTTCCAAACGGTGAATTGCTGTCGGCAAAAATGGTCGAACTAATACACAACTGTGAGAAACAATTCGATGATATGAAAGATGATTGCGATCGTGTTGTTAAGGTGGCTGCGTGTTTCAGGGAAGATTGTAAGAAATCTGGTATAGCACCTGAAGTTGCCATGATAGAGGCAGTTATGGAGCAATATTAA

## >PflaGOBP20

ATGGAGGGAAAATCTCGAAGGATATTAAGCGTTTTTATGATGGTTAGTCTATTTTCAGATATAAGAACTGATATGGTCGTGATGAAAGATGTTACTCTTGGGTTTGGAGAGGCTTTGAAAGTTTGCAGAGAGCAGAGCCAGTTATCTGAAGAGCAGATGGTGGAGTTCTTCCACTTCTGGCGTGATGATTTCAAATTCGAACATCGCGAGATCGGTTGTGCGATCCAATGCATGAGCCGCCACTTTGACCTACTCACTGACACGCATCGTATGCACCACGAAAATACTGATAAATTCATCAAATCATTCCCAAATGGTGAGATTCTATCAAAGCAGATGATCGAATTGATTCACATGTGTGAGAAGAAATTCGACTTCGAAGAGGACCATTGTTGGCGGATACTCCACATCGCCCAATGCTTCAAGTTGTCGTGTCAAGAAAGGGGTGTAGCACCGACAATGGAAATGCTTATGGCCGAGTTTATTATGGAATCGGAGCCATGA

## >PflaPBP1

ATGCGAAAGTTTTTAATATTGGTAGGCATTGTAGTACTTCATGTCGATAATAACAGGGTTCAGGGATCACAAGATATCATGAAAGATTTGACAATACAATTTGGAAAAGCGCTGTCAACTTGTAAGAAGGAATTGGATCTTCCAGACACAATCATGGCAGATTTTAAGAATTTCTGGAACGATGGCTACGAACTCTCCAATCGGTTCACGGGTTGCGCTATAATGTGTCTGTCTTCCAAACTCGACTTACTCGATCCTGAGGGCAAACTTCACCACGGGAATGCCCAGGAATTCGCAATGAAACATGGTGCAGACGCAACTATGGCTAAACAATTAACGGATCTCATACACAATTGTGAGAAGTCGATTACTCCGACTGAAGATGATTGTATAAACGTTCTCGAAGTGGCCAAATGTTTCAAAGCTGAAATCCACAAATTGAATTGGGCGCCAAACATGGATCTTATTGTTGGCGAACTGCTTGCTGAAGCTTAA

## >PflaPBP2

ATGGCGTTAAAAGTTGTTACGGGTATTTTAATAATGGTCGTTTATTTACACGAAACTCAGTCGTCTTCGAAAACTATGAAGATGCTTACAACTGGTTTTATGAAAGTGTTGGACACTTGCAAAGAAGAGCTTAATATATCAGAAGGCGTATTAAGCGACTTATATCACTATTGGAAAGAGGATTATGATCTTATGAAGAGGGATACTGGGTGTGTTATTGTGTGTATGAGCAGGAAACTGGATCTTTTAGATGACAGTGGTAAAATTCATCATGGAAATACAGCTGACTTCGCCAAGAAACATGGAGCTGCCGAGGAAACCGCTTCACAAATAGTATCATTACTACATGAGTGTGAAAAAACCCAGGACCATGTTGAGGATCCCTGTATAAAAGCCTTAGAAGTGGCTAAGTGCTTCAGGATTGGCATTCACAAGCTCAATTGGACTCCAAAGATGGATGTCTTAGTAACAGAAGTCTTGACTGATATATAA

## >PflaCSP1

ATGCGTGCAGTGGTTTTAGTCTGTGTTTTATATTGCTCTATGGTTTTTGTTTATGGGCAAGACACAAACGATGTGAAAAATTTACCAAAATACGATAATAGATATGATTTTTTGGATATAGATGAAATAATGGACAATAAAAGGTTGGTGAGGAACTACGTGGACTGTCTATTAAATGTGAAGCCGTGTACTCCAGAAGGCAAAACATTGAAACGATTGCTACCAGAAGCACTTCGTACAAAATGCGTGAGATGTACGGAACGACAAAAGAAAAGTGCTGTGAAAATTATTAAGCGGCTCAAGACCGATTATCCAGAGGAGTGGGCCAAGCTCTCCAGCCGTTGGGACCCGACCGGAGACTTCACTAGATACTTCGAAGACTTTCTTGCGAAAGAATCTTTCAACACCATCTCTGGTAGCGGTGAGTACATTAATATAGCTATTAGAAACGATAGCAGATATCTACGCAACTTCATCAAATGCTGCAAATCTTTGAAAAATCAAAGACAACTCTTAATTATAAGATTACTATCTTATTGTAGCTGA

## >PflaCSP2

ATGAAGGTCTTGATCATAGTATGCTTGGTGTCTGTCATCGCGTTGACATCAGCACGCCCGGAGCAATACACCAGTAAATATGACAACATGAACATTGACGAGATCCTGCAGAACCGTCGCATGTTGATTGGTTACATCAAGTGCGCTCTTGGCTTGGGACCTTGCACTCAAGACGGCAAGGAATTAAAAGAGCACATAGCCGATGCTTTAGAGACTGAATGCTCGAAGTGTACAGAGCCGCAAAAAAGTGGAGTGAAGAAAGTAATAAGTCACTTGATCAACAACGAAAAGGATTACTGGAAACAGCTCTGCGATAAATACGACAAGAATCGCAAATACGTCACTAAATACGAAGCTGAACTCAAAAAAGTTGCTTAA

## >PflaCSP3

ATGATGTACATGAAGTGTGTGATGGTGCTATGCGTGGTGGTCGCAGCTACTGTCGCTGACGATGACAAGTACACCGACAAGTACGACAACATCGACATTCAGGAGGTGCTGGAGAACAAACGGCTTCTGCTGGCATACATCAACTGCATCCTAGATAAGGGTAAATGCAGTCCCGAAGGCAAAGAACTTAAAGAACACTTAGTAGACGCATTGGAGACTGGCTGTAAGAAATGTACTGAAAAGCAGGAGAAAGGTTCTACCACTGTCATCGACCATCTGATCAAGAACGAAATAGAACTGTGGCGTGAGATCTGTGCCAAGTACGACCCAACCGGGGAGTACAGGAAGAAGTATGAGGAGCAGGCCAAGTCCAGGGGCATCGAAATCCCTCCCGAGAAGTAA

## >PflaCSP4

ATGAGGGTAATAATTTGGATCAGTCTGCTGTGCATCATATCAGCTGTGAGCGCTAGACCCGGCCTAACATACACCGACAAATTCGACCACATTAACGTCGACGAAATACTGGAGTCAGACAAAATATTAAGGGCATACGTGAAGTGTCTTATGGACAAGGGTCGGTGCACGCCCGACGGGAAGTCGTTGAAGGAAACTCTACCTGACGCCTTAGAGAATGAATGCTCCAAGTGTACGCAGCTGCAGAAAGATAAGTCGACAAGAGTCGTCAAATTCTTGATCAACAAACGACCTGACTTGTGGAACGAACTTTCCACCAAATATGACCCTGATAATGTTTATCAGCAAAAATATAAGGACAAGATCGATAGCATCAAAGCCGAAAGTTAA

## >PflaCSP5

ATGCCTACATCATGGTGTCTCGCTCTTTGCGCCGCTCTATCGGTGGTCGCGGCTGAATTCTACAACCCGAAGTATGATAACTTCGACATACAACCACTTTTGGAAAATGACAAAATCATGACGAACTACATTAAATGTTTCCTTGATCAAGGACCTTGCACGACTGAAGCTAAGGATATCAAAAAAGTTATTCCGGAAGCATTAGAGACGACATGTGGCAGATGTTCTCCAAAACAAAAGGAACTGACCAGAAAAGTAATAAAAGCAATTATGGACAAGCAGCCGGACTCGTGGAAAATGCTGGTCAGCAAGTACGATAAGGACAAGAAGCACGTGGAGACATTCAAAAAGTTTTTTGAAGGGCATTAG

## >PflaCSP6

ATGAAGGTCTTGATCATAGTATGCTTGGTGTCTGTCATCGCGTTGACATCAGCACGCCCAGAGCAATACACCAGTAAATATGACAACATGAACATTGACGAGATCCTGCAGAACCGTCGCATGTTGATTGGTTACATCAAGTGCGCTCTGGGCTTGGGACCTTGCACTCAAGACGGCAAGGAATTAAAAGAGCACATAGCCGATGCTTTAGAGACTGAATGCTCGAAGTGTACAGAGCCGCAAAAAAGTGGAGTGAAGAAAGTAATAAGTCACTTGATCAACAACGAAAAGGATTACTGGAATCAGCTCTGCGATAAATACGACAAGAATCGCAAATACGTCACTAAATACGAAGCTGAACTCAAAAAAGTTGCTTAA

## >PflaCSP7

ATGACTACATCATGGTGTCTCGCTCTTTGTGCCGTTCTATCGGTGGTCGGGGCTGATTTCTACAGCCCGAAGTATGACAACTTCGACATACAACCGCTTTTGGAAAATGAAAAAATTTTGATTAATTACATTAAATGTTTCTTGGATCAAGGACCTTGTACGACTGAAGCTAAGGATTTTAAAAATGTGATCCCGGAGGCGCTGGAGACGTCGTGTGGCAAATGTTCACCAAAACAAAAGGAACTGATCAGAAAAGTGATAAAAGCAGTTATGGAGAAGCAGCCGGACTCGTGGAAGATGCTGGTCAGCAAGTACGATAAGGACAAGAAGTACGAAGAGACTTTCAAGAAGTTCATTGAAGGGCACTAG

## >PflaCSP8

ATGCCTACATCATGGTGTCTCGCTCTTTGCGCCGCTCTATCGGTGGTCGCGGCTGAATTCTACAACCCGAAGTATGATAACTTCGACATACAACCACTTTTGGAAAATGACAAAATCATGACGAACTACATTAAATGTTTCCTTGATCAAGGACCTTGCACGACTGAAGCTAAGGATATCAAAAAAGTTATTCCGGAAGCATTAGAGACGACATGTGGCAGATGTTCTCCAAAACAAAAGGAACTGACCAGAAAAGTAATAAAAGCAATTATGGACAAGCAGCCGGACTCGTGGAAGACGCTGGTCAGCAAGTACGATAAGGACAAGAAGCACGTGGAGACATTCAAAAAGTTTTTTGAAGGGCATTAG

## >PflaCSP9

ATGAAGACCATTATGATACTTTGCTGTGTCGTAGCTGCATTTTGTGAAGACGAATTCTATAAGACAAGTGAAGGGTTCGATATATCTGAAGTACTTAAGAACGATCGCCTTTTAAAGGCGTACACGCAATGCCTTCTCGACCAAGTTCCATGTACTCCAGATGTCAAAGAGCTTAAAGATAAAATACCTGAAGCACTGGAAACAAATTGCGCAAAATGCACTGAGAAACAAGAAGAATTGGGCAGACAACTCATCCAACATATCAAAAATACGCATCCTCAACTATGGGAGCAGCTGAAATCTAAGTACGACCCAGAGGGCAAATATGTGACAGCTTTCCAGGAATTCCTGAATGAATAA

## >PflaCSP10

ATGAAGTGGTATTTCTTTGTATGTTTTTATCTGCTGGTTCAGGCGGTAATATCCTATGACCCTCTGGTAGATACAGACGTGTCGATTTTAGAAGACAAAGCGATGATGAAGGTTATATATGACTGCTTACTCGACAAAGAACCTTGTGGAGAGTTTCAACCACTGAAAGACAGAGTGTTAGAAGTGTTGGCAGATAATTGTGCAAAATGCCCTGGACCACTAAAAGAGAAGTTCGCAACCGTCGTTAAAATCGCTAAGGAGAAATATCCTGAAGAAACGACAGCCGTTTTAAAAAAATACAGCAAAAATTAA

## >PflaCSP11

ATGCAGAGTGCCATCCTCCTTGCCCTGGTCTCCGTGGTGGCATACTCAGCTGCTGAGTCCTACACCGATAAGTTCGACGGCATCAACGCTAAAGAGATCGTTGACAACCACCGTCTCCTCGAGGGTTACGTCAAATGTGCCCTAGACAAGGGGCCTTGCACCACCGAGGGATTAGAACTAAGATCACACATTGAAGAGGCCCTATCGAATAAGTGCGCCAAATGTACCGAGAAGCAGAAGGAACTGACGCGCTTCGTTATCGGCCATTTGATCAATCACGAGCCAGAGTTTTGGCATATGCTTTGCGAAAAGTTTGACCCCAAAGCCAAATATGTCAAGGAATACGAAAAGTTTGACCC

## >PflaCSP12

ATGAAGTCTCTGATATTGTGTGCTGTGGTCGCCACGCTTGCATCAGCCCGGTACACCAGCTACCCGACCACACATGATGACTACGATATCGATCATGTAGTACTTAAGCCGGAAACTTTTAAGGACTTCCTATACTGCGTAATCGACCAAGGACCCTGCGATGAGGCCCATGCGTTTTTCAAAAAGTATACACCAGATGTACTCGAGACTGCATGTAAATATTGCAACGAACCGCAGAAAAATATCCTGGTGAAATTCCTGGCCGCAGCACCTAAGCACTTCCCCAAAGAATACGAAACTTTCAAGAAAATATATGATCCTAAAAACGAGTACATACCGAATGCGGCGAAGGTCCTTGCTGGGGCTTAA

## >PflaCSP13

ATGATATTGATGTTATTAACTTTCCTGTTGCTGTTACTCACATCCGGAACCTTAACTGAGGAGGACCATACATATACGAACAAATATGACAATGTGGACATCGATGATATTCTTGACAATGAGCGCCTTCTTAATGGTTATGTAAATTGTTTGCTAGATAAAGGCCCTTGTACGCCAGACGGCAAAGAACTAAAAGATAACATTCCTGATGCTATCAAAAATGATTGCACCAAATGCACGGAGAAACAACGCGACGGCGCAGACAAAGTGATGCATTACATCATCGATCATAGACCCATAGACTGGAACAAGTTAGAGGAAAAATACCATTCTGATGGAACATACAAAAAAAAATATTTGGAGACTAAAAACTTAAACACCAATAGTACGAAGATAAATACACATAACTAA

## >PflaCSP14

ATGAAGATCGGCATTGTTTTCACGTGTCTCGTGTACACCGTGTTCGCCGATACGTTGAGCAAAAAATACGAGAATTTTGATATCGAGCCTATCATCAACAACGATAGGCTGTTGAAGGCGTACATCAATTGTTTCTTGGACAGGGGTCGTTGCACTTCCGAGGGGACAGACTTCAAAAAGGCACTGCCTGAAGCTGTAGAGACTGTTTGCGCAAAATGTACGGACAGCCAGAAAGACAAATTCAAGAGGGTGGGCAAAGCGACAAAAGCCAAGTTCCCTACACTATGGAAAGAGGTCGTGGCCAAACACGACCCCACGGGCAAGTACGGAGACGCATTCGTTAAATTCATCGAAAATTAA

## >PflaCSP15

ATGCCTACATCATGGTGTCTCGCTCTTTGCGCCGCTCTATCGGTGGTCGCGGCTGAATTCTACAACCCGAAGTATGATAACTTCGACATACAACCACTTTTGGAAAATGACAAAATCATGACGAACTACATTAAATGTTTCCTTGATCAAGGACCTTGCACGACTGAAGCTAAGGATATCAAAAAAGTTATTCCGGAAGCATTAGAGACGACATGTGGCAGATGTTCTCCAAAACAAAAGGAACTGACCAGAAAAGTAATAAAAGCAATAATGGACAAGCAGCCGGACTCGTGGAAAATGCTGGTCGGCAAGTACGATAAGGACAAGAAGCACGTAGGGACATTCAAAAAGTTTTTTGAAGGGCATTAG

## >PflaCSP16

ATGAAGTGGTATTTCTTTGTATCTTTTTATCTGCTGGTCCAGACGGCAACATCCTATGACCCTCTGGTAGACACAGATGTGTCGATATTAGATGATAAAGCAACGATGAAGGTTTTATACGACTGTTTACTTGATAAAGAACCTTGTGGAGAGTTTCGACCACTGAAAGACAGAGTGTTAGAAGTGTTGGCAGATAATTGCGCAAAATGCCCGGGACCACTAAAAGAGAAGTTCGCAACCGTCGTTGAAATTGCTAAGGAGAAATATCCTGAAGAAACGACAGCCGTTTTAAAAAAATACAGCAAAACCTAA

## >PflaCSP17

ATGCAGACCGCTATTGTGATACTCTGCGGTGTGGTCGTGGCGTGCGCTGCCCAGAATCAGGCCGACAACGCGAGACCACCAGTGTCAGATACTGCTCTGGAGGATGCACTCAATGACAAGCGGTTCATTCAGAGACAACTCAAATGTGCTTTAGGAGATGCTCCTTGTGATACGATTGGCAGAAGATTAAAAACTCTAGCTCCCCTGGTTCTCCGCGGAGCCTGTCCTCAATGTTCTCCTCAAGAGACCAAGCAAATTCAGCGTACGCTCTCGTACGTACAACGAAACTATCCGCAGGAATGGGCTAAGATCGTCCGTCAATATGCCGGATGA

## >PflaCSP18

ATGAGGGCGATTGTATGTCTCGCAGTGCTGCTTGTGGCTACGGCTGCCGTGTCTGGTGAATACTACCAGAGCAGATATGAAAATTTTGATCTGACGACCCTGGACAATGTGAAATTACTCATGGGATACGGAAATTGTTTCATCTTCGAAGGCCCGTGCACAAGTGAAGGGAAAAGTTTCAGAGACTTTATACCTGATGCTGTTCAAAGTCTTTGCTCCAAATGTAGCCCAAAGCAAAAGGAGATTATAAGAGCGTTCATAAAGGCATTGCGCGCTAAGCTGCCTGAAATATGGGAGAAACTGTTACAGAGATACGACAGCGAGAAGAAATACATCGACAAGTTGGAACAGTTCCTTAACGAATAG

## >PflaCSP19

ATGAAGGGTTTCTACGTGCTGTGTTTTGCACTGTTCGCTGCTGTCTACTGTAAAGAGACATACAGCTCGGAAAATGACGATCTGGATATTGAAGCTTTGGTGGGAAATATTGATTCTCTGAAGGCCTTTATTGGATGCTTCTTAGAAACATCCCCTTGCGACGCTGTTTCCGGGGATTTCAAAAAGGACATTCCTGAAGCTGTGGCAGAAGCATGTGGCAAATGTACTCCAGCCCAGAAACATCTATTCAAACGTTTCCTTGAAGTCGTCAAGGACAAGCTACCTCAAGAATACGAAGCCTTCAAAACTAAATACGATCCCCAAGGAAAGCATTTCGATGCTCTGTTATCCGCCGTCGCTAATTCTTAA

## >PflaOrco

ATGATGACCAAATCCAAACCCCAGGGCCTCGTGTCCGATCTGATGCCCAACATCAGGTTAATGCAGATGGCTGGGCACTTTTTATTTAACTATCATTCTGAAAATGCTGGCATGTCCACACTCTTGCGCAAAGTCTACTCGAGTATTCACGCATTCCTCATCACGATCCAATTTTTGTGCATGGGCATCAACATGGCGCAGTATTCTGACGAAGTCAACGAATTAACTGCTAACACCATTACAGTCCTCTTCTTCGCACACACCATGATTAAGCTTTTATTCTTTGCCCTCAACTCAAAAAGTTTCTACAGAACTCTTGCTGTGTGGAATCAATCCAACAGCCACCCTCTCTTCACCGAATCTGACGCCCGCTATCATCAACTGTCGCTGAACAAAATGAGGAGACTCCTGTATTTCATCTGTGCCGTCACCGTATTATCAGTTATAAGTTGGGTGACCATAACGTTCTTTGGAGAATCAGTTCGTTTGATTGCTGACAAAGAAACTAATGAGACACTTACTGAGCCGGCTCCTAGGCTGCCTTTGAAAGCTTGGTATCCTTTCGATGCAATGAGCGGCGCCATGTATATTTTTGCGTTCGCTTTTCAGGTATACTGGCTGCTCTTCTCAATGGCGATTGCCAATCTATTGGACGTGATGTTCTGCTCCTGGTTGATATTCGCCTGTGAACAGCTGCAGCATTTGAAAGCTATAATGAAACCGCTCATGGAACTCAGCGCTTCCCTTGACACATACAGACCCAACACTGCTGAGTTATTCCGTGCCTCTTCAACAGAGAAGGCAGAGAAGTTTCCAGAAACCACGGATCTTGATATCCGTGGTATATATTCGACCCAACAGGACTTTGGTATAACACTCCGTGGTGCTGGTGGCAGGTTACAGACTTTTGGACAACCGGCGCCCAACAATCCCAACGGCTTGACCCAGAAACAGGAAATGTTGGCAAGATCAGCCATAAAGTATTGGGTGGAGAGACATAAGCACGTAGTACGATTGGTAGCTTCTATCGGGGATACATACGGAACAGCGCTTTTATTCCACATGTTGGTTTCAACTATCACACTTACTCTTCTGGCTTACCAAGCAACAAAGATCGATGGCCTAAACGTATACGCATTTAGTACAGTTGGTTATCTCAGCTATACTTTGGGTCAGGTGTTTCACTTCTGTATCTTTGGTAACAGGCTCATTGAAGAGAGCTCTTCGGTGATGGAGGCAGCATACTCTTGCCAATGGTACGACGGATCTGAGGAGGCTAAGACTTTCGTTCAGATAGTATGCCAGCAATGTCAAAAAGCGATGAGCATATCAGGAGCCAAATTCTTCACCGTTTCTCTCGACCTGTTTGCTTCGGTGCTGGGTGCTGTGGTGACCTATTTCATGGTGTTGGTGCAACTGAAGTAG

## >PflaOR3

ATGAGCAGTTATTCAATAATGTGCGTTTGTGCTGTGTCGGTCGGTATCATAATGCCTCTGACGGAACAATTCCAAGTTCTGCCGACGAACGTGGAGTATGCGAGCATTGATGTTTACTCCTCGCCGACATATGGCATCCTTTACTTGCACCACATTTACTACAAACCTGCTACGTGCATCATAGATGCTGCCATGGACACGATATTAGCTGCTTTTGTTGCTTCTGCTATTGGTCAGATCGAGATATTAGCCTACAATTTGCAACATTTCGACTTGATAGCGAAACGGAAACATACAAGAGCTATTGTGAAGAAAGAAGCTGTGCATTCGATGCCTTATTATATTCGTTCGACCATCAAAGAATGTATAATACTGCATAAATGCATAATACGCTACGTGTCATTGATTGAGAGCGCGTTTAGTTTAGCGTCGGCTCTGCAGTTCATGCTGAGTATTATGGTGCTGTGCTTGATTGGTATCCAGTTCCTGTCGATTGAAAATCCTGCGAGTCACCCTATGCAAATTGTTTGGATGGCCATATATTTGTCTTGCATGTTGGTTGAAGTCTTTATCCTTTGCTGGTTCGGGAATGAGCTTATTTGGAAGAGTTTGGATCTGCGTCAGGCTGCATTCAATGGTCCTTGGCAAGAAGTCGATGTGCCAACTAGGAGATTCATTATCATATTCTTAGAACGATGCCAGCGTCCTTTGAGAGTATCTGCAGGGAAAATATTCACTTTGTCTCTTGACACATACACTGTCCTCATCAATTGGTCCTACAAGACATTCGCCTTAATGAGTAACGTTAAAAACTAA

## >PflaOR4

ATGCCGATAACAGAAACATTTAAAAATACTTTCTACAAAAGTGATTACGATTTACATGGCCGTAACATAAAATTGTATACATATCATCCACAAATACAATTTGGATTCGCTATCAATGGAATTTTTTTCAACGTAACAAACTCTAAAATACGGTTAATTTGGCCTTCTTTTAGCGTATTGATCTGCCTGGTGGCAGCGGGCTTAGAATTGATGTTCATTTATCACGGCATTGAAATAGAAGACTACGCATTTGCTACAGAGAGCTTTTGTTACTGCTTGATGTTGGGCCTTGTGCCGCTATATTACTTGTGCGTTCACAAAAATAAACCAAAAATCCTAAAACTTCTTCAAGACATGAATGATGATTTCGATTACATCTGTTCTTTTGGGCCACAATTTAAAGAGAAGTTCCTGGAGGGTCAGTTACTGATATGGAAACTGTTCTGTTCATGGATTACTTTCAGTATACTTTTGTTGATACTCTTTTGTTCGACTCTTGTATTGTCTGTAATGTATCAGAGCATATTCGCAGTACAGGATGAACATTACGTCAGACCTTTGATATTTCCTCTGTGGCTGCCAGAAGATGACCCTTACAGGACCCCAAACTACGAAGTGTTTTTGGTCCTCGAATTCGTTATGATGTTGGTCTATTATTTTGCATTTTTCGTGTACGTGTATATGTTGTTTCACTTTCTACTGCATTACTATAGTTTATTAAATATAGTAAAACTGGCGATCGAGAACGTATTCGACGATCTGGATCCGACGGTTGCAAAACTGTCAGTACGAGACGAACGTCGCGTGCATCATCAGGCGGTACTTAACTCGAGGATGAAGCAAATTGTAAAATGGCACATTTCCGTCATTGAAGCCATCGATACAATATCTGTTATTTATGGAATAGTCTTAGTTTATCAAGTTCTGTTAAGCTCCGTCGCTATATGCTTGATGATGTACCAGGTGGCCCGCAAATTAGATGAAGGGACACTCGATTTCATATTTGTAGCCATTTTCGTGGGAGGCACTTTGCAGTTATGGATACCCTGCTATTTGGGATCACTGATCAGGAACAAAGCATATGAAGTCGGTGACACTTGTTTCTACTGCGGCTGGGAAAAGTCAGAGCTGGGCAGGATGATACGCCAGGATCTCACCATAATAATCCTGAGGACACAACGACCCATTATAATCAGCTTTACAGCGTTACCAGCTTTGCAGCTAGAAACATTTTCATCGGTAATGAGTTCGGCGTATTCTTACTTCAATATGTTGCGTCAATCAACTTAA

## >PflaOR5

ATGGGAATCTGGAATGAAAAAGACAGATCAAGAGAAAAGTTAAAGCAAAGATTGATATGTCTATTCAATCTGTTGTGGTTAGATACTGACGCAATTGGAGCCCTATTTTGGACAATTGAAGCAGCACAACAAGGGAAAAGTCTATCTGAAGTGACTCGGGTGGCACCATGTACTACTCTTTGCATTGTCGCCAATTTGAAAGCCATATTTCATGTAATCAACGAAAATGCTTTAAAAAGTATTCTCGTAGGTATAAAGCAATTAGAACATGAAGAAAGTCAAAGGAATAATTCATTACCGTTGAACGCATCACGAAACAGAATGAAATCCTCTGAAGCGAAATTTTTAAAATCTGTAGTAAACGTAGTCATCTATTTAAATTTTATGACGATAACTGCCTTCGCTTTGACTCCTTTAATTTTGATGGCATTAAAATATTGGAAATATAATGAATTGGAACTAATGCTACCTTACATGGTTAATTATCCTTTTGGCATCAATAAGTATGATTTTAAACACTGGGTGTTTTTGTGCTTACATCAATATTGGTCAGGTTGTATTGTGGTCCTGCTGATGTTATCTTCCGACTGCTTCTACTACACCTGCTGCACTTTCATCAAGATACAGTTCGTTTTGCTTCAATTTGACTTCGAGGAGCTTTTTGATGATAGAAACGCCATGGAAAGCTATGCGACTACGCTAGAGGAGTTTAAGAAATTAATAAAAAGACATTTGATTTTAATAAAGTCAATAGAAAATATAAATAAAATTTACGGCGTCTCCACTCTAGGTAACTTCATGTCAAGCTCTGTTCTCATTTGTTTATGTGGCTTCAACGCTACGGAAGTGGAAGATGTTGTGCTCATGTTGACGTTTGTATGTTTTCTGGTCATGAGCCTGTTTCAAATATATCTACTCTGTTTTTTTGGTGACGTGTTAATAGCCTCGAGCCAAGGTGTTAGTAACGCAGTCTATAGCAGCAAGTGGTACAAGACACATCGTTCTATGGGGAAATTAATTATTATCATTCAAACCAGGGTACAAAAGGCATGCAATCTAACAGCTTTCGGCTTCGTTGACGTAAACCTCCAGGCATTTCAAAAGATACTAAGCACCGCTTGGTCATACTTCGCCTTGTTGAAGACTATGAATGCAGGGGGCGAAATAAGAAATTAG

## >PflaOR6

ATGGATCAACCTATGTCTTACAGGAGTATTCTTCCTCATTTAAAATGGCTTCTATATATGGGATTTTACCACTACAACGAGCCTGCCAGCGAAGGGAGAAGAAAGCTATACAAAATACACAGCAATCTTTCTATCTTCTTTGTTGTATTGTATACAATGCAACTTCTGATTCAAATGTATTGTTATCGAACGGATTCGGAAAAATTGTTTGATTCATTTCTCGTTTTTCCATCATGTACAGATTGTATTTACAAACTGTGTGTTCTCAAATTCAGGGGAGACAAAGTAAATGAACTTCTGCATATAATGAGAGGACCATTATTTAATCAACTCAACGCCAAAGAAATTTTGAAAAACGGTGAACACGAAGCTGGATTTATTCACCGAGTGATTATCAGATTTACGTTGCTTGCAACAATATTTTGGGGACTATACGTACCAGCATTGCTACTTACTGGAAACGAAGTAGATTTCACCCTGTGGTTTCCCTTCGATCCACAATCTACAAGCACTTTGACAGCTGTAGTCACTGTTTACGGTCTACTAACGGTAATTGTGGCGGGATGCACCAATAGTATTGCGGACAGTCTCACTGTTGGATTATTTATTCAATGCAAGACACAGTTCAAGGTTTTAAGACTAGAATTGGAGACTTTAGTGGAAAACGTTCAACAAAGTTGCAATAAAAAATCAAACCATTATCTCAATTCGCTAAACAGACATTTACGTCATACAGTTTTGCATCACTTAGAAATACTCAGATTCTTTAACCTGATTCAAGACTTATATGGTGATGTATTCGCGTGCCAATTCGTAGTAACTGCTCCAGTCATGTGTACATGCATTTACAGGTTTTTAACTATACAACCATCAGTTTTCCAATATGTGGCTATCTCAGGCTACTTTGGTTTCGTGTTGATCCAACTGTCCATTTATTGTTACTACGGGAATGAAGTCAGACTTCAGAGTGAAGAGCTTATAACGGCGGTTTATTTTATGAACTGGGTGGAGACTCCGGTAAAATTTCGTCGTTCGCTGACAGTAGTGATGGAGAGATGCAAAATACCAATAGAACCTACAGCTGGTGTTATCGTTCCTCTGTCTCTGTATACATTTATTAGAATACTGCGAATCTCTTACACCTTGTACTCAGCCTTAAAACAAACAATGGATTTATAA

## >PflaOR7

ATGTCTAATATGTATGGTAATAAAACTTTTCAAGGATTTCGACCGCATTTTGATGCATTGGCAAGAGTGGGATACTATAAAATCGTTATGAAACCAATATCGGATATAAAAAGATCGATGCATCTTATGTATCGATACATTGTTACGTTGTTTGTAGTAATTTACAACTTGCAACACTTTATACGTTTATTTCAGGTCCGGAATAATATTGAGCAGGTTGTGGATGTACTATTTGTATTACTTTGTACGCTGAACTGTCTGGCGAAGCATATCAGTTTCAATGTTAAGTCGATCCGCGTCGATGGACTCATTAAAATTATAAATGATCCCGTATTTACGGCGAAAAGTGAAATTCAACAGTCGATCATAAATAAAAATTCAAAAGCAATGTTGCAAGTGTTAAATATTTGTCATTTCTACATATACACATGTGCCGTTTTGTGGGCCACTTACCCCATGGTCAATAAATCTATGGGCATAGACGTGGAGTTCACTGGATATTTTCCATATGATACAAGTACTTCGCCAATATTTGAGATGTCTCTAGTGTACATGTCTATTTTAATTGCAATTCAAGCATATGGACACGTGACAATGGATTGTACGATCGTATCATTCTATGCTCAAGGGAAAACGCAACTGCGGCTTCTCAGATACAAACTGGAAAATCTGAGCGTTATAAGTAATGACACGGTGAAAATAGAATCCGCATTATATGAGATCAGAAATAAATCAACATATTATAAGGATTACGGGTACGAAGACATTATGATGTACGAAAAAATAACCAATTGCGTGAAACATTATCAGAAGATTGTGTGGTTCGTCAATGAAATTGATTCTATATTTCAACAAGTATATCTGATTCAATTTTTCGCAATGTCGTGGATAATATGCATGACTGTTTATAAGATGGTGGGCTTAAATTTTCTCTCCGTGGAGTTTGTGTCGATGTTCGTTTACTTACTATGCATGCTTGGACAGTTATTAAATTACTGCTACCACTGCACGCAATTGAAAGTGGAGAGCGAGTTAGTAAACGATTCAATTTATTGTGGAGATTGGCTGACTTTATCACCTAAACACCGTCGAATGCTTTTGGTGATGATGGTACGATGTCACCGCCCTCTTGTACCGCGCACTGCGCATATACCATTATCTTTGGAAACTTATCTTTCGGTAATAAAGTTTTCATACACTTTGTTCACTTTTCTGAAACGTAAATAA

## >PflaOR8

ATGTTCAGCCTCTCTTTTGTGGTCATTCACGATTTGACATTCGTAAAACTGTACATCTTCTTTGTTAAGAACAAAGATATCCAAGACGTTACTAGAACCCTTGAGATGGACTTACAGCAGTTCTATCAGAACGACCAAAAAATACGAGCCACTGTTAGAACTACGAAGATACTTACTGGAGCATTGGTTTTCTCCTGTGTATTTACTATCGCAAGTATTGATTTACAAGGAGCAGTTGAAGACCACAAATGGAAATCCAAAGTAAAGTCCTTAAATCACTCATCTCAAGTACCACCTCGAGCATTTCCTCAACCTATTTACATACCTTGGAGTTATGAAAGTGATTTATCTTACAGAATAACCTTCGCTTTAGAAACATTGGCCCTTCTTTGGACTGCTCATATAGTGATTGCAATGGATACACTAGTTTCCAGTTTGATACTACATATGTGTGCCCAATTTGAGATCCTACAGGAAGCCGTTACCACGGCATATGATCGTACTATTTCTTATTTATATCAAGGCGTAGAAGATTCCAATACAAATGAAATACATGTAGATAACAGTAAAAGACAAGATATAGTTAAATCTTGCTACCCGACTGAAGAAATAGAAGCTGCGTTAGAAAAGACGTTTAAGGATTGCATCGCTCAGCATCAGGTTTTGATTACCTGTACACAGAAGTTCGCAAGACTATATTCCTACGGATTTGCCGTTCAGCTTGTTTCCAGTATGGCTGCAATCTGCGTGGTCATGGTTCAACTTTCGCTAGACGCGTCAAGTCTCAACTCGCCGAGCCTGGTGTCATCACTCGCGTTCTTTACTGTGATGCTTATAGAATTGGCGATACAGTGTTTTACTGGAAACGAGTTAACCTTACAGGCGATGAATGTAGCTGACTCTGTGATGAACTGCAACTGGGAGATACTTCCGATACGTGTCCGTCGACTGTTGCTATTCGTCATCATGCGCGCCCAGCATCCAATCTACCTGACGGCTGCCGGCTTCGCCAACATGAACTACCAGTGCTTTTTGTCAATTCTGAAAGCAGCATACTCTTATTACGCTGTTCTAAGTCAGAGGCAGATGTAG

## >PflaOR9

ATGTCTTCAAAGGACACAAGGGAGCTTATAAGAAATGGATCAGCTCCATATCCCGAAATAATGAGTTCATGGTTTCCTTTTGATACAACAAAAGGTTACGGTTATTGGACGAGTATAATAATACACTCCTTAATCTGTTTCTATGGCGGGGGCGTTGTGGCAGCTTACGACTCTAACGCTGTAGTTTTAATGACGTTTTTTGCTGGGCAGTTGAAGTTACTATCGGCTAATGCCGCTAGAGTGTTCGGCGAAGGGAACGAGATTGTGAGTTATAAAGAAGCAAGAGAGAGGATTAAGAAATGTCATTATCATCACTTGGACTTGGTCAAAAACGCCATGATTCTCAAATCTTTGCTATCTCCAGTATTATTTGCATATGTCGTCATATGTTCTTTAATGATTTGTGCCAGTGCCATACAATTAACATCGGAAGGAATGCCAAAAATGCAGCAAATTTGGATTGCAGAGTACTTAGTAGCTCTAGTTGCGCAACTGTTTCTTTATTGTTGGCACAGTAATGAAGTATCTATAATGAGCGATAACGTCGTAAATGGAGTATACTTGAGCAAGTGGGTTAACCAAGACGTACGAATGAGACGCAATTTGATGATGCTAAGTGGTCAGCTGCGTAAGCGCATTGTTTTCACCGCAGGTCCTTTTACAACGCTTACCGTCAATACATTTGTTACGATACTGAAGGGGTCATATAGTTACTACGCAGTCTTGAATGAAAAAAATGATTAA

## >PflaOR10

ATGGAAACAAATAAAAAAATCAAACCCATCGATGCTTTTAAAATGCTACTTCGCACATTAAAATACACTGCATATTTCATGCCGGTGCCCGAGTTGGAAAATCCAACAAAAAACATGTTGCACGACATATACAGAGCTTTCAACATATTCGTGTTGTGCTTATACGATTTGCAGCATTTTATTTACGTCATACAGGTTTTTGGCGACATCGACAAAATGATAGACAGCCTGAGCGTGATGATCACAGCTGTGAATGTAACCTTCAAAGTGGTTACTGTCAATATCAACAACAAAAGGTTTAATCGTCTGCATGATATTTTAAATGATAAGATTTTTAGCGCAACCTGTCCAAAAGATGACGAATACATCAACGACAATAAAATAAGTTTAGAGAGACTATCGAAGACCGTCAACAGAACAATAATTGTGATTGCGATTTGTTGGTTTCTCACACCGATCCTGAAGAAACTCGGTGAAGATGAAGGCGCTCTACCGGCGTATTTCCCATTTGATACGGAAAATTGGGTTGGATTCCTGTGTGCTCACACTTATTTAACAGTTGTCATCATCTGGATAGGATACGGACATATGACCTTAAATTTCCTAATGGTTGGCTACTACAGTCAAGTTAAAGTACAGCTGAGAATCATAAGATACCATTTGGAACATTTAGCTGATGATGATGTTGGTGACGAGCAAATCAACGTTCCGACTGTCTGTCGTGATTTAGAATTCACGTACAAAGATCACAAAAGTATTAAATACCAGCAACGACTTATTGATCTAGTCAAAAGATACGAAATGGCGGTTTGGTTTTCAAATGAGATCGAATCGATTATGAACAAGGCTATGCTGGTACAGTTTGTTGGCTCCACTGGTATAATTTGCACAGTAGTTTTTAAAATGACTGGGATGCCTTTTAACACTGGCTTCATCTATGTGCTGCTTTATTTGGCGTGCTTGCTCCTGGAACTGTACATATATTGCTACTATGGTACTCTCTTGGAATATGAAAGTCGTTTTTTGAACGAATCTTTATATATGAGCAACTGGATTTCACTGTCTCCACGTTTCCGCAAACAGTTGCTCATCGGAATGGCACGCTGGGGGAGACCAATATCCCCAAAAGCTGCTGGCTTGGTCGCAATTTCGCTGAACACTTTCATTGTGGTCCTACGCGGCTCATACACAATATACACGCTTTTAAAAAAATCATAG

## >PflaOR11

ATGGAAATGGGTTGGTTCATTAAAAACGAAATGTATTCTGTGACTTTGTCTTTAACAATTTTGAAACTGGCTGGGTTCTGGTTACCATTAGAATTGAAGGGCTATAAAAGAAGTTTGTATAGATTGTACGGTTTCTTGTCTTTCATGTTTCTTACGGGCACATATCTCATCATACAAGTGGTGGATTTATTCATGATTTGGGGTAATTTACCACTAATGACCGGAACAGCATTTTTGCTATTCACAAACGTAGCCCAATGCACGAAAATTGTCAACATCCTGACAAGGCAGCAACTTATACAAAGCATCATTAGTGAAGCCGATTTGCTAATGAAAAGTGTGGACGATGAACAAGCTAAAAATGTTGTGAAAAGTTGTGATAAGAATACAAAACGGCTTCAAGTGTTTTATTACGTCCTGACAACAGTCACTGTAGCTGGTTGGGCCGGAAGCGCTGAGAAAAATCAACTACCCATGCGAGCTTGGTACCCGTATGACACAACCAAACTTGTCGCTCATGAACTAACATACGTCCATCAAGTGGGGGCTTTGTTCTTAGCAGCGTACCTAAACGTTGCTAAGGATACTCTCGTGACGTCGCTCATAGCTCAGTGTCGATGCCGGCTGCAATTACTTGGCCAGCAACTGAGGGATTTGTGCGAAGATCAACCTGCAAAGGTATTAAATTCTGATCAAGAATGTGTTGTATCTAGACGCCTTCGAAGTTGTGTCTTGAACCACCAATCGACTCTGGAAGCTGTGAATATGCTGCAAAACTGTTTTTCATTACCGACCTTCGTTCAATTTACTGTTTCCATGATCATCATTTGTGTTACTGCTTTTCAACTGTCTTCGCAAGTTGGGAATCCTGTTCGATTAATCTCGATGGGGACTTACTTACTAAATATGATGTTCCAAGTGTTCCTTTATTGTTACCAAGGAACCCAATTATCAGAGGAGAGTACTCGTATAGCAAGTGCAGCCTGTGCGAGTCCATGGTATGCTTATCCAACCCGTATCCGTCGTTCTCTATTGGTGATCATGACCAGGTCACAGCGCCCAGCGAGGCTGGTCGCCAGCGCATTTACAACGCTATCGCTTGCTTCATTCATGGCTATCATAAAGGCTTCGTATTCATTCTTCACCGTATTGCAACAAGTCGAAGAAAGAAGAACTTAG

## >PflaOR12

ATGGTGGAAAGTAATGTGGCATTGTTTCTAAGTCGTCCGAGAATTCTTTTAGGAATATTTGGAGCATGGTTACCGCCTAAAAAATATACAAAACTGCACAGAATGTATTTAATTTTTATCATGTCGACACAATATTGTTTTGCGTTATTCGAAATAATTTACATAGCCAACGTATGGGGGGATCTTGATGAAGTAACGGAAGCTTCGTATTTGTTATTCACGCAGTCCTCTCTGTGTTTTAAAACGACCATGTTTTTGTTAAATAAAAAGTATTTTACTGAGTTGATAACCGAAATGAATATCGACACTTACGCTCCGAAGACAGAGAACCATAAGAAGATACTTATGAAACACTCGCGTAAAATGAAACTGCTGGGCGCATTACTACTTAGTAGCGCAAGCTTAACATGTACCCTTTGGGCTGTTATGCCATTGTACGATGACGTCGAGAAAAGATCCTTTCCATATAAAATTTGGATGCCCGTGGATCCCGAGTTTTCACCAGATTATGAAATGGGGTACATCAGTCAAGTTGTAGCTATTTACATTAGCGCTATACTCTTTGTTGGCGTAGATACTACCGCGCTTTCCTTGATTATATTTGGGTGTGCTCAGCTGGATATCATCTCGGATAAAGTGCAAGAGGTAAAGAGTACAATATCTGATAATATAAATCGCGAAATGCTCAGAGAGCTATTTAAGGATAACTATGATCGTTTGGTCGAATGTATTCAACAACACCAAGCAATAATTCGATTCATGGATGGAGTCGAAAACACGTTCCATGCGCATATATGTTTTCAGCTCTGCGGTACCGTCGCTATCATCTGTATTATTGGACTACGAATTTCAATTGTGGAACCGAACAGTGTACAATTTTTTTCCATGCTAAACTACATGGTGACTATGATGTCACAATCGTTCCTTTGTTGCTGGTGTGGCCACGAGCTGACAACAGCGAGTGAAAATTTGTGTATTGCCTTATATAAGTGCAAATGGTATGAGCAAGACTTAAGGTTTCGCAAATTACTATGTATATTTAAGGAAAGGACGAGGAGACCCGCTTTGTTTAAAGCTGGACATTATATAGCGCTATCAAGGGCTACCTTTGTCACCATTCTCCGATCGTCTTATTCCTATTTCGCGGTGCTGAATCAGGCTAAGAAATAA

## >PflaOR13

ATGCTCTCGATTAAATTATCGGACGTTTTTAAAAATAACATATTTTTCTGGAAGATTTTTGCGTTATGGGATACAGAAAAACATAAGAAGATCTATAAAATATATTCAATTCTCTATTTGCTGACAACATATCTGATTTATAATGCACTACTTATTGTGAATCTTATATTTACGCCCCGAAAAATGGATTTGATCATTCGCGAATTTATATTTACGTTTACAGAGATAGCGATTGGATTCAAAATTTTGATGATAATTAGGAACAGAAATGAAATAACTTCAATCTTTGATATTCTTAATTCAAATCTATTCAAAGAAGATAACAAAACTGGAGATGTTTATAAATACACTATGTATTATAAAAAATATTTCAAAATGTTTGCTGTTCTGTCTAATTTTTCGTATTGTTCACAAGTTTTCGGATCGTTTATCGGCTATTTTTTATTCGATACGAATTTAGATTTACCGATATTTAAATATTATTTCTTAAGCGATGAAATAAGACATAGATATTTTGTTTTTTGGTTTGCTTATCAATCGATTGGTATGTATGGTCATATGATGTACAACGTTACAATTGATTCTTTTATAGCGGGCTTAATATTAATGGCTATGGCCCAATGTAAAGTTTTAAATAGCAAATTAAGTGATATTAAATTATCAAAACAAATGATACACGAATCATTTTATAATCAAGATCAAAGGCAACTATCTGAGTTGAATAATTGTTTAAAATACTTCGATCTGATTTTAAAATACTGTACAAGTGTTCAAAACTTGACCAGCGTAACACTTTTCGCCGAATTTGCTATGGCGTCGGCTATTTTCTGCGTAGCGATGTGCGGATTGCTATTGCCCTCAACAACGGAAACGTATATCTTTATGGTAACGTACTTGATGGCGCAGGTTTCAATCATTTACGTATACTGCGCTTTGGGGACACAAGTCACTGTTGAGATAAACAAATTAACTGACGCCGCTTACAATAGCGAATGGATACCGCGATCTAAGAACTACAAACTAAGTGTCGGACTCCTGATGCTGCGAGCCAATAGATCTGTAGTGCTGACTGGGATGAAGTTGTTCCCGGTATCACTCGGGACTTTTACCTCGATCATCAAAACTGCGTATTCATTTTTCACTCTGATCACAAATGTACAAGAACGCCAGGACAAAGGAACAATATAG

## >PflaOR14

ATGTTTAAAATAATCGGTTTATACTTCGATAAGGACGCTAATGATGGTTTTATATCGAAGTACGGTAGATATTTATTTTTTTCTACGTTATTCGTCATTTTTGTTACAAGTCAAGTGTTATTTGTTTTTCTAAGCCACAAGAATGATGCTAATTTTCTGGACATCATCAACGCTGGAGCGTGCACCTTGCTTGTGCTTGAAGACTTACTGAAACTAGTAGTGATAACTTTGAGAAAGGATAAAATCAAAAGAGTAATCGGAGATATTGCTGAGGTCTGGCCAAGAGACAATGGACTTAATAAGGAAAAAAGACAAATTATGTGCGAATGGTATGAAAAGATAAAGCGATTCGATGATGCTTTCTTCGTGTTTTCTGTCGTCGGGCTGGCAACATTCCTGTTTACGCCATTTTTTTACACTTTGTACTTCTACTTGATGGAAAAACAAACAAGATACTTGTTCCCATTTGACTTGTATCTTCTTATAGAAGTGGACTCGTTTGTGAAATACTTAATTGTATACACTTGCCAAGTTATACCAGCTTCATTTCTTCACATCTGCGTGTATGTTTCAAGTGACTTACTTCTTGTTGCGCTTGTGGGCAATCTCACAAGTTTGCTGTGTCTGATGAAATATGATCTTGAATACCTAACCATGCCGTATGATAATAACGAGGAAATAGATGACAATCAAGCCATTGATAACATTAAAGGAATTGTTCGTAACCATCAAAAACTGTTAAGAATTTGTGATGAATTAAATGAAATATTTGGTGTTATTCTACTCATTCTGGTATCATTTACATCACTTATTATTTGTTTCTTCGGATTCCTGACAGTGATTCTTGGAGGAATTAAAAATATCATCGCGGTTTGTGGAATGCTATTCGCTATTTTTGTACTAGTATGGCCGGGACAATTGTTGTATGATATGAGTGCCGACATTGCTGATGCTGTTTATTGTAGCTCCTGGTACCGACTGGGGACTTCGTTTAGGACTTATGTTTACATGATTATAATGAGATACTCAGTACATCGTGGTCCTATCTCTCGCTGGTGA

## >PflaOR15

ATGGGCTTTATTACAAATATTTGGAAGAAAATAACTTATACGAAAGCTTTGGAGACTTCCAGTGGTTATTTTGAAACAGCATTCTTTGAATCTGTGTATCGGGTCCTTTATTTAAATGGTTTATCAATGCATGATGGAGGTCTTTACCATATCTACAGTAAAGGTGTCCTCATTATGATGTTTCTCTTTGTATGCAGTGAAATATGGTATTTATTCATCTCTGATGACGGCTTGGATGGCTTTATACAAAACTTGACTGCATTAATAATACACCTCATTGCCGTGTACAGAAATATTAATATACTGCAACACCTGCACGTGTTTAAGAGACTAGCGAAAGCTATGGAGTCGCCTTACTTCGATATATCGACAGAGGAACGTAAAAAGATTATGTACATGTGGCAGCAACGTAACGAGAGGTTTCTCATGTTGCTGCTGGGTATCGGGACTTGTACTTTGGCTGCTTGGAACATATATCCTTTGGTAGACGATCTGGACTATAATCTGCCGGTTGCGATCAGTCTCCCCTACGACTACCGCAATCCAATTCTCTATCCGATCACGTATGTGTGCGTCGTTACCGCTTTCAATTACACTTCGTATTACGTGATGGTCAACGATCTCATCATGCAGGCGTATCTGATGCATTTGCTATGTCAATACAACGTGTTGTGTGACTGCTTTGAGAATATACTGCAGGATTGCGGTTTTGGATCAAAAGCAAACAACACGACCGCTTTGCTCAAGAATACATATTTGTCCGAAAAATTTAATCAACGCCTCGGTGATTTGGTCAATCAACACAAGCTTTTGCTCAGTAACACTATGGACTTGAGAAATATACTGAGTATACCGATGCTGTTTCAATTTGCGGCCAGTACGATGCTAATTTGTTTTGTCAGCTATCAAGTTACACAGGCCATCACCGAAAGCATTACTAAGTTCCTGATGAATTTCTTTTATTTGAGCTACAACATGTTTGAACTGTTCATATTCTGTCGCTGGTGTGAAGAAATTAAGACTCGGAGTGAGCAGATCCGTCAGGCTGTGTACTGTTCGAAGTGGGAATGCGGAATGACTATGTTGCCAGGAGTACGCTCCCGTATAATGCTGATAATAGCTCGAGCCACTAAACCAATGGTGCTCAGCGCTGGTGGTCTCTATGAACTGTCGCTTAATTCTTATGCCACTTTGGTAAAAACGTCATATAGCGCACTGACGATTCTGCTTCGATTTCGGCAAGACTAA

## >PflaOR16

ATGATTTTTAAAACTACAACAAACGTTAAAATGCCTTTATACGGTCCAAATTATTGGATACTGAAGAAAATTGGATTACTGTTGCCCGATAATAAAATCGGGAAAGCATTTTATATATTGATGCATGAAATAATAGCTGTTTTTGTATTCACCCAATACATGGAGCTTTACATAGTGAAGTCCAACCTGGACGAGGTTATTACAAATCTGAAGATCTCGATGTTGAGCATCATCTGCATCTTGAAAGCGCACAACTTCATTATTAATCAGAAGAGCTGGATCAAAATTATAGATTATGTCACAGTTGCTGACCTGGATGAGAGGAGTAATGTGGATAAAGTGAAGAAAGCGATAATTGACAAATACACGAAGTATTGCCATCGAGTAACCCACTTTTACTGGGGATTGGTTTTCATGACTTTCATCACGGTGATAACCACGCCGCTGTTGAAGTATGCGACATCTGACAGTTTCCGAGAGGAATTACGAAACGGCACCGAGTACTTTCCTCACATATTCAGCTCTTGGATGCCATTTGATAAATATAATTTTCCCGGCTCTTGGATAACAGTAATGTGGCATATAGCTGGGTGCGCTTATGGAGCGACAATTATGGCGGCTTTCGATACTAGCATCATGGTTATAATGGTGTTCTTCGGTGGTAAGCTGGAGTTGCTTCGGGAGAGATGCAAGCAGATGTTGGGGTGCGGCGGAGCTGGTATAACCGATGAGCGCGCTAAGCAGATAATTCATCAGCTACATTGTGTTCATGTGGAAATATTGAAACATTCGAGACTGTTCAACTCCCTGCTGTCGCCGGTCATGTTCCTGTACGTCGTCATGTGTTCACTGATGCTCTGCGCGACCGCCTTCCAACTCACCTCAGCCACAAATATTACGCAGAAAATCTTGCTCGCCGAGTACTTTGTGTTCGGAATCGCTCAACTGTTCGTCTTCTGTTGGCACAGCAACGACGTCATCTTCAAGGGTCGGCAAGTTAGGTTGGGTCCCTACGAAAGCGAATGGTGGGCAGCGAGTGTGAAGCAGAGGAAGCACGTGATGATGCTCACTGCGCAGTTGAACATACTGCACGTGTACTCCGCGGGCCCGTTCACTGACCTCACTCTGTCAACCTTCCTTACGATCTTGAAAGGAGCCTACAGTTATTATACT

## >PflaOR17

ATGGAGGGAATAAAATTATCTTACGAGAGTATCACGCCTCATTTAGACAAATTACTGATCATGGGATTCTACTACGTCGATGCGGTGCCGAGTGAACGGAAGAAAAAGCTTTATAAAACTTTGTTCTTTCTAGCATTTTTAATCAACTTTTTGTATCTACTGCAAGTTTTTCTTCAAATGTATTGCTTACGACATAACTTCTCTAGATTCTGTGATGCGTCTCTGAATGCGTGTCCGTTCATAGTATGTATTTACAAGATGTACGCAATAGGATTTCGTTCTGAAAAAGTTAAAGGGCTGTTAAATATAATGAAAGGTCCAATATTCAACCGATCAGATGCAACTAATCGTGAAATTTTAAAAAACGGAGTGCGTGAAGCAAGCTTAACTTTACGAGTCCTACTACGCCTTGCCTTATGTGCGACTGCTCTTTGGGGATTTTACGTTCCAGCCCTGCATTTGACTGGTCACGAGATACAATTAACGCTGTGGTATCCTTTTAATCCATTCTCGAAAATAAGTCTACTTAGTGTGGCTATATTATACCCGTGGATTGTTGTGATTTATGCTGGTTGCAGCAACAGCATATTGGATTGTGTCACCGTTGCTTTCTTCCTTCAATGTAAGACACAACTTAAAGTTTTAAGATTTTTCATGTTAATTCAAGACTTGTATGCTGACGTGTTTGTGTGTCAGTTCGTGTTTACAGCGCCGTTGATTTGCTTAATTGCTTATAGGCTTGTAACTATAGGACCCACAAGTGCACAATATATTGCGTTGATATTCTTTTTAAATTACATGCTAATTCAGTTATTTGTTTACTGTTACTATGGCAACGAAGTAACACTCCAGAGTAATGAAATATTGACATCGTCCTATTGTATGAAGTGGCTGGAAATTCCAGTATCAATCCGCAGATCTCTTCTCATTATGATGGAAAGATTCAAGGTGCCAATAGTACCAACAGCTGGAGTCATTATACCTTTGTCTTTATATACTTACGTTAGAATTTTACGACTGTCCTACACGTTATACTCAGTTTTGCAACAATTCATAAATTAATAG

## >PflaOR18

ATGTTAATCGAATTGAAAAAATTTTTTCGAAAAAAAGACAGTCTAAGCCACACTGAAGCTCAGAGGAAAAAATGCAATTCTAACGTATTTACACCAATATTTTCATTGCCAAGAATTACAATTTCGTCGGCGATTTGGTTAAGCGAAGAATTCAGGAAGCGCTATTTGGTGGCAAACTTCGGAATGATTTGTTTTCTAGCGGTATACGTTCTGCAAGTGATGGCAATGGTCATGGCGAGAAACGACGCAGAACAACTATTTCAATGTTTCAGCGTGTTTTCATTCTGTTTCATGGGCTCGCTTAAATTATACTCTCTAAAGTGTTGCAGTAAATTTTGGAAGTTTCTCCTAGCTGAAGCATTAAAACTGGAAGAAGATTATGTTAATGTTGCTAGCAATGTTAGCGAATATGAATCTGATGGGGAGGAAGAAGAGAGAAAAGAGCAGAATTTTATTGAAAATGAAATAAAAGCGTATACAAAAAGATACGAATTCTTTTCCGGTTTACTGAGAAAGTTTTACACTTTCACTCTTCTGGTTTTTGTTATTTCTCCATATGCTGAGTATATTGTAAGAATGATGAGTTACATGAATACGACCATGGGATTGCCACACGTACTGCCCGTATGGGCACCATTCGCAAATTTAGATATTACGGTCTACGTAATCACCATTGTGGCGGAGATAATAGCTAGCACTTATTGTGTTTATGTCCACGTGATATTTGATGGCATGTTTGTCGGTATGACTGTATTCGTTTGCGGTCAATTCCGTTTGCTGCGTCATTGTTCGGAACGTATAGCTGGTTGCGGTCAAATGCTTAAGCCCATATTTTTGAGAGAACAACGTGCCCTTTACAGGACCATCAGATGCCATGAGAAGCATGTCAGTCTTGTTAACTCAATAACGATTTTAAATAAATTGTTAACAACCATTTTGGGAATATATTTCACATTGGCGACATTTACCCTATGCGCCGTTGCTGTTGAGTTGAATTCGAATAAACTGGGTATGATGGAGCTTATATCAACTTTGCAATACATGGGCGGTACATTGATACAATGTTTTATGTTCTGCTACTTTGGCGACGCAATGTCAAGTGAAAGCTTTGTCAAGATGGGTGAAGGTCCATTTGGTGCAGCATGGTGGTGTCTCAGTCCGCACACGCGTCGCTACCTCGCCACTCTCGGCGTGGGGATATCCAAACCTCACAGATTGCTGGCTGGTATTTTTCATTCTGCGGATTTACCTTGCTACGTTCAGATTATTCGCACAGCTTACAGTTACTTCGCTGTTCTCAGACAGTCTTAAAGCAATATAAACAAACGCTTTGCAACACATGCAAACTTCAATCTGCACTAA

## >PflaOR19

ATGGCTGAATTTGAAAGTTTACCGACCAACTACATTAACACCTTTGATCATTTTATTAAAATGATCAGATTTATTGGTGTAGACTTTTACGACGATCCCAAGAGTAGCTTCTTCAAGAGATACCGCAGGCATTTGTTTTTCACTACTATATTCATCACGTTCCTGACTTGCCAAATCATTTTCATCTTACTCAGTCCCAGTACCGATGCTACATTTTTTGATGTTGCCAACGCTACAGCCTGCACCATATTTGTGATCCAAGATCTCATCAAACTTGCACTATTGACTTACAAAAAGTACGAAATCAAAGGTGTTATCGCAGCCGTGGCTGAAGTTTGGCCAACAGACATACAGGATGAGAAGAGGAGGGAAATCTTCATGCATTGGAGGGCTAGGTCCAAGTTTTACAATGATGCTTTTATCATTGGCGCCGGTATTGGTTCTGTCGTGTTTGAACTGTCGCCGGTACTAACAACATTATATATTTACTTCGCTAAAGAGGACATAGTATATTTATATCCTTTCGACATATACGTACCTTTCGACGTGGACAATGTGTGGAAGTACATCTTCGTTTCTTTGTGTCAAATATTACCATCTACTTTCCTCCATTTCTGCGTATATATGCCGAGCAATTTCACTTTAACATCGTTAATGGGCAGTGTAACAGCAATCATGTCTCTGATACGACACGACCTCGAGAATATAATACAACCAAACGAAAACGAACATTCTGAACCAAGTCAACGAGAGATCGAAAATAATGTTATTAAAATTAGAGAAATAATTAAAAGCCATCAAACTTTACTTGAGGTCATTGACAAATTAAATGAAATATTTGGACTGGACATGTTTGTTTTGTTCACGTTTACATCTGTTATACTCTGTGGCTTCGGATTTCTGGCAGTGGTGGAAGGCGGTATTAAGAACTTTACAGCTGCCTTTGGATTACTCTCCGTGGTGTATCTGGTAGCGTATCCTGGACAAATTCTGGACGATATGAGTGTAAGTATTGGCGACGCTGCGTATCAAAGTCTGTGGTACGAACGCGACTGCAGCTTTAGGAAATACATTTTATTTATTATGCTAAGATCTCAGAAGCCTTGTCGTATGTCAGCCTTGGGATTCGCCGACTTGTATCTGCCGACATGTACAAAGATGCTGAGTACTTCTTATTCCTATTTATCTATGCTGAGACAAACATACCTGGACATGGATAGTTAA

## >PflaOR20

ATGTCTTCAAAAATTAAATCAAAAAGAGAATTAGAATTATTGAATAATTCAAAAATACGAAATTACATTCATTACATTGTCATACCGTTACAAATCGCCGGCTGCTGGGACTGGTACAGAAAACCAAAGGCAAAATATCAAATATTGGTCAACAATATCTATTTGTTTTTAGTTTTATTTTCACTGGTCAATTTAATGATGACCTTATTGGTCAGTCTATATACAGAATGGACGAACCTGATGGAAAATTTGGAAAAGATTGCCGATGGAATACCTGTATTTCTGGCTATTGCAATTATTGTATATTTCGCAACCCACAAACAAGGGTTGTACGAGCTAGTCGACTTCATGAACCTCAACTTTATGCACCACTCTGCCAGAGGCCTGAGCAATGTCACCATGAAACAGAGTTACGAAGCTGCTAAAAGATTCGCATATATTTACACAGCTTGTACTATAACCAGCGTATCCATTTATGTATTAATGCCAATCTTTATTCATATATGGACAAAGCAACCTTTGGACAATTGGGCATATATGGATGTAACACGGTTACCAATGAAAATTCTGGTGTTCCTCCGACAGTGTCTCGGACAGACTTTCGTCTGTCTTTCAATAGGGCAACTAGGTGTATTTTTCGCCGCGAACGCCATCTTGATCTGTGGCCAACTAGATTTGCTGTGCTGTAGCATTAGAAATGCTCGATACATTGCCCTATTGAAGAATGGAGTTACGCATCACGCGATCAAAATGACTCATTCGGTTGACCTGCAAAATGATGAGAGGCATCAACAAGTCGTAAACATTCCGTTTCACGAAGACTCTGTATATGATTATGACCAACATCACATTGATAAAAGTTTTACAAAAAGCACGCAATTTGATATATATGACAGAAGACATGATGATGCAACCTGCGAAGCGCTGCGTCACTGCGCTCTTATTTGCCAAACAGTAGCAGAGTATAAAGAGAGATTCGAAGACTTTGTGTCACCCTTGCTGGCGTTGAGAGTCGTTCAAGTCACAATGTACCTTTGTACGCTTTTGTACGCCGCAAGTGAAAAATTCGACATGGTCAAAGTTGAATATCTTGGAGCCGTTGCATTGGATATGTTTATTTATTGTTACTATGGAAACCAAATAATTTTACAGGCAGATCGTGTGTCAACAGCTGCATACCAAAGCTCATGGTACACAATGGGAGTGCGTCCAAGGAGACTTCTCCTCAACCTTTTGCTGGCCAACAGAAGAGAAGTCAAAGTGCGCGCTGGGAGGTTCTTAACAATGGATTTACATACATATCTTGTTATTATCAAGACCTCATTCTCTTACTACACCTTATTAGCGAATGTCAACGAGAAGTAG

## >PflaOR21

ATGAAGTTAAATTTCGAAGTGATTTATAAAATTACATTACGAGCTCTAAGTTTTACAGGAATAGATCCATGGATACGTCGTGATAAGATATGGGCATTGCGTTTTCTTTTACTGCACGGACCATTTCTGATAATGTTTATCATTTTAATGTACAGTATGCAAAATCACTTTTTCAGTGGAAATTACCCTCAACTGTGTGGCACTACCACAATTGCAATGGTTTTTATCGGTGCATCTCTCCAATACCTTGTTGGAGTGGTATATCAAAATGAATTACAGTGTCTTATTAATATAATGATTTCGGATTATGAAATGGCCGATCATTTGGATGAAATAGAGCGAAATTTTGTTGTAAAGTATGCCATAAAAGGTGGTGGGATGTCTAGGCTGTGGCTGATCCTTATTGGCCTAGCCGGAGGACTGTTTCCAATGAAAGCAATAGTTCTCATGATTTATTCTTGGACACAGGGATCATTTCAATTATATCCCATATTCGACTTATACCATCGGGATATTATGGAATCTCAAAGTAAAATTGGCATGTACATTTTTTGGAACCTTTTCATATGGATGTATAATATTTACGCTTCCCTCATTTTCATTGGGGTCGAACCACTGGCGCCTATATTTTTAATACACGCGTGCGGCCAATTAGAAATACTTGCTCATAGAATCCAAAAACTCTACTCGGACGAAAACGACAACCAAAAACGGGTGGAGTTGATCAAAGCAATCGCTGTGCAATTGACTAATATTTACAGGTTCGTCGATATAGTAAACGAAATATTCAGAGCACTGTATGAAGTTCTTCTAAGAGCATGTGAAGTTGTTATACCCGTAACAGTGTTCTTTCTAGTCGAGGGTTACCGGGCTAACGAAATACGCATAGAATACCTAAACTTCGTTTTTGCCAGTATATCAATATGTTACTTTCCTTGCTATTACAGCAGCTTAGTCGGCGCGAAGAGCGAGAAAGTTCGACTGGCCTTTTATCAATGCGGTTGGGAGACGCAGAAAGACATACGCACACGCACCTCTCTGCTTATTCTTTTGACCCGCACCCTTCGCCCTACCACTATCAGGACAATATTACGTACACTGGACCTCGATACATTTAGCGATGCCTGTCACCACGCCTACGCATTGTTTAATCTTTTACTTACAATGTGGACATAA

## >PflaOR23

ATGATTCAGCTAAAAGATAATCCACACAGCTCTCTAATGGGACCTAACGTATTTAGTTTGAGATTCTGGGGATTATTGTTACCCGAAAAGAAAAACAAAAGATATTTCATCTGCTGGCTTGTTTATTTCTTCATGGTACTTTTTACTATCACCCAATACGTTGACGTATGGCTTATAAAATCTGATATCAATCTATTATTGCATAATTTAAAAATAACAATGTTAGCAACTGTAAGCATTACAAAATATAGCAATATTCTCATTTGGCAAAACAGATGGAGAAGAGTTATCGATTTCGTTATTAGATATGATTTCGAGCAAAAATACTCAACTAATGAAAATAAGCAAAGAATCATCAAAAAGTTTACTTCGTACTGCAAAAAAATAACTCTCATGTTCTGGTTGTTGAGTTACTCAACGGCAATCATGATGATACTGCAACCGTTTTCAATGTTAGTTATATCACCTCTATTTAGGGAAAATATTGCTAATGGTACTCAAACGTATCTCGAAATAGTAAGATCTTGGGTACCCTTTGACAAGACTACAAAATTAGGATATGCTGGTGCTTCAGTAATTCAAGCATACGCAACAGTCATAGCCAGCGGGATCATAGCTTCTTTCGATACTACATCTATAGTCATTATGGTTTTCTTTCATGCTCAATTTAAGTTGTTAAAAGAAGATTGTGCACATATTTTTGATTTTAATGGCAAACAGTTTATTAACTGTCATAAAAGACACGTTGAACTGATAAAATACTTCTACATCTTCGACTCCTGCCTTTCCCCGATTTTATTTCTCTATATGGTTGTCTGCTCCATCATGCTTTGTGTTACAGCATATCAGATAAATATCGAAACCATTCTAACACAAAGACTGTTCATGATGGAGCTGCTACTGTTCGCGGTGGCTCAGCTGTTCGTATACTGTTGGCATAGTAATGAACTTTATTATGAGAGTCAAGATGTTTTTCGGGGACCTTACGGAAGCCCTTGGTGGTCACAAACAGTAGCTACGCGCAAACAGCTTCACATCTTAGTAGCACAGCTAAATCACACTTTGGTTTTCTCAGCGGGTCCCTTTGCCAAGCTTACCATAGCAACATTTATATCTATATTAAAAGGGGCTTACAGTTATTATACTCTGCTCGGCCAGACCTATCAAGTCTAA

## >PflaOR24

ATGACCGAATTGGACGAATTTTCCGATGAATTCAAAGAGACTTTCGCCCGTTTTGTACATATGTTTAAATTAATCGGTATATATCTGTATGACGAACCCGTTGGTCATATTTTGTGGAGGCGTTGGCGATTTGTTTTATTTATGTTCTTATTAATAACGTTCTCTATATCACAAGTTTGTTATTTTGTTATTTATGACACAAAAAGTTTTGACCTATTTGACATTGCTAACAGTTCAGCTATTATTTTGATTCTCTGCAATGATGTCGTAAAATTAATAATATTACATTGGAAAAAAGATCAAATTAGAGAAGTCTTTGCGAATATGTCAGACATGTGGCCTGTATATTTAGAAGACAAAGCAAAGAAGAAGCTATTTGATCACTGGCTGCGGAAGATAAGGCTCTTTAACCGGACCATGGCTAGTTGTCTATATGGTGCCGCCCTTATGTTTCATCTGAGTCCATTCTTGTTGACAATGTACAACGTCGTTAGCGGCCAAAATACTTACGTATATCCTTTCAATCTCTACATTCCGATTGAAGATACCTCTATATCCAGATACCTGATGGTATATGTACTACAAATATTATCACCAATAATGACGCATACTTGTTTATATCAGACCAGTAATTTCTTACTTGTGGCGATGACCAACAATTTGACAACGCTGTTGTGTTTACTTCGACACGACTTGCAGACTATCAACGATGGTGAAGATTTACCAGAAGCAGCAAGAAATGAGCAAAAACAATTGGATCATCTCAGAAAATTGGTAAAGCGTCATCAGAAATTATTGAAAATGTCTGATGTGATCAATGACATTTTTGGATTCGATATGTTTTTGATTGTATCATCGATTTCAATCGTCATATGTTTCTTTGGATTTATCGCAGTGACAGTGGGCGGCATTTCACCTACGATTGCAGCCATCGGCATGTTGGTGACCATTTTCTTCTACGCTTGGCCGGGACAACTTTTATACGATATGATCACAAAAGCCGTGTACTTTGAGTGCTTTGGGATTTTCAAGTATAACATTATCAACTTTTACAAAGATCACCACGTCGTCCTGGTCATATTTATCTATGATGAGACGCGTTTACCAAGACGTCAATAG

## >PflaOR25

ATGGACGAGACATTCACAACCTTTCATATTGTTTTATCATTTGCTGGAATATCAATATTTGCTAAAAACAATTGGAATTCAAAACTATGGCTATGTTTGCAAGCTTTTAACTTTACCATCGGTCTCTTTACATTTATTTTCACAAGCGTTTTTGTTATGGTAAACATTGAAGACTTAGTGATTTTTATCGAAGGTGCATGTATTTGGACGACTGGTGTCATCATGTTTATAACTTTAGGCGTTTGTTTAGTATTCAGAAAGGATTTTAGAAGCTTTGTAACGGAGATAGTATTTAATGACAAACTTCTAGAAATGCCGTTAGTGGCATTTGTCGTGGCGTCGGACAGACGTGGAAAATTGGGCGAATTGAAATCTCTGGTTTTGGAATCTGAAGAGAAATTATTCAAGTTGACTAGAATACTTTTGAAAACTTACGTATTATGCGTGTGGTTATGTTCAACTTTGTATATTTGCGATCCAATTTACGGGATGATTGTAAGAGAAGACGAAAATTTGCGACTTTTAGCATTTGACATGTGGTTTCCGTGGGGAATGGAGAACTTTAATACATATGTGGTTTCGTTCATTTTCCACGCTTACGCTGGATACCTCTGTTGCATTGCATATCCTGGTCTCCAGTCTACTATAATACTTCTAGTCGGACAAACCATTCGGCACCTCAGGGTGATGAATTTCATCATGAATCACATGCAAGAGATAGCAAGGGAGCTGGCTGGAAATATAAAGGAACAAGAGTGTTGTTTAGAAATACTGTCTCAATGCGTCGACCATTACGTTTATGTAAAAAAGTTCAGCAATCGTGTGAACGTGATATGTCGGCCTTTCTACTTGGTACTCATACTGGTTGCTACAATACTCGTTTGTGTTTGTTCCGTCAAAATTGCTATTTCGGAAAAATTATCACCGGAAATAATGAAATACTACGTTCATGGATTCTGTTTCGTGCTGGTGGTACTCATGTTTTGCTTTTTGGGTCAAAAAGTTGAGAATGAGTGTGCAGCATTAGAAATGGCAGTGACCGAAAATTGGTACAATTTCGGCAGGAAATACAAAACCAATTTGAGAATTTTCAAAATGGCTGTCAGTCAACGAATGCCGATTTATATTTTTGGGTCGCTTACTTTATCATTACCAACGTTTACATGGTTCATAAAAAATGGTATGTCGTTCTTTACTCTCGTTATGTCGTTTGTGGAAGATTGA

## >PflaOR26

ATGGCTCTTACAGAATTAATGCAAACACCAGGAAAAGACCATTTCGCTTTCAATTTAAAATTTATGTATTATCTTGGGCTGTGGCCAAACAATTGTTCTGTTTGGCCTTACACGCTATTTTACAAATTTTACGATATAACGCTGTTCGTTTTGGCATTAATATACATTGTGATGTCTAATATCGGCACATATCAACACATAAATGACAGTGTCATTTTGTTGTCCAATACGGACAAAACTTTGGTTGCTTACAATTATGTTTTGAAGATACTTTATTTTATGAAGGGTCGGGATGATTTGAGATGGTTGATAACGGAAATAAGAAATTCCGGGGACGTAATTACCGACGAACGTAGTAAATTGATGACTATGCATGTTGTAGGCGTTGTCGCGTTTGGTACCGCTCTAGGCGGAATATTTAGTTTTGTGTCATTTTTCGATGGCAAAATGACTGTTGAAAGTTGGATGCCATTTGATGCAATGAAATCTAAGATGAGCCTTGCCTTAGCAAATCAAATAATTGCTATTACTTTCTTGATTCCTGTCGTGTACAGAGGTTTCGCTTTGCAAGGCATAATATGCAGTATAGTCATGTATTTTTGCGACCAGCTAAGGGATATACAAAATCGGATGCGTAACTTAAAATACACGATTGAAACCGAAGATACTTTTAGACAAGAGTTTAGAGAAATAATCAAAAAACATATTCGAATAATGAGGTATCCGAAGAATTCGAGGCCGAACAGAATCCACTTACTATGGATTGTTAAAAGAGAGCATGAATTGAAATGGCTTGCTGATATAGCTAACAAAACTTTGGTCGAGTTAAGGGAAGCCAATCGAATAGATAGATTGCATTTAGAATTTTATGTCACGAGTGATCCAGAAACACGAAATAAATCTGATAAACCAGTGGATATAGTTATAGTCAATGAAAACGGTAAAATAAACAATGTTATATTTAGCAAAAAGAATACGGACGAAAATGTTGCATTATTGAGACCTTTTAATAAAGACAAAGTTGATATAAATATAAAATCCACTAATGTAGATAATAATACTGAAGATAGTTACGATATAACAAAACAATATCCTATATTAGGATGTAGAGTTAAACGAGGGAGACCTCATTGGAATAGGATGTTTGGATACTGGGTACATTTGTATCCTGGGGAATATTTAGATGTATATTGCTGCGGACCAAAGAACTTAGTGAATGAAATAAGAAAAAAGTGTAAACTAATTACAAGGACGACAAAAACCCAATTAAATTTTGTTCAC

## >PflaOR27

ATGTATAGAGATGAGCGTATCTATAGTCTAGTAAAGATTCTGCTTTTTCTTATCCCTGTTTGGTCGAAACCAAGGATAGGTACTTTGATCTTAAAATACTTTTCTCTTGTGTTAATCGTTATTTTCCAGTCTCGACTTGCTATGGTGTTTTTCAAGAAATACGACGATTTAGCTGGCGATTTTATAACAAAAATCCATCTTTTCAACCACAAAGAAAAGTCTGATTTTGCTATGAAGACACATATACTTGTACACAAAATGTCACACTTTTTCACAATGTATCTAAATTTTCTAATGATCATGGGTATTATACTCTTCAATGCGACACCATTCTACAATAATCTGACCTCTGGTGTTTTTCAAAGAGACAAACCGGCGAATGTGACTTTTGAACACGCTGTATATTATGGCTTACCATTTGATTATACAACTAATTTCAAAGGTTTTCTCGTTGTGTTTTGTTTTAATCTATACATAACCTGTACTTGCTCATTCGCCTTTTGTACAATTGACTTGCTTACTGCCCTAATGGTGTTTCACGTGTGGGGTCATTTGAAAATTCTCATTTATAATCTTGAGAGTTTCCCGAGGCCTGCAATTGAGTCCTTAGAAGAAACGGGAAGTGTTTTAATATGTCAAATGTATACACAAGAAGAAATGCGAAGAACACGTTTATTACTGTGTGAATATATACATCATCATCGTCTTATATCTGAGTAA

## >PflaOR28

ATGAAGAAGTGTATCAAAACTTTTTTTAACAAAGATGATATTGACTTTACCAGCTGTGACGTAGATCCGCTCGAATTTCACAAAACTTTCTATTTTATCATGAATGTTTATCAAATTATTAAAACAATTAATTCGCCAATGTACATGTACATTATCAAATATTGTGTATTTGTTTGCGGTTTGTCGACTTCTGCCATCATCATCACCTCCCTAGCCCACAGTATACAAGTGTCAAGTATACCTTTGATCATCGAATGTGTCACTTACGCCGTGACAATATTTTACGGAGTTCTTATATCTTTATGTTCCAAAATTAATGTCGAATGTTATAGAAACCTTGAAAGGATTATAAGACAAGATTTTCAGTATGTCAATGGAAAAGCAAGAAAATACAGGAAACCTTATTATGATCTTCAACTTAAAACATGGAAGCTATCATTAATTTCAATAATATTCACGATCGGTGTTACAATCGGTATTGACATATTCAATGTGGCCTGGATAGTCGTAAGCTATTTAACCAGAAACCCGGAAGAGAAATGGGAGAGGCCAAAGATTTTTCCAGTCTGGACGTTCGAAATAGATTATGAGGTTACACCAGCATACGAATTGTTCTTCGTTTACGCACACATATGTGTATTTTTTTATGGATTCAATTACTTGTTTTCGATTCACACTCAAATAGTTTGGGTTGGACAAATAACATCAAAAATTGACCTCATTTCCTGGCTCATCAAGGACCTTCTAGTGGATATTCGTCCAACAAGGAACCATAAGAAGAAACTATTTCACAACAACCTTATTAAATTGCGAATGAGAGAAATTATAATACAACATCACTCTGCATACGAATTACTGGAGAATTATAGTGGAGTTTATAAAAAAATGCTAATGGTAGAACAGAAATTAGCGCCACCATTAATTTGCTTAACAGCATATTGCTCTGCCGAGCAACTGGAAGAAACTGGTGAGGTTAACGGTATACTTATGATACTCTGCGGTGCCATTATTATCCAGTACTCGATACCTAATATTTTGTGCACATTCTTGGGAACTAAAATTAGGTCGATCAATGAAGTTTGTTTCAACACATTATATTGGAACCCAGGTGCAGTTTTGCGTCCATATATGATCCTCATGATGCAGCGGTGTATGAGGCCGCTACCCATACAGGCGCGCGGCTTCAAGCCAGTCTCCCTCGAGACATTTTCTAAAAAAATCACATCTGCATATTCAATGTTCAATATGCTGAGACAAGCAAACTTAAAATAA

## >PflaOR29

ATGGAGGAAAAATCAAAGGACAATCATTACTTAGAACCTCCAAAAGAGCAAACATTCTATCGAAGATTTGCATTCTGGATGACGTTTATAGACATCGGAAACCAGGAGTGGTGGGGCTATGAAACACCTAACAAGCTGATAGTATTCCACTCGAAACTTTTGAAATACTTTCCAACCGTTTGTGTGTTTTTGGAGATCGTATATTATTACGTGCATTCCCATGAGCTAGAACTGGCTGTGAAGTCAAGTATATTCATTATCATGTTCATGACGATAATGATAACCGTTAAAATCTGGGCTGGCCACCGAGGTGTTCATGAAAAAGCTGTAACAAAATTTTTTCGGAAATACCATCTCTACAATTATTATCAAGAAGAGCGTAACGATTTTGTTAAAAATACACTCATAACTGCCGAAAAATTAACCAGATGGACGCTAGGGATATGGTTTCTCATAATGATGTTTGACTGGGCGCTGTGGATGATTATGCCGATTCTGTATAACGTACAACATAGACCAGAAATCGAGAACAGAACAAGTCAACTGCGCACATGCCTATACATGTGGACACCGTTCGACTACAGATACAATTATGGGACCTGGGTGATAGTACATATAGTAAACGTTTATTTAACCGGTTTAGGGTGTTACTACCTTGTCTGGATTGATGTATTAAACTATGCAATCTTATTTCATTTACATGGACACATAAAAATTTTAAAACATAAAATCTTAACTGATTTCGAAGGGGATTTGAATGATATGGAAATTAAGAAGAGGCTGGGAAAAATTGTTGAATACCATGTTTTTATTCTATTAACTTTCAAAGAAACAGAGGCGGCTTTTGGATTAAACGTGGGCGTCAACTACCTTTACAACCTAATTACTGATAGTATTTTATTATTTTTGATGATGCGTGTGGAAAGACAAGATAAGGTTACTTTCGCAATAATGTTGATGATGTGGATGGGAGCAATGATATTGTTTTCATTTGGACTTGAAGAAATTAAAATACAAAGCGACACGTTGCCACAGATAATGTATGACGTACCTTGGGAGCATTGGTCACTTTCTAACCAGAAAATGGTGGTCCTGCTGTTGTTCCGAATGCAGCCTGAACTCGCTTTTAAAGCCACAGGTGGAATGGCAACAGGAGTGAGACCCATGACCTCAATAATAAAATCGACGTTTTCATACTACGTTATGTTGAAGTCTATGGGTGGTGAACAA

## >PflaOR30

ATGTCGTTTAGAAATACGGGTAACGAAACTCTGACGGTTTTACAAATGAATAGAGTGTACGAATATTTCTATATGACAGGAATGACTAAATTTGAAATAGGAATACCAAAGAATTGGTCAGAAAATTTCATCCAATGGCATCGTACATTTTCAAGATTTATTAGTATTTGCATTTATCTCTTTATGATTTCCGAATGGTTATCCTACCTAACTCAGTTAGATCTGAACGAACAAGAGAGATCTGACTTTATAGTATTTTCTTTCTCGCATCCTATGTTGTATTCATACTGTTATTATATGTCTTGTAAACAGTCATATGTGAAAAGGATGAATAAACTTTTGGTTTATATGAAAACCGTTTACAACGACGAAGAGGAGGAACGGCGGATGATTACAAAAGGCAAAATATACATCACAGTGACAGTGGCCTGTATATTTATTACATTATCTTTGCATGGTTTGGATGCATTATTGAAATGTATTCTTTCAGATGGTACCTTCACAACCGTTATCACCGCATGGCCCGAGGCTCACGACACCCGCCCAGCAGCCGGCGTAGTCAGGGTTGTTGCATACATCATTTGGTGGATCTTCATGATCAGGGTCTCCTCTGCTATGTGTCTCGTTATGGCCTTTACTATAAACTCGTGTAGTCTATATTCACACCTCCAAAGTTATTTCAAAAGTCTTACTAAAATATTTCTTGAAGACCTAAACCAAGATGAGAAGGAAAAGAAATATGAAGAAGCCTTAAAAGTCGGAATCACACTTCATAAAGGCTGTCTGAAGTATACGAAGGATGTCAGTCGGCTGTGCAATATGCCTTACGGAATGCAAATTGTCCTCAATATTTCTGTAATGGCGATGTTTATGATAAAAATGGTGAATTCTGAACGTTCCTATACGAACGTATTGTACACCGTGACTTCGGGATCAGCTATGTTGTTCAGCACCGGATTGTACATGTGGAATGTCGGTGACATTACTGTCGAGGCATCCCGAGTGGCAACAGCAATTTGGATGTCAGGATGGGAGAATTGTCACATGAGATCTGTTAGGATCAGGAAACTGCTGATGATTGCAATGGTGCAAGCACAGAATCCTGTCATCATAAGAACGCTCGGCAACATACCAATTTCTTATCAGTCGTACTTGTCGATAGTCAAGTTGTCATACTCAATATTTTCAGTTCTGTATTAA

## >PflaOR33

ATGGAAGCGTCAACATTTGGTGAAATTTTAAAACCAATCAAAATTAGTTTCGGTTTTTTAGGAATACCATACAAAGAGAAACGTCTCGGATTACGATTTTGTTTTATATTTACTATTTTGTTCATAACAGTTATGGAAGAATTTGGATTCATCATTTCGAAAATTTCTTCTGATAGTTTCCTAGAAATAACTCTAGTAGCTCCATGTGTTTGTACAGGAATTTTATCTGAACTTAAATTTTGGTTTGTTGTTCGTAAGAGATCAAAAGTTTTCGATTTAATAGAAACATTTTCTGATTTCTACGACCGTATTTATCGAGATTCAGAGAAAATTACCTTAATTAAGAAGGATATTATGAATTTAAAAACATTGATAAATTACATCTTTATATTGAACATCAGTCTCATAACGGTTTATAGTTTTTCAACTTTACTATTAATGTTATATTTTTATGTAACGAGGAAGGAAATAGTTTTCCTAATGCCTTTTGCTCTTTGGGTTCCATTTGAAATCGACAATTGGTACGAATGGAGTGTAGTATATTTGCATTGCTGTGTTAGTGGGTTTATTTGTGTTTTGTATTTCTCGACTGTAGACGCCCTATATTGTATCCTCACATGCTGTGTCTGTTGCAACATGACCATAATTAAACAAGATATACTGAATTTAGACTTCAATGATGATAACATTACACAAAATGTCAAGGAGATTGTAAAAAGTCATCAATACGTCTTGAAGTTGGCGCAAGATTTGGAAGATATCTTCGCAGCACCTAACTTGTGCAATGTTTTTGTGGGTTCCGTTGATATTTGCATCATAGGATTTAATTTAACGTTTGGAGAATACTCTACTGTACCAGGTTCAGTTGTATTCCTACTAACAGTCATTTTGCAAATTTTGATGATGAGTATTTTCGGAGAAAATATTATTACGGAGAGCACGAACATTGGACAAGCTGCTTATATGTGTGACTGGTACAAAATGGATGCACCAACGAAAAAGATGATATTACTTATTCAGTTACGAGCTAGCAAACCACAAATACTGACAGCCTACAAGTTTTCAATCATATCATACGCCAGTTTTTCTAAGATAATGAGTTCTTCGTGGACCTATTTTACTTTACTCCGAACTGTGTATAATTCCGATGGAAATACTAGCAACAAACAATGA

## >PflaOR34

ATGTCGTACAAACAAATCGATTGCTTTCATACGCAATACAAAATTTGGAAAATTTTCGGCATAAGTTTTAATTTCAATTCCGAATATTATAAGGTCTACTGTAAAATCTTTTTATCATTTTTTGTTTTCCTGTATCTAGCACTTGCCACTTTGAATTTCTTTTTTATACAGCTAACAATGGACGTAATAATTGAAGAGACGATATTTTATTTTACAGTTTTATCTACCATTTCTAAAGTATTAACGTTTCTTATTTTGAGTGAAAAACTTATGGTTATTCTAAATGATTTGGAGAGCGAAAGATTCCAACCTAAAACTCGAAAAGAATGTGAAATATTAGATAAAGCTAAGCGTTTTAATGTTTTTTATCGTAAAATTGTTACTATAGTTTCTCTTGTATCACACATAACACATATTGGATCTCCTGTTATACTACATGTTGTTTCAAAATCAAAATTGGAACTACCTTTATGTAGTTATTCGTTTTTGACGGATGACTTTAAAGATAAATATATTTATCCCTTGTATTTGTACCAATGCTTGAGTATACAATGTCTTCTGTGGTACAATGTGAGTATAGACACATTTTTTCTTGGTTTGATGATACTTTCAATAGCACAATTAGATATTCTGGATATGAAACTACGTTCTGTAACAAGTGATGTTAAACAAAACCCAAAAATCGAAACTGGCGATATTAATGAACATAATAAATTGGCAACAGATAACCTGGTTAATTGTTTCAAACATTATGAGGACGTGTGCAAATTTGTCACCTTGATAGAAAGCGTATTTAGTGTTACGCTTTTCATCCAGTTCAGTATTGCTTCGAGCGTAATCTGCGTTTGTCTTTTCAGATTCACTTTGCCGGCGCCATTACAATATTACGTTTTCCTCGGAAGTTATTTATTCATAATGATAATTCAGATAATGGTTCCATCGTGGTACGGTACACGCATAATAGACAAGAGCCAAAACCTATCTCTATCTTTATATGACTCCAATTGGACCGTGGAATCGCTCCGTTTCAAGAACAATCTGAAGATTTTCGTCGAAAGAGCTAAGCGACCACTTTCTATTGTTGGCTGGAAAATGTTTCCTTTGTCGCTGGGCACTTTCACTTCTATTATGAATTCCGCGTATTCGTTCTTCACACTACTTCGTCACATGCAATCTCTGGAGGCGCAGAAATAA

## >PflaOR35

ATGAAGTTAGATTTCGAAGACATTTATAAATTTTCATTTGGAGCTTTGTATTTGACAGGAATACATCCATCTATACGCCGAGACAAGAAGTGGGCCTTACAATTTCTTATATTACACGGATCGTACCTAATGTTGTTTCTTGTAATGGTGTATAGCATGCAATATCAATTTCTCAATGGCCTGTACACTGAACTTTGTGGTACTGCATCTATGGCCGTGATATTCTTCGTTGTGTCCATTCAATATACTATTGGGTTGGTATATCAAGACGATTTGCAGGAACTTATTGATTGTATGAAAACAGATTATGAAGTTGCTGAGGAATTAGATGATATAGAACAGGAAATTGTTGTTAATTATGCTATGCGAGGAGGATGGATGTCGAAAATGTGGTTGGTTGTTATATCATTTACTGGAATACTGTTCCCATTAAAAGCATTTCTTCTTATGTTATACTACTGGGCAAAAGGAACATTCCGATTCGTTCCATTATTTGATTTATACCACCCCGATGTTATGGAATCACATTTGAATAATGGAGTAGGAATGTTTATTTTCTGGTTTAGTTTTTCGTGGTTTTACAATATTTATGCCTGTACCGCTTTCATTGGTTTCGAAGCTCTGGCGCCTATGTTTGTAATACACGCGTGCGGTCAGTTGGAAATTATCAGTAACAGAATCTACAAACTCTTCTTGGAGGAAAATAACAATAAAAAGCGCGTGGAACTGATCAAAACAATTGCACAGCATTTAATCCATATATACAGATTCATAGATAGAGTAAACGAGATATTTAGAGTGCTGTATGAATTAGGACTGAAAGCATGTGAAATCACGATACCTGTTACACTGTTCTTAATGCTTGAGGGTTACCGCACCGGTCAATTGCGTTTAGAATACATAAACTTTACTGTGGCCAGCCTAGTTCTATGCTACGTTCCCTGTTATTACAGTAGCTTACTTACTGAAAAGGGTGAAGACGTTCGACTGGCGCTGTACCAATGCGGTTGGGAGAGGCAGAGAGACCTGCGCACGCGCAACTCTCTCCTTATTCTCCTCACCCGCGCACTTCGACCAACTGCTATCAGCACAGTATTCCACCCTATGGACCTGGATACTTTCAGCAGCGCCTGCAACCATGCCTACGCGTTGTTTAATCTTTTAAATACAATGTGGAAGTAA

## >PflaOR36

ATGCAAAACTCGGACTCTGCAGACAGGACGGTACCCACGCAGTATGTCCGGAATTCTCGTGCATTCAGACAATTCAAGAATCCACCTCAACCACACATGTGTATCCAAGATACAATTAAAAATACTACAGAAAAATTATTTATAAACGTACTAGGCTGGCAAAAAATAGCCAATCCTAAGCAATATTCAGATCCAATTCCTTTATATGGAGGGATGCAGATTACTCAAGGATGTGGCCCAAATACTAACCGACCTCCACTTCTAGTGTTTGCAGTGATGGTGAACCCGGATATATTAAAAGCGAATGGAAAAAATGCCCTCAATCTTTCTGACCGTGATGCCTTAGTGAGCCTCCTTTGTGACTTTGTAGAAGCTATGAATCCTGGTTTACTATTAACACGGAATCCCATTATCCTAAAAGATAGGGACCTTGCTGGGGAATTAAAGGATGTCTGGCTTGCAGTTCAAAAAAAACGTGAAAGAGAAAAAGAAGTTAATCAAGATGTCATGTATAAGGTGTATGATATTGACGGCATTGGTAATGAAGATGTCAATATTGAAGACAAATCAAATGGCATTGATAGTTGCAATCAAAAGGATAATAATTCACCAAATAAATCACATTATAATGATACGAAGAGTATAATTAAATCTTCTAAACAAATTCTCTTAAATGCTGGTCAAAAGTCTGAATTTGATGCTGGTATGAATAATCAAAATTTGTCAAACAGAGACAATAGATGTACCAACAGTAATGAAGATTCAACATATTGCACTCAAGTATACAGACAAATTATGTCGACAAGGGAATATTCATGTCAAAACAATGAAGTCGATCAAATATTCCTCACTAATGAAAGTACGAAGCCCTCAACCTCATTCCGAAAAGATTGGAGTCCGACACAGGACAAGTCAGGACAGTGGGATGAATTTGCCAAACGTAACATAGCAACGATAAAACACAACGAAGCTGACGCTAGACAGCAATGCTTCAACAATAGTAACAGTAACAATCCTGTAACTAATGGCAAATATTCCTTCTTTCCTGTATTTAACAATAAGGAAGAGGATCCAGATATAATGAATAGCAAAGAACAGGTAAGAACTGATGACAAATCCAAAATTATTTTAGACCCAATGCAGAAATTAGTTCTGCATTCGACTGATAAACATATATGTGATAATAAAACAAGTGCTCTCAGTTCATTAAGCTCATAA

## >PflaOR37

ATGGAATTGGATTTCGATAAAGTCTTTGTTATCTCAAATCGTGCATTAAGACTATGCCGTGCACATCCATACGTAAAGAGGAATTTATCATGGGCTGTGTCATTTTTATTCCTTCATGGAATATTTACTTTCACTTTCATACTCATTGCCCATACCGAAATATATTTTTTGAAAGAACAAAGTGCTCCCGATATATGTAAGAACGTCATATTTATGACGGTGTACTGTGTTGTAACCTTCATGCACTTCATGATGACGTTTTATTTGAAAGACATAAAAACACTAATAAAAACAATGAAGTTGGACTACAAAGCTGCTCTTAAACTATCAGAAAAGGAACTGATTGTTGTTAAGGAGTATGCGTCGAAAGGAAGATTTGTCACAAAACTGTGGTTATATATATGCTTGGCGGTCGGGATATTGTTTCCCTTGAAGTACTGGTTAAAGACGATTTACTCATATATTATAGGTGAACCTAAATTACAACTGCTGTTTCATGAGTACTCTCCAGACATAATGTCCGCGACAAACGATATAAAATTTTATTTAATACTAGCTGCTGTAACTGTTTTCTACGATATTTACGCCAATTTCATGTACGTTGGATTTGCTCCATTTTGCCCGATAATTATTCTACACGCCTGCGGTCAAATGGGGATCATTAAGTTGAGAATGGAAACACTTTTTCCGAAAACTGGATATAGCTCTTCAGAAACCATATCAAGATTAAAGGACATAATAAGACGATTACAAAATATTTATAGCTTCGTAAATTTAATTAACGAAGTGTTCACTACATTTTATGAATTTTTAATAACCGCCGGCGCTATTCTTTTGCCGTTCATAATTTTTATTGTAATTGAGGAAGCTCGTAATGCTGAATTTAGTATGGAATATGTCTCTTTTATCTTCGGCACGGTTGTTATTATATACTTACCGTGTTGTTACAGCAGTATTCTTATGGAAATGAGTGAACAAGCAAAGTTGGCATTATACGCTTCTGGCTGGGAGTCTCATATGGACAGACGCGTACGTAGTCTAATTTTGGTGATATTAACTCGCGCAGCAAAACCGGTCGTGGTTAAAGCAATATTCCGTGATTTGAATCTCAACACATTTAGTGAGGAAGCTCGTAATGCTGAATTTAGTATGGAATATGTCTCTTTTATCTTCGGCACGGTTGTTATTATATACTTACCGTGTTGTTACAGCAGTATTCTTATGGAAATGAGTGAACAAGCAAAGTTGGCATTATACGCTTCTGGCTGGGAGTCTCATATGGACAGACGCGTACGTAGTCTAATTTTGGTGATATTAACTCGCGCAGCAAAACCGGTCGTGGTTAAAGCAATATTCCGTGATTTGAATCTCAACACATTTAGTGAGTTCTGTCATCTTGCTTACGGCATCTTCAATATTCTAAATACAATATGGGAGTGA

## >PflaOR38

ATGTTCATAACAGTTATGGAAGAACTTGCATTCATCATTTCGAAAATTTCTTCTGATAGTTTCTTAGAAATAACTCAAGTAGCTCCGTGTTTTTGCATAGGAATTTTATCTCAACTAAAATTTTGGTTGGTTATTAATAAGCGATCCAAAGTTTTTGGCTTAACAGAAATACTGTCGGATTTCTACGACCGTATTTATCAAGATTCAGGAAAGATTAACTTAATTAGAAAGGATATTATGAATTTAAAAAGATTGGTAAAGTATATCTTCATCCTGAACATCAGTCTTATAACGGTTTATAATTTTTCAAGTTTGCTTTTAATGTTATACTTTTACGTCACCAGAAAAGAAATAGTTTTTAGTTATCCTTTTGCCATTTGGGTTCCCTTTGAAATAGACAATTTGTATAAATGGAGTATGGAGTATACGCATTGTTTTATCAGTGGATTTATTTGTGTTCTCTATTTCTCGACTGTAGACGCTCTGTATTGTATACTCACATGCTGTGTCTGCTGCAACATGATCATTATTAAACACGACATACTGCAACTCAGCTTCAATGATGATGATATTGCGCAAAATATCAAGGAGATTGTTAAAAAGCATCAGTATGTCTTGAGGTTGGCCGAAGATTTGGAAGAAATTTACGCAGCACCTAACTTGTGCAATGTACTGGTGGGTTCCGTTGAGATCTGCATCATCGGATTCAATTTAACCTTTGGGGATTATTCCACTATACCAGGTTCAGTTCTATTCCTACTATCAGTCGTATTGCAAATTTTGCTGATGAGTGTTTTCGGAGAAAATATTATTACAGAGAGCACGAACATCGGGCAAGCAGCTTATATGTGTGACTGGTACAACATGGATGCTCCAACGAAAAAGTCGATATTTCTTATTCAGCTGCGGGCTAGCAAACCACAATCTCTGACGGCCTACAAGTTTTCAATCATATCATACGCCAGTTTCTCTAAGATAATGAGTACTTCGTGGACCTATTTTACTTTACTAAGAACTGTGTATAACTCCGATGGATAA

## >PflaOR40

ATGATGCTAAATTTCAAGGAGATTTATAAAATTTCACTAGGAGCTTTATATTTGACGGGTATACACCCATCGATACGTCGTGACAAGAAGTGGGCCTTACATTTTCTTTTACTTCACGGATCGTTTCTGATAATGTTCATAATATTAATGTACGGCATGCAATATCAATTTTTCAATGGCCTGTATACTGAACTCTGTGGTACTGCAACTATGGCTGTGGTATTTGTTGATGTATCTATCCAATACACTATAGGCTTGGTATTTCAAGATGATTTGCGGGAACTTATGGAGACGATTATAAAGGACTATGAAGCCGCTGATGAACTGGACGACACAGAACGGAATCTGGTTGTAAATTATGCGATGCGAGGAAGGTGGATGTCGAGGATGTGGTTGGTGGTTATATCGCTGACAGGAATATTGTTTCCAATGAAAGCATTTATTCTCATGTCATATTATGGCATCAGAGGAAAATTCCGTTTGGAACCGTTATATGATTTATTCCATCCTGATATTATGGAAGCGCAGATGAATAACGGTGTCGCCGTGTTTATTTTTTGGTTTGGTTTCTCATGGTTGTATAATATTTACGCTTGTACTGTTTTCATTGGGTTCGAAGCTCTGGCACCCATGTTTGTAATACATGCATGCGGTCAGTTGGAGATAGTGAGTTATAGAATCTATAAACTCTTCTTGGACGAAAATGACAATCGAAAACGTGCGGAATTAATCAAAGTAATTACAAAGCGTTTGGTTCACATTTACAGGTACCCACTATTTTATACTAGATCTATTCCTATGGATATGTACTCGTATGTGCTGAAATTAAGTTAA

## >PflaOR41

ATGAAAGCCTTATCGACCAACCTCAATTCTTTTAAAAAGTTTGCTCAGCGTATGTTTCATGATGATAATATAGATGATATCTTGTTGATACCGATGCTACTTAAAGACGCTAGTGTTGTATTCTGGATACTTTATCACTTGCTGTATCTGTATGTGTACGTAATTGGATCAACCGCTTATCACATAACATATTCGAAGACCGTCATCGACTACATAAAGGGTATCGTCGCAATATCCATTACAATCATTATAGGAAATAATAGTCACTGGTTTATTGAAAAAAGATTAACGCTAAAAGAAATCCTGGAAAAGCTGAAAGAGAACAATAAACGCACTATAGAATTATTAGCTTTGAAAAAAGAGTTTCACAGATTAATGATCATCATAAAGGGAATAACAGTTGCTTTCTATTTGACTAATGTACTAAATAGTCTAGTCATATTTTTACCCAGTCGCGTATTGCATGTGCCGAATGATTATTCTATGACTCTTTGTTACGGATTGGAGCCATTAACAGAATCACCTAATTATCAAATATGCTTCATCATAATGAGCATCACGGATATCTCCAGCGTCATAATCGTTCTACACTATCAAACGATCTTAATTTTCTTCGTTCTACACACCATTATCACATTTCAGATTCAATCCAAGCATTTAACAAGTTGTTTCATTGATATTACAACCGAGATGTCTGTTAAACAAAATTTAAGCACTATTGTTAAACACCATACGCTTCTACTATACATTGTTTCTAGATTAAAGTATGCATACAGTGTACCCTTAGGTATAAACTTCGGCTGTAATGTTATCTGTGTCAGTATGTTTTTCTTTTTGCCAGAAGGTCAACACGTTTTTTACATTCCCATATTAGCTTACTGTTTTGCGACGTTTTTTCTATACTGTTTTCTTTGTCAAAAATTAGTGGACGCTTCGGGGTATTTCGAAAGATCTGTCTATTCTTGCGGATGGGAGAATTTTGATTTAAGCGAAATGAAAACTGTTTATATAATGATGATTCAGTCCCAGAAGTCTGTACAGATTTTAGCAGCTGATATCATGCCTATCAATGTGTATACGTTTGCTAATACACTTCAAATTATGTATAAATTTATTACCGTTTTTAGACTTTAA

## >PflaOR42

ATGAAGTTGGATTTCGATAATATTTACAAAATTTCGTTAAGACCTTTATATTGGACAGGATTACATCCATCGACACGTCGTGACATGAAGTGGGCGGTACTTTTTCTGTTACAATACGGACCGTGTTTCATTATGTTTCTTGTATTAATGTACGGCATGCAATATCAATTGTTCAATGGATTATACAGTGAAGTATGTGGCACTACCACTATAGCGATAGTCTTCGTTCTTGTATCTCTACAATATTTAATAGGGGTGGCATATCAAGATAAACTCCAATGTCTTATTGATATAATGAAAGCGGATTATGAAAGTGCCGATGATTTAGAAGACATCGAGCGAAATCTTGTTATAAAGTATGCCATGAAAGGAAGGTGGATGACTCGAATGTGGCTTGTGGTTATCGGTTTAACTGGAGGATTGTTTCCAATGAAGGCGATTCTTTTTATGCTTTATTATTGGGCAAAGGGAACATTTCGATTAGTTCCGTTATTTGACTTATTTCATCCGGATATAATGGAATATCAAATGAATAGCGGAGTTGGGATGTTTATTTTTTGGAACGTTTTCACATGGGTATATAATAATCTTTATCTTTGTACTGTTTTTATTGGGTTTGAAGCACTTGCTACTGTATTTATAATACATGCGTGCGGTCAGTTGGAGATAGTAAGTCACAGAATATACAAACTGTTTTTGGATGAAAATGACGATCAAAACCGTGTGGATCTGATCAAAATAATTACGGAACATTCGATTCGTATTTTTAGATACCTTGATATGGTGAATGAGATCTTCAGTGTACTGTATGAAATTCTTCTTAAAGCATGTGAAATCACAATACCAATAACGCTGTTTATTATACTAGAGGGTTACCGTACTGACCAAATGCGTTTAGAATACCTGAACTTTATTCTCGCCGCTGTATGTTCATGTTATGTTCCCTGTTATTATAGCAGTTTACTCAGAGAAAAGGGTGAAAACGTAAGATTGGCTCTATATCAATGCGGTTGGGAGACGCAGAAAGACCTGCGCACACGCACCTCTCTGATTATTTTGCTCACCCGTGCACTACGACCTACCACCGTCAGAACAATATTACATACGCTGGACCTGGATACTTTTGCCGATGCCTGTCACCATGCTTATGCTTTGTTTAATCTTTTAAATACAATGTGGACATAA

## >PflaOR43

ATGTACACAGAGAATGTACTGTTTGAACGAATATTGAATAAAGTAAACAAAATTTTAGCAGTAATCGGCTTGCGTTTGCAAGAGGAAGACAGCAATAGAAACTTTATGTCCCGCTTATGGAACCGTCGGTTTTATTGTATCTCCTGGTTGTGGCTGAATACAGACTATTTTGGTGAAATACTTTGGTTCCTATACCAAATTGCTGAAGGAAAAGATTTCGCTGAACTAACGTTCTTAGTACCTTGCATGGTAGTTTGTCTCCTAGGCGAATATGCAACATACTGTCTATTGAAATTTGCACATCATATCAATGAATTGATTGGAATTTTACGTAGATCACAAGCCCGAATCATTTTGGATGACAAGAAACAAAATATGATAGCGAAATCAGCTCATTTCATTGATCGTGTTGTAAATCTTCTGATTGGATTTAATATTGCAATGGTGGTAGCTTTTGCTATGGTGCCGTTGGTATTCACTGTTAAAAATTATTTCGAAAAGGGAACAGTTGAATTGCAATTGCCATTTCTAATTCTGTATCCGTTCGACCCGTATGATATAAGAGTTTGGCCAATTATTTATTTGCATCAGCTATGGTCGTGTAACATTGCATGTTTCTTTGTTTTATCGTCAAATCTGCTGTATTACGTATTCTGTACGTATATCAATATTCAATTCACCCTTTTGCAAATTGATTTTGAGACACTGAATATTGCTGACATTTCAAGCACGTCAGAGATTAAAATTAAATGTCGATTGGAATTTTTGAAGTTGGTAGAACGTCATAAAGATCTTATACGATGTGTGGAGTTGATGGAAATTGTTTATTCCAAGTCTATGTTGTTGAATATTATAATCAGTTCCCTTATAATATGTTTCACCGGCTTTAATGTTATGATTATTGATAATCCAGCCTTGATAATATCATTCCTGGTATTTCTCTCCGTCGGTTTTTCGCAAATATTTTTCTTATGCTATTACGGTGATCTGATTGTGAGTTCCAGTACGTCTGTAAGTGGTGCTATATATAACTCTACGTGGTATGGAGGCGATGCTGCGATTGGCAAAGAAATACTTTTAGTTTTGACAAGAGCACATAAGCCCTGCCTTCTTACTGCCTTTGGGTACGCCGATATCAATCTCATGACATTTACAAAGATACTGAGCACGTCGTGGTCATATTTTGCTCTACTAAATACTATGTATCAACGTTGA

## >PflaOR44

ATGAAGATTCTTCGTCTGTTCTTGATGAGTATAGGCGCTTGGCCTGGTGAGGCCTTAGGAGAGAGGCTACCAGTGAACCTATACTTACGGAAGCATGTTGTAGTTCAGACATTCGCCGCATTGTTCGGCGAAATGTATTTTTTAATCCGGAACTTTAGGATCCTACCTTTTTTCGATCTAGGACATATGTACATCACCAATTTTCTGACAGCTTTGACTTTTGCTCGAGTTTTGCTGCCTAATTTCAAAAGGTATAGGATCATATCGGATCGGTTCCTGAATAAGTTTCATTTGATACATTTCCGACACCAGGGGAGATATTACGAAGAGATGTATAATAAGATATCCAAAGTGTCATTTTATTTCGCGCTATATTTGATGTCAATAATGATGTTCGGACTGATTCTATTCAATCTCTCGCCAATGTATAACAACTACAAAATGGGTTTGTTCAGCAACAGCATACAAGACAACGCGACTTTAGAATTCTCCGTGTACTACAGTTTCCCAAGCTTCAAACCATTGAAACGTTTCTACGCATGTACTCTGTACAACTACATACTATCGTTTGTATGCGCTGTGTCTGTTTGTGGAATGGATTTATATCTATCGATGATGGTGTTCCAAGTCATTGGTCATATAGAAATATTGAGATATAATTTGGAAAATATGGAGCGACCGAAGAAAAAGCGACTCTGCATGATAAGAAATATCGGAGTAATGTTGGATATCTATGATATAAATGAGAATAAATCGGTTAATATAATGCTTAATCAATGTACGGACCATCACCGATTTATTGTAAGCGTTACAGATGAAATGTCAGATTTCTTCGGTCCCACTCTAGCTTTCTATTACCTGTTTCATCTCGTCAGTGGCTGCCTGTTACTACTTGAGTGTTCAAGGGGGGATCCTGATGCTTTGGCCCGTTATCTCCCGCTGACGATTATAATTTTCGGCCAGTTGATGCAGATCTCCATTGTGTTCGAAGTTGTGGGTCACGTTAGTGAAAAGCTAATTGATTCAGTTTACCTGGTTCCTTGGGAATGTATGAACGTTGAGAATCAAAAGAAGGTGTGCTTCATACTGAACAGAGTCCAACGTACGATACACGTCACCGCTATGGGTATTACACCTGTAGGTGTCCAGACAATGGCTGGGATATTAAAAACATCATTTTCGTATTTCATGTTTTTGAGAACGATGGACGTT

## >PflaOR45

ATGAATTTGTTTCGAAGAAAATTAAAACTGCCTGACGTAGAAGACGGCAAAATAAAACACGAAAGGTTCACAAAAACGGCAGGTACTTACAAATACACAACGTTCTTACTCGCTATTTGTTTGATGTATCCTAACCCAAGCAAGGACAAGAAGAGATTAAAATGCCTGTGTGTAGCGCTGCTAGTTTTCATGCTGCCATATGTTCTGTTCCTACATCAATCATTTTTATGCGTTATAAACAGGGATATCGGAACTTTCTGCAGACACGTAGCTATCGGCATACCGCTCCTCGTCGGCCATGGTAAGGTTTTTGTCTCGTGGGTCTACAGAAAGAGTTTCAAATCCCTTATTGATGAGGTTGACGACGATTACGAAAGCTACAACCACGCGTCTGATGAAATCAAAGAAATAGCTGTACAAACGATCAAGAAGTGTAAAACAATCGAGACCTGGTGGCCTGTTGCAGTAGGATGTTCGACCTGCATTTTTCCACTTTGGGCCGCAAAAATCATGATCTACAACACGCTGTTCACGGCAGAACCAACTAGAGTGATGATTCACGAAATGAACGTGTGGATTTGGTCGGATGAAGACAAATATAAATCACCAACTTTTGAGTTTCTGTTTTTATACATGGGTATTGGTGTGTTAATACTCTTTCTGAGTTTTTCAGCTTACGTAGGTATGTTCAGCGTCTTCATCTTCCACGCGTGTCTCAAATTCGGAATTATCTGCAATATGCTGGATCACGCTTTCGATGACGCGCATGACGTGCCTGGCATTAAGCGGAAGCTGGTCGCTATTGTTAAGGAACAATGTCGAACCTTTGAGTTTGTAGAAAGAATTCAGTATACGTACCAGGTGTGGCTAGCAGTTTTGTATCTGCTCACAGCGTGTCAGTTAGTGCTGACCTTGTACCAGTCTATCGGCACGGACAAAGGCGAAGGACTAGATTTTCGCTACTTGACGCTAATAGTGAATGCGGTTATCTACTTCTTCTTACCCTGTACCTGGTCTACAAAAGTCTCTACCATGGCAGCAAGCGTTTCTGACGCCGCTTACTGCGCGGGATGGGAACGAGTCCCCGACACCGACGTGCGTCGAACTACTGCAATCATCATCGCTAGATCACAAATTCCTTTACGAATCACCGCCTTAAATGTAATTACTTACAACATGGAGATTTTCGTATCGATGATGAATACTTCATATTCCGTCTACGCTATACTCCGCAGTTAA

## >PflaOR46

ATGAATTTGTTTCGAAGAACATTAAAACTTCCTGACGTCGAAGATGATACCGGCAAATTAAAACACGAAAGGTTCACAAAAACTGCAGATACTTACAAATACATAACGTTCTTACTCGCTATTAGTTTGATGTATCCTAACCCAAGCAAGGACAAGACGAGATTTAAATGCCTGTGTGTGGCGCTGCTGACTTTCACACCGTTATATACTCTATTCCTACATCAATCATATTTATGCGTAATAAACAGGGAATTTGGAACTTTTTGCAGACACGTATCTATCGCCATAACGATCCTCGTCGGCCATGGTAAGGTTTTTGTCTCGTGGATCTACAGAAAGAGTTTCAAATCCCTTATTGATGAGGTTAACGAAGATTACGAAAGCTACAACCACACGTCTGATGAAATCAAAGAAATAGTTTTACAAACCATCAAGAAGTGTAAAACAATCGAGACCTGGTGGCCTATTGCAGTCGCCTGTTCAACATGCATTTTTCCACTTTGGGCCGCAAAAGTCATGATCTACAACACGTTGTTCAAGGCAGAATCAACAAGAGAGATGGTTCACGAAATCCACATGATTTTTTGGTCAGATGAAGACAAATATAAATCACCAGTTTTTGAGATTATGTTTCTTTACACATGCATTGGTGCGTTAGTACTCTTTCTGACTTTTTCAGGTTCTCTAGGTATGTTCAGCGTCTTCATCTTCCACGCGTGTCTCAAATTCAGAATTATCTGCAAAATGCTGGATCACGCTTTCGATGACGCGCATGAAGTACCCGGCATTAAGCAGAAGCTGGTCGCTATTGTTAAGGAACAATGTCGAACCTTTGAGTTTGTAGAAAGAATTCAGTATACGTACCAGGTATGGCTAGCAGCTCTGTATCTGCTCACGGCGTGTCAGTTTGTGGTGACCTTCTACCAGTCCATCGGCATGGACAACGACGAAGGGCTAGATTTTCGCTACTTGTTGCTAATAGTGAATGCCATCATCTACTTTTTCTTGCCCTGTACCTGGTCTACAAAAGTCTGTACTATGGCAGCAAGCGTTTCTGACACTGCTTACTGCGCAGGATGGGAACGAGTTCCCGACACTGACGTGCGTCGAACTACTGCATTCATCATCGCTAGATCACAAATTCCTTTACGAATCACCGCCTTTAATGTAATCACTTACAACATGGAGATTTTCGTTTCAATGATGAATACTTCATATTCCGTCTACGCTATCCTCCGTAATTAA

## >PflaOR47

ATGGAGAATTGGATTGTTTTTATTAAAAATCTCACGCGAACAGTTGGTGAGAGGACCGAAATCGACGAAATCATGGCGTTCGCAATGGTTTTGCAAAGGATTCTCGGCCACCAAATACTAGACCCTAATTGGTCGTGGAGAAAATACATTTTTCATCAAATCTTTAGCGTTGTTCTCATCATTTATGTCTTTTTCGGGACTCTTGATGTTGTAAAAAGCACTGACGATCAGGACATGATAGCCGAAGCTGCTTACACTTTGGTCTTAATTGTCATGTGCCCTTTTAAATTGTTTATTTTTATTAATAACAGATTTGTATTTAGAGAGTTGTATATTATGACTAAAACGACACTTTACGAAGCGATCAGAATCAATTCTGGTGAAAAAATTAAGGAGGTTTTGAAAAACGGTCGCCAAATGTGTATTGCTATGTTCGGAATGGTTGTAATACCTGTATCAGTATATGAAATAACAACGATTTGGAATTACGTAAATGGAAGGAGGGTGTTATTATCTCAGTCAACGAACACTCTTATGCCTATGACCACGCCGTATTACGAGTTCACTTGGTTACTGCACAGCATATTCATGATAGACGTGTCTGCGATTGCTATTGTCTTGGACATGTGGTTTGCTTTGCTTATGTACTTCTTCTGTACTGCTCTCGGTAGCTCGGTGAATATTTTAAATGTCGGTACGAGACAAGATCAGGAGAGTCAGTTGTGTTACGCGGAACGTCTGCAAAGTGCCCTCCGAAGGTTTCAAGCGATGCATGTTGAGCATATCAAGTATCTTGGACTATTGAACAAAATGTATAAGTACTTGGGGCTGATACCACTGTTGAACGTGGCTTTGTCCATCTGCATCGTCTTACTAGCAATGAGCGAAGGAATAAAATGGAACTTCGCACTGCACATGTTACCGTTGCTTGCTGAGATTTTTGCTTATAGCTGGTTTGGTGAACTGCTCAAATCAAAGATGCATGAAATACGATTGGCACTTCTGAACTTCGATTGGATAAGCCTCCAGCAGAGCCATAAGAAATGCTATCAAATTATGACATTGTTCGTGTACAAAGATTTTGGAATAAAGACAGCAAACGGCAGGCAACTATGCAGGGTCACTATGTCCGAAGTGCTCAAGGGAAGTTATCAAGTTTATACGGTTTTGCAGAGTGTATAA

## >PflaOR48

ATGGACTCGCTGACGGACGACGTGTTGTCCGTGTTCGCGTTCATGTACTCGTGGAGCTGCCTCCGCCTGGACAGCGCCGCGGGCGACCAGCACCGGTCGCGCCGGCGCCTGCTGTGCGCGCTCTGCATGTGCGGCTACAGCGTCGACATGATGTTCTCGATGCATGTGTTGTATGAAACGATGGACGAATCGTCGCTCAATAGGCTGTGCATGATGGTGTCCTACAGTTTGAGTGTAGTCGTCGTCCTCGCCAACACGATATTACACTTGGTGTACGAAGACGACATCGGGGAGTTGATCCGCCGGCTGCGTCGCCTGGAGCGGCGCGTCACGGCGCCGGCGCGTCGCGAGGTGCTGGCGCGCCGGGCCCGCGCCGTGCGGCGCCTGGTCGCGGCGTACGTCGCGTTCGTCGGCTCCGGCACGCTCGTCATGACCGTGGCGCCGCTGCTCATGGTGTTAGCGAGATACATGCTCACCAATGATCTACATTTATTAAATCCGTTTTTCGTTAAATTTAATTATGACGTCAGGATTTGGCCTTGCATGTATGTGTACGGCCTGTGGTCAGGTGTCACAGCTGTGTTTACCATTATCAGTAACGATCTCTTGTACTATACTGTGTGTACATTTACTGTCGCGCAATTCGAGCTATTGAAACTCGATTTTGAGAATTTAACAATAAGGCCGCGAAACCTTCTTCGTCAGTGGAATTTTATCGATAATGATCTAAGAGTTAACACGGTTGCATTGATAAAGAGGCATCAAGAAATAATAAGGTACGGACACTGCACTTGA

## >PflaOR49

ATGGATTTATCCAAGATTTTTGGTTTTAAAAAAAGGGATAATAAATCTCCAAAACATTGTATTTACGTATTAGTTATTCGCATGGTGTGCAAAACCTACGGCTCGTGGCCTAGTGAGGAGTTCGGAGAGAAAAAAACTAATTTTCCGTTAGACAAGATTGTGTTTTTTGGTTTAGTCATGTTATTATATGGACAAATTCTTTTCGTGATCAATCACAAGCACGAGTTAAATTTCTTTGACTTGGGCCATAATTATATATCGATATTTATCAACGTCATGTGTTTGCAACGTTTCAGTATGCTGTGGACAAAGAAATACAAAAATATACTGAATGAGTATGTTACACAAGCACATTTATTTCATTATGCAGCCGGAAGAACAGGGTACGCACAAATGATTTCGGACAAAGTGCATAAATTCTTGAAGATCCTCACAGCAGCTCTAGCGTTTCAGTTATTTGCTGGTATATTTTTGTATGTCACATGGCCACACGTGAACAACTACAGAGCTGGACTGTTTGATGAAACAAATACTGCGAACATAACAATCGAATACGCATTTTACTACGACACCCCATTCGAGATACCCTACAAATTGAGATTCGCTTCAAATTATATTTGTTCGTATTTCTCCACTTGCCCTATGTTCACCACTGAAACAGCTTTGTACGTTCTCTTATCCAATTTGTGGGGTCATTTAAAAATTTTTGAACACAACATGCACAACTTTCCAAAACCCGAAAATAATTTGTGGTTCTCTGAAAGTGAGAACAAGACAGTAAGGGAATTACTGGTTGAATATATAAATCATCATAAATTATTTAATGGATTTTTCAAAAGAATGTTAGAAGCATTGGGGCCTATGATTTGTGGGTACTACTTGTTTCTTCAGTTGAGCTTATGTATACTTTTGTTGGAACTCTCTAAACTGGACTCCGCCGCTTTTGCGAAATATGGACCATTGAACGTGATACTTTTTCAACAGTTGGTCACGATATCATCTGTTTTTGAGTTTCTTGGAACTAAGCAAGAAAAATTGAGAGACCTGGTCTACAGTTTACCTTGGGAAAGTATGAATAACGAAAATAGAAGAATAGTTTATTTATTTCTTATCAATATTCAGACACCTGAAATGAAGGCTATTATTGTACCTATAGGAGTTGGAACTATGTCAGCAATTTTGAAAAATACGATATCTTACTTTGTACTGCTACGAACACTATCTAATAACAAGGAC

## >PflaOR50

ATGTGTATATACAGTTATATTCTAGTCACATTTCTAGTTATTTGTACGATATTAGGTTTAGCAGCGGAAATAAATGTGGGCGTTGAATTGTCTGTGCGGATGTCATCCCGCATGTCGCAGTTCGTGTCCACTTGTGATGTGCTGGTGGTGGTGGCCACTGCCGTCGCCGGGGTGTACGGTGCACCAAGACGCATGAGGAACATGCTCAAATTCATGACCAGCGTTGCTTCTGTAGACACGAGTATAGGTGCTCAGTATTCAGCGGTGACTGAACGGAAGCTGTGTGCGGTTCTGCTCAGCATACTTATATTCTTCACCGTATTAATTATAGACGACTTCACTTTCTACGTGATGCAGGCGAAGAAAATAGACCGGCATTGGGAGGTGATGACGAACTACATATGCTTCTACTTGTTGTGGTACGTGGTCATGATATTGGAGTTGCAGTTCGCGTTCACAGCTCTCTCGTTGCGGTTACGATTCGCTGCCGTCAACGATGCGCTCGCGCTCACCGCCAGGGATATATCTTTACCATTAGGTAAATTGAACGAGCCAACTGCCTTGAATATGTTCGCGATACGAGTTGCCCCTGTGGATGTGACGCAGCGGACCAGCAATGGGAACGCGATCCTGATGGACTCTCTGAGCGAACACACCGTTATCATAAAGAAAAACACATTTGGATCAGCTCGGCTGGCGGTGGCGGCTCCGGAGGCTATACGGCGCCTGGCTGCTCTTCATGCTGCTCTCTGTGACGCGGTGCAGCGGCTGGACGCCAGTTATGGGGCGCCACTGATCGTGGTCCTCATTTCTATGCTGCTACATCTCATCGTCACCCCCTACTTTCTCATCATTGAAATCATGGTGTCGACAAAACGAATTCACTTCCTGATACTTCAATTCTTATGGTGCGTCACACACATGCTGCGTATGTGTGTAGTAGTGGAGCCATGTCATTATACTATCCAGGAGGGCAAGAAGACCGAGCAGTTGGTATGTCATCTGATGATGTCCTGTCCGTCGAGTGGGGCGCTGCCATCGAGGTTGGAGTTATTCTCTCGCCAACTCATGTTTCGATCCGTGACTTACTCTCCTCTTGGGATGTGTGTTCTTGACCGTCCACTCATTGTTTCTGTAATAGGAGCAGTCACTACTTATCTGGTCATATTAATTCAATTTCAGAGGTATGAC

## >PflaOR51

ATGTTTTTATCGAAAGTTTTTGGTTTTAAGAAAAGAGATCCTCAGTCTCCAAATGATTGTATTTACATAGTGGTTATTCGTATGGTATGCAAGTCCTACGGCGCGTGGCCCAACAAAGAGTTCGGAGAGAAGAAAAATTGTTTTCCGCTGGACAAGATTGTCTTTTTCGCTTTATCCATGTTACTTTATGGACAAATTCTTTTCGTGATCAATCACAAGCACGAGTTAAGTTTCTTTGACTTGGGCCATAACTATATATCGATATTTATCAACGTCGTGTGTTTACAACGTTTTGGTATGATGTGGACAAAGGACTACAAACAAATACTAAAAGAGTTTCTGACACAAGTACATTTGTTTCATTATGCAACCGAAAAAACGGGATACGCAAAAATGATTTCGGACAAAGTACACAAATTTCTGGAGATTCTAATCGCAGCACTGTTATTTCAATTTTTGGGTGGTGTATTTTTGTATGTCACGTGGCCCCATTGGAATAACTACAGAGCAGGACTTTTTGATGGAACGAATACTGCGAACATAACGATTGAATATGCCGTTTACTACGAAACACCAGTTGAGATACCGTACAAATTGAGATACAGTACAAATTATCTGAGTGGGTACTTCTCCACAGCACCTTTGTTCACCACTGAAACGGCGTTGTACGTTCTGTTATCTAATATCTGGGGTCATTTAAAAATTTTTAAACACAATATGGATAACTTCTTAAAACCAGAAAACAATTTGCGTTTCTCTGAAAGTGAAAACAAGGCAGTAAAAAAATTACTGATTGAAAATATTAATCATCATAAATTATTTGACGGATTTTTTAAAAGAATGTTAAAAGCTCTCGGACCGATGATTTGTTTATACTACTTGTTTCTTCAGATGAGTTTATGTATTCTTTTATTGGAAGTTTCTAAATTGGACTCCGCAGCTTTTGCGAAATATGGACCATTAACTTCAATATTCTTTCAACAGTTGGTCACGATATCATCTATTTTTGAGTTTCTTGGAACTAAGAATGAAAACTTGAAAAACTTGGTGTACAATTTACCATGGGAATGTATGAATACCGAGAATAGAAGAATTGTTTATTTTTGGCTTATGAGTATTCAAAAACCACAACTGAAGGCTATTATTATACCTATAGGAGTTGAAACCATGTCAGCAATTTTGAAAAATACGATATCTTACTTTATATTGCTACGAACAATATCTAAAACCAAGGAT

## >PflaOR52

ATGGCGGCGCTGGAGGCCGACACTTCGTTCGAACACTCGTTAAGATTAACAAAAATAGCTTTATTCATATCGGGAATTAATATTTATCAAGAAAAGTTTAGCACTGCAGTAGAGTACGCTATGAAGTACGTAGTGTTTTATTGCACGACGCTGTGGCTGCAGACGGACGGGCTGGCCGAGCTCGCTTGGATCTGCGTCGGCATCAAACAAGGACAACCTTTGCAGGAGTACTTCAGCGTCACGCCGTGCTTCACTTACTGCTTGTTGGCTGCAGCAAAAATGTTCAACATTTACATTTATAAAGATGTACTGATAAAGACAGTTAATCAAATGCGGGAGCTGCACGCCGCCGTGCTGCGCGCGCGGGGCGAGGCGGCGCTGGACGCGGGCGGGCGCGCGCTGGTGCGCTCGGCGCTGCAGCTGCTGCGCGCGCTGGTGGCGCTGCGGTTCTACTCGGCGCTGCTGGTGGTCGCCGTGTTCTGCTTCGCGCCGCTCCTCGTCATGGCCTACCTGTACCTCGTGCACGACGACAGGCGCGTCTTGTTTCCGTTCCCTACCAAATACTTTTTTGATCCAGAGACTCCTCAACGATGGCCGCTTGTTTATATACATCAAGTTATTTCAACGTACATAGCGTTCTGCAACGTGTTCGGCCCCGACTCGCTGCTGTACGCGAGCTGCGTGTTCCTGCACATCCACTTCACGCTGCTGGGCCGCCGCCTGCGCCGCCTGCCGCTGCGCACGCTCCCGCCCCCCCAGCTCCGCGCGGCGCTGGCGGCGCTCGCTCACAGACACACACAACTCATACGCCTCGTGGACGACGTGGAGCTGCTGTTCTCCAAGTCCACGTTGTTCAACTTCATAACCAGTTCAATTCTCATATGTCTCAGCGGATTTAACGTAAAGTCTCTTACAGGAGGCGGCGAGGTGTTCGCGCAGTTCTTGTTCATGGTGATGAGCGTCGCGCAGATCGCGATCCTGTGCTACCTGGGAGACCAGCTCATGACGTCGAGCGCGCGCGTGGCGGACGCGGTGTACGGCGGCGCGTGGCTCGAGGCGGACCCGCGCACCAGGCGCGACCTGCTCTTCGTGCTGATGCGCGCCGCGCGGCCGAGTCGACTGACGGCGGCCAACTTCACCGACGTCAACCTGACCGCCTTCGCCACGATACTGAGCCGCTCGTGGTCGTACTTCGCGCTGCTCAACACTGTTAACGAATGA

## >PflaOR53

ATGAGTGGCTCAGAGCTCAATGCAACAGATCGCAATCAAAATCTTCAAACGGAAGGATTGTTTAGAAGTCCAATAAGAAGGGGGCCTGGTCCAAGGAAAAAACCATTTGCAGCTAAGCTGTGGGATTTAATACAGCCCGCGGCAAGACTATGGGGGCTCATCACTGCCGGAGTAATGAGCGGTGCTGGTGCCCAGCTTGTTGTCTTGCAGTATGAAGTCGCACCATTCGTAATAGTAGGTGCAGCGTTAGTTCTTATCTTGGAGACGATGTGGGTTGTCGCGTTGTTCGTGGACCTGATATGTCGGCGAGGGGACTACACGCTGCCTCTGAGGTGTTGGGACTTCATGAGGTGGTCCTGTGCACGGATGAGGGCTCCGCTATATGCCTGTGCCGCGACTGCTCTCATTTTCAGCGATCTCACCTACCTAACAACCATATCAGGTGGTATGTTACTGGTCCAAGCTGCCCTTAGAGCCGCTGTACCATTCTCACCTTATGCGATTCACAGTACGCATGCTCCCCGAGCTGGTAGCTCGTTGCTGAGTCAGCTGGAGTCGCCGATTCCTGACGTGTACTACAACGCACAGTCTGATGATGAAAGGTGTGAAGAAATGACAGTTATCGAAGTGAGAACTCCGGATCCGTCTAGACCCACAACGCCCAGACCACCATTACTGGAGCTGTGA

## >PflaOR54

ATGGCGCCGTTAAAGTTTGACAAGAAGCAGAAAACTCATACGATGCGCCTGTCTCGTACCTTATGCTACGTCTCCAATGTGACCATCACAATAAGCAATGTAATTCTCCTGACGGGCCTTATGATTGATTTGGGCCTGAGCGATGACAAGTCAGTGCGCGTGGGAACACCTATCAACCGTGGTTGTTGGATTGTGGATGTCGCTGTGGTGGCCATCGTCTCCTTCGCGGGATCTTACGGCGGAAGAAACAGAACTCTACATATGATACGATATTTAAATACCCTCGCAAAGATAACAGCCCAATCAAGTTCTAACGTAAAGAAGAGCAATGAAAAGAAGAAGATGGTGTTTGCGGTAGCGTCTATATGTTTTATGGCCATGCTTGTGGGCACGGACCTCTACAAGATGTACAGTTTAACCATAATGCACGATGAGGACTGGAACGTAACATGTATGTACTTGACGTTTTACTTCGAGTATTTCTTGCTGTTGCTTCTTCAGTTGGAGTTCATGATGAAGGCGCTACTCCTGCGAGAGGCGTTCCAATCCATCAACGACGAGCTGGACATTCTTCTTCTAAAATTGGCGCGACCCGATAGTCTTCGTAAGAACTTCGTTTCTAATAATCACTTCACAATTCCTAAGGCTCATCCACCTAAAGGTGCAGTGAATGCCACATTTGACACTGTAACTGTAAACAATGAAACCAAAACTTTTGATTGTACCGAAGACAACAAAGCGCCTTTAGTTATCAGTCGTCTCTCTCACACGTACGGCTCTCTTTGCGTTTTGCTGAGAGGGCTGAACGACAGCTATGGTGTCGTTAATTTGGTGTTGCTTGTGGCTATTCTTCTCCACCTAGTCATCACGCCGTATTACCTGATAGTCCACGTCATAGATACAGGCAACATACAGCTCTCTATGATGGGCCTGGTCGTACAACTGTGCTGGCTGTCATTTCATTTCACCTCAATGCTGATGGTAGTTGAGCCTTGCCATCAAACAACAGAAGAGGTGAACAAAACACAGATATTGATAGGTCAGTTGACACGGCTGTCCAGGGACGATCAGCTGTCCGCCGAGCTGGAACTGTTTTACCGCCAACTCCGCCTGGAGACCGCTAGCTACTCGCCGCTAGGGATGTGCACTCTCGCAAGACCTTTAATCACAACGGTCCTTGGAGCAGTCACAACGTACCTGGTAATCATCATACAGTTT

## >PflaOR55

ATGGCGTCATCAAACATTGAACAAGCGACACAAGAAATAAACTCATCCCTAAAGCTGAGTAAATTCTGCATGAATCGTATAGGTGTCTCTTTTGATAGACCAAAAACGACAATGGCGTATGTTGTGCAAAAAATTACGTTTTTGGTATCAATTTCGGCTATATGCTATCACGTTTTTAGTGAGGTTGCATATATTGGCTTAAAGTTATCTAATTACCCAAAGGTTGAAGAGGTGGCACCACTTCTTCATACTTTTGGTTATGGATCTTTAAGTATTGCGAAGGTTTTCACTTTGTGGTATAAGGTAGACGTATTCAACCAACTTATTAAGGAACTTGTACATATATGGCCAGTGGCCCCTCTCAGTTATGAAGCTCAAAATATAAAGAACAGAAGTTTGGCGGCCCTTCGCATAGTTCATACAGGGTACTTCTATATTAATATATCTGGGGTGTGGTTCTACAATTTGACTCCAATTGTCATGTACTTCTATAATACACTACAAGGTCGTGAAGCGGAAATTGGCTATGTGTGGGTGTCGTGGTATCCTTTTGACAAACGGAACCCGATTTGGCACGTGTTGGTTTACATCTTCGAGGTATTTGCTGGTGTAACTTGCGTATGGATCATGGTGAGTACTGATTTGCTATTCTCGGCGATGGCTAGTCACATCAGTTTACTGTTGCGCTTGCTACAACAACGCCTAGAGACTCTAGCCGGTGATGTACATAAAACGCAGGAGACGTACTACGAAGATTTAACATCAAGCATAAAACTGCATCAGCGTCTTATCAGGTATCGCGACGATTTAGAAGAAGCATTTTCATTTGTCAATCTCATTAACATTGTGATGAGTTCTGTGAACATCTGCTGCGTAGTATTCACTATAGTGCTTCTGGAGCCATTTCTGGTAGTAAGCAACAAACTGTTCCTGTTATCAGCTCTTATTCAAGTTGGCATGCTCTGCTGGTACGCTGATGATATTCTTCATGCGAACGCGGATGTTGCTTCGGCTGCGTACAACTGCGAATGGTACCGCTTGAATCCTAAGTGTCGTCGGGCATTGCTCTTCGTTATGCGAAGATCACAAAAACCTATCGCTTTCACTGCAATGAACTTCACTAACATATCTTTAGTGACGTACACTTCTATCTTGACAAGATCATATTCCTATTTTGCTCTGTTATACACCATGTATAATGACAATTAA

## >PflaOR56

ATGGAACAATTTTCCAAAAATTACACAAGGTTATTTAAATATTGTTTCTTTTTCTACAGTCGTTGGAATTTTGTATTTATAGATAAGAGAAAATTGTCATTTTTTGGAAAACACTTGTGGGCTATTATAACCACTGGAGTCTTGATTGTACAGTACATATCACTAATACTATACATAAAACAGGCTTTAAGTGGTGTTATAAATATTTATGACATGGCTTTTGTATTGTCGACAGATATGGTTCTATTCCAAGCATTATTAAAAGGTATTTTGGTTGTAAGAAAGAAACCACATATTGAGCATATTATAAATGTGTTGGGTACTATGTGGAGGGATGAAAATTTGACAGATTTGCAATTTAGTAAAAGAAATAAAATTTTGAATAAAGTTGACTTTATATTGAAAGTAAATTTCTGGTTAGACATCATAGGAATGTCGCAATACTTGCTTCTACCAATAAGCGAAGTACTCTACGGGATTTTATTTAGTCCGCATGAAGAGCTTCAATTTAAATTCTTCGTTAACGGTGTTTATCCGTTTGATGAGACTGCTAATTGGATTAATTATATCCTAGTCTATACATCTCAACTTTATGCAGTTACATACGTCGTATATCTATATCTTGGATGGAAATTCTTATTAATGATTCTGAGTGGTTTATTGAGCGCCGAATTCGTTTTACTACAAGAAGATGTAATTAACCTCAATAAAAGCAATCAATCTTTGGCTGACCCGAATGAAATCTTACAAATGAACGGACCTCGTTTCCTTGATATTAAGGAAATTGTAAAAAATCATCAAATTTTAATAAGTTTGTGTGCTGAGGCCGATGAAATATTCAATTTTGCTATATTTATGGACGGTACGCTTGCCACAGTCACGATATGCTTATTCGGATTTATTTCAAAGTTCGCTCACAGCAAAGTGGAGTTGGCAAGAACTTTGATGACGGGAAGTGCTCTTCTAATGCAGATATTCATTACGTGCTACTTTGGAGAAATGCTTACTGCAGCGAGCGCAGAAGTTGCCAACGCAGCATACAAAGTCAAATGGTACAAATCTAATAGGTATTGCCACAGGACTCTATGTCTCTTAATGCAAAGGTCTCAGCGCCCCTGCAGCCTTACGTCCTTGGGATATGTACCAATTACTATGAGATCATTCAGTACGGTTCTGAGTACCACCTGGTCTTACTTCTCTATTTTAAATACCATGTATCAAGAATAA

## >PflaOR57

ATGCTTACAAAATCTAACGTAAAAAATGTTTTCCTAAATGACATGAAATTTATACTTTCCATTGTTTCACAAATATTTCTTTTTCCATTCCTTCAAAGGTCGAAAATGAAAACCTTTGGTTTTTATCTTGTTTTATTTGTTATATTCTTGACAATAATACAGCTTGTGCTGACATTGTATACAACTGGTTTCTCCGACCTTATGGAATTTTGTAATGTAGCTCCTAACATTGGTGTTTGCGTTTTGACTTTAACCAAATATCTTAAAATACATATGAACAAAGAACTATACGACTCCATCGTGTCCTATTATCTAGAAGATATATGGACAATCTTAGACGATACATGCGTATTAACTTTGAAAACTATTTTACAGTATATATCTACTTTGAGATTCGTCACTAAAGTATTATTCAACTATTCTCGCCCACTCATTGTTATCATCAATTGCTTTCCTTTAGTAATTATGGCCTATGAATACAAAATTCGGGGTAATGATTTAAAATACTTATTTCCTTTTGATGGTTGGTATCCATTTGATAAGATCACATTTTTTGCTATCCCATATACATGGGAGTGTTTTATGACAGGATTGGTTATTTACATATATACTACAACAGAGATTTTAAATATTACTTGTGTAGCTTGCATTAGTATGCAATTGAAAATACTGAGTAATTCAATTACTGAGGTCATATCAGACGAAGATGTAACTAATCTTGCAAAAGGCATTAACTTGCATGAAACTCATAAAATTATAAGAAATAAATTGAAGACTATTATAAATCAGCATCAAGATTTACACAGATTTTCTTTTGGATTAGATCAAGCTTTAGGCGATGTTATGGTTCTTGACTACATTTTCGGGACGTTATTTATGTGTCTCACTGCTTTTACTTCAACTATGACTGATGACTTATACCAGACTCTTCGATGTTTCTTTTTTTTCTTTTCTCTAGTTATAACTGTTTCAATACATTGCATGATTGGACAAGTAATACATGACCATAGTTTACTTTTAGTTGAAGCATTGTATTCCTCCAACTGGCCTCATGCTGACAAACAAACAAAAATTATAATGATGATGTTTATGATGTACCTACAGAAGCCTTTTGAGCTTACCGCAAAAGGTTTAATCCGAATGGATATAAATGCAATGACTAAGATGTATTCTATGTCGTATCGATATTTTAACTTACTTCGCACAGTTTATGTCAAATAA

## >PflaOR58

ATGAGAAATTATTTAATATTGAAGAATGCGTGTAAGAAAATTTACTTAGCAGGCTCAGGAAACTTTTGGTTTGAAGATGTGTACGACGACAATCGTTTCAGTTATAGAATTTATAGATTGATTTTGATGTCGATATACGTAATGATGACAATTTTGGAGATTATGGCTACTTTTGACAAAAATCTTCCAGAGGAGGAAAAAAGAGATGCTGTATCATTGGGCGTAAGTCACACTATAGTAATGGTGAAGATATTCTCAGTTATTTACAATAAAGATTTAATCAAATCACTGAATGGAAAGATGATTAAGCTTTGCGAGGAATACGAAGAAGAGAAAACAATTGCTGATCAGTATAGGATTATAAAGGTCAATGTTCTTGCTTACTTTGCGGCAGTATACGGTGCCGTTGTTTCCTACATCTATATAGGAGTTAGAAAACTGCAGGAAGGATCACATTTCATCACAGTCGTGACGTATTGGCCGTTTTACGAAGATAATTCCGCATTAGCTAACGCATTTCGTTACTTGACAACGATTATTTTATGTATTTTGATGATCACAATGATAACCATTGATTGCTTTGCAATGATGTACTTAATTCTGTATAAGTATAAGATAATAACGTTAAGAAGATACTTCGGCTCATTAAAAGTGGATTTTGATCAAACTTCCCGTACCAACATCGTTTTGGCTTCTGACAGATTAATTCAAGGATTTGTTCGTGGAATTATAATGCATAGTGAAGTTTTGTCGTTATCTAAGGAAATCGACAAGGCATTTGGAACAGTATTTGCACTTCAAGTTTGCCAAAGTTCAGGTTCCGCTGTATCACTCCTCTTGCAAATTGCGCTATCGAGTCACGTGACCTTTGTCGTTGGAATCAAAATATTTTTATTTATCGCGGCAATGTTCTTCTTATTAGCATTATTTCTCTGTAATGCTGGCGAAATTACGTACCAGGCATCCTTACTGTCAGATTCGATATTTTACTGTGGTTGGAATGAATGTCCAGTGACGTCAGTTCCACGACGCAACCTGCGCCGTCTGGTGGTGATCGCGTGTGCGCAGGCGCAGCGCCCTCTGGTCATGAAGGCGTTCAAGATGCTTGAGCTCACATACTCTACCTTTCTTTTGGTAGTTCGAGGAACATATTCAGTATTTGCCTTGTTCTACGCACAACACAAACAGTAA

## >PflaOR59

ATGGAATACCTGTATTTCTGGCTATTGCAATTATTGTATATTTCGCAACCCACAAACAAGGGTTTGTACGAGCTAGTCGACTTCATGAACCTCAACTTTATGCACCACTCTGCCAGAGGCCTGAGCAATGTCACCATGAAACAGAGTTACGAAGCTGCTAAAAGATTCGCATATATTTACACAGCTTGTACTATAACCAGCGTATCCATTTATGTATTAATGCCAATCTTTATTCATATATGGACAAAGCAACCTTTGGACAATTGGGCATATATGGATGTAACACGGTTACCAATGAAAATTCTGGTGTTCCTCCGACAGTGTCTCGGACAGACTTTCGTCTGTCTTTCAATAGGGCAACTAGGTGTATTTTTCGCCGCGAACGCCATCTTGATCTGTGGCCAACTAGATTTGCTGTGCTGTAGCATTAGAAATGCTCGATACATTGCCCTATTGAAGAATGGAGTTACGCATCACGCGATCAAAATGACTCATTCGGTTGACCTGCAAAATGATGAGAGGCATCAACAAGTCGTAAACATTCCGTTTCACGAAGACTCTGTATATGATTATGACCAACATCACATTGATAAAAGTTTTACAAAAAGCACGCAATTTGATATATATGACAGAAGACATGATGATGCAACCTGCGAAGCGCTGCGTCACTGCGCTCTTATTTGCCAAACAGTAGCAGAGTATAAAGAGAGATTCGAAGACTTTGTGTCACCCTTGCTGGCGTTGAGAGTCGTTCAAGTCACAATGTACCTTTGTACGCTTTTGTACGCCGCAAGTGAAAAATTCGACATGGTCAAAGTTGAATATCTTGGAGCCGTTGCATTGGATATGTTTATTTACTGTTACTATGGAAATCAAATAATTTTACAGGCAGATCGTGTGTCAACAGCTGCATACCAAAGCTCATGGTACACAATGGGAGTGCGTCCAAGGAGACTTCTCCTCAACCTTTTGCTGGCCAACAGAAGAGAAGTCAAAGTGCGCGCTGGGAGGTTCTTAACAATGGATTTACATACATATCTTGTTATTATCAAGACCTCATTCTCTTACTACACCTTATTAGCGAATGTCAACGAGAAGTAG

## >PflaOR60

ATGAAGATTCTTCGTCTGTTCTTGATGAGTATAGGCGCTTGGCCTGGTGAGGCCTTAGGAGAGAGGCTACCAGTGAACCTATACTTACGGAAGCATGTTGTAGTTCAGACATTCGCCGCATTGTTCGGCGAAATGTATTTTTTAATCCGGAACTTTAGGATCCTACCTTTTTTCGATCTAGGACATATGTACATCACCAATTTTCTGACAGCTTTGACTTTTGCTCGAGTTTTGCTGCCTAATTTCAAAAGGTATAGGATCATATCGGATCGGTTCCTGAATAAGTTTCATTTGATACATTTCCGACACCAGGGGAGATATTACGAAGAGATGTATAATAAGATATCCAAAGTGTCATTTTATTTCGCGCTATATTTGATGTCAATAATGATGTTCGGACTGATTCTATTCAATCTCTCGCCAATGTATAACAACTACAAAATGGGTTTGTTCAGCAACAGCATACAAGACAACGCGACTTTAGAATTCTCCGTGTACTACAGTTTCCCAAGCTTCAAACCATTGAAACGTTTCTACGCATGTACTCTGTACAACTACATACTATCGTTTGTATGCGCTGTGTCTGTTTGTGGAATGGATTTATATCTATCGATGATGGTGTTCCAAGTCATTGGTCATATAGAAATATTGAGATATAATTTGGAAAATATGGAGCGACCGAAGAAAAAGCGACTCTGCATGATAAGAAATATCGGAGTAATGTTGGATATCTATGATATAAATGAGAATAAATCGGTTAATATAATGCTTAATCAATGTACGGACCATCACCGATTTATTGTAAGCGTTACAGATGAAATGTCAGATTTCTTCGGTCCCACTCTAGCTTTCTATTACCTGTTTCATCTCGTCAGTGGCTGCCTGTTACTACTTGAGTGTTCAAGGGGGGATCCTGATGCTTTGGCCCGTTATCTCCCGCTGACGATTATAATTTTCGGCCAGTTGATGCAGATCTCCATTGTGTTCGAAGTTGTGGGTCACGTTAGTGAAAAGCTAATTGATTCAGTTTACCTGGTTCCTTGGGAATGTATGAACGTTGAGAATCAAAAGAAGGTGTGCTTCATACTGAACAGAGTCCAACGTACGATACACGTCACCGCTATGGGTATTACACCTGTAGGTGTCCAGACAATGGCTGGGATATTAAAAACATCATTTTCGTATTTCATGTTTTTGAGAACGATGGACGTTTAG

## >PflaOR61

ATTTTTGGTTTTAAAAAAAGGGATAATAAATCTCCAAAACATTGTATTTACGTATTAGTTATTCGCATGGTGTGCAAAACCTACGGCTCGTGGCCTAGTGAGGAGTTCGGAGAGAAAAAAACTAATTTTCCGTTAGACAAGATTGTGTTTTTTGGTTTAGTCATGTTATTATATGGACAAATTCTTTTCGTGATCAATCACAAGCACGAGTTAAGTTTCTTTGACTTGGGCCATAATTATATATCGATATTTATCAACGTCATGTGTTTGCAACGTTTCAGTATGCTGTGGACAAAGAAATACAAAAATATACTGAATGAGTATGTTACACAAGCACATTTATTTCATTATGCAGCCGGAAGAACAGGGTACGCACAAATGATTTCGGACAAAGTGCATAAATTCTTGAAGATCCTCACAGCAGCTCTAGCGTTTCAGTTATTTGCTGGTATATTTTTGTATGTCACATGGCCACACGTGAACAACTACAGAGCTGGACTGTTTGATGAAACAAATACTGCGAACATAACAATCGAATACGCATTTTACTACGACACCCCATTCGAGATACCCTACAAATTGAGATTCGCTTCAAATTATATTTGTTCGTATTTCTCCACTTGCCCTATGTTCACCACTGAAACAGCTTTGTACGTTCTCTTATCCAATTTGTGGGGTCATTTAAAAATTTTTGAACACAACATGCACAACTTTCCAAAACCCGAAAATAATTTGTGGTTCTCTGAAAGTGAGAACAAGACAGTAAGGGAATTACTGGTTGAATATATAAATCATCATAAACTATTTAATGGATTTTTCAAAAGAATGTTAGAAGCATTGGGGCCTATGATTTGTGGGTACTACTTGTTTCTTCAGTTGAGCTTATGTATACTTTTGTTGGAACTCTCTAAACTGGACTCCGCCGCTTTTGCGAAATATGGACCATTGAACGTGATACTTTTTCAACAGTTGGTCACGATATCATCTGTTTTTGAGTTTCTTGGAACTAAGCAAGAAAAATTGAGAGACCTGGTCTACAGTTTACCTTGGGAAAGTATGAATAACGAAAATAGAAGAATAGTTTATTTATTTCTTATCAATATTCAGACACCTGAAATGAAGGCTATTATTGTACCTATAGGAGTTGGAACTATGTCAGCAATTTTGAAAAATACGATATCTTACTTTGTACTGCTACGAACACTATCTAATAACAAGGACTGA

## >PflaOR62

ATGCCGCCTTCCAATCGAGAAACAAAACAGAACGTATTAAATACGAGTTCTATGAAGATTCTTCGTCTGTTCTTGATGAGTATAGGCGCTTGGCCTGGTGAGGCCTTAGGAGAGAGGCTACCAGTGAACCTATACTTACGGAAGCATGTTGTAGTTCAGACATTCGCCGCATTGTTCGGCGAAATGTATTTTTTAATCCGGAACTTTAGGATCCTACCTTTTTTCGATCTAGGACATATGTACATCACCAATTTTCTGACAGCTTTGACTTTTGCTCGAGTTTTGCTGCCTAATTTCAAAAGGTATAGGATCATATCGGATCGGTTCCTGAATAAGTTTCATTTGATACATTTCCGACACCAGGGGAGATATTACGAAGAGATGTATAATAAGATATCCAAAGTGTCATTTTATTTCGCGCTATATTTGATGTCAATAATGATGTTCGGACTGATTCTATTCAATCTCTCGCCAATGTATAACAACTACAAAATGGGTTTGTTCAGCAACAGCATACAAGACAACGCGACTTTAGAATTCTCCGTGTACTACAGTTTCCCAGGCTTCAAACCATTGAAACGTTTCTACGCATGTACTCTGTACAACTACATACTATCGTTTGTATGCGCTGTGTCTGTTTGTGGAATGGATTTATATCTATCGATGATGGTGTTCCAAGTCATTGGTCATATAGAAATATTGAGATATAATTTGGAAAATATGGAGCGACCGAAGAAAAAGCGACTCTGCATGATAAGAAATATCGGAGTAATGTTGGATATCTATGATATAAATGAGAATAAATCGGTTAATATAATGCTTAATCAATGTACGGACCATCACCGATTTATTGTAAGCGTTACAGATGAAATGTCAGATTTCTTCGGTCCCACTCTAGCTTTCTATTACCTGTTTCATCTCGTCAGTGGCTGCCTGTTACTACTTGAGTGTTCAAGGGGGGATCCTGATGCTTTGGCCCGTTATCTCCCGCTGACGATTATAATTTTCGGCCAGTTGATGCAGATCTCCATTGTGTTCGAAGTTGTGGGTCACGTTAGTGAAAAGCTAATTGATTCAGTTTACCTGGTTCCTTGGGAATGTATGAACGTTGAGAATCAAAAGAAGGTGTGCTTCATACTGAACAGAGTCCAACGTACGATACACGTCACCGCTATGGGTATTACACCTGTAGGTGTCCAGACAATGGCTGGGATATTAAAAACATCATTTTCGTATTTCATGTTTTTGAGAACGATGGACGTTTAG

## >PflaOR63

ATGGATTTATCCAAGATTTTTGGTTTTAAAAAAAGGGATAATAAATCTCCAAAACATTGTATTTACGTATTAGTTATTCGCATGGTGTGCAAAACCTACGGCTCGTGGCCTAGTGAGGAGTTCGGAGAGAAAAAAACTAATTTTCCGTTAGACAAGATTGTGTTTTTTGGTTTAGTCATGTTATTATATGGACAAATTCTTTTCGTGATCAATCACAAGCACGAGTTAAGTTTCTTTGACTTGGGCCATAATTATATATCGATATTTATCAACGTCATGTGTTTGCAACGTTTCAGTATGCTGTGGACAAAGAAATACAAAAATATACTGAATGAGTATGTTACACAAGCACATTTATTTCATTATGCAGCCGGAAGAACAGGGTACGCACAAATGATTTCGGACAAAGTGCATAAATTCTTGAAGATCCTCACAGCAGCTCTAGCGTTTCAGTTATTTGCTGGTATATTTTTGTATGTCACATGGCCACACGTGAACAACTACAGAGCTGGACTGTTTGATGAAACAAATACTGCGAACATAACAATCGAATACGCATTTTACTACGACACCCCATTCGAGATACCCTACAAATTGAGATTCGCTTCAAATTATATTTGTTCGTATTTCTCCACTTGCCCTATGTTCACCACTGAAACAGCTTTGTACGTTCTCTTATCCAATTTGTGGGGTCATTTAAAAATTTTTGAACACAACATGCACAACTTTCCAAAACCCGAAAATAATTTGTGGTTCTCTGAAAGTGAGAACAAGACAGTAAGGGAATTACTGGTTGAATATATAAATCATCATAAACTATTTAATGGATTTTTCAAAAGAATGTTAGAAGCATTGGGGCCTATGATTTGTGGGTACTACTTGTTTCTTCAGTTGAGCTTATGTATACTTTTGTTGGAACTCTCTAAACTGGACTCCGCCGCTTTTGCGAAATATGGACCATTGAACGTGATACTTTTTCAACAGTTGGTCACGATATCATCTGTTTTTGAGTTTCTTGGAACTAAGCAAGAAAAATTGAGAGACCTGGTCTACAGTTTACCTTGGGAAAGTATGAATAACGAAAATAGAAGAATAGTTTATTTATTTCTTATCAATATTCAGACACCTGAAATGAAGGCTATTATTGTACCTATAGGAGTTGGAACTATGTCAGCAATTTTGAAAAATACGATATCTTACTTTGTACTGCTACGAACACTATCTAATAACAAGGACTGA

## >PflaOR64

GTTTATAATTCTTCAACTTTGCTTTTAATGCTGTATTTTTATATCACCCGGAAGGAAATAATTTTCAGTTTACCTTTTGCTGTCTTGGTACCATTTGATATCAACAATTGGTATAAATGGATTGTAGTGTATTTACATTGTAGTGTTAGTGGATTTATTTGTGTTTTATATATCTCGACTGTAGACGCTCTATATTGTATTCTCTCATGCTGTGTCTGTTTCAATATGATGATGATTAAACACGACATACTGCAGCTCAACTTCAATGATGATAACATTACGCAAAATGTCAGGGAGATTGTTAAAAAGCATCAATATGTTTTAAAGTTGGCGCAAGATTTGGAAGATATCTTCGCAGCACCTAACTTGTGCAATGTCCTGGTGGGCTCCGTTGAAATTTGCATCATCGGATTCAATTTAACCTTTGGGGACTACTCCACAGTACCAGGTTCAATTCTATTCCTACTATCAGTCATTGTGCAAATTTTGATGATGAGCGTTTTCGGAGAAAATATTATTACGGAGAGCACGAATATTGGACAAGCTGCTTATATGTGTGACTGGTACAAAATGGATGCACCGACGAAAAAGATGATGTTACTTATTCAGTTGCGAGCTAGCATACCACAAACACTGACAGCCTACAAGTTCTCAATCATATCATATGCCAGTTTTTCTAAGATAATGAGTACTTCATGGACCTATTTCACTTTACTTCGAACCGTTTATAACTCCGATGGAACAACTAGCAACAAACAATAA

## > PflaIR8a

ATGGAATGGTATATATTGCTTTTGATAATTTTTATTTTGAAGTTTGTGTGCGTCGTATCCGAGTTAAGTTTGCGTTTTGTCTTCATTGTAGAAGTCCATGATCAGGATTTGGGTCAACAGATAGGTCGGGCTTTGAAGAATGTGGAGCAGTCTACAGGAGCTCGAGTGAGTGATGGCCTCGTTTCTTTGGATAGAGATAACGAGGAAGAAAGCTATAGAAAATTATGCTCATCAGTTTCTAAAGGTGTATCAGCCATAATTGACTTGTCTTGGTCTCCGTGGGGGATGGCGGAAGAAATGTGCCAATCGACAGGAATACTATTTCTTCACACAGCACTCGGGTCCCAGCAGTTAGTGAGAGCTTTGGATGATTATCTGGAGACAAGAAATGCGTCTGATCAGGCCCTTCTCTTGGAGACCGAGAGTGATGTCGACAGAGCCTTATACGAATTGCTTGGTTCCTCCAATATCCGTATTTGGACTCACAGCGGTTTCACGCGAGACGCCGCAAGGATTTTAAAGAACATGCGTCCGGAACCCAGCTACCACAGTGTTGTAGGCGGTAGGGATTTCGTCATGGAGACTTATAGAAGGGCTGTGAAAGAGAAGCTAGTTCGTCGCAACTACCGATGGAACCTGGTCCTCACTGATTATTCAGGCCCTATTTTGGATGTGGCACAGTTTATTTTGCCAACAATTATACTGCACGCCGATCCTGAAGAGTGTTGTAGGTTGATGGGTCAGAAAGATGAATGCAATTGTCCACAAGAGTTCCAGAGAACACAATATATTTTAAAAGCTCTGCTGAACTATGTCACTGAAGTGTACTTCAAACTTGATGATTCTTTGCAATTGTCTGCAAGAGTTGACTGCGAAGCAATGGTGCAGAGTCTGAACAACACAAAGGAGATATTACTGAAGCAGTTCCATGAAGATTCGGTGAACAATGAAACCCTTTTCTACTGGAATGGAGATAAGTCCAGTCTGTTTCTCCGTTCCCGCTTTATTCTTTCAACCTTCACTGTTGAAGAGGGGCTACAAACAGTTGCAAGGTGGTCGGCAAACGAGGACTTTCACCTTTTACCTGGTATTGTGTTGAAACCATTGCGGCAATTCTTCAGAATTGGAACTGCACCGGCCATACCTTGGACAATGCCTATTGTAGATCCTGTTTCTGGAGAAACCATGTTAAACGATGATCAGCAACCTTTGTATGAAGGTTATTGTATCGATCTCATCGAAAAGTTAGCTGCGGAAATGGAATTCGAGTACGAAATAATTACGCCAAAGTTCGGCACTTTCGGCAAAAAACTTCCGAATGGCTCGTGGGATGGCGTAATCGGCGATCTTATGAAAGGCGAAATCGACATAGCAATCGCAGCGCTCACTATGACGGCTGAAAGAGAAGAAGTCATAGATTTCGTAGCACCATACTTCGATCAAACTGGGATAACTATTGTCATCCGCAAACCAATTCGTAAGACCTCCCTCTTCAAGTTCATGACGGTGCTCAGAACAGAAGTGTGGCTGAGTATTGTCGCCGCCCTGATACTGACTGGCTTCATGATTTGGCTACTGGACAAATATTCACCTTATTCAGCAAGAAATAATCCTGAAGCTTATCCTTACCCTTGCAGGGAATTTACCCTGAAAGAGAGTTTCTGGTTTGCGCTAACATCATTCACACCGCAAGGTGGCGGGGAAGCCCCTAAAGCTTTATCGGGCCGGACGCTGGTAGCAGCCTACTGGTTATTCGTCGTATTGATGCTGGCCACATTCACAGCGAACTTAGCCGCTTTTCTGACTGTAGAGAGGATGCAGACGCCGGTGTCCTCGCTGGAACAGCTCGCACGTCAGTCCAGGATCAACTACACCGTAGTGGAAGCCTCATCCATCCATCAGTACTTCATAAACATGAAATTTGCTGAAGACACCCTTTACAGGGTCTGGAAAGAGATAACCTTGAACGCAACATCAGATCAAGCGCAATACAGAGTGTGGGATTACCCCATTCGTGAGCAGTACGGTCATATACTTCTCGCTATAAACGCTTCAGGTCCCGTACCAGACGCTCAGACCGGGTTTCGTCAAGTGAACGAGCATGTTGATGCCGATTTCGCGTTCATACACGATTCTGCTGAGATAAAATACGAAGTGACAAGGAACTGCAACTTGACTGAAGTTGGAGACGTGTTCGCTGAACAGCCGTACTCTATAGCGGTGCAGCAAGGCAGCAGACTACAGGAAGACATCACTAGGGCCCTTTTGGACTTGCAGAAGGAGAGGTTCTTCGAACAAATCAATTCTAAATACTGGAATGAATCAATGAGACTGTCATGCCCTGATGCCGACGAGTCTGAAGGCATCACTTTGGAGAGTCTGGGTGGAGTGTTTATAGCAACGCTTTTCGGCTTAGGTCTCTCCATGGTAACGCTGGCTTGGGAGGTGTTCTACTATAAACGTAAAGCCAAGAATAAAGTACAGATTCTAGACAGCGTGGAAAAAGCAAAACCGTCCGGGGCGCAAAAGAAGAATTTAGAAAAAATGGCGGACGGTGTTGCCAAGCTGCGAAAGAGGAACAAAAAGAACGCTGGCAAAGCTGTGAAGAGTGTTACCATCGGTGATAGTTTTAAGCCTGCTGTCGGCAAAAGCAATGTTTCCTTTATAAGCGTCTACCCAAAGCAGGCTTACCAACCGTAA

## > PflaIR85a

ATGTTGAAAATATTTTATTTATTCTTAATAATCCAAAATTCCTGCTGCTTCAACGATAGTAGCATTTTGTTGCGTGAGGTGATAGCGTATCGAAATGAAACAGGATTAAAAGCTGAATTTGCATCAGAAGTGGTAAATACAATCTACGGTGCCTTCCGTCAATGGTTCTTTACGATAATCTTCTGTGATTTTACGTATTTCGAAAACAGAGTTCTCAAATATACCGAAACTAATGATAATGGATACCCGGTTTTACTTCTCGATGGATGTCCAGCTAAGAACAGCACCAGAAACAAACCGAGGATTGATGTACATGGGACAACAGCATATGTAATAACATCCGATGAGCTTACACTTAAAGCAAACGAATTCAGTCTTCAAGCTCTGTATAGATCGGGCGTATTTAAACCGAGGAGTACCGTCATATTCGTTATAAATGTTCCTGTTGAAATCGACAAGTATTTCTATTTTCAAATGACAACTCATTTTGAGCTTCTTTGGAGTCACAGGATAGCGAACTCAGTTCTTATACTGTGGTCAAAGAGGCTAAGAATGTATTCCTATAATCCATTTTTACAAACCGTCAAAGAAATAACGGAAGTGAAAGATGTATCAAAAGAATTAGGGGACCAAATGAAAAACTTTTACGGCTACGAATTGAGATTAAGCGTTTTCAGAAAGAAATACTTGTCTGATGAGACTGGACCGTTGTCGTGTAATTCTGGCTTGGCTCACATTGTGATGCAATATCTTAATGCATCTTGCAAACCTCTACCACCAAGAGACAAGAAAACTATTGGGGACGTGCTATCTAATGGTTCATTTACAGGCCTAACTGCGGATTTAGTTGACGGTTACACGGATATGGAACTAGAGTCTCGAATTCTCAAGGATACATATTATGGTTATATTGATACAACCTATCCGCTAATACAAGATAAATTGTGCTTTTTGGTCAAAAAATCTGGAAAACAATCCGCATTTACAACCGTCGTGAATCTTATCACAGTCAAAATACTTGTAGTATTACTAGCTGATGTGATTTTACTGATTGCCTTGTCTGTATTTGTTCGGTATGTCGAACATACGTTACGACAGATGGAAGATCATCGTAACTTAGGAGAAAATGTTATAGATCTTTTGAAGTGTTTCTTAAAACAGACAGTAAATATACACTTTTCGGGACCTGGATTTAGAATAATAGTTTTGTTTATATTATTGTATTCATTAGTAGTGAGTAGCGCAATCGACGGTATTTTAGCTTCGACAATAACCTACCCACGTTATCAGCCAGATATAAACACTATAAAGGATCTAAGCCAAGCAAATTTAACAATCGCCATACATGACAGAAATATGGATATCTTTAACAAAACATTGTATTCTGATTACGGATTTTTATTGAAAAATCGGATTGAAATGGTCAATGATATCAAGTTGAAAGAAATTTTAGACAGAAGACAATTCCAATACGCAATATTGTTGCGTAAATCTGAATTATATGCTATAAGCAGAAAAATGTCTAACTTTGAGAACGGAAGGCCAATATATCATACAGTGTCAGAGTGTCCATTGCCGTGTTGGATAGTATATTCTCAACGATATGGGAGCCCATATTTAGTAAAAATGAATCATATTTTACACCATTTACACCAAGGTGGTATAACACAACACTGGACTGATGAATCTCGCCGCTCCAAGAAAAATTCAAAGCTACTTTACGTTCCCGATCATAGGAGTCAAAGGCCGCTGCCGATAAAAAATCTTAAGGAAGTTTTCATTCTGTATTGTCTTGGTATACTCATCAGTATTATTGTATTTCTTGGCGAACTGGTTGTCCATAGTTCAAAGAATTTATTTATAGTTTAA

## > PflaIR60a

ATGTCATACGTGAAGGTATTGATTAAATATTTTTCATTTATTATTTTAATCAAAGGGTCAGGATGCGTTGTAAATCCTAACGGACCAACTGTAGTTAATGATTTTACAGAATGTATACATCAAGTAATTGAAAATGATTTCATAGAACCTGGATTGCTTTTCTTTGCGAACACAAATAAATGTAGCACATCTGTGAGCAGAATAAGGTCGGAACTATTAAAAAAAGTAAACAGTCACCTAAAGTATTCTGTTCAAATTATAAATCCAGAAGACGAAGAACTCCTTTGTGATGACAAAGATAATTCCATAACCAGAGTTTTGCATCGTGATCAATTCACAGCAGTCCCAATTGCAGATTATTACATCATCATAGTTGATACCTACAGAGATTTTAATAATGTTGGAAGTAAGCTCATCAGAAATCGTAATTGGAATCCACATGCGAAATTTATAGTACTTTTATTCGGTTTAATATATGACGATACAGAGAAACTTGAATACGTTGAAAATTTATTAAATTGTCTGTTCAGGAACAACGTCATCAATGTTATTGTAGTAGTTCCCCACACAAGTTTTATACGTAAAGCTATAATTTACAGTTGGAGGCCATATGATCCACCGCAATATTGTGGTTATAAAAATGAAACTGCTGAGAACAGATTGATAGTTGTAAACGAATGCAACAAAGGTGTTTTAAAATATGATAGACAAATAATAAAAAATATTCATCCAACAAATATGGAAGGCTGCACCATAAGCTTTTATGGTATAGAAAGACTACCATTCGTAACGAAAATCGGAAATAGCGCAAACATTGAAATATTGTTTATTGAAGAAGTACTAAAAACGTTGAATTTAAAATTTCACTACACAATAAGTTCGAACTCATCTCGAGGTGAAAGACTTGATAACATATGGAATGGTGGGCTAAATAAAATCGTGTTAAAGGAGTACGATGTGTTATTTGGTGGAATTTTTCCCGACTTTGATGTCCATGAGGATTTCGAATGCAGCTCTACATATTTAACAAACTCTTATACGTGGGTAGTTCCTCGAGCATTGCATCTACCACGTTGGGCCGCTTTCACGAAAGTATTTCAGAGAACGGTGTGGTATTCCGTTGTTTGTGGATTTTTGATTTGTGTTCTGACTTGGAAAATATTTGGCATCCCAAACGAAGACTCAAGTTACTATAAAAGTTGGTCACATTGTTTTATTAATTCATTGATTTGTTCTTTAGGATTCACAACATACGTCTTTCCAAAAAGGGATAATTTACGTGTATTTTTCATATTTTTCAACATTTACTGTGTAATACTTGTGACTGCGTATCAAACGCAATTATTTTACTTTTTGACAAATCCAGCCTTTGAGGATGATCAATTAGATACTATTGAAGAATTGGTAAACAGTGGCCTTAAATTTGGCGGTTACGAAGAACTGCACGATATTTTTTTCAATTCAAGTGATGCGTTTGATAATTATATAGGAGAAAAATGGGTGGATGTATATAATATGTCAAAAGCAATGTTGGATGTGGCAGTTCATAGGAATTTTTCTATTTTATGTAGTCGTTTAGAATTAGCATATATTTCAGCTTCAATACCAGAACTAAGTGACAATTTTGGAAATTACAAATATTTTGCCTTTGTGAGTAACGTATTCACTGTTCCAATTGAAATGGTTTCGATTAGAGGATTTATGTTCATGAATAAATTTTCTTACACAATCGGCATATTAAAGCAAAGTGGGGTTACTGTAAGAATAAAAACTTATTATGCAGATTTCACTAAAAGAAAGAGGGGGCATTCGTTGAGAAAGCTTGACAGTGAACGTTCTGAGTTTGAACCTTTGTCGTGTTACCATCTCCAGGGTGGTTTCCTAGTTTTCGCTTTTGGTATTTTAAGCGGCGTCATAACGTTTTTTAACGAAATGTTATCAGAAATTAACTATATACAAAAGAAGTATCTCAAATTATCAAAATATTTTTTATCATTTAGACGTTGA

## > PflaIR25a

ATGTTTTTTGTACAAGCGTTTCACGTCACATTTATTGTTTTTCTTTCTCTCGCACCAGTTACAATAAGCCAAACAACGCAAAATATAAATGTGTTGTTAATCAACGAAGAGAACAATGCATTAGCAGAAAAGTCTTTCGAAGTTGCTAAGGAGTACGTGAGACGTAATCCTAGTTTGGGCCTGGCTGTAGATCCAGTTATTGTTGTGGGCAATCGATCTGATGCTAAAGCCTTTCTTGAAAGTGTTTGCAGAAAGTACGACGACATGTTATCAGCAAAGAAGACTCCACATGTCGTCTTAGATTTCACAATGACCGGAGTAGGCGCTGAGACCATAAAGTCGTTCACAGCAGCTTTGGCCTTGCCCACTATTTCTGGATCTTTCGGACAGGCGGGCGATTTGCGACAATGGCGCAATCTAGACGGGAACCAAACCAAATTTCTCTTACAAGTAATGCCACCTGCCGATTTATTGCCTGAAGCTGTTAGGGCAATTGTTACCAAACAGGATATTACTAATGCTGCTATAATATTTGATGAATATTTTGTAATGGATCACAAATACAAATCGCTCTTGCAAAACATACCGACGCGTCATGTCATAACACCGGTGAAGAGTTATAACAAGGACGAAATAAAAACGCAACTTAGAAGTCTACGCGAATTGGACATAGTAAATTTTTTCGTGATAGGTAATCTGAGAACTATAAAGAACGTTCTAGATGCCGCCGACGAAAACCAGTATTTTGGACGAAAAACAGCTTGGTTCGCCCTGTCATTGGATAAAGGTGACATTAGCTGTGGATGCAAGGACGCAACAATAATTTATATGAGACCAACGCCCGAATCAAAAAGCAGAGATCGTTTGGGGAAAATAAAAACGACGTACAGCATGAACGGGGAACCGGAGATTACGTCAGCATTTTACTTCGACCTTTCTCTTAGAACATTTTTAGCCATAAAAGCGTTACTGGATTCTGGAAAATGGCCAAACGATATGAAATATATAACATGTGATGATTATGACGGTAAGAATACTCCTAGCAGAGGCTTGGACCTTAAATCTGCATTTCAAGAGGTGAAAGAAACTCCAACATATGCCCCCTTTTACATTCCCGAAGATGACGCAATGAATGGAAAGAGTTACATGGAGTTCAATACTGAGTTGGCTGTGGTAACCGTCAAAGATGGAGCTTCAATAGGCAGCCGTTCATTAGGTTCTTGGAAAGCGGGCTTATCTAATCCGTTGTCCTTGACAGACCCAGAGAACATGAGCGATTATTCCGCGCAGTTGGTATACAGAGTTGTGACCGTTGAGCAAAGCCCTTTCATTATTAGAGACGACAGTGCTCCGAAGGGTTTTAAAGGATACTGTATAGATCTAATAGAAGAAATACGGCAAATAGTGAAATTTGATTACGAAATAACTTTATCGCCGGACGGTAACTACGGCACTATGGATGAAAATGGCAATTGGAATGGAATTATAAAGGAATTGATTGAGAAAAGAGCAGATATCGGCCTCTCATCGATGTCGGTAATGGCTGAAAGAGAGAACGTTATCGATTTCACAGTACCCTATTACGATTTGGTAGGAATAACAATTTTGATGAAATTGCCCAGAACACCTACGTCCCTCTTTAAATTCTTAACAGTATTGGAGAATGATGTCTGGTTATCTATATTGGCTGCTTATTTCTTTACTAGTTTTTTAATGTGGGTCTTCGACAAATGGAGTCCGTATAGTTATCAAAATAATCGTGAGAAATACAAGGATGATGAGGAGAAAAGGGAGTTTTCCCTCAAAGAGTGCCTTTGGTTCTGTATGACTTCTCTCACGCCACAAGGTGGGGGTGAAGCACCTAAAAACCTTTCTGGGCGTCTACTTGCTGCGACATGGTGGCTATTTGGATTCATCATCATAGCATCGTATACTGCAAATTTGGCGGCTTTCCTCACAGTTTCACGACTTGACACACCAATTGAGTCGTTAGATGACCTCTCCAAACAGTACAAAATTCAATATGCACCACTTAATGGCTCTGCAGAGATGACTTATTTCCAGAGGATGGCTGATATTGAAGTTCGATTTTACGAGATATGGAAAGAAATGAGCCTTAATGATAGTCTATCTGACGTAGAACGTGCTAAACTAGCGGTTTGGGACTATCCAGTTAGCGACAAATATAGTAAAATGTGGCAAGCTATGAAGGAAGCGGGCCTTCCAAACACAGTCGAGGAGGCGGTGCAAAGAGTGAGAGACTCCAAAACTTCGAGTGAAGGATTTGCGTGGCTGGGTGACGCGACTGATGTAAGGTACAGCGTGCTGACTAGTTGTGACCTTCAAATGGTTGGAGATGAATTTTCACGGAAACCTTATGCTATCGCCGTTCAACAAGGATCTCCGTTACGTGACCAATTTAACAACGCGATACTCCAACTCCTCAACAAACGGAAACTGGAAAAGCTCAAAGAAAACTGGTGGATCGGTAATCCTGAGGCCAAGAAGTGTGAGAAACAAGACGATCAATCCGATGGTATCTCAATCCAGAATATTGGTGGCGTATTTATTGTAATCTTCATGGGTATAGGACTTGCGTGTATCACCCTAGGTGTCGAATACTGGTGGTACAAATGGAGAAAACGATCTGTTATTGGAGACATTACTCAGGTCGAGCCAGCGAAATCATCTAGAAAGAACGAGGACCAGTTTGGAAATTCCAAACTGAACGAAGGGTTCACGTTCAGATCCAGAAATCTGGGCCTATCTAGTTTTAAACCAAAGTTTTAA

## > PflaIR76b

ATGGCTGAGGGGATGGAACTCATGATTTCCACAATATGTAACGTAACATCAGTTTGTGAAGATGTTTCTGATTATAAAGAGCAGCAAGTATCGCTTAAAAATATACAACGTTCGGCGTTGGCAGCGGAACTAAATGGTAAACATCTCCGCATTGCAACATACAATAATTTCCCCCTAAGTTGGGTGGAATCCTTCGATAATGGCACATTGGTGGGACAAGGACTCGCGTTCTCTATCATGGACATCTTGAGGCAGAAGTTCAATTTCACATTCGACGTGGTTGTACCAGGAATGAACTACGAGATCCTAGGCGACAAACCGGACGACTCTGTTATTGGACTTGTCAACTCAACTAAAGTTGATATGGCAGCAGCATTCCTTCCAAAAATCAACCATCTAGAAAAAATGGTGTCTTTTTCTGCCACCCTTGACGAGGGCACTTGGATGATGATGTTGAAGAGACCGAAGGAGTCAGCGGCTGGTTCCGGACTGTTGGCGCCATTTGAAACGCACGTATGGTATCTTATTTTAGCGGCAGTGTTTTCCTACGGACCTTGCATTACTCTTCTCACCCACATCCGATCCCGGATTGTAAGTGACCACGAGAAGAAGATTTCATTATCGCCGAGCTGTTGGTTCGTTTACGGGGCCTTCATTAAACAGGGGACCACGCTCTCGCCTGAAGCAAATACGACTCGCATTCTCTTCGCGACTTGGTGGATGTTCATCATACTGCTATCGGCGTTCTACACAGCAAACTTGACTGCTTTTCTTACTCTATCTAAGTTCACCTTAGATATAGAAACTCCTAAGGATCTCCTGAAGAAGAATTATCGTTGGGTGTCACCTGCTGGCGGATCAGTGCAGTACATTGTGCGCGATTCTGATGAAGTTCTGTACTACTTAAATCCAATGATAGAAAGAGGGCGTGCGATATTCCGGTCTTATCAAGAGGCCGTAGACTATTTGCCGCTCGTGAAGGGAGGTGCAGTGCTGGTGAAGGAACAGACGGCTATAGACCATTTAATGTATAACGACTACGTGGCCAAAGCCAGGGCCGGAGTGGAGGAATCAGACAGGTGTACCTACGTGGTTGCTCCGAACCCGTTCATGCGCAAGCTAAGAGCATTCGCTTTCCCAACCAATTCAAGCTTGAAGACCATTTTTGATCCTGTGTTAACTAGCCTAGTGCAAGCAGGTATTGTGGACTTCCTAAAATTCTATGAGCTGCCCAGTACGAAGATATGTCCTTTGGACTTGCAGTCAAAGGACCGAAAGTTGCGCAACAGTGACCTCATGTTGACCTACATGATAATGGCAGTAGGTCTTGCCGCTGCTTTCTCCGTGTTCATTGGAGAGCTAACACTGAGACGATTAGTCACAAACAAGAAGTTGGAATTGGATCGTCATGATGCTACTGGTGGACGCTCTAAGAAAACCAAATTCGGCAATGGGCACCATTCTAAAGTTGGCTACGACAACAATCAACCTCCGCCGTATGAGTCTCTCTTTGGAAAGAGTACGGGCAGACGCAACGCGAAAAGGAAGATGATCAATGGCAGAGATTACTGGGTTTTTAATCTAGACAACGGCGACACTAGACTTGTACCTGTTAGGACACCATCTGCTTTTCTGTACCACATTGACAAGTAG

## > PflaIR75p

ATGAAGTTGGAAGCCATTGTTTTAGCTCTCATTGCAAGTTACACCTTGGTTGTATGTATGGATGAAGGTATTCTAAATAAGGTGGTTTCATTTGTAATGAATGAAGACAGGCCCACATCGATGCTGGCTCCGAGCTTGTGCTGGAGCAAGTATGATGTTCGTGAATTAAGCAAAGCTTTGTTTAAAATGGGAGTGAACATGGCAATGTCCGACCAACCGAACCGGCCTGAACATCGTTTGCAGCACATTGTGATCCTGGCTGATCTCTCTTGTCGAAGGATTCGAGAATTCCTTCAAAATAGCAGTAAGAAGGGAGTACTGAAGGCTCCATACCGGTGGCTCATGCTCGCAGCCAACGATTCCTGGCCGCAAGACGGCATCATCAGTGAGCTTGACCTCCCAATAGACAGCCAAGTGGTGATCACGCACGACATCGGTGACGGACTAGCGCTATTTACTGAAGTATACAAAATTGTGGAGTACTCGGAAACTGTTTACGCTCCATACTCGGTATGGCGATCGGCGGGACCTTCGTATCGATACATTAACGTGAATGACTCAAGTCCATTGTCTTACGACAGTAAAGATATGATAATTACGAGTAAAACCGGTGCAATGCAAGATCTCAAGACTTCAATTTCAGTAGCGACCAGAAGAAGGGACTTTAGGAAGTATCCAATCACCATGGTGAATGTTATCGCTGACAGCGAGGACACGAAGAAGCACATGGGTGACAGGCTATTCCTACACCACGATTCTATCACCAAGATGTCGTACATGGTGGTGCACATATGCATCGAGATGTTGAACGCGACTGAGAAGCTTCTGTACACGGCCACTTGGGGCTACAGACAGAAAAACGGGTCATGGACCGGAATGATCGAACACTTCGTCAATAAAGAGGCGGATATAGGTACTTTGGCAATCTTCACGATCGAGCGCACGCACTTCATCGAATACATCGCGATGGTCGGCACCACCGGCGTCAGGTTCGTGTTCCGGGAGCCGCCGCTGGCGTACGTGTCCAACATCTTCAAGCTGCCCTTCACGGGCGCCGTGTGGTTGGCGATACTCATCTGCGTTCTTGGCTGCTCCGTGTTCCTGTATATAACTTCCAAATGGGAAACCACAATGAGTACGCATCCTATCCAGTTGGACGGCTCATGGGCTGATGTCTTGATACTGATAATTGGCGCGGTCTTGCAGCAGGGATGTACTTTGGAGCCGAGATTTATTGCAGGACGCTGCGTGACGCTGCTGCTGTTCATCTCGCTGACCATCCTGTACGCAGCGTATTCCGCCAACATAGTGGTGCTACTGAGAGCTCCCAGCTCCTCCGTGAGGACTTTGCAAGACCTGCTCAATTCTCCCCTAAAGTTGGGCGCCAGTGACTTCGAATACAATAGATATTTTTTCAGAAAATTAAATGAACCTGTTAGAAAAGCTATTTACGAGAAGAAAATAGCACCGAAAGGTAAGAAGCCGAACTTCTTCAGCATGGAGGAAGGCGTTGCTAAGGTCAGACAGGGTCTATTCGCATTCCATATGGAGTTAAATCCCGGCTACAGACTGATACAGGAAACATACCACGAAAGTGAGAAGTGTGATCTGGTTGAGATCGACTACATTAATGAGATGGATCCTTGGGTGCCGGGTCCGAAACGAACGCCTTTGAAAGATCTTTTCAGAATCAATCTCCTGAAGATCCGCGAGAGCGGTATCCAGGCGTGCGTTCATCGCCGTGTGACCGTTCCCAAGCCTCGGTGCATGGGGCTCGTCAGCACCTTCAGCAGCGTGGGCATGACCGACATGTACCCTGTGGTTCTCACCACGCTGTATGGTATGCTTCTCGCTCCAGCCGTGCTCATACTTGAGATTTTATACAAGAGAAAAATTAGCCAGCTACGAATTTGTGAAGGAGTTTTGAATAAACTTCACAGTAACGAGATCCTTAATTGA

## > PflaIR2

ATGTTGGTTAATGCGATAGCCGAGTATTTCAAATATAAACTAGTTGGTAGTATTGTTATATTGACGTGCTGGCCGGATATAGATAAAATAAAGCTAATTCATCAATTCTCACAATGCGGCATGAGTGCTACTGTGTCGTCCAATGTGAGTTTACTCAACGACATCAGAGAACACAGTTTCCAAGGCATACTTTACTGGAATGAATGCAAAAATCTGGATCAAAACGCTTATCAGATAAATAAAAAATATTTTTCGCATTGGTATAAGTGGCTTGTAGTTGGCAACAATATCCCTAGGTTCTTCAACAACACTAGATATGATGTCGATGCTTCTCTTATAAGCATTGGAGTTGAAGATGTTAAGACTTCATCAGAAAATAATACCCATAACTATTCCGCCTATGAAAAATTTGAAACTGTATACGTCCATCCCGAATTAGGTGCGTCACGGTATCCTTGGGCATTTTGGAGTCCAATAAATGGATTACAACTGACGCATGAACAAGAAAGACTTTCACGCATTTATGATTTGAAGAAATATCCACTAAGAGTGGCAACTCCGGTGGGACTTTACAATGAACACTATAAAGGAACATTTCTGGATTACCTGACAGATAACACAGATTTAGGCAGAGATCCGAACATCCGCTGCAGTTATGAAACAGCTAATTTACTGTTGGAGAGAGTCAATGCTAGTAAGATTGTTGTAAAAACAGCACTATGGGGTGCCGAGACACATAACGACAGTATGGTGAGGAAATTGTTCGACGGACGGTCGGATTTGAGCGGTGGTTTCTTAAGACCTTTAAAACATCGATTGAAACGATTAGATTACATTTTTGCAGTGTGGCCATTTGGGGTGGGTTTCACTTATTTGGGTGAACGTAAAAGCAGTAGCAATATGTTTCTGGAGCCATTTTCAATAAGTGTGTGGTGGTGCGTTTTCATAATATCTATGATTTTGGCGGTGGCTCAAAGAGTGACAGCCAGGTCTGAAATGGAGAAAAATGGAGCGTTTATTGACGTTATAGCGACGTGGTTGCAGCAAGATTCGGGTGCAGTACCAACGGGAATATCTGGCCGATGGACGTTTACAGTGATGTCGATATCAGCTATGCTGGTCCACGCGTACTATACGTCTGCGATAGTCTCCGCACTAATGAGTACAGGCATCGGGGGACCGGACTCGCTGAGATCATTAGCCGACTCTAAATATGCGATTGCCTCTGAAGACTATAACTATATTCGTTTGTTGATGTTTGACGTTATGACTAACGATAGTGATTTGGAATACCTGAAGAAGAAAAAGATGCATCCGAATTTCTACATAGATATAGAGAAGGGCGTAGAACTTATAAAAGGAGGCAATACTGCTTATCATACCGAATATAACAATATATATCATCATTTGAGACGATTTGACAACGAACATGTGTGCAAACTGTCCTATGTAGACACTTCTCCTGAGATGCCTATCTACACAACCATGGGGTACAGAAGTCAATATACTGATATATTCAGATCTTCAGCAAACTGGCTCCGTGAAACAGGAATGGGAAAACATCTGGTCTCCAGATGGCGAGCACTACCTCCTCCGTGCACGTCATCAATGCTGGCTGATAGAGTGACATTCTCTGATATCGTACCTCTGTTAGTTCTTACTATAGTAGGAGGAGTATTAGCGTTCAGTATACTTGTGCTAGAAATATTTGTTTCAAGAATAAAGAAAAATATGAAACCTCTTGATCGTGCTGTTAGCAGTGTTTCTTCAAATATAACTTATATACATGAATATGTAGATTAA

## > PflaIR75q

ATGTATGCGAGGTGGATTATTACATATTTATTAATTTATACAGAATATGTTAGAACGGATACTAGTATTCTTCGTGAAAATATTGCAGTAAATTTCATAAAAGATTTTTTGACGCGAGATGTGACATTGTCAAAATATTTTATTGTCACGCATTTGGATATATCAAAAAAACAATCCATTAATTTATGTAAACAAGTACAGCTTGTAAATAGATACTGTGCGGTAAAAACTATGAGTACGAAGGGCCGTAATCAGGGTGCTCTACATTTGGCTAACCTACACAACAATTCTACAAAACTAATGCTGAATGAGCAATCCTGCAATATGTCCAGTAGGAGCCCATCTAAATGGATATTATTTTACTACAAATCACAAGCACAACAAAAGATCAAAAATTGGAAACAGGATGTTGATACTATCCTCCAAAATATCTTTATATCCGTGAACATGGAGGTATACGTCGTACTTATCGACAGTGACTACTTCCATGTCCATCTAATATATAAAATCAAACCCCATTTGCCATTCATTTGGGAGGAGTACGCTAAATGGAGTAAGCGCGGTTTCGATCGGCCTTATCCCGAGCAAGTCCTTGCAATGAGAAGGCGCAACCTTCGCAGAGAGCGGATTGTAGCTGTCACTGTTATGCTGTACGACGATACACTGGCGCTCATGCCTAATTACCTGCGTAATGCAAGAGATACAGTATCCAAGTTAATGTATTACATGACTGTTCATTTATTTGAGTGGGTGAACGGTACTGGGTACGTGAACCGTACTGATGTTGGCGGTCGTAGACTGAAGGATGGACGGTACTCGGGTGTTATTGGTGAACTAGCTGAAGGAAGAGCAGATTTTTCAGGTACACCGCTGGTACCCATGAAAGACCGCATTTCTCTGGTGAACTTTGTGCTGTCGCCGGCTCCGGTGGAAGCCATGTTCATATTTAAGAAGCCGTCGCTTGCATCCGTGGCTAACATCTACACGATGCCTTTTAGCTTCGGAGTATGGTTGGCCACTATTTTACTCATCGTTCTGCTGTCTTTGTCTTTTATTTTGAGTTATTGCATAGAAGAAAAAAAAAACAACCATTACAAAATCGGTTTGTTATCCAAACTGTCGGAAGGTTTCCACGAGAGTTTATGTATTATGTTCCAACAAGGCACCGCGTCTGATCCGGTCTCTTTCGCCAGCCGCCAGATTCTTCTGCTGGGTCTACTGGCCTTCATGTTCCTATACACTGCATATTCTGCTAATGTGGTGGCATTACTGCAGTCTACCACCAACGAGATCAACAATGTCGAAACGTTATTGCAATCACCGTTGCAATGTGGCGCTGAGAATATTGAATACATACGACATATTTTTAAGACCGAGACACGACCGATACATCGTGCGTTAGTTGATCGAAAAATAACTCCATTTGGCGACCAGGTCTACGCGCCTATTCCGGAAGGTATACGAAAAGTGAGAGAGGGCATGTATGCGTTCCACGTATTGCTGTCTTCAGGATACGACGAGATACAAAAAACTTTCCTGGATGATGAGAAGTGCAACCTTGGACGAATAAAATACATGTCCTTGGTGTACCCTTACTTCGGCATCGCGTACAAATCGCCAATTAAGGAGCAACTGAGAATTGGAGCTTTCCGCATAATCGAGAGTGGTGTCCAAGATCGGTCGACGCGCCGGGTCATGGTGGAGGCTCCGCAATGCGCGGGAGCTGCTATGTTCGCGCCAGTTCGTCTCACCGATGTCACTCCCACTATACAATTCATATTGTTGCTCTACACAATGGCTCTGCTTATATTTGGACTGGAGCTGTACTCGGACAGACGAAGTATACGCAAACGGAAACAGGAAGTGAATGAAAACACACATCACAATTGA

## > PflaIR21a

ATGAGAAGTTTCTTGCTGTTTTCGGTTTTGGTATATTCTTGCTCAGATTATGTGGCATGTGAAGAAATTGCTGAATTTTCTGAATTTTATCCGTCGCAGAGCTTGGTAAACTTTAAATTTAAATATAAAAATTATAGGAATAATGGGTTTGAATACAATAAAGTTAATTTTTATAATCATGATGAAAGTGATGCTTCTAAATGGAGAAATTTAACTTCAGAAACAAATTATCGTAACAGAGTTAAGCACAAAAGAGCCTCAGATCCTGTGTTTCATGGTCATCCGAAAACTAAAGAAGAAATATGGAATGAGAATTTTTTAAATCATACAACCACATTTGATCAGACGCCTTCTCTTATTAAACTTATTCATAATATAACTTTGACATATTTAAACGATTGCACACCAGTTGTATTATATGATAATCAAGAAAAAGCGGAGAGTTTCCTTTTTCAAAATTTACTAAAAGGTTTTCCTGTTACTTACGTTCTTGGATATATAAATGATAATAATTATTTAGTAGAGCCCAAGTTGATACATCCAGTGACTGAATGCCAACATTACATAATGTTTTCGATGGATGTCAAAGCAGTTGTAAAAGTATTAGGTAGCCAGCCCACGAGTAAAGTTGTTATTATAGCAAGATCGTCACAATGGGCCGTGCAAGAATTTCTTTCAGGACCACATTCAAGAATGTTCGTTAACTTATTGGTAATAGGACAAAGTTTCAAAGATGAAGACGCTGAAATACTGGAGGCACCATACATTTTGTATACCCACAAACTGTATACGGATGGTCTCGGAGCGAGCAAGCCTAAAGTACTTACCAGTTGGTCACACGGTAGATACTCCAGGAACGTGAATTTGTTTCCGAAGAAAATGACTGAGGGATACGCTGGACATAGATTTATAGTAACAGCAGCGAATCAACCACCATTCGTGATAAAAAGAATAGTATCCGACAAAGATGGCGGTAATCCACATACAGTATGGGACGGAGTGGAAATTCGACTGTTAAAATTACTTTCTCATAAAAACAATTTCTCTATAGAAATACTCGAGCCACGAGAATTGAATCTGGGACCAGGAGACGCGGTTTCAAAAGAAATAGCATTGGGCCGCGCTGATATAGGAGTAGCTGGAATGTATCTGACAACCGAACGGACATTAGCCATGGACTTGAGCGCTGTTCACTCTCACGACTGCGCTGTCTTCATAACACTCATGTCTACAGCATTACCTCGTTACCGAGCAATATTGGGCCCTTTCCATTGGCATGTTTGGGTAGCGTTGACATTCACTTACTTGTTCGGCATCATTCCGTTGGCCTTCTCTGACAAGCATACGTTGAAACATCTGATTAATAACGCTGGGGAAGTCGAAAACATGTTTTGGTATGTCTTTGGCACATTCACGAACTGCTTCACGTTTGTTGGCCGAAACTCGTGGAGCAAAACGAGAAAAGTCACTACAAGACTGTTGATTGGTTGGTACTGGGTATTCACAATAATCATTACAAGCTGTTACACCGGCTGTATAATAGCATTTGTTACACTACCCGTGTTCCCAGATACTGTGGATACGATTCAACAATTGTTATCAGGATTTTACCGTGTCGGAACATTAGATCGTGGAGGTTGGGAGAGATGGTTTGTCAATTCTTCAGATATTTACGCAAATAAACTTCTGAAGAAAATGGAACTTGTGCCTAATGTCGAATCTGGGATAAGGAATACAACTAAGGCATTTTTTTGGCCATACGCGTTTCTGGGTTCTCAAGCTGAGTTGGAGTATATTGTGCAAGCGAATTTTTCTAAGACCGAGTCCAAAAGGGCGCAACTTCATGTATCCAATGAATGTTTCGCCCCATTTGGGGTGACAGTAAGTTTTCCAAATAATTCCATATACACGGCTAAGTTTAGCGCTGATATAAGCAGGATGTTGCAAAGTGGAATTATCTATAAACTCACACGAGACGTGCGTTGGGACATGCAGCGCAGTAGCACTGGAAAATTTCTATCGGCTGGTGTCGGTTCCCTGACAATAACTTCCGCAGAAGAAAAAGGATTAACGTTAGAAGACACTCAAGGGATGTTCCTGCTTTTAGGTGCTGGATTCTTAATGGCTGCCACAGCCTTACTATCTGAATGGCTGGGTGGATTTACAAACTGGTGCCGTTACAAAAGGATTCGAAATGCCTTAAATAATACAAATGTGACTGAAGTGCGACGTCACCATGAAGCTGATGATGGTAGCAGTAGACCTAATTCTCATTCAAGAAGCTCAACTGCTAGTACTCGAGATACGCTCGAAGGTCATATCATAAACGTAAGTGAAGAAAATTTCACAATTCACAACGAATTCGATACAAGCACTTGGTCAACAAGAAGATCCAGCTCTGTAGATTTGGATAAAGAAATAGAAGATATATTTCTTAAAGATGACCAAAGAAAACGCATTAATTCTGAAGGAAGTTTAGCACAGACAGACGAAAGACTGTAG

## > PflaIR40a

ATGCGGGTCGCTGTTTTAACTCATCCGAGAAAAGGAGTATTTCGAGTTTACTACAACCAAGCTGTACCAAATCGTCTCCACCATTTAGAACTAGTAAACTGGTGGCATGGACGATTGTACAAATCCCCCGTTTTACCACCAGCTGACAGAATATATCGAGATTTCAAACAGAGAGTTTTTGAAGTACCAGTATTACATGCACCGCCGTGGCACTTTGTGAAGTACAACAACGATACGACGGTCAACGTGACAGGCGGACGAGACGATAAACTTCTGTCACTAATGGCCAACAAATTGAATTTCAAGTATAAATATTACGATCCGCCAGAAAGGAGCCAAGGTTCCAGCATATCTGGAAATGGAACATTTAAAGGAACACTTGGATTGATTTGGAAACGTAAGGCGCCCTTTTTTGTTGGAGACGTGACAATAACGTGGGAAAGACTGCAAGCTGTGGAATTTTCCTTCCCAACCCTGGCTGATTCGGGTGCTTTCTTAACGCATGCACCAGCAAAACTAAGTGAAACTCTGGCCATCATAAGACCTTTTCGCTGGGAGGTGTGGCCACTTGTGTTAGCAACTCTGATTGTGACCGGTCCAGCCCTGTGGATCGTGATAGCTGCACCGTCGCTATGGCAACGACGTTGGAAACGCGACGAACAAGTCCAGCTATTTAGCAAATGCTGCTGGTTTACCGCAACATTGTTTTTGAGACAATCTTCAAGTAAAGAGCCTTCTAATGCACATAAAGCTCGTTTGGTATCGGTACTTATATCGCTAGGTGCTACCTACGTGATCGGAGACATGTATTCGGCAAATTTAACTAGTTTGCTAGCGAGGCCGGCCAGGGAGCCCGCTATAGGGAACTTGCAAGCTTTGGAGGAAGCCATGAGAGAAAGAGATTATGAACTAGTTGTTGAGAGACATAGTTCTTCACTGACAATTTTACAGAACGGTACTGGTGTTTATGGAAGACTGGCTCGACTGATGAAGAGACAGCGTGTCCAGCGCGTACGCAGTATCGAAGTCGGCGTGCGACTGGTGTTGTCACGCAGACGGGTCGCTGTACTCGGTGGCAGAGAGACGCTTTATTACGACACTGAGAGATTTGAGTGA

## > PflaIR41a

ATGTTACTTTCTGTAACTAATTTAGTGCACATCGAATCACTATTGCAAATATTATTTGAAAAGTACATGTATGCTACTTATTGCATTGCTGTCGTGTCGGAAGGAAAATTTGCATTCGAATTCAACATTACTGGAAGTGTGATACACATAGAAGCAAATGGTAATGAAAGTCTTGTCCGAAATATTTTGGTTGCCTCTGACTTTGGTTGTACGGATTACATCGTTCAAATGAGTGACCCTAAAGTATTTATAAAAGATTTCGAAGAAGTAATTCACTTAGGACTCGTGCGAAAAAGTAACAGGCGACTCATATTTCTACCTCACGTCGATGATTTTGAAAGCGCAAAAAATCTGTTAACACTCCTCGATATGAAAGGAACAAGTTTTGTGGCAAATATTCTACTAGTTGTGCCGTCTGTTCAAGATCCGAATTGCACAATTTACGACCTGGTAACACATAAATTCGTTGGCCCCATTGAATCATCCAGTGATCCCTATTATTTAGACCAGTGGGATACCTGTGCACAGAAATTTAGAACTGGTGTCAACTTATTTCCTCATGATATGTCTGATCTGCAAGGGAAACTTGTTAAAGTTGCTTGCTTTACCTATAAACCATACATATTATTAGATATTGACACTAATTTGACGTCAATTGGTCGCGATGGAATTGAAATGAGAATTATTGAAGAATTTTGCAGGTGGATAAATTGTAACATTGAGATAATAAGGGATGATGAACATGCGTGGGGTGAAATATATGAAAATAAAACTGGTGTCGGTATCTTAGGACAAATCTTAGAAGATCGCGCCGATATTGGAATGACCTCCCTGTATTCTTGGTACGAAGAGTTCGTCGTTTTAGACTTTTCTGCAGCCCACATTCGGACAGCAGTCACTTGCGTTGCTCCCGCAGCGCGAATCTTGTCCAGTTGGGAACTACCCTTATTACCATTTACATTGTACATGTGGTTAGCGGTGGTTTTTACCTTCATATATGCATCTCTTGGATTAATTCTTGCTACACGCCGCTTTGATAGAGCTTTTTTGACAACCTTCGGTATGATAATAACACAGTCGCAACCTGATGCAGGCAGCTCTTGGCGTGTCAGGAGTGTCACCGGGTGGCTTCTCATTACGGGTCTGATAATCGACAACGCCTATAGCAGTGGCTTGGCTACCACATTTACGATACCTAAATATGAACCCTCCATTGACACTATCAAACATGTAGTTGAGAGGAAATTGGAGTGGGGCGCTACTCACGACGCTTGGACATTTTCACTGGCTCTTTCACAAGACCCAATAATCAAACAATTGTCGAGTCAATTTAGGGTAAAGTCTGCAGAGGAATTAAAAAAATTGAGCTTCACAAGAAGCATGGCATACAGTATAGAAAAACTACCGGCAGGTTACTTTGCAATTGGAGAATATATAACTAAAGAGGCTTCATGGAATCTGGAGTTGATGTTAGAAGACTTTTATTACGAACAATGTGTTGCGATGTTGCGTAAGAGTTCACCGTACACTGAAAAGATTAGTCAATTAGTTGGTCGACTGCATCAGTCCGGATTGATATTGGCTTGGGAAACCCAGGTTGCTCTAAAATATTTAGACAACAAAGTTCAGCTTGAAGTGAAATTATCGAGAACAAGAAAAGATATTGAGACTGTAGAAGCATTGACATTGAATAATATTGTGGGCATTTTCATCATTTATTTCATCGGCGTTATGGTTTCCGTTTTATTATTCGTAGCAGAAGTGATATATTTCACTTATCGACAATCATCTACCATTTATAGAAATACCTAA

## > PflaIR93a

ATGAAACGATGGCTAATGTTATTCGCTTTTATATTTCTGGAAGTCACCGCGGAAGATGTTCCTTCTTTGATAACTGCAAATGCGTCTTTAGCGATAGTACTCGATCGCCAATTTCTCGGGGATCAATATCAAGTAATACTGGACGACCTAAAGCATTACATAAAGGAACTGACAAGAGTAGAGTTGAAACATGGCGGTGTTGTTGTGCATTATTACTCCTGGACAACAATTAGTGTGAAGAGAGATTTCATCGCTGTCTTCAGCATAGCGTCCTGCGAGGCAACTTGGGAACTGTTCACCAGAACGGAAGAGGAGCAGCTCCTCCTTTTCGCACTTACAGAAGAAGACTGTCCAAGATTACCTTCAGAAGCGGCTATCACAGTCACCTTCATGGTACCTGGACAAGAGCTACCTCAACTGCTTTTAGACTTGAGAGGCGCTAAAGCATTCCGTTGGAAGTCTGCTGTTATTCTTCATGATGATACTCTGAGTCGGGATATGATTTCACGTGTGGTACAATCACTCACATCGCAAACCGAAGGAGACGATGTGTCTGCTATCTCTGTCTCCGTTTTTAAGATGAAACATGAAGTCAACGAGTACTTGAGAAGAAAAGAGATGCATAGAGTTTTATCTAAGCTACCCCTTAAAAATATAGGTGAAAACTTCATAGCGATCGTAACTACAGATGTTATGTCTACGATGGCTGAGACAGCAAGAGATCTTCTAATGACCCACACGGCAGCACAGTGGCTCTATGTCATATCGGATACGAACAAACATAATGGAAACCTTTCCAATTTGATTAACGCCTTATACGAAGGCGAGAATGTCGCTTACTTCTATAACATCACTGATGATGATCCTATTTGCCAGAACGGACTGCTGTCCTATAGTCACGAAATGATGAATGCGTTCGTATCCGCTCTGGATGCTGCGGTACAAGACGAGTTCTACGTGGCTGCCCAGGTCTCTGACGAGGAGTGGGAGGCTATTAGACCAAATAAGATTCAAAGGAGAGACTTGCTACTCAAACATATGCAGCAACACATTGCTGCTTATAGTAAATGTGGAAACTGCAGCACTTGGCGAGCCCTCGCAGCCGACACCTGGGGCAGTACCTACAGCGTGTACAGTGATCAGAACGAAACCCAACATCAGGATACGAAAAATGTTACTACAGTGGGGGTCATCGAAGACGTTGAACTTTTACAAGTTGGATATTGGAGACCCGTTGACGGCCTTAGATTATCGGACGTTCTTTTTCCTCACATCGAACATGGCTTTAGAGGAAAAGAGATACCTGTTGTCACTTATCATAATCCGCCATGGACAATCCTACATGTGAACGAGACTGGATCAGTTGTTAAATATGGCGGTTTGTTTTTCGACATCATGGACCAATTGGCCAAAACTAAGAATTTTTCAATGAAGCTACTTTTGTCTACGAATGTCAAAAATGATATGACTAATAAAACTAAGGACCTGATATTCAGCAAAAGTTCGAGCTTAGCGTTGATGGCAGTGGCCAAGGGTCAGGCCGCCATAGCTGCAGGTGCCTTTACCGTCATACCACAGCCACTTCCAGGGACAGTGTACACGCTCCCTGTCAGCACGCAGCCCTACTCCTTCATGATCGCCCGTCCTCGGGAACTCAGCAGAGCGTTACTGTTTCTGCTACCATTCACTACAGACACTTGGCTGTGCTTGGGTTTCGCCGTGATACTAATGGGCCCCACGCTTTACGTGATCCACAGGCTAAGTCCGTACTACGAAGTGATGCACGTACCACGCCAAGGTGGTCTGGCCACTATTCACAATTGCCTGTGGTATGTCTATGGAGCATTGCTCCAGCAGGGTGGCATGTACCTACCCCAAGCTGACAGCGGGAGATTAGTCGTGGGAACTTGGTGGTTGGTGGTGTTGGTGGTCGTGACAACGTATTCCGGGAACCTGGTGGCGTTCCTCACCTTTCCTAAACAGGAAATTCCTGTGACCACCGTAGCGGAACTATTGGAGAACAGAGATCTTTACACTTGGTCGATAACCAAAGGGTCTTATTTGGAGGTAGAGTTAAAGAACTCCGATGAACCCAAGTATTTGGCGTTGTTAAATGGAGCAGAACTGGTGACTGGCGCTAGCGGAGTTGATGGTAACTTGAGAAAAGGGTCCAGTAATGCTCACCTTCTTCGACGGGTCCGCACCGCGCGACATGCTATCATCGACTGGCGCATGCGACTCACTCAAATTATGCGCTCGCAGCATCTTCTCACTGACACCTGCGATTTCTCTTTGAGCACGGAAGAATTCTTTGACGAACAAGTGGCCATGGTACTACCTGCCGGCAGTCCGTATCTACCAGTTATAAACAAAGAGATTCATCGTATGCGCAAAGCCGGTCTGATAACGAAATGGCTGTCAGCTCACCTGCCAAGACGAGACCGCTGCTGGAAGAGTTCAACAGTGGCGCAAGAGGTGAACAACCACACCGTCAATCTGAGAGATATGCAGGGCTCTTTCTTCGTTCTGTTTTTAGGGTTCTTTTGTGCATCATCAGTACTCTTCATCGAGTTCTTCTACAACAGACGCAAGAGACGGAG

## > PflaIR75c

ATGAAAGGGATAAATAATTTCGCTAAAAAAGCATCCCAGAATTATATAAGAGTTGGTAATTTTGACATAAATGAATTGTCTTTGGAAATAGAAAAATATCTAATTAGAAGTAATGAACCAAATGGAATTTTATTGGACTCAAAATGCGAAACGTTTAACGAAATACTTTTAAAAGCAGCTGACAACTTACTGTTGGATGATAAAAACAAATGGCTTCTGGTTTCCTATTACGAAGCGAACATTTCAAGTAATACGACCGATCCAATTAATAAAGAAGACGGTACCAGCGAAAATATAATTAATGCAACAATTTTAATTGAAGATAATAATTCAGAGACAATGAATCATCAAAATATGAGACATGCAATAAATGACAAGTTACTGGAAGTAATTGATAATTTAAACATTAGTTTTGATACAGATTTAACCGTCGCCATTATGCATGATTCATTATACTATAAAATCTATGATGTTTTCAATTATGGTAAAATCCAAGGTGGTGTCGCAAACATGGATCTATTAGGATTCTGGAATTCGAAGGACGGCCTTCAAGTAAAAGAATGTCGTCATAAATTTTATGAAAGATGGAATTTTCAGCGCCTTGAATTGAGAATGGTAGCAGTGATGGATGCTCCACCGAAACAATTCCATCCTGACATGATAATCGGAGAAAATCCTGATCACGGTGTAGCTGTTATCACAAGGACCAGTACTATTCTACTCCACACTATCGCTGAAATTCACAACATCAGATTTAAATACACTATCTTGGATCGCTGGAGAGGAAGCTTCTTTAAGAATAGCACCAGAGTTGCAGCCAATTCTCTATATTTTAGAGAGCAAGACATCACACCGATACTTCGTTTGTTTGATGGAATAAGTGAGAAAATGGATATCGTCTACCAACCAGTGACTTTTCTGGCCACAAGATATTACTATAGAATTCCAACTGAAGGACCTGGGAAATTTGAGAATAAATTTCTTACTCCACTCACGTCAACTGTGTGGGGATGTACTGTGGCTATGTCTATATTGTGCACAGTCACCATTTTAGGTTCAGCATTGTTGGACAGGCGCCCATCGACCGTTCAATATGCAATATTTACAGTCATCGCTTCAATATGTCAACAATTTTACGAGGACGGCCTTGAAAATGAGAGAAGAATTGGTACTGCTCGACAGACGACCGTGTTGATAACGAGCACATCATGCCTGCTCTTGTACAACTACTACACCAGCAGCGTCGTCAGTTGGCTTCTAAACGGACCGCCGCCTTCCATCAGAGATCTCCAGGAACTTGCGGATAGCTCACTTGAGTTGATATATGAAGATATTGGCTACACCCGTACGTGGATGCATAATTCGGATTACTATTTCAACATCAGAAACGCAGCTGTAGAAGATCAAATGAAGAAAAAAGTTCACAAAAAGAAAAGCCTGGACCAACTTGTGTACACATCTCCAGAAGACGGCATTGAACGTGTGAAATCCGGAGGCTACGCTTATCATACCGAAGAAAACACAGCCAATATGTTAATTTCAAGACGATTTACGCAGAGTGAGCTGTGTGAATTGGGTTCACTACTATCGTTGGAGAAGAACATATTGTTCCCTTGTTTGCAAAAAAACAGCCCCTACAGGGAGTTTTTCTCATGGAGTCTCATGCGGCTCCGAGAACGTGGAATAGTACGATGCATTCAACAAAGAACGTACTCCCCTGAAGTCACCTGTGAAGGTAGTTCTCCTAGAGCTCTCGCACTTGGCGGCGCCGCTCCCGCGTTCATTCTATTAGGGTTTGGTTATCTGTTAGGATTAACTGTACTGCTGTTCGAAAGGTTATCAAAACCATTTCCAAAGGAAACAAAGTCCAAATCACCCTCAAAATAG

## > PflaIR87a

ATGTTTATAAGTATTTTCTTACCGTATTTACTTTTCGTACAATTGGTAACAATAGCTATAAGTGAGAATTCATTGCTAATGGCTGGAGGAAACCCAGCTCAACAAAAACAACAAACAAACCAAACAAAAACTGCAGAATGTGTTTTGAAATTATCCGCCAAATACTTTGTTGAAAAGAAAGCCCTAAGCGGCAGCATCGTCATCATCAATATTAACTCTTATGTTTCTGAGACGCAGAGACTATTATTACAAACCATACACGGTGGAATGAAATATTCAGTTTTGGTTAAAGATTCGTTTTACCCGCATGCAAACGCGTCTCGGTTTCGGGAAAAAGCGAAAAATTATATGTTGATTTTAGAAGATAAAACTGAATTGCAAAGAAACATACGGCAGTTGAACAAACTACCATCTTGGAATCCTCTCGCAAAAGCCATTGTTTTCTACGAGTTGAAAGACGAAGAAAATGGCGGAGTTGTTGCAATGGATTTTATCAACGAACTGAGAAATCATAAGCTTTTGACAACGATTATATTTATTCATTCGAAACATAATGAGCAAGTCACTGCTTACACATGGAGTCCTTACAGCGACACAAATTGCGGTGGATCATGTGATTCAGTTTATATTTTGGACAAGTGCAAAAATAACATCATAGAGAAAGTTCAAAATCAAAAAGAACTGCTTCCTCCAGATATGAAAGGATGTCCTTTAATTATGTATACGGTTGTTTCTGAACCATATGTAATGACACCTTTATATAAACAAAATAAAACTAGTTATAAAGATGCTTATATATTTGAGAAAGGAATTGAAATACGTCTTTTAAATATTATAAGTCAGTTTACTAAAATGACACCGATTATTCGTATGTCAGATAAGCCAGAAGATTGGGGAGATATTTATCTAAATGGATCAGCTACAGGTGCTTATAGTTTTATAAGAAATGATTCAGTGGATTTAATGTTTGGAAACATCCAAGTTAGCAAAACGATTCGTAAATGGTTTGACCCGACTGTCATATACACACAGGACGAGATTACGTGGTGTGTGCCAAAAGCCCGTCCAGCTAAAGCTTGGGATAATTTGGTTTTCATATTTCAGTGGATGTCGTGGCTGGCGATCTTATTTTTTTTTATTATAATGGGAACGGTTTTTCATATCATGTTTTATAAGGAAAACAACAGGAAAGTAACAAAATGGCCAACGAATTCTTTTCTGATGACCTTTAGCATGTTGCTCGGTTGGGGATCAACGTTTCAACCGAAGACGATCACATTCCGAATACTGATATTTGTTTGGCTTTCCTTCAGTTTGCTTATGGGGATTTCGTACGAATCCTTCCTTCGTACATTCTTAATGCATCCAAGATTTGAGAAACAGATAAGTACTGAAGCGGATCTAATAAATTCTGGAATATCATTCGGTGGTCGAGCTACTTACAGATCCTATTTCGGGAACAACAACGCAAACACGTCTTATTTGTACCGCAAATATTATTCCACATCATTTGCAGATGGCATTAAACGGGTGGCGTTTAATAAGAACTTTGCGTTAATTTCATCGAGACGTCAAGCTTTTTACCAGGACCAAAAATTAGGGAAAGGCGAGCCTTTAATTTATTGTTTTCCCGAAAGCAATAATCTTTATAAATACGGCGTTGTTATCATCGCTAGGAAATGGTATCCCATGCTCGAAAGGTTTAATAGCATCATTCGAAGTGTATCGGAGAATGGTTTGCTTGATAAATGGAATGAAGAGCTGGATACTGGTAAACTTAGTGGAGGCGTTAGTGCTATTTTGCCTCTGGGCATGAACCATTTACTCGGCGCTTTTATGTTTTTGGGTTTCATGTATATTGTAAGTAGCGTTGTATTTTTTTGCGAAATAGTTATCTGGTTAGTTAGAAGCCGTTCTCGATCTAAAGTATCTATAAAAAAACTACATAGAAATTGA

## > PflaIR75d

ATGGATACGAGTTTCATACTGAATTATTTTGTATCCAAGGACCTTTACTTGCTAACGGCATTCGTTTGTTTGGAACATGATCTGTCGATAATAAATAACGCTCGATCTAATGGAATGAGAATAAAAGTATGGTCCGACTTCAATCATTTACCTGAAATGCCAGCATACGACTATTACAAAGAAGGAATGGTCCTAGATCTTGATTGTCCTGATGCTTCCCTCGTACTACAAAAGGCGTCTGGTAATCGAGCATTCATTCACCGGTACTCGTGGCTTTTGCTTCATCGTGCCGATATCAACATCACTAACCTGCAGGAAATATTGTCATCTGTGGCTATTCTGCCTGACGCTGATGTCGTCTTGGCTGCCAGGGATGCTGTCGTGGATTTGTATAGAATAAAATCGGATATCTCTCTTTCTGTAACTAGGATGGCGATGAACAAAAATAGTAGCATTGAAGATATGAAGAGTCTTTGGAGGGAATTACCAACGGCTGTTGTTAGAAGAAGTGATTTAAAAAATGTTTCCTTAAAAGCGGTTACAGTTGTTACCCAGCCACAGTATTTCAAAGGATGGTCGGACTTGAGTAGTCGACACATCGACACTTTTCCGAAAGTAACGTATCCTTTGATGATGCATCTCAGAGAAGATTTAAATTTCAGGTACAATATGAGACAAGTGGATTTGTATGGGGAGAGCAACAACGGCTCGTTCGACGGAGCCGCCGGCATGTTGCAACGGAAGGAAGCAGAAATCGGTATCACCTCCATGTTCTTGCGAAAGGATCGTTCGGCAGTACTTCATTTTTCGGCCGAAACAATTGAATTAGTAGGCGCATTTATGTTTCGTCAACCGTTATTACCGACCGTGTACAACGTGTTCCTGTTACCATTCAGTCGGAGTGTCTGGTTGGCATGTTTGGCAGTTACCATAACATCAGCCGCACTCCTAACTTTGCTCGCTATTATCAACCGTTATTTCTCAAGCGTCGACGGTTCTCTACAAATGCTTTCAGTATCGGAATCTTTCTCGTTTGCTATTGGAGCTATTTGTCAACAAGGATTCTACGCGACTCCAAGCATTGTATCAATTCGTATGGTGATGCACTTCACATTGATGGTCGGATACTTTATCTTCACATCTTACTCGGCGAAAATTGTATCGATCCTGCAGACGCCGAGTGACGCAATAAAGACCATAAGTGACTTGACACACTCACCAATGAAACTGGGCGTGCAGGAAACCACTTATAAGAAAGTGTATTTTGCGGAAAGTACTGATCCAGCAACCCAGGAGCTGTACCGTGTCAAGCTACTGCCTCTTGGTGATGCTGCTTATCTCAGTGTTGTAGACGGTGTGGCTCGTATGCGAAATGGTTTCTTTGCCTTTCAGGTCGAGGAGAGTTCAGGTTACGACATAATCAGCAAAACGTTTACGGAAACTGAGAAATGTGGTCTGGCGGAAATACAGGCGTTCAAACTGCCTATGGTTATGGTGCCTATTGCAAAGCATTCTGGCTATCGGGAACTGATAGCTTCCAGACTTCGATGGCAACGCGAGGTGGGCTTGTTTGATCGTTCCCGTCGAACCTGGATGTCACGTCGGCCGGTATGCTTAGGAGTTGGTGGAGGATTCGTGACGGTGCGTGTTGAAGATATTCTACCCGCCTTGCATATGTTCTTCGCTGGAATAGTAATTGCTGTGTTTCAACTTATCGCTGAGTGTGTCGTTCATAAGGTATCCATTACTACATGTACAGTATTATCCAAGCACCTTAGTCAGATTTCTCCTCTTATAGTTATGAATAGTAAGTGGAGTACTGGGCTAAATCTGCTTACAGGGTCTTGA

## > PflaGluR1

ATGTTCGGTAAAAAGAGTTCTGTTATTTTTTGTGCGTCACTAGTATTTTTGGTGATATCGATAATACTGGCCACATGGGGAATACCGAAGATAGTGAAGAGGCAAATCCAGAATAATGTGCAGATAGAAAACTCGTCGAAAATGTTCGAACGTTGGCGCGTATTGCCAATGCCCTTAACATTTAAGGTGCATCTATTCAACGTGACCAACACAGACGACGTCAACCATGGTTACAAACCAAAAGTGAAAGAAATTGGACCGTATGTTTATAAGGAATATCGCGAAAAACTTATTTTAGGATATGGAGAAAATGATACAATAAAGTACATGTTGAAGAAAAGGTATATATTCGACCAGGAAGCGTCTGGTGGACTGAAAGAAGACGATCTCGTAACTGTTATACATTATCCTTACATGGCTTCTATTCTGACCGTGCTGGAAATGTCACCAGCTTTCGCATCGGTGATAAATAACGCCTTGAATACACTGTTTGGGGGAGTGTCGCAACCATTCCTAACGATAACCGTAAAGGAGCTATTGTTTGACGGAGTCTATCTCAATTGTACGGGTGGACAACAGAGTCTGGGTCTCGTATGCGGCAAGATCAAGTCAGAGAAACCTGCCACCATGCGCCCAGCAGAAGATGGTACCGGATATTACTTCTCAATGTTCAACCATCTGAACAGCACTGAAACCGGCCCATACGAAATGGTACGCGGAACAGAGAATTTGTACGACTTGGGGCACATAGTGTCGTACAGGAACAAAACTGTGATAGAGACGTGGGGGAACCCCTACTGTGGGCAATTGAACGGAACGGACTCCACGATCTTTGCGCCGGTAGACGGATCTAATGCACCAGAAAAACTATATACGTTTGAACCAGACTTATGCAGATCCCTGTACGCGAGTCTGGTCGGCGAACGGACCCTCTTCAATATGTCAGCATATTATTACGAAATCTCCGAGACAGTTCTGGCATCGAAGAGCGCAAATCCGAATAACAAATGTTATTGCAAAAAAAATTGGAGCGCTAAACATGATGGTTGTCTCCTAATGGGGGTTCTAAATCTGCTGCCGTGTCAAGGCGCCCATGCTATAGCATCTCTCCCACATTTCTATCTGGCTTCAGAGGAGCTCCTGGAGTACATCATCAGCGGTATCGAACCTGACAAGGAGAAGCACAACACCTATTTGTACTTAGAACCGGTGACTGGTGTGGTCCTGAAAGGGCTGAAACGTCTTCAGTTCAACATAGAATTACGTAAAGTTGAATCCGTTCCGCAGTTGGCAAAAGTGCCAACTGGTTTGTTCCCATTGTTGTGGATAGAGGAAGTAAGTATCAAATACTCCTACATAAATATAGGGTTCACAATATGTTGA

## >PflaGR1

ATGATAAGTGAAAATAATAAAACAAATGAGGTTAAGAAATGCAATTTAATTTATACGTTACGACCAACGAAAGCTCTGTTAAATTTCTTCGCGTTATCCTATAATGTCAAAAAAGAAACGCAGGAGTTTAAAAGACAATGCTTGTCCAAAATTATAATAATGGGTATAATCATTGGCTTACTGAATTTTACGTCTTTGGCCCTTAAAATTTTATTTGAATATGAGAGTCTACCTGACCTCTCTGTACGTTACATGGACGTATTTCAAGTTATTTGCGGATACTGTCAATACATAATTGATTTATATTGCGTTTACTCATATGGAAATGAATTATGCATTAATTATTTTGAACAGTACAGCAATATGGACAAAATACTGGGAGCTCTATATAATCACGAAATTAAATCCAGATTACAAAAATTATCATGTTTCCTAGTTTCCTTATATGTTGTAGCAACAGCGTGCGATTTCATTGCATGGGGTTTGCGTATAGGCTGGATAACACCTCTGTTATACACAATAGAAATTATTTTTGCTTTAATGAAGACATTAACGGTGTTAGATATGATTTCTCATATAATGCAAGTTGAATACCGATTAAGAATTATCGCTGAGTTGCTCCAAGAAACGCATAACTCGAACGGAACAGCACAAGACGTCAATTATGGAAAGGATCAAAGTAAACTCTACTGGTTCCATTCGTGCTGTTCTACGCCATCTAATGAGTTAAAAAAAATCTTGAAAACTATGAAATGTCTTTCGCCTGACGGTCACCACGAGATGAAGTGTCTATACAAATGCTACTTATTGCTACTCGAACAATGTGCGTTTATTAACTCAATGTATGGGACGAGGATCCTGATAATCATCTGCAATCTGCTTGTAAACATGGTGGCATATTTGAACATTACTTATAGAATTATCATCAAATCACAGGCAACAGATAATATATTGCTGAATTTATCGATATACTTTCCTGCTGTATCGGGTGTGATGCGACTTGTTGCTTTTGTCTCATTACTGGTCACGCTCGTGGCCCAGTGCGAGAGGGTCTACCAACAGAGATACAGGATTATACGTATGATAGACTACATCCTTTTCGACAGAAATTCAGATCCAAACTTGAAAGCAGTATTGGAAGTTTTACGTAATTTGGTACTATCGAGAAAAATTTCCTTCCACGTGGCAGACCTCGCCAGTTTGAACTACCCTTTGCTAGTCTCCACGGCCTCTATTGTTGTAACTTATACCATAATACTTTTGCAAAATGCGTAA

## >PflaGR2

ATGATTCCATTGAAGGGAGATCCACGTCAACAAAACGCCTATTTCACTATGAAACTCTTGCATTGGCTGCTCAACATATTTAGTCTCAACGGCAACATAAGAAAACTAAACGTCGCCGAAGAACGTCTATTGAAATTGAAACTTTTCATAACTCATATTATCTATATTGTTGTCATGTATTACGTGACTATCGCCATTTTGAGAAACCTGGCTTTAAACAGAGACATTGAGATATTTCCAACCATAAGTCAAATGTTAGGAATGTCGGAAGAATATGTTAATTACGTTATTTACACGTACTACGCACGTACTACGGATAATGTGAAGTATTATAACATTTACAATGTAATCGACACAATATTGCAATTGACATTTTTAGATCTGAAGAAAATAAGAAGAAACGTCAACCTCGTGCTGCTGAGTTCACTAACCGTATTATTTCAGTATGTTGTTTATCATTCGTACTACTTTTTGCCTATTTTGATCAAAAAAGGTACCGGATTAACCGTTTTGTTGAATTTCATTGTATTGTTGAAGACATTGGGAGTTACAAATTACATGTGCAACGTCTTACAAATTCAATCTCGACTCAAAATCATCGCTGAACAATTAGCAAATTATGAAGACAGCGCTAGTGCTTTTGTAATGGTCGCTCACACAAATGAAGAGAATGTTATATCTCAAAGAGCATCAACAAGATACAACCTTATTCACTTTCCTCGTATACGTAATCACAAAATCATGCGTTTAGTCGAAAGTTATTCACTCTTGGTCGAACAGCATTCGACAATTAATGATTTTTTCGGAAACAGGGTCTTCGTGTCCGTCTGCTGCATCATATTCGACAGCATCGGCGTTTTGTATGTGACAGTTGCGATACCGATCGGCCATTTTTTTCAGGATAATACAAACAATCAAACTTTTGAACAAATGCCAGCATTACCCATGCTCAGTCGGTTTATGACCAACATGATACTGCTGGTGTCCATGGTGTATCGTTGCGAGAGATGTTATGAAGAACGAAACAAAATACTGAACAATATTGACAAACTGATATTTTATAAGAAAACTGGAGTGAACACAAGGAAGGATTTAAGACTGTTCAGAATGATGGTGAAGGTTCGCCCGATCGTGTTCCACGTGAACTACTTCTTCTCCCTGCAGTATCCTCTACTCATGTCCATTCTGTCCGTGTGCATTACATATGCTCTCGTTCTGTTTGAATAA

## >PflaGR3

ATGATTCCATTGAAGGGAGATCCACGTCAACAAAACGCCTATTTCACTATGAAACTCTTGCATTGGCTGCTCAACATATTTAGTCTCAACGGCAACATAAGAAAACTAAACGTCGCCGAAGAACGTCTATTGAAATTGAAACTTTTCATAACTCATATTATCTATATTGTTGTCATGTATTACGTGACTATCGCCATTTTGAGAAACCTGGCTTTAAACAGAGACATTGAGATATTTCCAACCATAAGTCAAATGTTAGGAATGTCGGAAGAATATGTTAATTACGTTATTTACACGTACTACGCACGTACTACGGATAATGTGAAGTATTATAACATTTACAATGTAATCGACACAATATTGCAATTGACATTTTTAGATCTGAAGAAAATAAGAAGAAACGTCAACCTCGTGCTGCTGAGTTCACTAACCGTATTATTTCAGTATGTTGTTTATCATTCGTACTACTTTTTGCCTATTTTGATCAAAAAAGGTACCGGATTAACCGTTTTGTTGAATTTCATTGTATTGTTGAAGACATTGGGAGTTACAAATTACATGTGCAACGTCTTACAAATTCAATCTCGACTCAAAATCATCGCTGAACAATTAGCAAATTATGAAGACAGCGCTAGTGCTTTTGTAATGGTCGCTCACACAAATGAAGAGAATGTTATATCTCAAAGAGCATCAACAAGATACAACCTTATTCACTTTCCTCGTATACGTAATCACAAAATCATGCGTTTAGTCGAAAGTTATTCACTCTTGGTCGAACAGCATTCGACAATTAATGATTTTTTCGGAAACAGGGTCTTCGTGTCCGTCTGCTGCATCATATTCGACAGCATCGGCGTTTTGTATGTGACAGTTGCGATACCGATCGGCCATTTTTTTCAGGATAATACAAACAATCAAACTTTTGAACAAATGCCAGCATTACCCATGCTCAGTCGGTTTATGACCAACATGATACTGCTGGTGTCCATGGTGTATCGTTGCGAGAGATGTTATGAAGAACGAAACAAAATACTGAACAATATTGACAAACTGATATTTTATAAGAAAACTGGAGTGAACACAAGGAAGGATTTAAGACTGTTCAGAATGATGGTGAAGGTTCGCCCGCTCGTGTTCCACGTGAACTACTTCTTCTCCCTGCAGTATCCTCTACTCATGTCCATTCTGTCCGTGTGCATTACATATGCTCTCGTTCTGTTTGAATAA

## >PflaGR4

ATGGGTATAATCATTGGCTTACTGAATTTTACGTCTTTGGCCCTTAAAATTTTATTTGAATATGAGAGTCTACCTGACCTCTCTGTACGTTACATGGACGTATTTCAAGTTATTTGCGGATACTGTCAATACATAATTGATTTATATTGCGTTTACTCATATGGAAATGAATTATGCATTAATTATTTTGAACAGTACAGCAATATGGACAAAATACTGGGAGCTCTATATAATCACGAAATTAAATCCAGATTACAAAAATTATCATGTTTCCTAGTTTCCTTATATGTTGTAGCAACAGCGTGCGATTTCATTGCATGGGGTTTGCGTATAGGCTGGATAACACCTCTGTTATACACAATAGAAATTATTTTTGCTTTAATGAAGACATTAACGGTGTTAGATATGATTTCTCATATAATGCAAGTTGAATACCGATTAAGAATTATCGCTGAGTTGCTCCAAGAAACGCATAACTCGAACGGAACAGCACAAGACGTCAATTATGGAAAGGATCAAAGTAAACTCTACTGGTTCCATTCGTGCTGTTCTACGCCATCTAATGAGTTAAAAAAAATCTTGAAAACTATGAAATGTCTTTCGCCTGACGGTCACCACGAGATGAAGTGTCTATACAAATGCTACTTATTGCTACTCGAACAATGTGCGTTTATTAACTCAATGTATGGGACGAGGATCCTGATAATCATCTGCAATCTGCTTGTAAACATGGTGGCATATTTGAACATTACTTATAGAATTATCATCAAATCACAGGCAACAGATAATATATTGCTGAATTTATCGATATACTTTCCTGCTGTATCGGGTGTGATGCGACTTGTTGCTTTTGTCTCATTACTGGTCACGCTCGTGGCCCAGTGCGAGAGGGTCTACCAACAGAGATACAGGATTATACGTATGATAGACTACATCCTTTTCGACAGAAATTCAGATCCAAACTTGAAAGCAGTATTGGAAGTTTTACGTAATTTGGTACTATCGAGAAAAATTTCCTTCCACGTGGCAGACCTCGCCAGTTTGAACTACCCTTTGCTAGTCTCCACGGCCTCTATTGTTGTAACTTATACCATAATACTTTTGCAAAATGCGTAA

## >PflaGR5

ATGATAAGTGAAAATAATAAAACAAATGAGGTTAAGAAATGCAATTTAATTTATACGTTACGACCAACGAAAGCTCTGTTAAATTTCTTCGCGTTATCCTATAATGTCAAAAAAGAAACGCAGGAGTTTAAAAGACAATGCTTGTCCAAAATTATAATAATGGGTATAATCATTGGCTTACTGAATTTTACGTCTTTGGCCCTTAAAATTTTATTTGAATATGAGAGTCTACCTGACCTCTCTGTACGTTACATGGACGTATTTCAAGTTATTTGCGGATACTGTCAATACATAATTGATTTATATTGCGTTTACTCATATGGAAATGAATTATGCATTAATTATTTTGAACAGTACAGCAATATGGACAAAATACTGGGAGCTCTATATAATCACGAAATTAAATCCAGATTACAAAAATTATCATGTTTCCTAGTTTCCTTATATGTTGTAGCAACAGCGTGCGATTTCATTGCATGGGGTTTGCGTATAGGTTGGATAACACCTCTGTTATACACAATAGAAATTATTTTTGCTTTAATGAAGACATTAACGGTGTTAGATATGATTTCTCATATAATGCAAGTTGAATACCGATTAAGAATTATCGCTGAGTTGCTCCAAGAAACGCATAACTCGAACGGAACAGCACAAGACGTCAATTATGGAAAGGATCAAAGTAAACTCTACTGGTTCCATTCGTGCTGTTCTACGCCATCTAATGAGTTAAAAAAAATCTTGAAAACTATGAAATGTCTTTCGCCTGACGGTCACCACGAGATGAAGTGTCTATACAAATGCTACTTATTGCTACTCGAACAATGTGCGTTTATTAACTCAATGTATGGGACGAGGATCCTGATAATCATCTGCAATCTGCTTGTAAACATGGTGGCATATTTGAACATTACTTATAGAATTATCATCAAATCACAGGCAACAGATAATATATTGCTGAATTTATCGATATACTTTCCTGCTGTATCGGGTGTGATGCGACTTGTGGCTTTTGTCTCATTACTGGTCACGCTCGTGGCCCAGTGCGAGAGGGTCTACCAACAGAGATACAGGATTATACGTATGATAGACTACATCCTTTTCGACAGAAATTCAGATCCAAACTTGAAAGCAGTATTGGAAGTTTTACGTAATTTGGTACTATCGAGAAAAATTTCCTTCCACGTGGCAGACCTCGCCAGTTTGAACTACCCTTTGCTAGTCTCCACGGCCTCTATTGTTGTAACTTATACCATAATACTTTTGCAAAATGCGTAA

## >PflaGR6

ATGATTCCATTGAAGGGAGATCCACGTCAACAAAACGCCTATTTCACTATGAAACTCTTGCATTGGCTGCTCAACATATTTAGTCTCAACGGCAACATAAGAAAACTAAACGTCGCCGAAGAACGTCTATTGAAATTGAAACTTTTCATAACTCATATTATCTATATTGTTGTCATGTATTACGTGACTATCGCCATTTTGAGAAACCTGGCTTTAAACAGAGACATTGAGATATTTCCAACCATAAGTCAAATGTTAGGAATGTCGGAAGAATATGTTAATTACGTTATTTACACGTACTACGCACGTACTACGGATAATGTGAAGTATTATAACATTTACAATGTAATCGACACAATATTGCAATTGACATTTTTAGATCTGAAGAAAATAAGAAGAAACGTCAACCTCGTGCTGCTGAGTTCACTAACCGTATTATTTCAGTATGTTGTTTATCATTCGTACTACTTTTTGCCTATTTTGATCAAAAAAGGTACCGGATTAACCGTTTTGTTGAATTTCATTGTATTGTTGAAGACATTGGGAGTTACAAATTACATGTGCAACGTCTTACAAATTCAATCTCGACTCAAAATCATCGCTGAACAATTAGAAAATTATGAAGACAGCGCTAGTGCTTTTGTAATGGTCGCTCACACAAATGAAGAGAATGTTATATCTCAAAGAGCATCAACAAGATACAACCTTATTCACTTTCCTCGTATACGTAATCACAAAATCATGCGTTTAGTCGAAAGTTATTCACTCTTGGTCGAACAGCATTCGACAATTAATGATTTTTTCGGAAACAGGGTCTTCGTGTCCGTCTGCTGCATCATATTCGACAGCATCGGCGTTTTGTATGTGACAGTTGCGATACCGATCGGCCATTTTTTTCAGGATAATACAAACAATCAAACTTTTGAACAAATGCCAGCATTACCCATGCTCAGTCGGTTTATGACCAACATGATACTGCTGGTGTCCATGGTGTATCGTTGCGAGAGATGTTATGAAGAACGAAACAAAATACTGAACAATATTGACAAACTGATATTTTATAAGAAAACTGGAGTGAACACAAGGAAGGATTTAAGACTGTTCAGGATGATGGTGAAGGTTCGCCCGATCGTGTTCCACGTGAACTACTTCTTCTCCCTGCAGTATCCTCTACTCATGTCCATTCTGTCCGTGTGCATTACATATGCTCTCGTTCTGTTTGAATAA

## >PflaGR7

ATGAAACATTACGGTGACTACATCGAAAGAACTATAACTACTTTCTACGTGTTCTATGCTATGAAGAAAATACCTTACAGTTTCAGTGGTTCCCCAAAATATATTCAAGAAATAGTCGATATTGACAACTCTATTAAAAGTAATCTTGGTGAAGCTGTTAATTACCGAAGGCTTGCATATTCTGTGTTTATTCTAGCGCTTGGAATCCTAGGAGTAACCTTAATGCGCTTGGGCACCATTTGGATCAGCCTAAATAATTCAAATTTAACATTGCCTTCAGAACTTATTATACAAGTCTCATTTACAAATGGAGTGGCTCTTATTATGACGGCACATTATTGCGTGCATTTGAGACTGTTGAAAGAACGATTCTCGATTGTGAATAAAACTATAAATAAGATAAAGGAATCATGCTCCTGGACTGAGTTAGTGATGAATGTGGATACGAACTCAAGGATAAATGAGAGTCAGGACAAACATTCCGGATTAAAAATAAGGGCATGCGCAAAGACATATGGCTTGATATACAAAGCAACGTTAACGACTAATGATTTCTTTGGTTTCTCCTTAGTGCTTACTATGGGATTTGGCCTTTTTGGCATAGTTCTTAACTTGTTTTACCTAATGGAAGCTACTGCTACGGGATTACCTAACGATGTCCAGAGATACTTCAATTTTCTGATATATATTTCTTGGCAGATATTTTTTGGCCTCGGAATAATTTTCCTTACTGTGTTTTATTCTGAAGCTGCCGTGAAAGAGGCAAGGAGTACTTCATATGTGTTGCATGAGATTATCAATAGCGGATTGAGTACACATATAACGTCCGAGGCAATGAAAATGTCTACGCAATTATTGCATCAAGTTCCTCGTTTTCATGGATGCGGCCTTTATGTTATGGACTACAGTTTGTTGTTGGAGGGAGCCCGGGCAGTACTCACGTTTTTGGTTTTGCTGTTGCAGTTTGTAATGGATTCATAA

## >PflaGR8

ATGTGTATATACAGTTATATTCTAGTCACATTTCTAGTTATTTGTACGATATTAGGTTTAGCAGCGGAAATAAATGTGGGCGTTGAATTGTCTGTGCGGATGTCATCCCGCATGTCGCAGTTCGTGTCCACTTGTGATGTGCTGGTGGTGGTGGCCACTGCCGTCGCCGGGGTGTACGGTGCACCAAGACGCATGAGGAACATGCTCAAATTCATGACCAGCGTTGCTTCTGTAGACACGAGTATAGGTGCTCAGTATTCAGCGGTGACTGAACGGAAGCTGTGTGCGGTTCTGCTCAGCATACTTATATTCTTCACCGTATTAATTATAGACGACTTCACTTTCTACGTGATGCAGGCGAAGAAAATAGACCGGCATTGGGAGGTGATGACGAACTACATATGCTTCTACTTGTTGTGGTACGTGGTCATGATATTGGAGTTGCAGTTCGCGTTCACAGCTCTCTCGTTGCGGTTACGATTCGCTGCCGTCAACGATGCGCTCGCGCTCACCGCCAGGGATATATCTTTACCATTAGGTAAATTGAACGAGCCAACTGCCTTGAATATGTTCGCGATACGAGTTGCCCCTGTGGATGTGACGCAGCGGACCAGCAATGGGAACGCGATCCTGATGGACTCTCTGAGCGAACACACCGTTATCATAAAGAAAAACACATTTGGATCAGCTCGGCTGGCGGTGGCGGCTCCGGAGGCTATACGGCGCCTGGCTGCTCTTCATGCTGCTCTCTGTGACGCGGTGCAGCGGCTGGACGCCAGTTATGGGGCGCCACTGATCGTGGTCCTCATTTCTATGCTGCTACATCTCATCGTCACCCCCTACTTTCTCATCATTGAAATCATGGTGTCGACAAAACGAATTCACTTCCTGATACTTCAATTCTTATGGTGCGTCACACACATGCTGCGTATGTGTGTAGTAGTGGAGCCATGTCATTATACTATCCAGGAGGGCAAGAAGACCGAGCAGTTGGTATGTCATCTGATGATGTCCTGTCCGTCGAGTGGGGCGCTGCCATCGAGGTTGGAGTTATTCTCTCGCCAACTCATGTTTCGATCCGTGACTTACTCTCCTCTTGGGATGTGTGTTCTTGACCGTCCACTCATTGTTTCTGTAATAGGAGCAGTCACTACTTATCTGGTCATATTAATTCAATTTCAGAGGTATGACTC

## >PflaGR9

ATGATCTTGAGATCGATAAAGAAGTTCGATGACTACATTTATGCCATCTTGTTCGTCGTATTCCTCGTCCCACATTTTTGGATACCATTCGTCGGTTGGGGCGTGGCGCACCAGGTCGCTATTTATAAGACCAGTTGGGGAAAATTTCAGGATGTGGAAGACGAGTGTACGGCGCAGCTGATATCCCGCTACCGGTACCTGTGGCTGAACCTGTCCGAGCTGCTGCAGCTGCTGGGCAATGCCTACGCGCGCACTTACTCAACCTACTGTTTATTTATGTTTGCTAACATAACGATCGCGGTGTACGGGGCGCTGTCCGAGATACTGGATCACGGGTTCGGCTTCAGCTTCAAAGAGATGGGCCTGTTTGTGGACGCGGCCTATTGTTCCACGTTGTTATTCATTTTCGTGGACTGTTCTCATAAATCAACGCTCAAGGTGGCTGCGGGTGTTCAGGACACACTGCTCTCCATAGACGTGCTCGCAGTGGACCGGCCTACACAAAAAGAAATTGACCACTTCATCCTCGCAATCGAAATGAATCCAGCCGTGGTGAGCCTGAAAGGTTACGCTGACGTCAACAGGGAGCTTTTAACTTCGGCGATAGGAACGATGACAATATATTTGTTGGTGCTGCTGCAATTCAAGATCTCGCTTCCGAAAGATCCTCAGACGTGA

## >PflaGR10

ATGTTCTTGACGCACATTCTCTATATTGTTGCTATGTATTTCATAACAATCGCCATTCTAAAAAATATTGCCTTATATAAAGCCGTGGAGATATTCCCTACAATATGTCAAATATGTATAATGTCCGAAGAATATTTTAATTACGTTGTTCACATGTACTCTGCACGTACTACGGATAATGTAAAGTATTATAACATTTACAACGTTATTGACACAATTTTGCGATTGGCACTTTCAGATCTGAAGAAAATAAGAAGAAATGTTAACTTCGTGTTGCTTAGTTCACTAACCGTATTGTTTTGCTATATTCTTTACCATTCGTACTACTTTTTGCCTATTTTGATCGAAAGAGGTAGCGAGATAACCATTTTACTGAATATTTTTGTTTTATTGAAGACATTGGGAGTTACAAATTACATGTGTAATGTTTTACAAATTGAATACCGACTGAATGTAATCGTTGAACAATTAGCAAATTATGAAGACAGCTCTAGTGTTTTTGTTGTTGTCACTCATACAAATAAAGAGAACAAAATATCCCAAAGAGTATCGAAGAATCAAATTAATAATTTTACGAGTTTTCCTCGCATACGCAACGATAAAATAATGCGTTTGGTTGAAACTTATTCACTCTTGGCCGAACAGCATTCGACAATTAATGACTATTTCGGAAACAGGATTTTCGTATCTGTCTGCTGTATAGTATTCGACTGCATCGCGGTTTTGTATGTGACAGTTGCAATACCCATTGACCATTTTTTTCAGGGTCATACAAAAATTCCAAACTACGAACATGTGCCAGCTTTAGCCATGTTCAGTCGGTTTATAAACAATATGATATTGCTAGTTTGGTGTATCGTTGCGAGAAATGTTATGAAGAACGAAAAAAAATACTGA

## >PflaGR11

ATGTTGTGCGGTTTCGCGGATATTAGTAATGCATATAATAATGTTTCCATTGATTCGTTATATCAGTGTATGGTTGAGTACGGTGTGGATCCACTATTGTCTAATTACGTGAGGAATTTTTTGAGGGATCGTCATTTGGTCTATAGGTTGGAGAGCGGTGAAATTTCAAGAATCTGTCGCAAAGGTTTAGCCCAAGGTGATCCCATGTCTCCGATCTTATTTAATATTATTACTACATTCATTTGTAAAAGATTTGATAAAGATGTAGAAATATCTCAGTACGCAGATGACTTCGCCTTTTACTGTAGGAATAAAGATATTTATGTTTGCGTTGAAGCTATCCAAAGTACATTATCAAATTTTTCGGTACTGTTGGAATCTATTGGACTAGATATCTCCATTCCCAAAACTAAAATTTGTGTTATAACCAGAAAATATCGGGTTCCGTCCATTAGTGTAAAAATTAATAACACAGCTATAGAGATAGTCGATTGCATAAAATTTTTGGGTCTCTGGGTTGATGGTCGTTTAAATTGGACAAAACACGTCAAAGAGGTTGCTCAAAAGTGTACTTCCTACATTAATTCTTTAAGTTATATTAGCGGGTCTAAATCCGGCGTTCACCCTGTTCATTTGAAGCGTATTTATATAAGTCTGATTAGGAGTAAATTGGAATATTGTTCTTTTTTTGTTTTCTAA

## >PflaGR12

ATGGCGCCGTTAAAGTTTGACAAGAAGCAGAAAACTCATACGATGCGCCTGTCTCGTACCTTATGCTACGTCTCCAATGTGACCATCACAATAAGCAATGTAATTCTCCTGACGGGCCTTATGATTGATTTGGGCCTGAGCGATGACAAGTCAGTGCGCGTGGGAACACCTATCAACCGTGGTTGTTGGATTGTGGATGTCGCTGTGGTGGCCATCGTCTCCTTCGCGGGATCTTACGGCGGAAGAAACAGAACTCTACATATGATACGATATTTAAATACCCTCGCAAAGATAACAGCCCAATCAAGTTCTAACGTAAAGAAGAGCAATGAAAAGAAGAAGATGGTGTTTGCGGTAGCGTCTATATGTTTTATGGCCATGCTTGTGGGCACGGACCTCTACAAGATGTACAGTTTAACCATAATGCACGATGAGGACTGGAACGTAACATGTATGTACTTGACGTTTTACTTCGAGTATTTCTTGCTGTTGCTTCTTCAGTTGGAGTTCATGATGAAGGCGCTACTCCTGCGAGAGGCGTTCCAATCCATCAACGACGAGCTGGACATTCTTCTTCTAAAATTGGCGCGACCCGATAGTCTTCGTAAGAACTTCGTTTCTAATAATCACTTCACAATTCCTAAGGCTCATCCACCTAAAGGTGCAGTGAATGCCACATTTGACACTGTAACTGTAAACAATGAAACCAAAACTTTTGATTGTACCGAAGACAACAAAGCGCCTTTAGTTATCAGTCGTCTCTCTCACACGTACGGCTCTCTTTGCGTTTTGCTGAGAGGGCTGAACGACAGCTATGGTGTCGTTAATTTGGTGTTGCTTGTGGCTATTCTTCTCCACCTAGTCATCACGCCGTATTACCTGATAGTCCACGTCATAGATACAGGCAACATACAGCTCTCTATGATGGGCCTGGTCGTACAACTGTGCTGGCTGTCATTTCATTTCACCTCAATGCTGATGGTAGTTGAGCCTTGCCATCAAACAACAGAAGAGGTGAACAAAACACAGATATTGATAGGTCAGTTGACACGGCTGTCCAGGGACGATCAGCTGTCCGCCGAGCTGGAACTGTTTTACCGCCAACTCCGCCTGGAGACCGCTAGCTACTCGCCGCTAGGGATGTGCACTCTCGCAAGACCTTTAATCACAACGGTCCTTGGAGCAGTCACAACGTACCTGGTAATCATCATACAGTTTC

## >PflaGR13

ATGTGGATCCTGTTAAATATCATGTCGGAACTTATAAAATGCATTCACAAAGCTATCGATAGTTACTCTATAGCAACAGCTGAGGGCGCTATGTTTGACGCAACAAATATCCATCTTAGGACTGTAAGTGTTAAAGAATGTTACAAAATGGAGGACTTATACGAAGTGTACACAAATATTGAGCTTATTTCGGTTTTATTCAATGAATCTTTGGGTGTTCAGTTGACTATTATGATAACCATGACTACTACTTTTCTTGGAATGTTGTACTATGTTACTGTTATTAGCGACCAATTTACAGATCAAGCATACTTATTGTTTTTTGTGAATATTTGGATATTTTGTGTGTTCATGCAATTATGGAAAGTGGCAAAAAGTGCTTGTAAATTGCAAGCTTGTGTTGAGGAACTTCGACGAAATCTGTTGGGATTACTAGTTCATTCAAATACAGGTGACATTCACTACAAACCCATTAAGAATCTTTACCGGCTGGTGACACAGTGCCCAATAAAGACAGCGGCGTTTGGCTCCATCGTCGTGGACATGACCCTGTTACCAACGTGTCTCGGCATATCATCATCATACACTGTTATAGCTTTGCAATGGAATAATGTAATATAA

## >PflaGR14

ATGGCGGGTGAAGTGCGTAAAATTTATCACATTGACAATACTGACTCTTGTTACACTTTAAGGCCGCTAAGAAAAATGTTAAGTTTTTTTGCACTATCGTACCATATCAAGCAAGAATCGCTAGAATACAGCCAACTACGATTGTTCAAGATTTTAACGATGGGAACTTTAATTGGTTTCTTAAATTTTTCAACGCTGACACTAAAAATTTTATACGAATTTGATACTATATCAGACGCTTCTATACGATATATGGACATATTTCAAGTTATTTGTGACTATTGCCAATATATAGTGGATTTGTTTTTTGTTTATGTATATGGTAAAGAGGTCTGCGTGAATTATTTCCAGCAATATGAAAACATGGATAGAATGTTTGGTAAACTGTACGATAAGGAAATAAGAACTAGATTATTAAAATTAGCATGCCTTCTCATGTCTGTGTGTGCTATAAGCGCAGCATGTGATTTTATAGCTTGGGGCCTGCAGATAGGTTGGATGATTCCCTTATATTACACCGTTGAAATTTTATTTTCATTAATGAAGACGTTGACCATACTAGACATGATCTCGCATGTAATGCACGTTGAATACCGATTAAGAATCATCGCAGAGGTGCTTCAAGAAAGCCATGATTCTTTTGGAGAACAGGGCGAATATGGTCGCGGTGAACACAGGGATAAGCACTACTGGTTTCATCCTGGCATT

> PflaSNMP1

ATGAAGATGCCGAAACATTTGAAGATCGCGGCCGGTTCTGGTGGTGCGGCGTTATTTGGTGTACTCTTCGGTTGGGTGATTTTTCCAGCGATACTGAAAAGTCAATTGAAAAAGGAGATGGCCCTATCGAAGAAGACAGACGTGCGTCAGATGTGGCAGAAGATACCGTTCGCGCTTGCATTTAAGGTGTACCTGTTTAATTACACAAACCCCGAAGAAGTCCAGAAAGGTGGGGTGCCAATCGTAAAGGAAATTGGACCTTATTATTTCGAGGAATGGAAGGAGAAGGTGGAAATAGAAGACCATGAGGAGAACGACACAATCACATATAAAAAGTTGGATACTTTCTACTTTCGACCTGACCTCTCCGGTCCCGGCTTGACAGGGGAAGAGCATATTGTGATGCCTCATATTTTCATTATATCTTCAACAACAATAGTGAGCAGAGACAAGCCAAGTATGCTGAATCTGATTGGTAAAGCAATAAACGGAATCTTCGACGGTCCAACGGATGTATTTGCTAGGACCAAAGCGTTGGATATATTGTTCCGCGGTATAGTCATCAACTGCGCACGAACGGAATTTGCCCCCAAAGCGGTTTGCACTGCACTCAAAAAGGAAGCCAGTTTAATTGCGGAGCCGAACAACCAGTTCCGGTTCTCGCTATTCGGGATGCGGAATGCTACAGCGGATCAGCACGTGGTCACAGTCAAACGCGGCATCAAAAATGTAATGGACGTTGGACAAGTGGTCGCTTTGGATGGTAAACCAAAATTAGATATCTGGAGAGATTCGTGTAATGAGTTCCAGGGCACAGACGGCACAATCTTCCCGCCTTTCTTGACTGCATCTGATAGACTTCAGTCATATTCTTCAGATTTGTGCAGGTCATTTAAGCCGTGGTATCAAAGGACAACGTCTTACCGCGGTATCAAAACACACCGGTACGTCGCCAACATTGGAGACTTCGCTAACGATCCAGAATTGCAGTGTTTCTGTGATACACCCGACCAATGTCCGAAGAAAGGTCTCATGGACTTGATGAAGTGCATGAACGCTCCGCTATTCGCATCGATGCCCCATTTTTTGGATTCTGATCCTGAGCTACTACAGAACGTGAAGGGTTTAAGCCCAGATGTTAACGAACATGGAATCGAAATTGATTTCGAACCGATATCAGGAACTCCGATGGTGGCCAAACAGCGCATACAATTCAACATTCGATTGCTCAAGACGGACAAACTTGAGCTTTTCAAAGATTTACCGGACACGATAGTTCCTTTGTTTTGGATAGAAGAGGGATTAGCCCTCAATAAGACATTCGTGAAAATGTTGAAGCATCAACTCTTCATTCCGATGAGGGTTGTGAGCGTCGTCCGCTGGTTGCTACTCTCGTTCGGACTGCTCGGTGTGATCGGAAGCCTGGCTTTCCACTTCAAAGACCGAGTAATAAAGTTGGCCGTGAGGCAGGATTCAGGAACGGCGAAAGTGAACCCTGAGGAGGAGCATAAGGATATCAGTGTCATTGGTCAGATTCAGGAACCAGCGAAAATTGACATTTAA

> PflaSNMP2

ATGTTCGGTAAAAAGAGTTCTGTTATTTTTTGTGCGTCACTAGTATTTTTGGTGATATCGATAATACTGGCCACATGGGGAATACCGAAGATAGTGAAGAGGCAAATCCAGAATAATGTGCAGATAGAAAACTCGTCGAAAATGTTCGAACGTTGGCGCGTATTGCCAATGCCCTTAACATTTAAGGTGCATCTATTCAACGTGACCAACACAGACGACGTCAACCATGGTTACAAACCAAAAGTGAAAGAAATTGGACCGTATGTTTATAAGGAATATCGCGAAAAACTTATTTTAGGATATGGAGAAAATGATACAATAAAGTACATGTTGAAGAAAAGGTATATATTCGACCAGGAAGCGTCTGGTGGACTGAAAGAAGACGATCTTGTAACTGTTATACATTATCCTTACATGGCTTCTATTCTGACCGTGCTGGAAATGTCACCAGCTTTCGCATCGGTGATAAATAACGCCTTGAATACACTGTTTGGGGGAGTGTCGCAACCATTCCTAACGATAACCGTAAAGGAGCTATTGTTTGACGGAGTCTATCTCAATTGTACGGGTGGACAACAGAGTCTGGGTCTCGTATGCGGCAAGATCAAGTCAGAGAAACCTGCCACCATGCGCCCAGCAGAAGATGGTACCGGATATTACTTCTCAATGTTCAACCATCTGAACAGCACTGAAACCGGCCCATACGAAATGGTACGCGGAACAGAGAATTTGTACGACTTGGGGCACATAGTGTCGTACAGGAACAAAACTGTGATAGAGACGTGGGGGAACCCCTACTGTGGGCAATTGAACGGAACGGACTCCACGATCTTTGCGCCGGTAGACGGATCTAATGCACCAGAAAAACTATATACGTTTGAACCAGACTTATGCAGATCCCTGTACGCGAGTCTGGTCGGCGAACGGACCCTCTTCAATATGTCAGCATATTATTACGAAATCTCCGAGACAGTTCTGGCATCGAAGAGCGCAAATCCGAATAACAAATGTTATTGCAAAAAAAATTGGAGCGCTAAACATGATGGTTGTCTCCTAATGGGGGTTCTAAATCTGCTGCCGTGTCAAGGCGCCCATGCTATAGCATCTCTTCCACATTTCTATCTGGCTTCAGAGGAGCTCCTGGAGTACATCATCAGCGGTATCGAACCTGACAAGGAGAAGCACAACACCTATTTGTACTTAGAACCGGTGACTGGTGTGGTCCTGAAAGGGCTGAAACGTCTTCAGTTCAACATAGAATTACGCAAAGTTGAATCCGTTCCGCAGTTGGCAAAAGTGCCAACTGGTTTGTTCCCATTGTTGTGGATAGAGGAAGGTGCTGAACTACCACCATCGTTACAAGACGAGTTGCGGGACTCGCACACGATGCTGGGTTATGTCAGCGTGTTCCGCTGGACTCTATTGGCGCTTGCAATCGTTGGCGCAGTGGCTGGTGCCGTCATCTTGGCGCGCGCCAAGTCACTCCTCATATGGCCGCAGAATAACTCCGTCAATTTTGTTTTACAACCGGGCATGCAGAGTAATAGCAATAAAATTTATTAG
