# Supplementary material for: Identification of Chemosensory Genes, Including Candidate Pheromone Receptors, in Phauda flammans (Walker) (Lepidoptera: Phaudidae) Through Transcriptomic Analyses
Source: Front Physiol. 2022 Jul 1;13:907694. doi: 10.3389/fphys.2022.907694 (PMC9283972; doi:10.3389/fphys.2022.907694)
Supplement: Supplementary file 2 [file Presentation1.PPTX]

## Slide 1
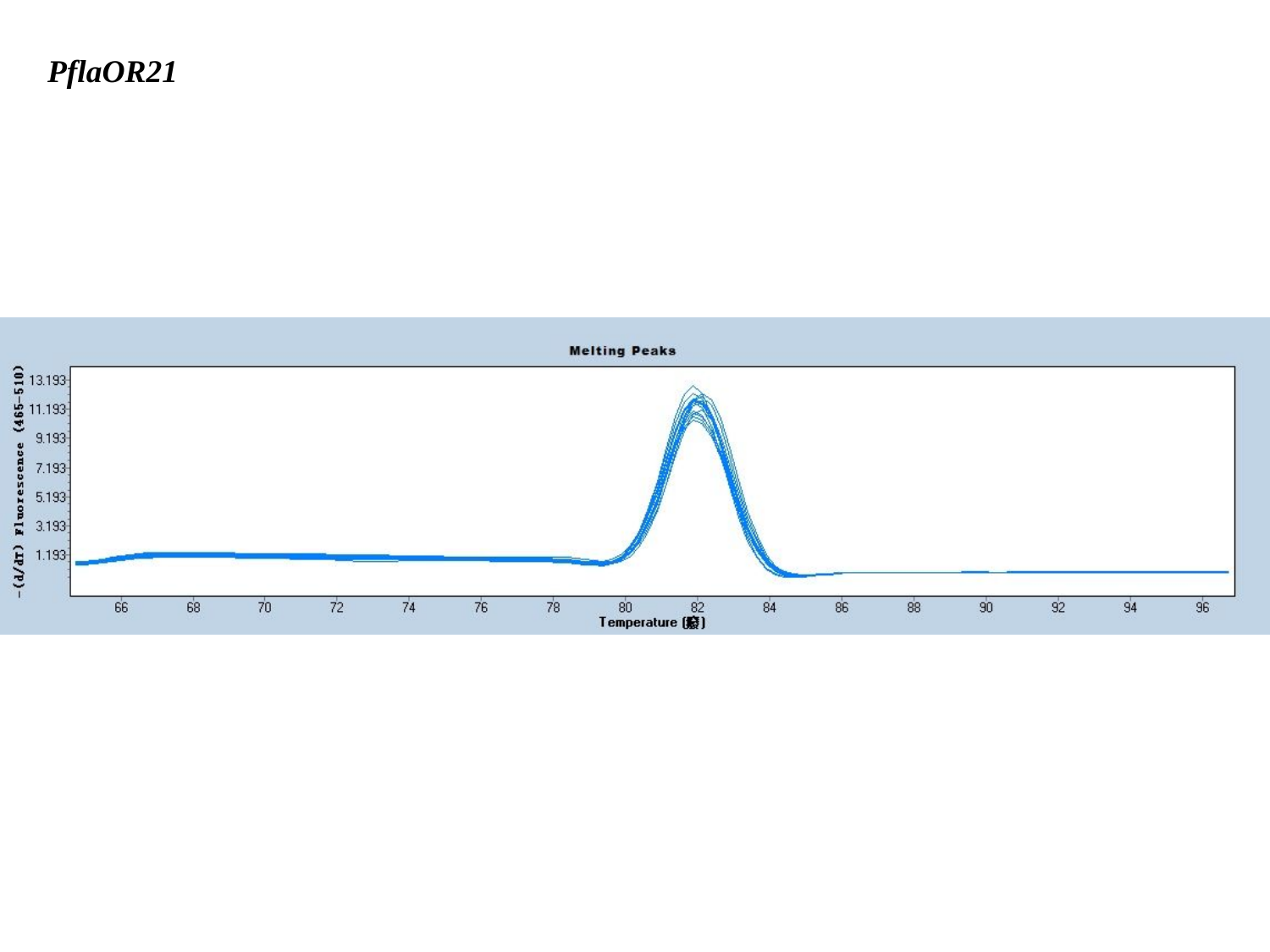

PflaOR21

## Slide 2
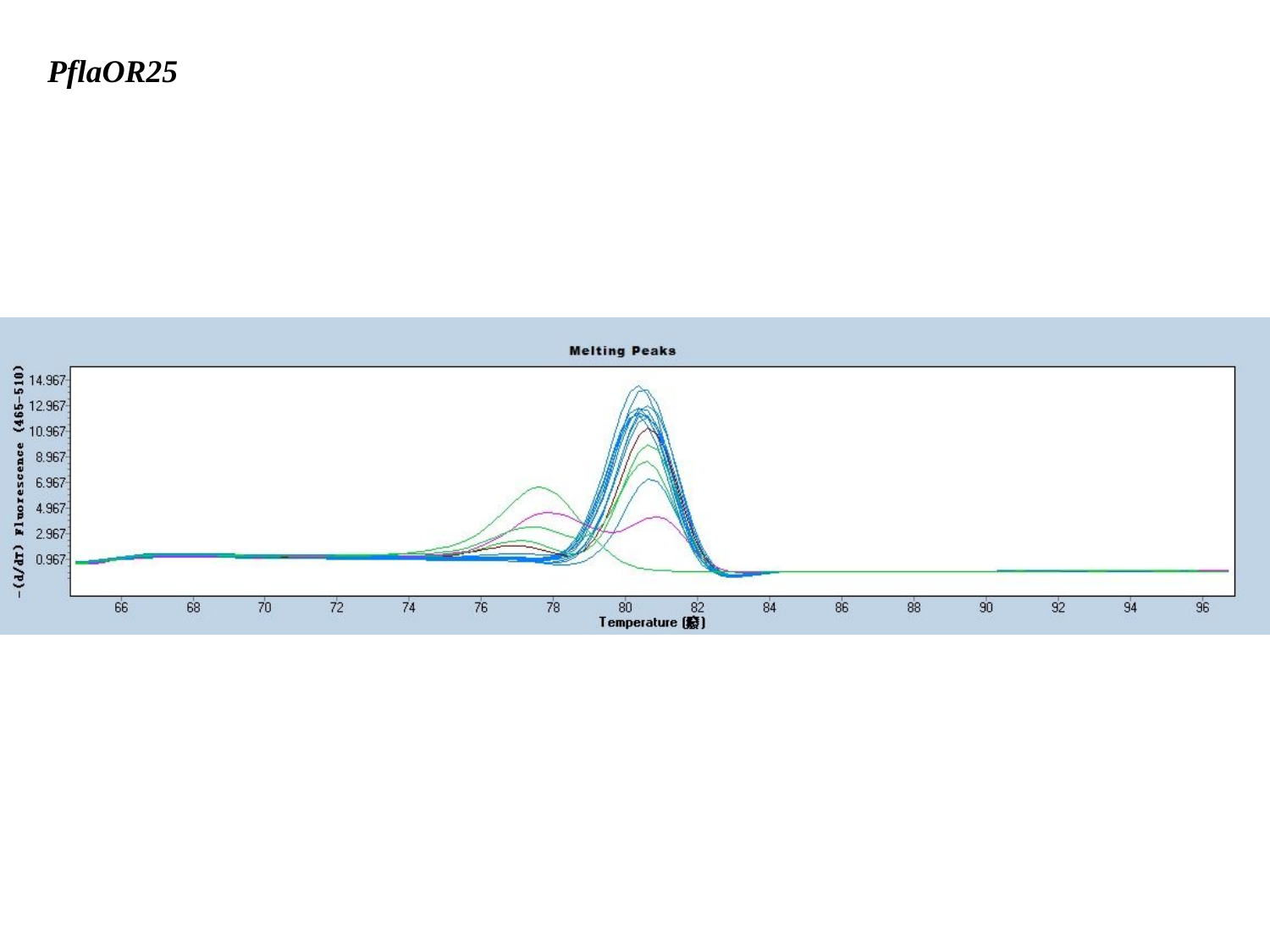

PflaOR25

## Slide 3
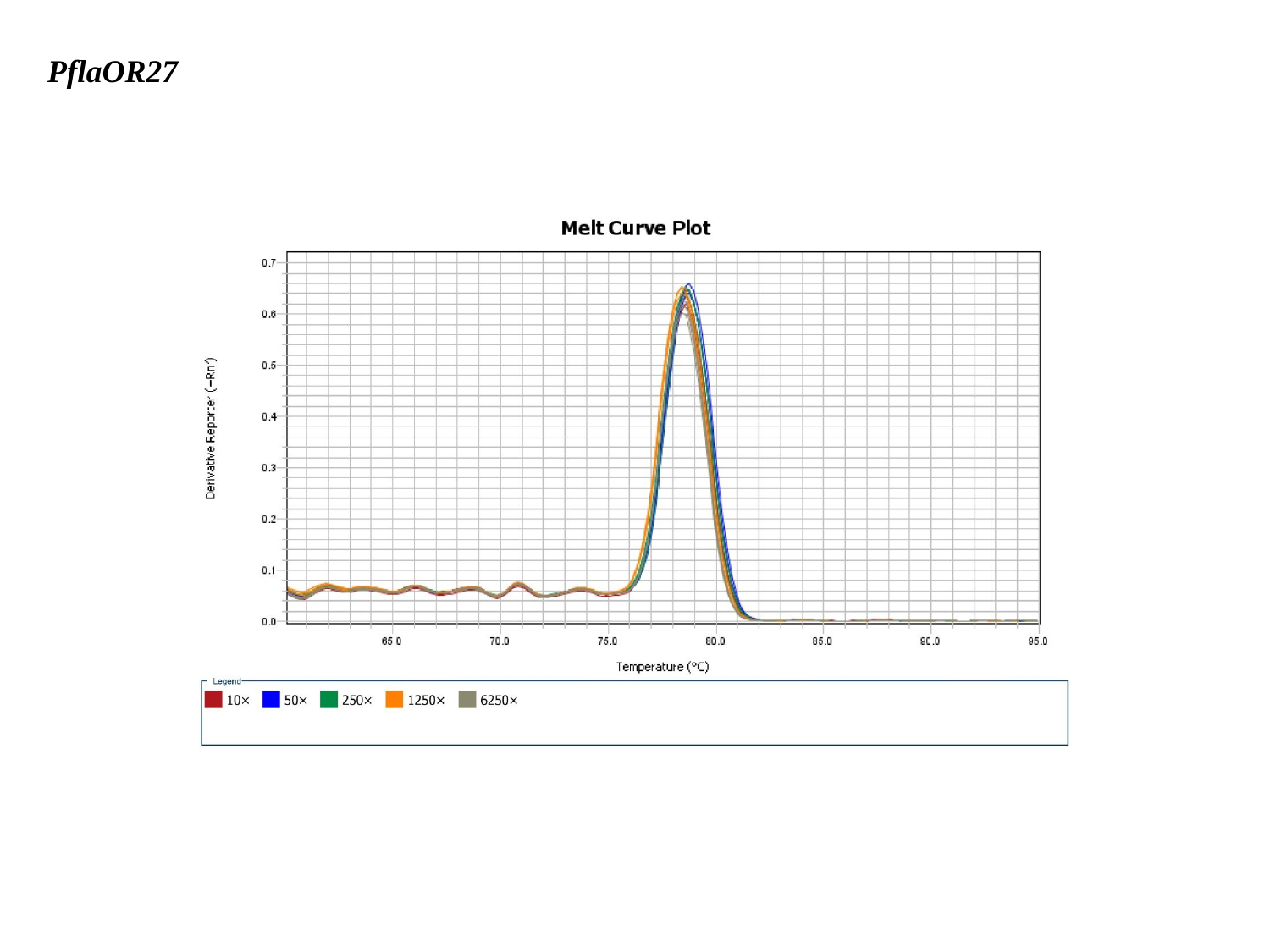

PflaOR27

## Slide 4
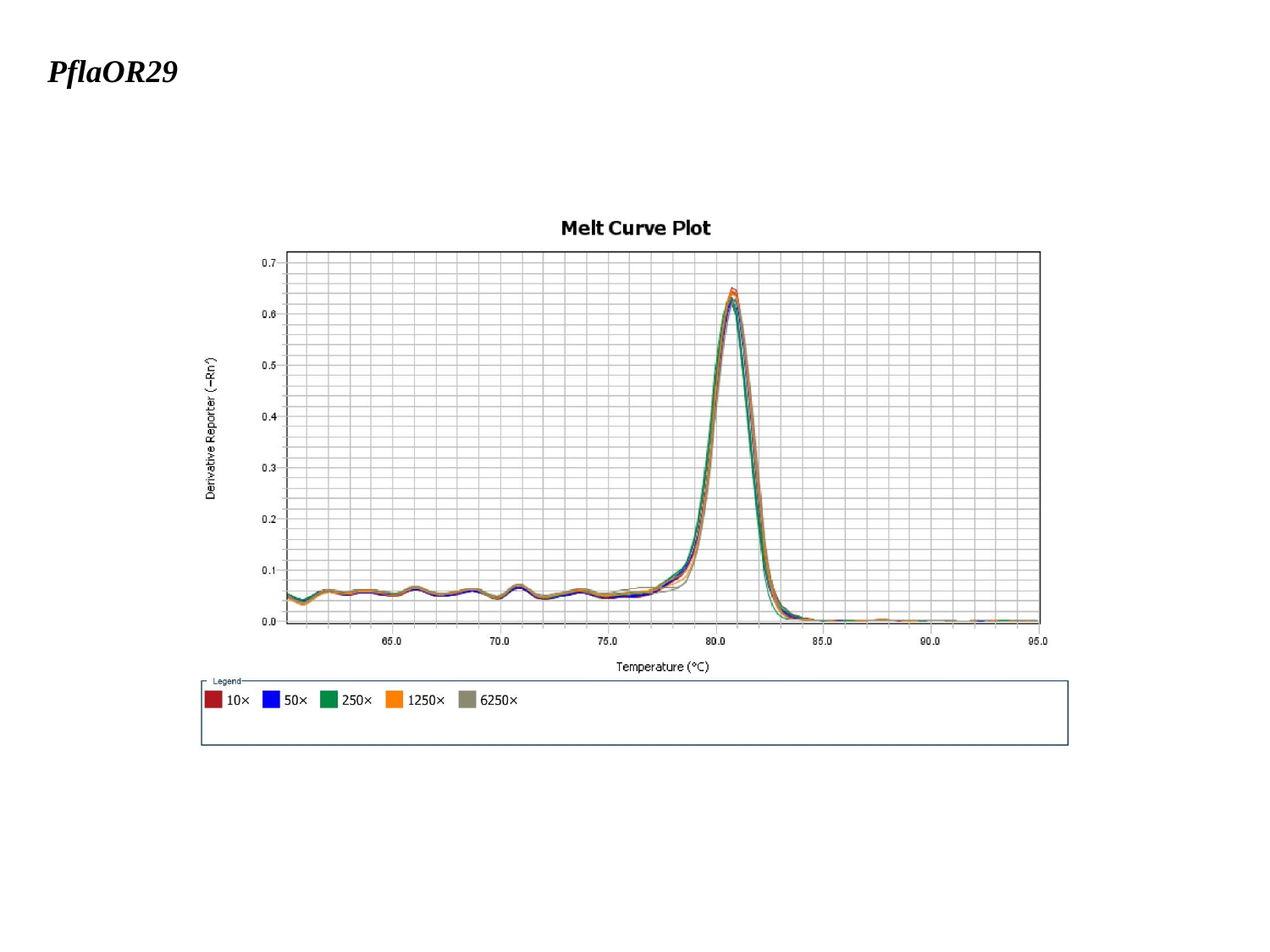

PflaOR29

## Slide 5
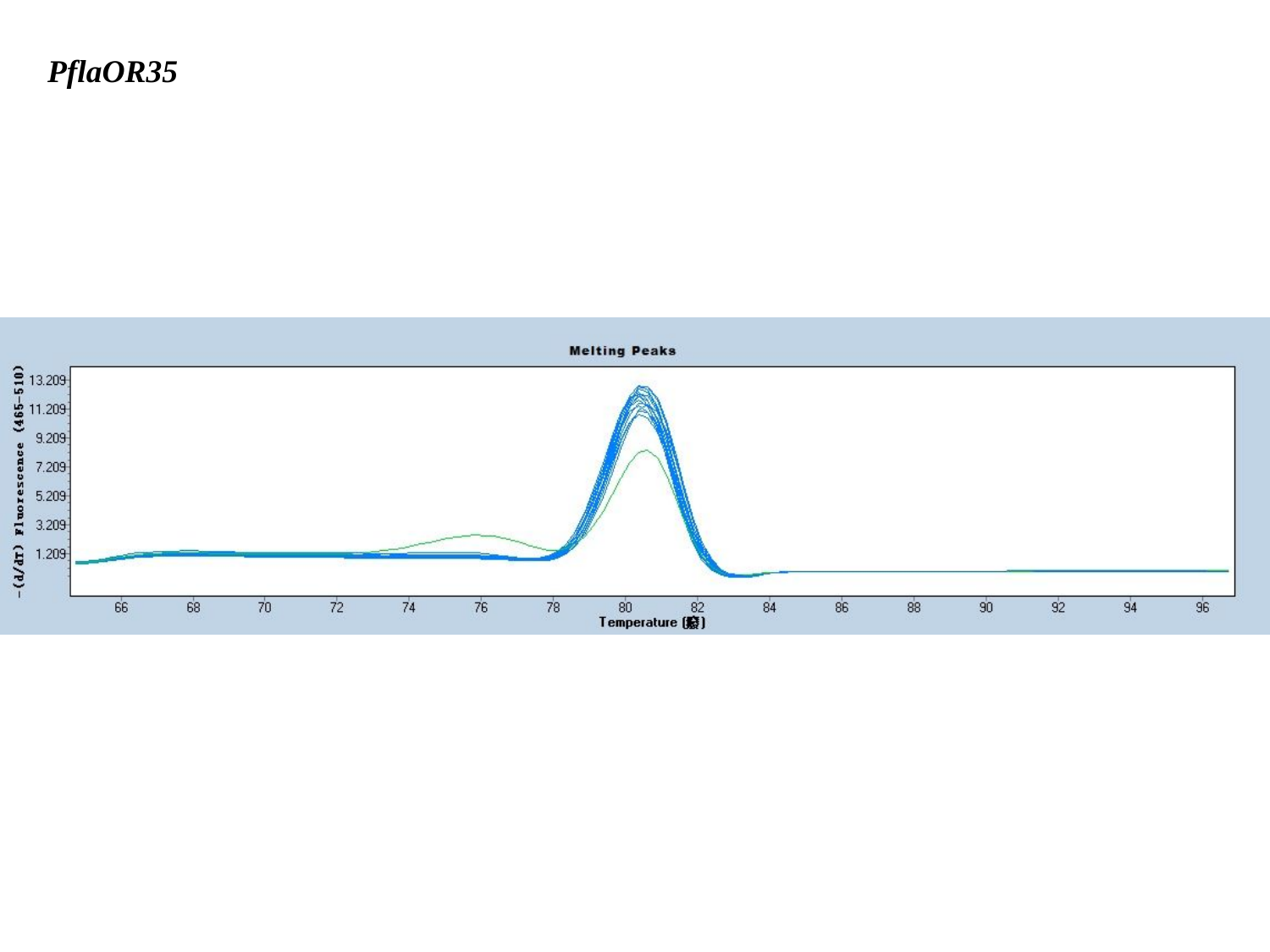

PflaOR35

## Slide 6
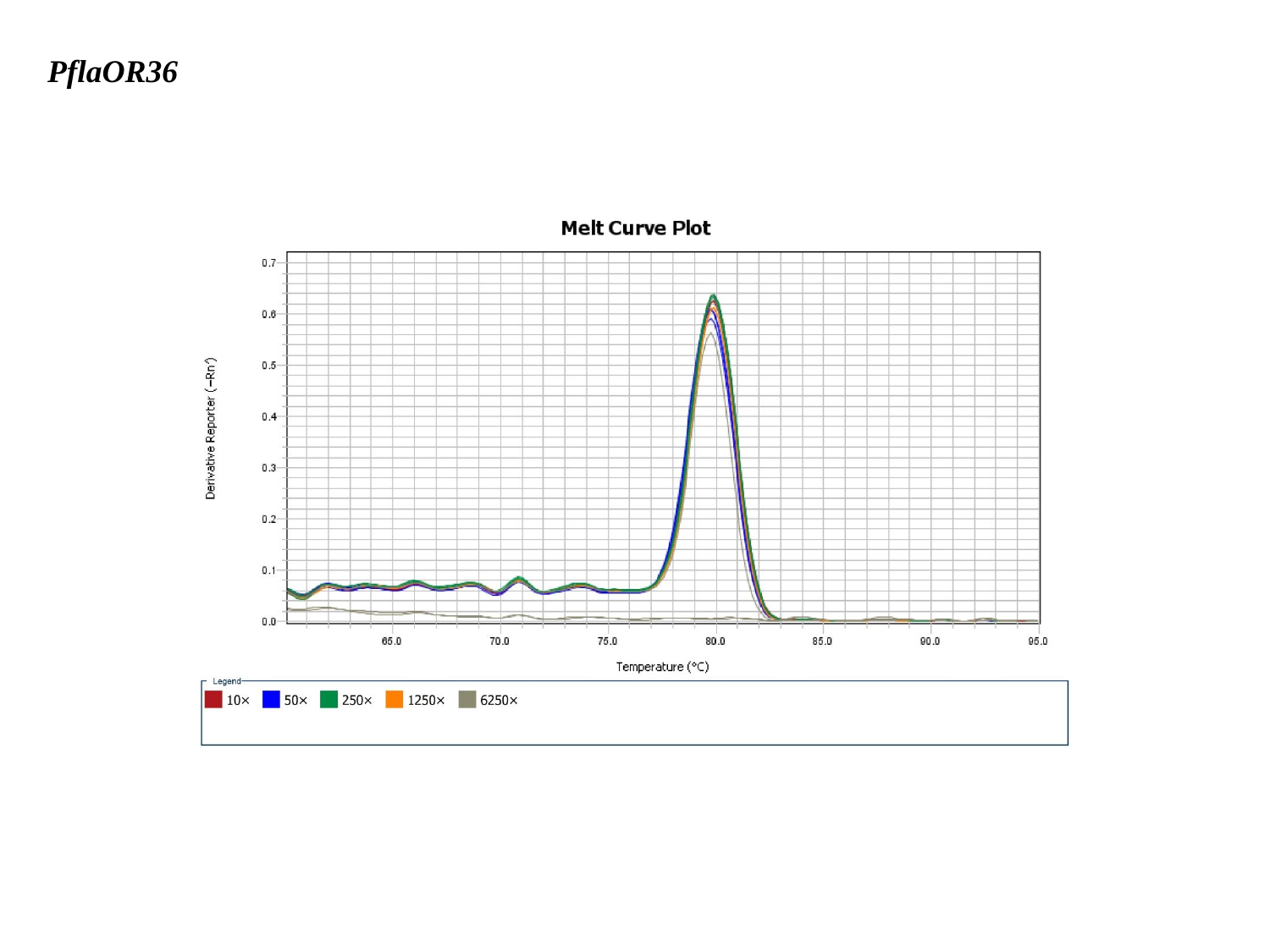

PflaOR36

## Slide 7
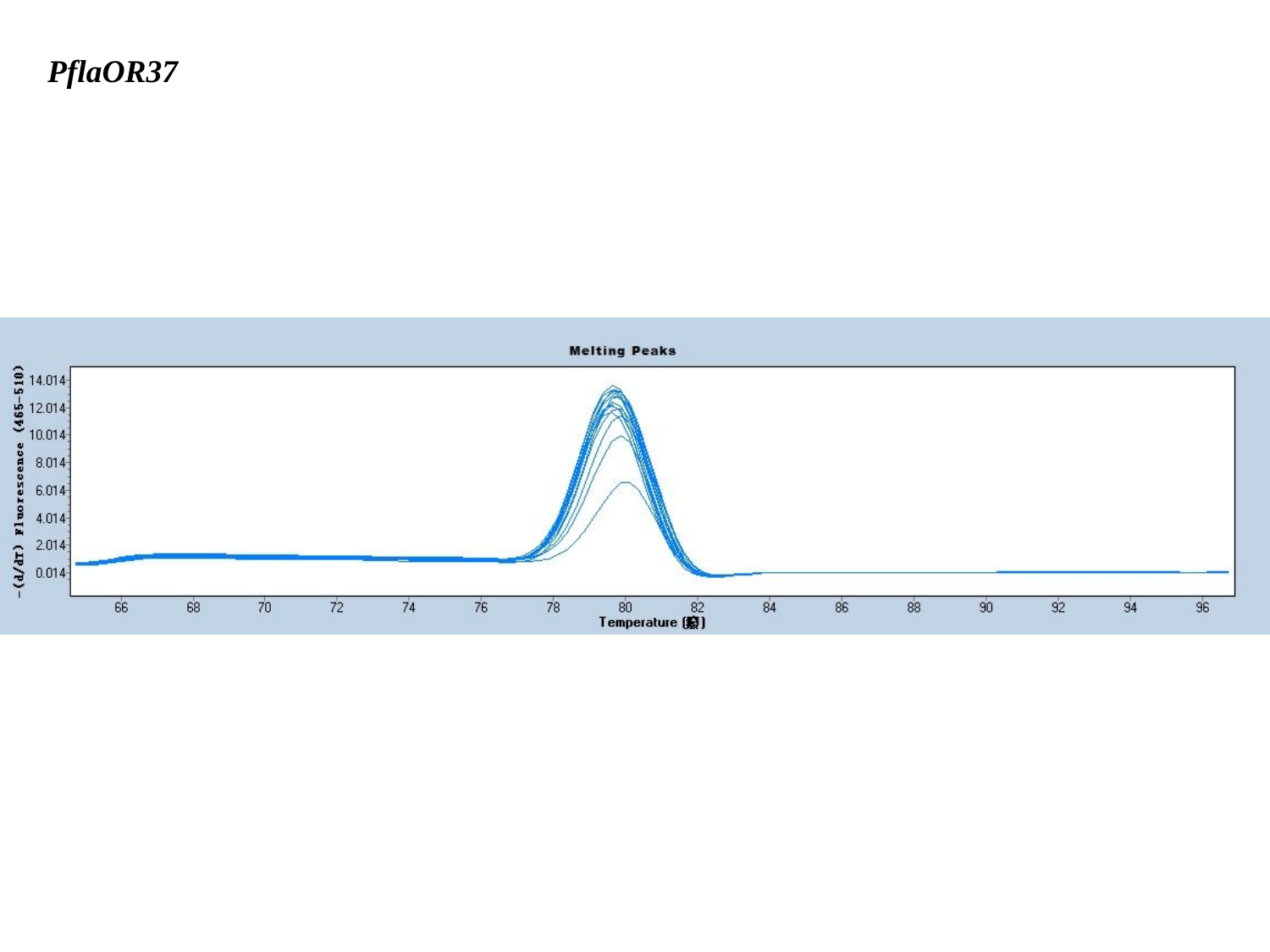

PflaOR37

## Slide 8
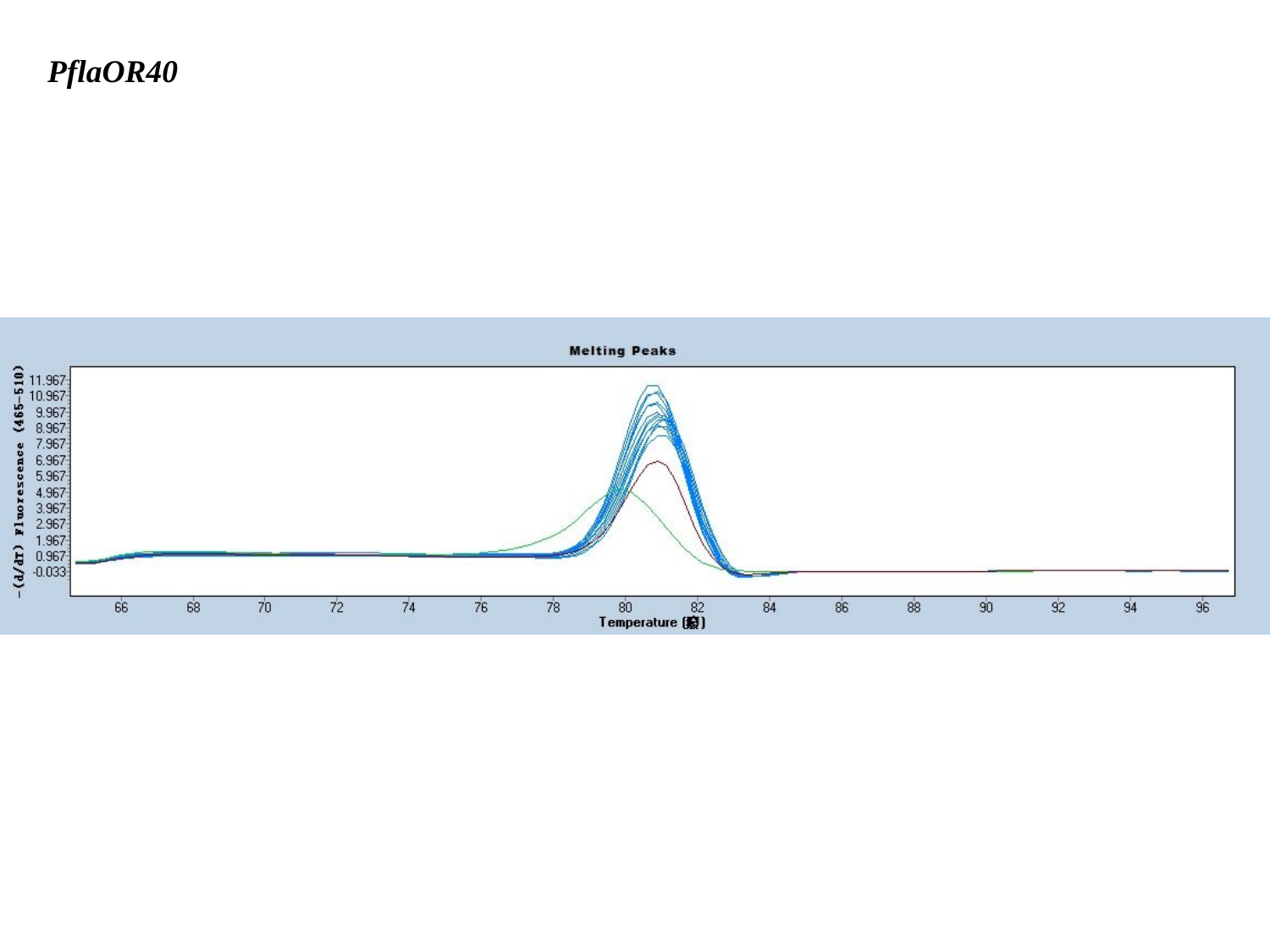

PflaOR40

## Slide 9
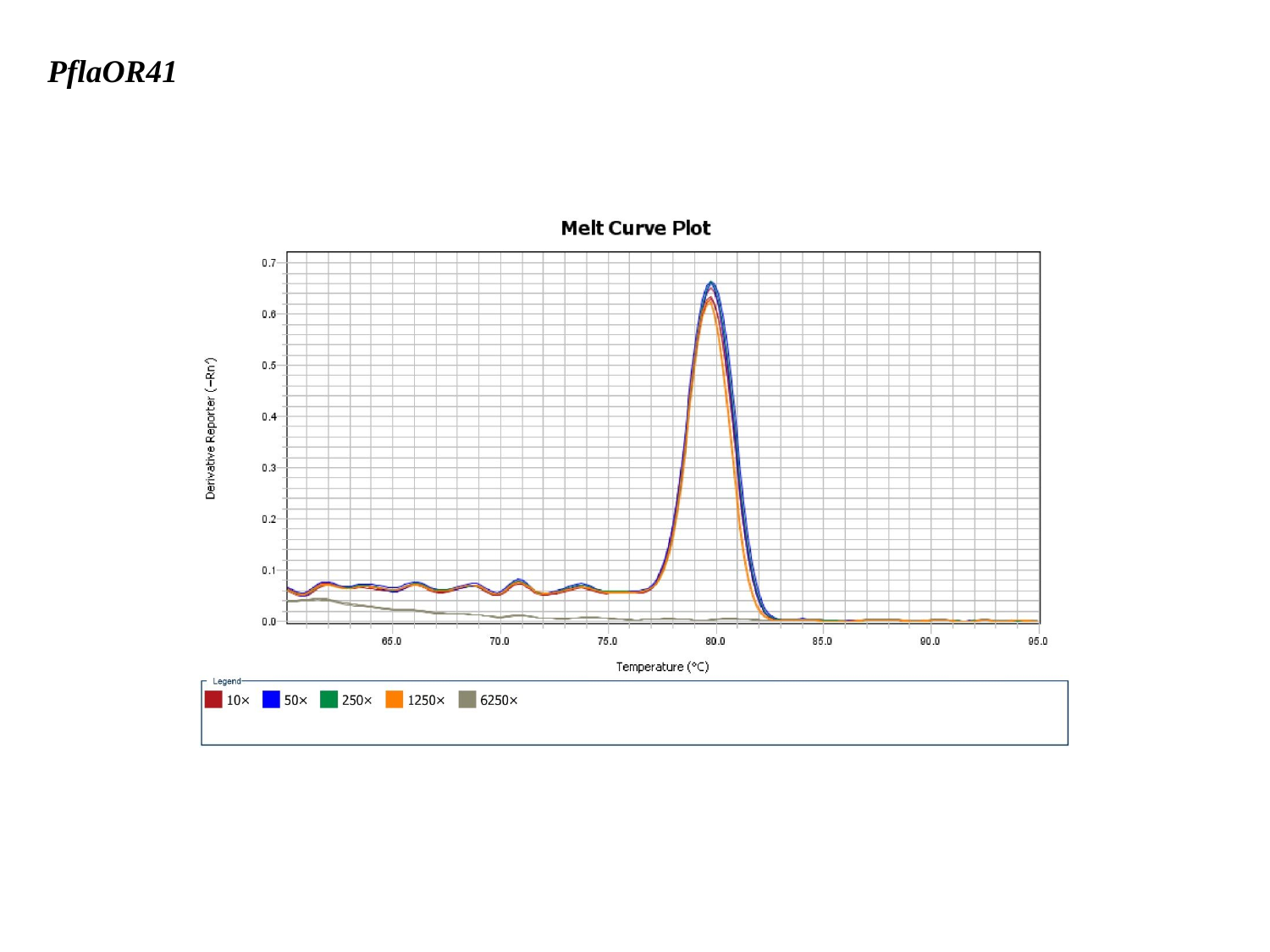

PflaOR41

## Slide 10
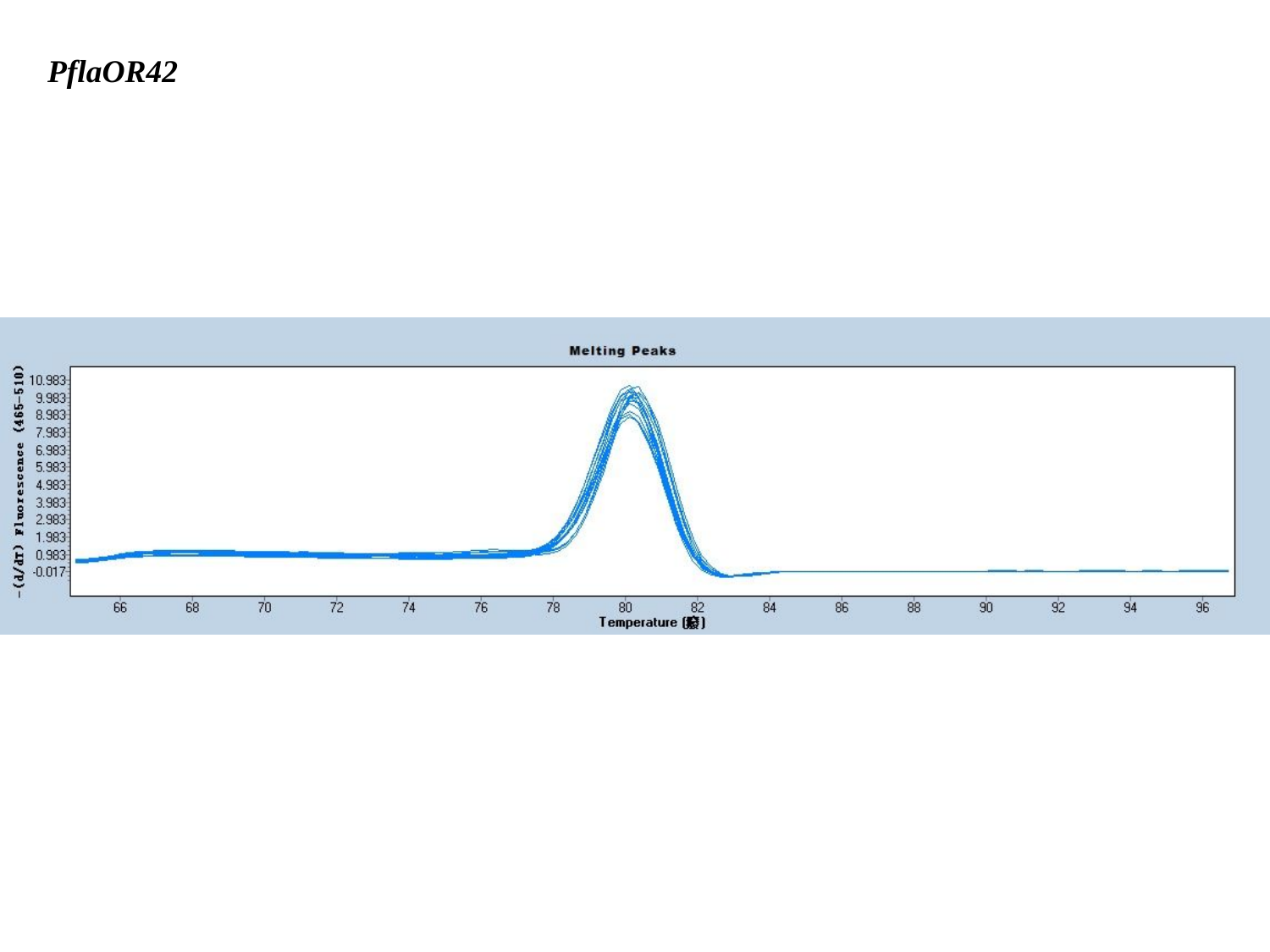

PflaOR42

## Slide 11
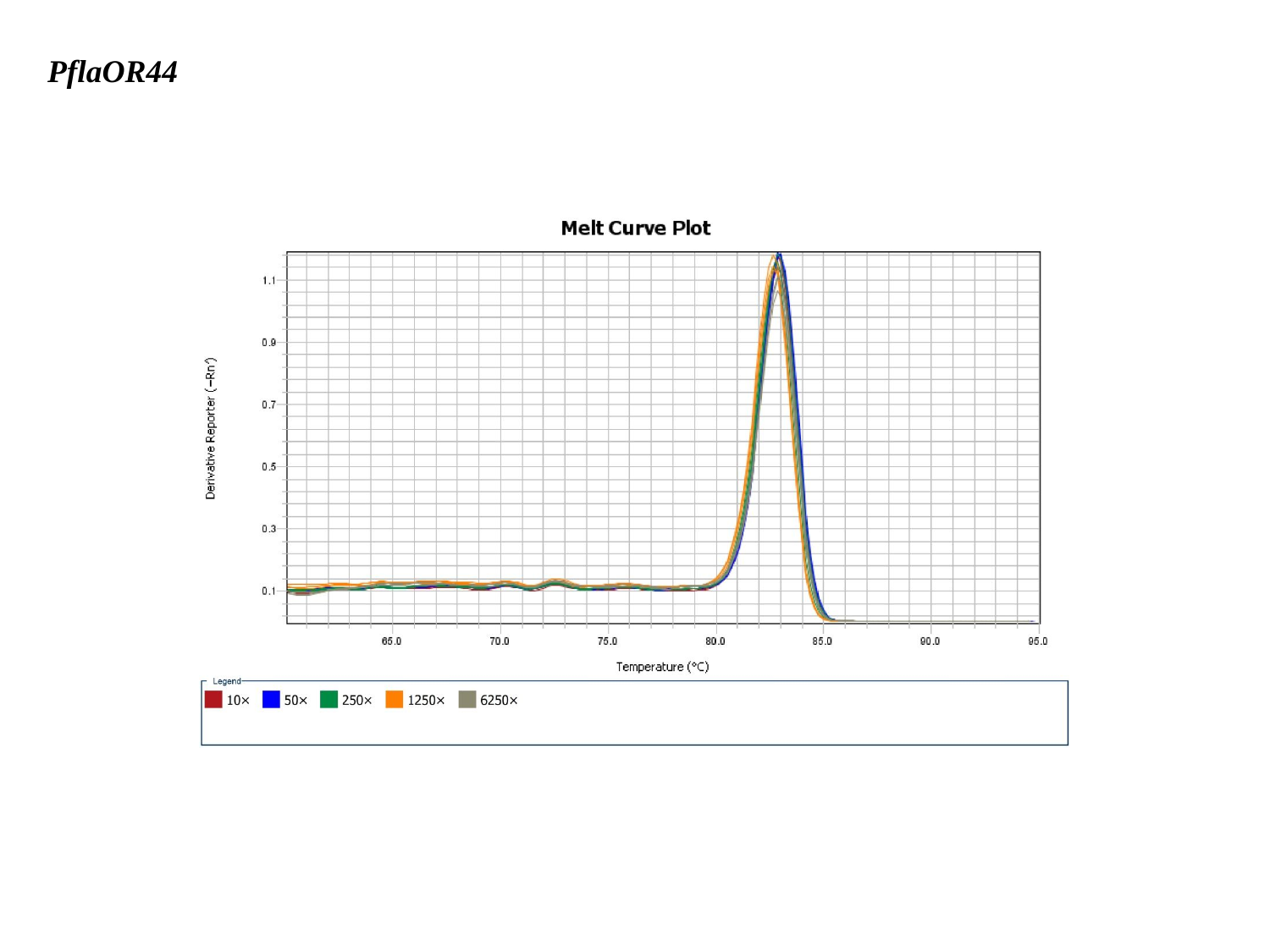

PflaOR44

## Slide 12
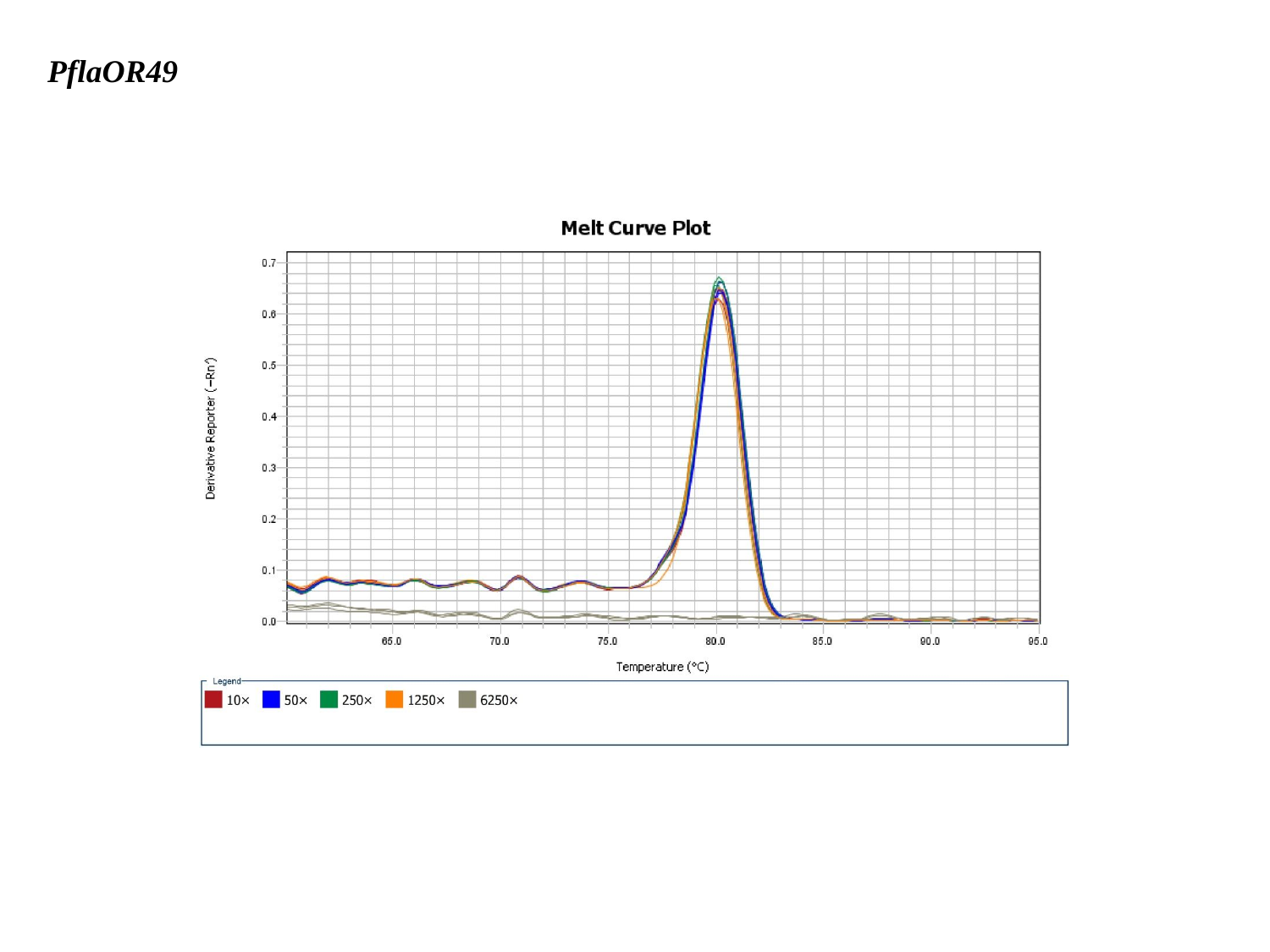

PflaOR49

## Slide 13
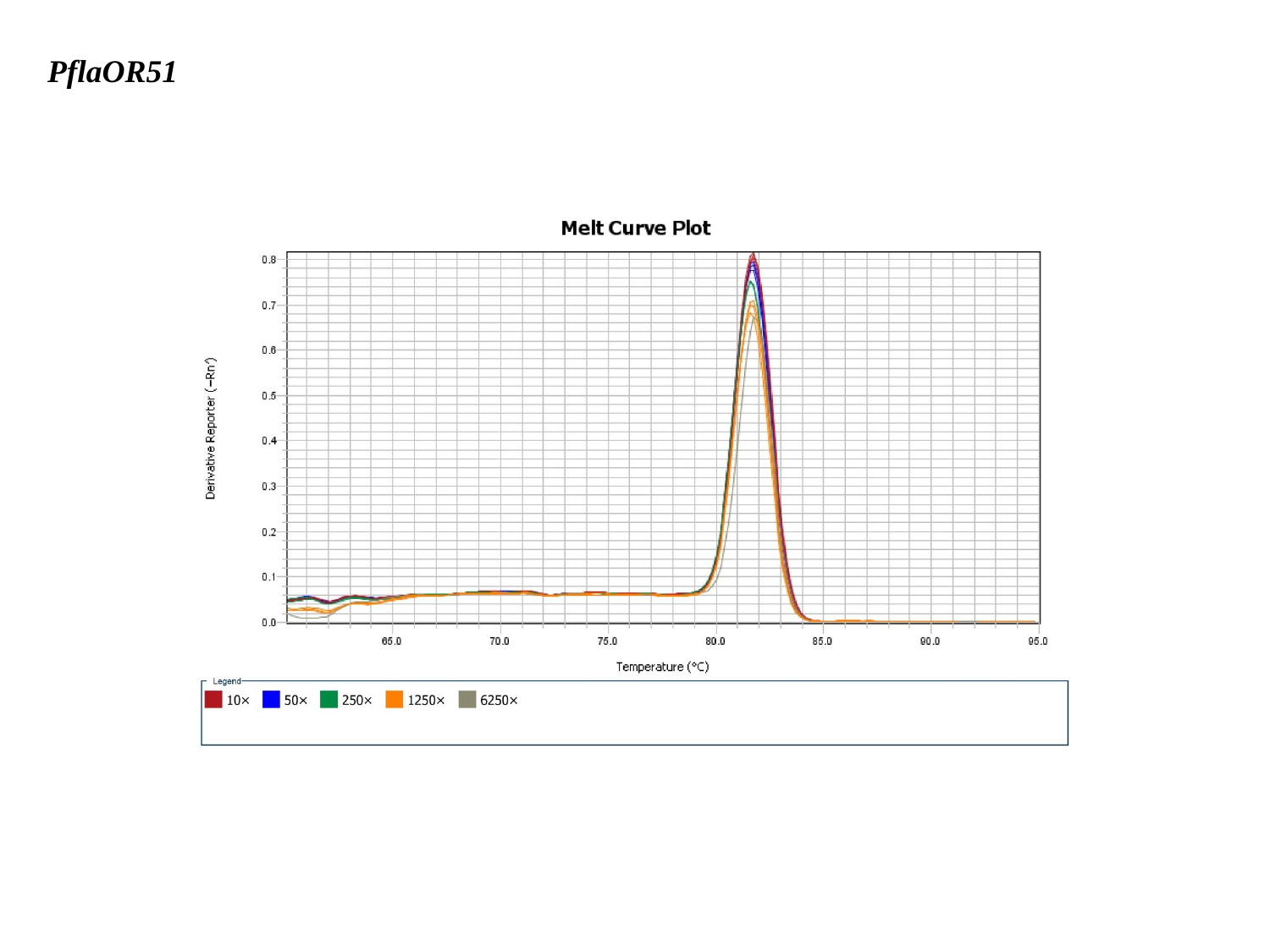

PflaOR51

## Slide 14
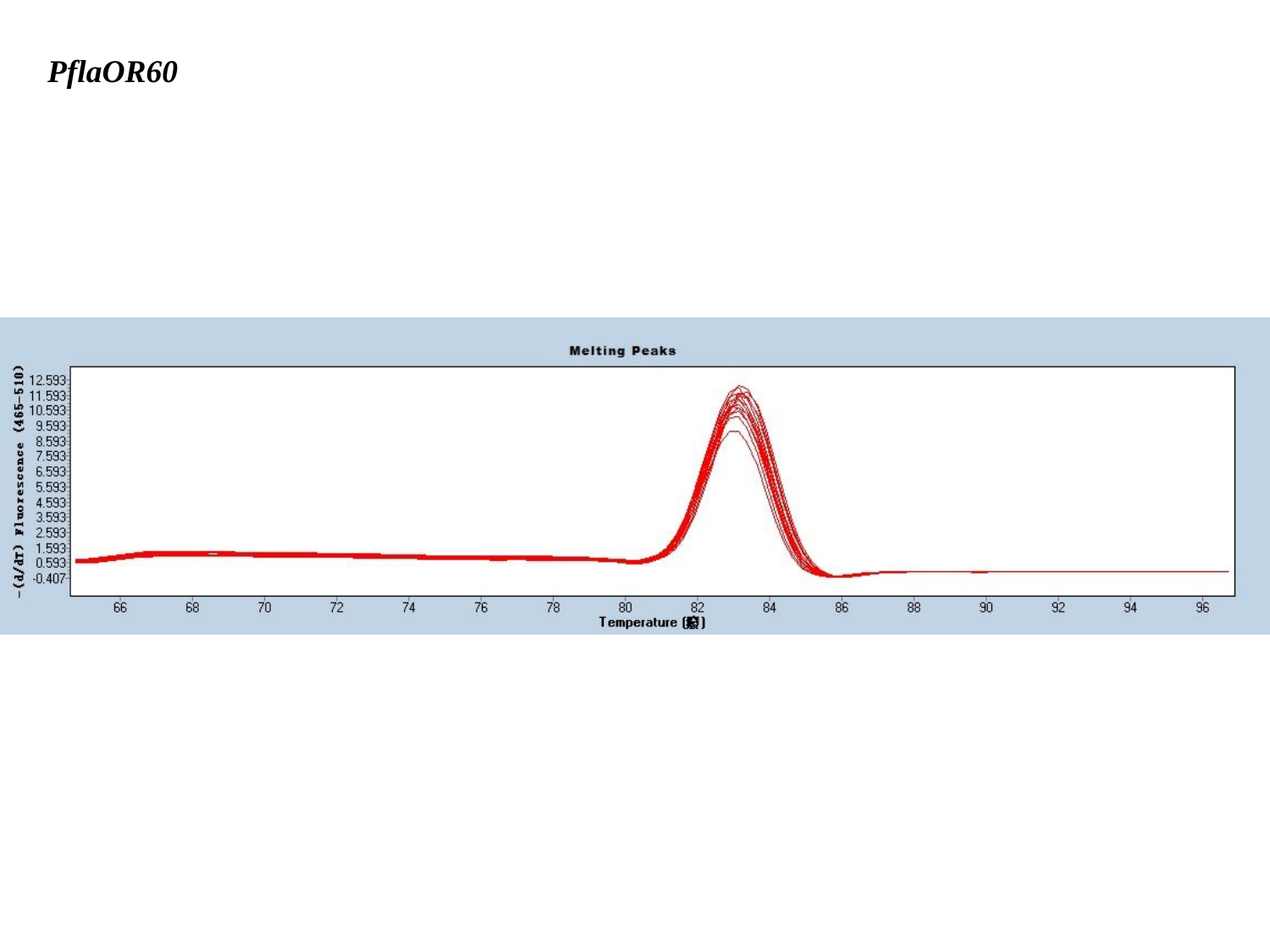

PflaOR60

## Slide 15
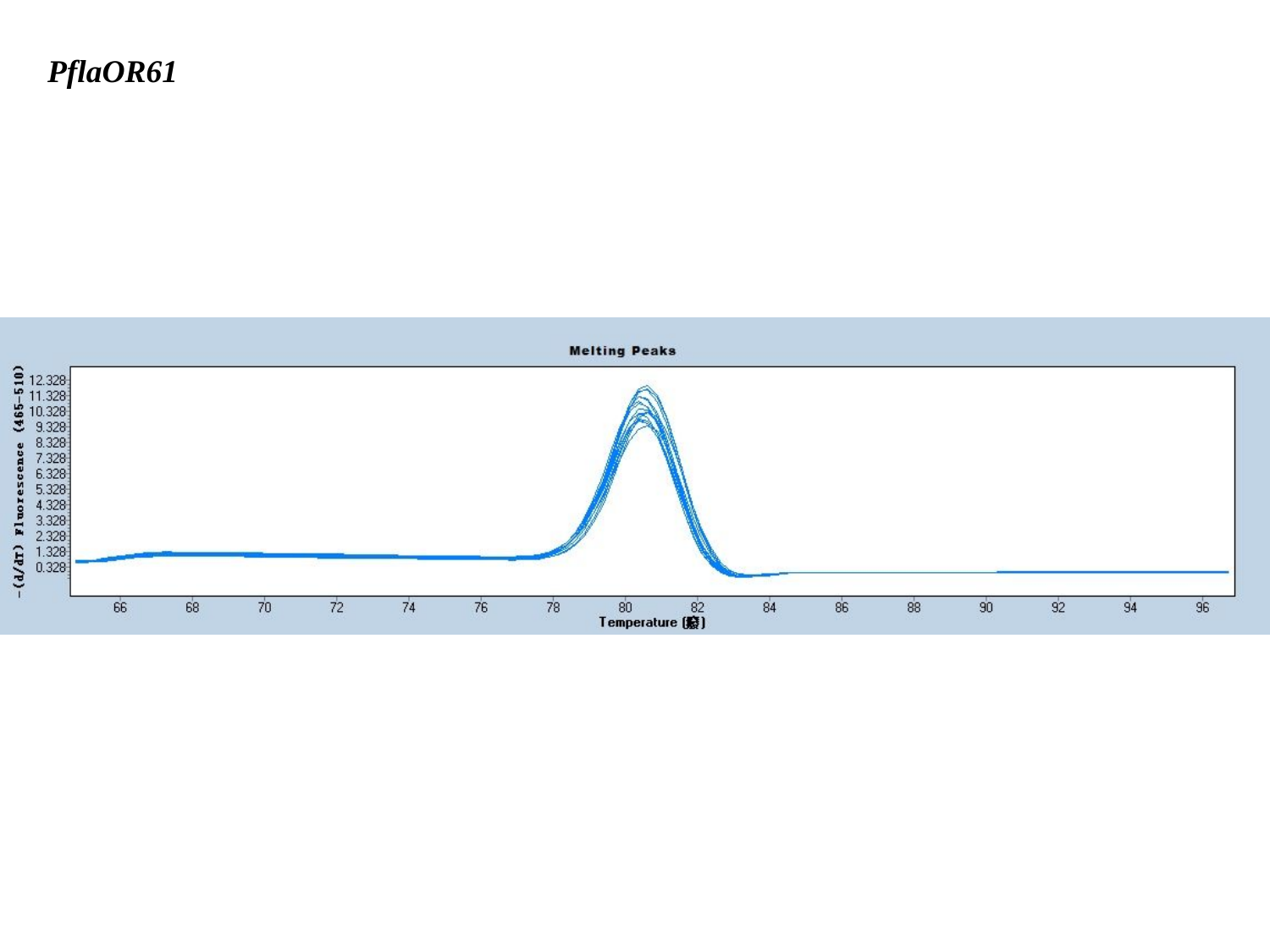

PflaOR61

## Slide 16
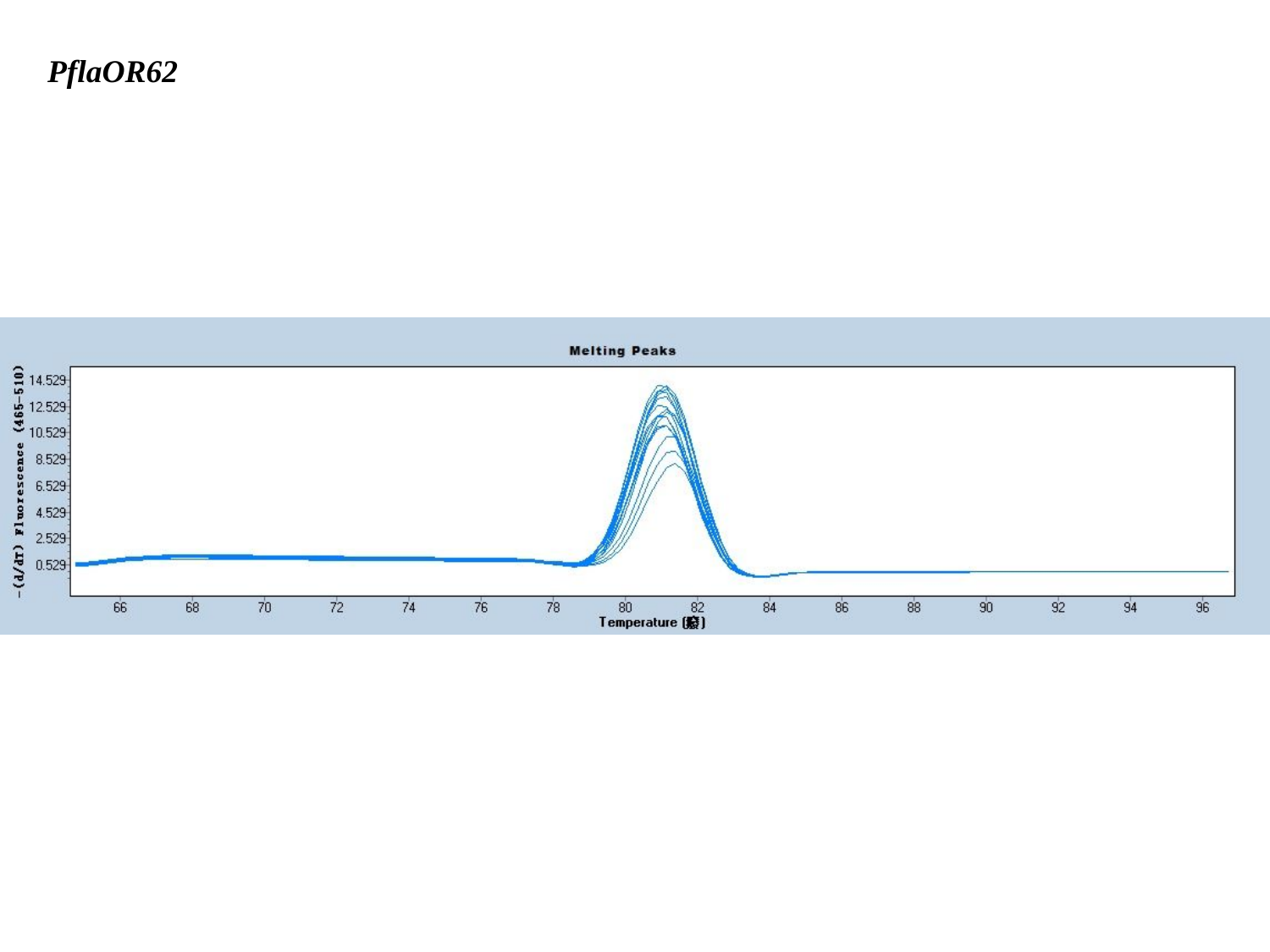

PflaOR62

## Slide 17
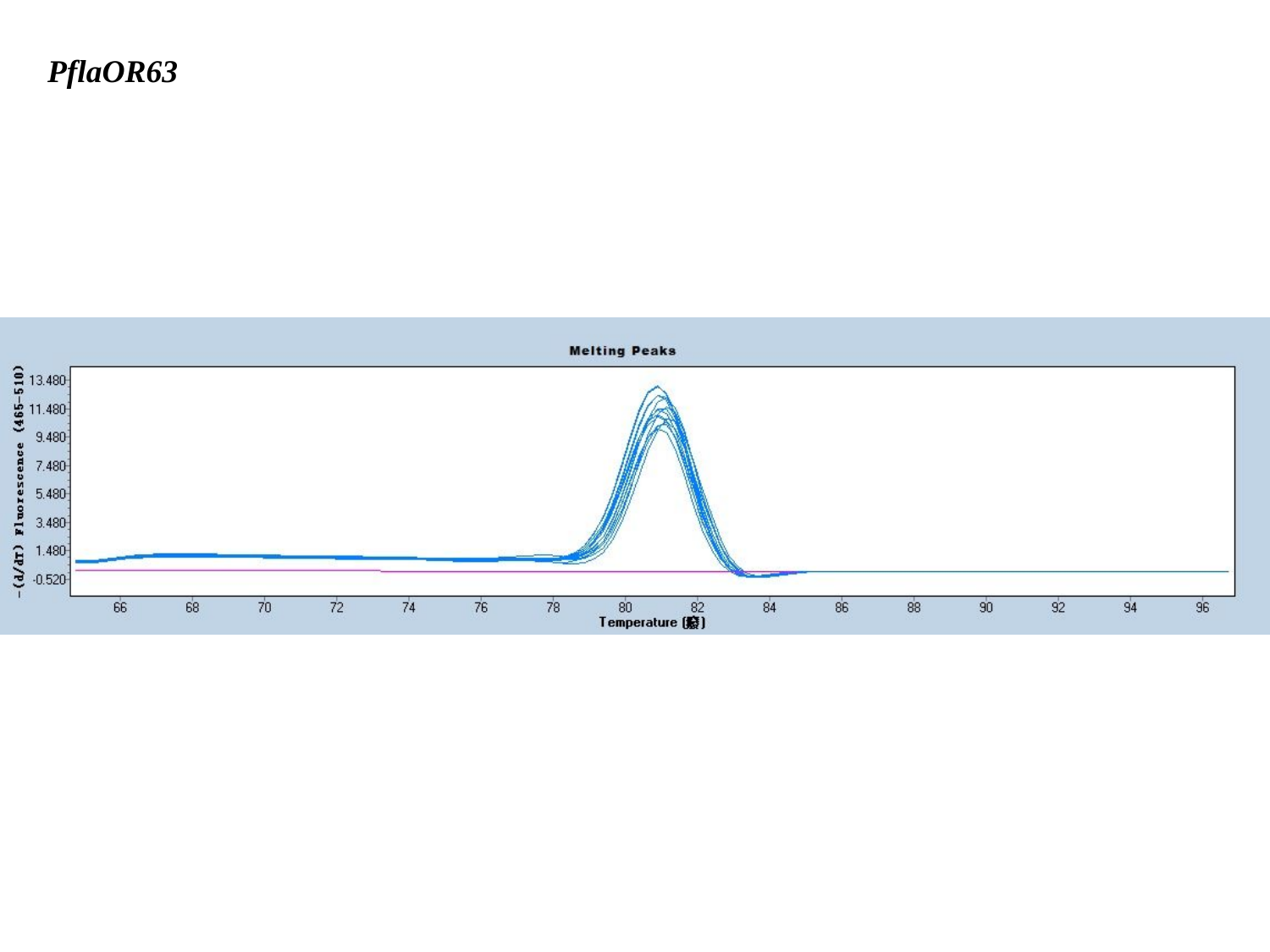

PflaOR63
