# Supplementary material for: Identification of Chemosensory Genes, Including Candidate Pheromone Receptors, in Phauda flammans (Walker) (Lepidoptera: Phaudidae) Through Transcriptomic Analyses
Source: Front Physiol. 2022 Jul 1;13:907694. doi: 10.3389/fphys.2022.907694 (PMC9283972; doi:10.3389/fphys.2022.907694)
Supplement: Supplementary file 3 [file Table2.DOCX]

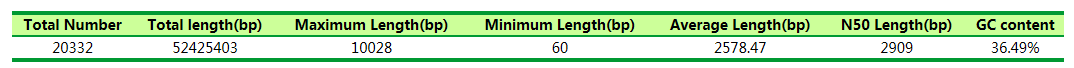


**Figure 1 |** Annotation for the antennal full-length transcriptome of *P. flammans*


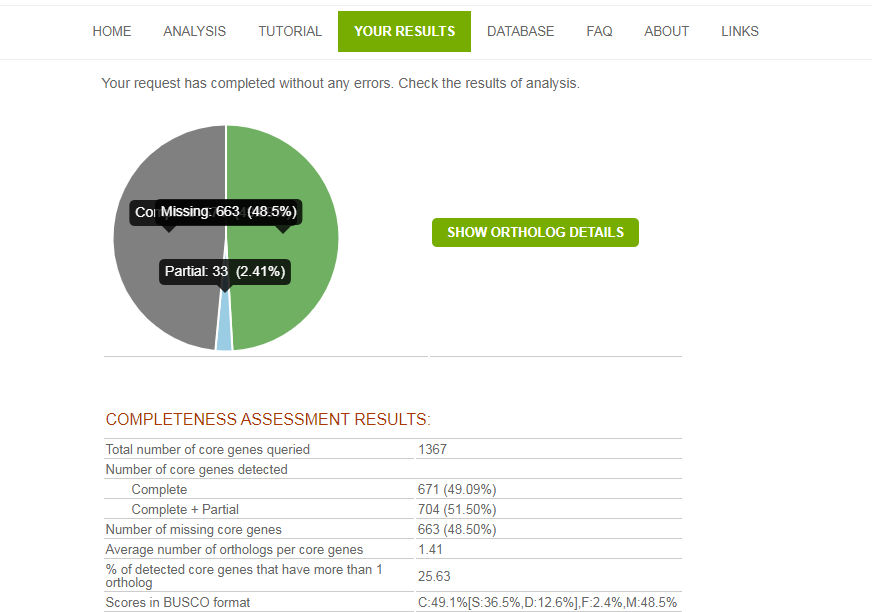


**Figure 2 |** BUSCO result for the antennal full-length transcriptome of *P. flammans*


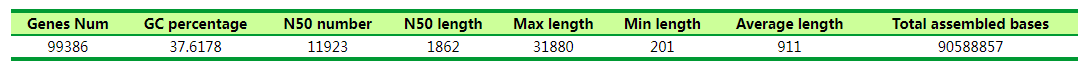


**Figure 3 |** Annotation for the unigene transcriptome of *P. flammans*


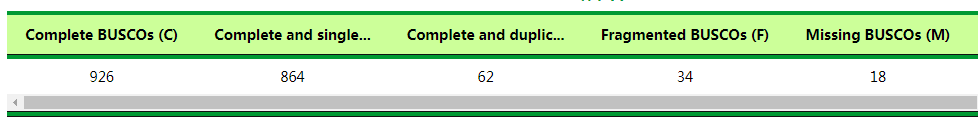


**Figure 4 |** BUSCO result for the unigene transcriptome of *P. flammans*


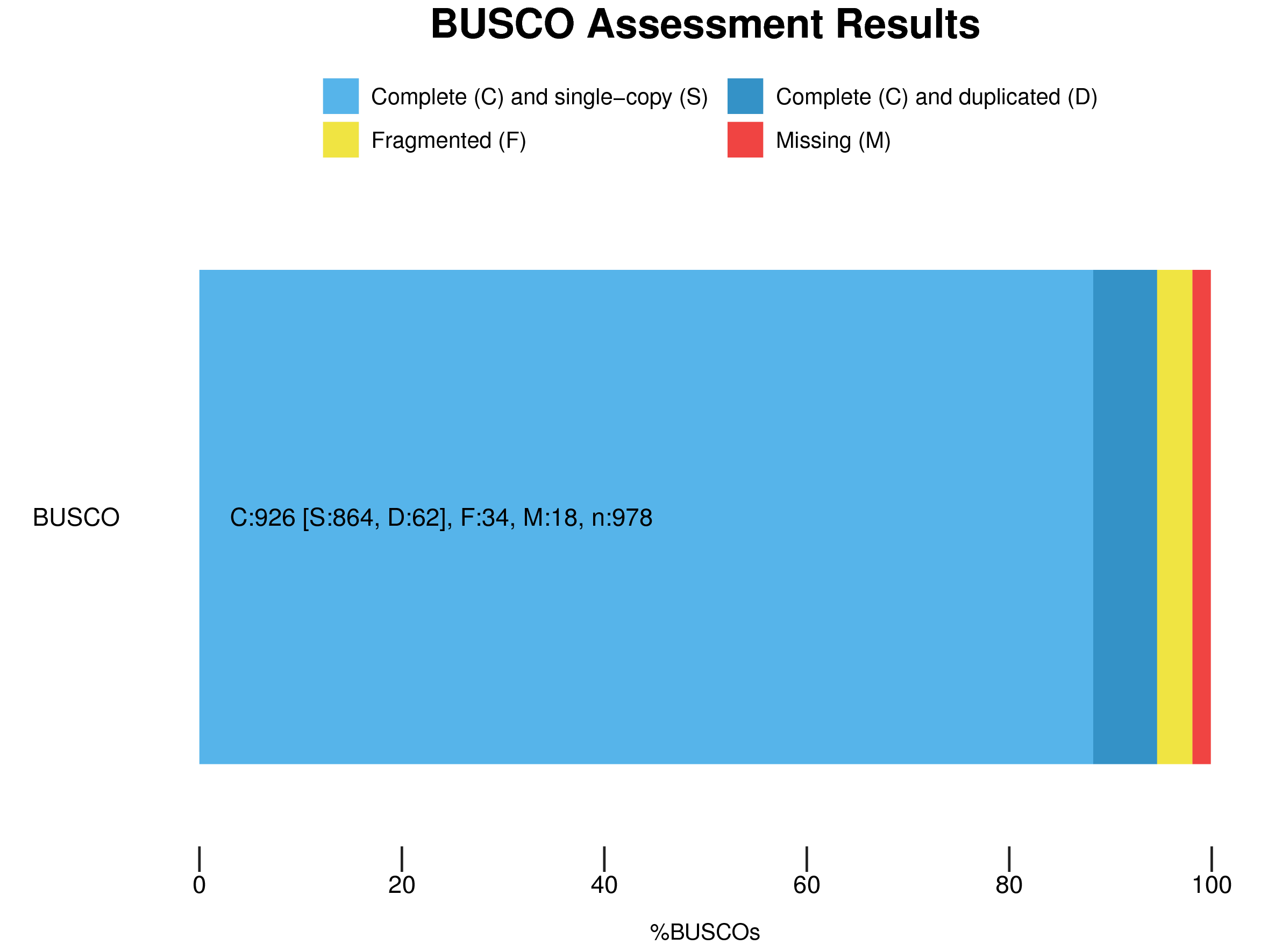
 **Figure 5 |** BUSCO result for the unigene transcriptome of *P. flammans*
