# Supplementary material for: Identification of Chemosensory Genes, Including Candidate Pheromone Receptors, in Phauda flammans (Walker) (Lepidoptera: Phaudidae) Through Transcriptomic Analyses
Source: Front Physiol. 2022 Jul 1;13:907694. doi: 10.3389/fphys.2022.907694 (PMC9283972; doi:10.3389/fphys.2022.907694)
Supplement: Supplementary file 4 [file Table3.DOCX]

**TABLE 1 |** Primers used in this study

| **Gene name** | **Primer sequences** | **Product length (bp)** |
| --- | --- | --- |
| *PflaOR21* | F: TCTAGTCGAGGGTTACCGGG | 137 |
|  | R: AGGCCAGTCGAACTTTCTCG |  |
| *PflaOR25* | F: TGAACGTGATATGTCGGCCT | 159 |
|  | R: ACATGAGTACCACCAGCACG |  |
| *PflaOR27* | F: GAGACAAACCGGCGAATGTG | 134 |
|  | R: GGCGAATGAGCAAGTACAGG |  |
| *PflaOR29* | F: TGGTGGGGCTATGAAACACC | 199 |
|  | R: GGTGGCCAGCCCAGATTTTA |  |
| *PflaOR35* | F: TACGCCGAGACAAGAAGTGG | 145 |
|  | R: ACGGCCATAGATGCAGTACC |  |
| *PflaOR36* | F: ACCGTGATGCCTTAGTGAGC | 110 |
|  | R: TTCCCCAGCAAGGTCCCTAT |  |
| *PflaOR37* | F: ACATAATGTCCGCGACAAACG | 138 |
|  | R: GACCGCAGGCGTGTAGAATA |  |
| *PflaOR40* | F: TTATGCGATGCGAGGAAGGT | 183 |
|  | R: CCGTTATTCATCTGCGCTTCC |  |
| *PflaOR41* | F: CCAGTCGCGTATTGCATGTG | 128 |
|  | R: GACGCTGGAGATATCCGTGA |  |
| *PflaOR42* | F: TCGCCGCTGTATGTTCATGT | 114 |
|  | R: GGTCTTTCTGCGTCTCCCAA |  |
| *PflaOR44* | F: CAGACATTCGCCGCATTGTT | 136 |
|  | R: TAGGCAGCAAAACTCGAGCA |  |
| *PflaOR49* | F: CATGGCCACACGTGAACAAC | 101 |
|  | R: CTCGAATGGGGTGTCGTAGT |  |
| *PflaOR51* | F: TATGTCACGTGGCCCCATTG | 196 |
|  | R: CGTACAACGCCGTTTCAGTG |  |
| *PflaOR60* | F: GTCAGTGGCTGCCTGTTACT | 104 |
|  | R: ATCTGCATCAACTGGCCGAA |  |
| *PflaOR61* | F: CATGGCCACACGTGAACAAC | 101 |
|  | R: CTCGAATGGGGTGTCGTAGT |  |
| *PflaOR62* | F: CAGACATTCGCCGCATTGTT | 136 |
|  | R: TAGGCAGCAAAACTCGAGCA |  |
| *PflaOR63* | F: TGGCCACACGTGAACAACTA | 168 |
|  | R: GGTGAACATAGGGCAAGTGGA |  |

**TABLE 2 |** Amplification efficiency and regression coefficient of qRT-PCR primers

| **Genes** | **PCR Efficiency (%)** | **Regression coefficient (R^2^)** |
| --- | --- | --- |
| *PflaOR21* | 114.89 | 0.999 |
| *PflaOR25* | 120.21 | 0.992 |
| *PflaOR27* | 92.69 | 0.997 |
| *PflaOR29* | 88.57 | 0.996 |
| *PflaOR35* | 113.32 | 0.997 |
| *PflaOR36* | 100.46 | 0.997 |
| *PflaOR37* | 104.59 | 0.991 |
| *PflaOR40* | 113.67 | 0.999 |
| *PflaOR41* | 109.27 | 0.999 |
| *PflaOR42* | 115.44 | 0.998 |
| *PflaOR44* | 103.15 | 0.994 |
| *PflaOR49* | 93.36 | 0.998 |
| *PflaOR51* | 91 | 0.998 |
| *PflaOR60* | 113.37 | 0.996 |
| *PflaOR61* | 115.52 | 0.995 |
| *PflaOR62* | 109.40 | 0.999 |
| *PflaOR63* | 112.09 | 0.998 |

**TABLE 3 |** Blastx matches for genes encoding candidate OBPs in *P. flammans*

| Gene  Name | Gene  length (bp) | ORF  (aa) | Complete  ORF | Signal  Peptide  (aa) | Cysteine  number | Best blastx match | | | | | Transcriptome  Source  (No.) |
| --- | --- | --- | --- | --- | --- | --- | --- | --- | --- | --- | --- |
|  |  |  |  |  |  | Gene description | species | Accession  number | Identity  % | E-value |  |
| PflaOBP1 | 417 | 138 | Yes | 17 | 6 | odorant binding protein | *Glyphodes pyloalis* | QIJ45759.1 | 33.09 | 1e-15 | 2 |
| PflaOBP2 | 429 | 142 | Yes | 20 | 5 | odorant-binding protein 16 | *Streltzoviella insularis* | QLI62019.1 | 26.45 | 3e-11 | 2 |
| PflaOBP3 | 366 | 121 | No | No | 6 | odorant-binding protein 15 | *Streltzoviella insularis* | QLI62018.1 | 80.17 | 2e-66 | 2 |
| PflaOBP4 | 489 | 162 | Yes | 23 | 6 | odorant binding protein | *Eogystia hippophaecolus* | AOG12873.1 | 83.33 | 3e-28 | 2 |
| PflaOBP5 | 417 | 138 | Yes | 18 | 5 | odorant-binding protein 1 | *Chilo suppressalis* | AGK24577.1 | 29.32 | 2e-11 | 2 |
| PflaOBP6 | 474 | 157 | Yes | 24 | 6 | odorant-binding protein 6 | *Bombyx mori* | XP_012547442.1 | 39.39 | 1e-21 | 2 |
| PflaOBP7 | 555 | 184 | Yes | 22 | 9 | odorant binding protein 24 | *Spodoptera exigua* | AKT26501.1 | 89.13 | 1e-119 | 2 |
| PflaOBP8 | 999 | 332 | Yes | 20 | 9 | odorant-binding protein 12 | *Streltzoviella insularis* | QLI62015.1 | 47.56 | 6e-79 | 2 |
| PflaOBP9 | 675 | 224 | Yes | 22 | 11 | odorant-binding protein 3 | *Streltzoviella insularis* | QLI62006.1 | 41.50 | 3e-42 | 2 |
| PflaOBP10 | 777 | 258 | Yes | 19 | 5 | odorant binding protein | *Athetis dissimilis* | QCF41921.1 | 57.63 | 1e-98 | 2 |
| PflaOBP11 | 420 | 139 | Yes | 18 | 8 | odorant binding protein 5 | *Argyresthia conjugella* | AFD34173.1 | 73.53 | 5e-70 | 2 |
| PflaOBP12 | 357 | 118 | No | No | 6 | odorant-binding protein 34 | *Lobesia botrana* | AXF48731.1 | 61.86 | 3e-48 | 2 |
| PflaOBP13 | 426 | 141 | No | 21 | 7 | odorant-binding protein 9 | *Galleria mellonella* | QEI46793.1 | 60.83 | 3e-49 | 2 |
| PflaOBP14 | 576 | 191 | Yes | No | 7 | odorant-binding protein 33 | *Lobesia botrana* | AXF48730.1 | 67.18 | 6e-61 | 2 |
| PflaOBP15 | 312 | 103 | No | No | 4 | odorant-binding protein 9 | *Spodoptera exigua* | AGP03455.1 | 56.45 | 4e-18 | 2 |
| PflaOBP16 | 363 | 120 | No | 25 | 3 | odorant binding protein | *Histia rhodope* | QGN01739.1 | 74.19 | 3e-28 | 3 |
| PflaOBP17 | 438 | 145 | Yes | 25 | 6 | odorant binding protein | *Eogystia hippophaecolus* | AOG12869.1 | 35.59 | 1e-16 | 3 |
| PflaOBP18 | 426 | 141 | Yes | 18 | 8 | odorant-binding protein 23 | *Streltzoviella insularis* | QLI62026.1 | 74.47 | 1e-73 | 3 |
| PflaOBP19 | 567 | 188 | Yes | 25 | 13 | odorant binding protein | *Glyphodes pyloalis* | QIJ45738.1 | 47.06 | 5e-45 | 3 |
| PflaOBP20 | 777 | 258 | Yes | 19 | 5 | odorant binding protein | *Micromelalopha troglodyta* | QNI19889.1 | 50.39 | 2e-96 |  |
| PflaOBP21 | 777 | 258 | Yes | 19 | 5 | odorant binding protein | *Athetis dissimilis* | QCF41921.1 | 57.63 | 1e-98 | 3 |
| PflaOBP22 | 459 | 152 | Yes | No | 2 | odorant binding protein | *Micromelalopha troglodyta* | QNI19889.1 | 52.63 | 8e-53 | 3 |
| PflaOBP23 | 435 | 144 | Yes | 23 | 7 | odorant binding protein | *Eogystia hippophaecolus* | AOG12873.1 | 80.65 | 5e-72 | 3 |
| PflaOBP24 | 402 | 133 | Yes | 18 | 5 | odorant binding protein | *Histia rhodope* | QGN01742.1 | 86.47 | 1e-77 | 3 |
| PflaOBP25 | 468 | 155 | Yes | 25 | 8 | odorant-binding protein | *Spodoptera frugiperda* | AAR28762.1 | 40.82 | 5e-25 | 3 |
| PflaOBP26 | 468 | 155 | Yes | 25 | 8 | odorant-binding protein 14 | *Streltzoviella insularis* | QLI62017.1 | 39.87 | 3e-31 | 3 |
| PflaOBP27 | 567 | 188 | Yes | 25 | 13 | odorant binding protein | *Glyphodes pyloalis* | QIJ45738.1 | 47.06 | 7e-45 | 3 |
| PflaOBP28 | 480 | 159 | Yes | 25 | 6 | odorant binding protein | *Eogystia hippophaecolus* | AOG12869.1 | 35.59 | 2e-16 | 3 |
| PflaOBP29 | 432 | 143 | Yes | 24 | 8 | odorant binding protein | *Eogystia hippophaecolus* | AOG12868.1 | 71.01 | 7e-61 | 3 |
| PflaOBP30 | 999 | 332 | Yes | 20 | 9 | odorant-binding protein 12 | *Streltzoviella insularis* | QLI62015.1 | 47.85 | 5e-79 | 3 |
| PflaOBP31 | 759 | 252 | Yes | 20 | 3 | odorant binding protein | *Eogystia hippophaecolus* | AOG12867.1 | 51.05 | 2e-89 | 3 |
| PflaOBP32 | 759 | 252 | Yes | 20 | 4 | odorant binding protein 9 | *Conopomorpha sinensis* | QGN03643.1 | 52.00 | 2e-94 | 3 |
| PflaOBP33 | 708 | 235 | Yes | 16 | 5 | odorant binding protein | *Glyphodes pyloalis* | QIJ45756.1 | 41.18 | 3e-45 | 3 |
| PflaOBP34 | 417 | 138 | Yes | No | 0 | odorant binding protein 19 | *Carposina sasakii* | AYD42194.1 | 58.82 | 2e-52 | 3 |
| PflaOBP35 | 336 | 111 | No | No | 6 | odorant binding protein 11 | *Grapholita molesta* | ALC79591.1 | 79.28 | 7e-60 | 3 |
| PflaOBP36 | 444 | 147 | Yes | No | 8 | odorant-binding protein 40 | *Lobesia botrana* | AXF48737.1 | 78.08 | 2e-81 | 3 |
| PflaOBP37 | 324 | 107 | No | No | 3 | general odorant binding protein | *Cinara cedri* | VVC30360.1 | 72.84 | 2e-25 | 3 |
| PflaOBP38 | 417 | 138 | Yes | 17 | 7 | general odorant-binding protein 72 | *Bicyclus anynana* | XP_023952583.1 | 77.04 | 2e-74 | 3 |
| PflaOBP39 | 555 | 184 | Yes | 22 | 9 | general odorant-binding protein 70 | *Papilio machaon* | XP_014371749.1 | 90.22 | 2e-121 | 3 |
| PflaOBP40 | 411 | 136 | Yes | 18 | 8 | general odorant-binding protein 69a | *Ostrinia furnacalis* | XP_028165883.1 | 69.85 | 3e-65 | 3 |
| PflaOBP41 | 597 | 198 | Yes | 24 | 9 | general odorant-binding protein 72 | *Papilio xuthus* | XP_013173037.1 | 64.46 | 1e-51 | 3 |
| PflaOBP42 | 552 | 183 | Yes | 25 | 6 | general odorant-binding protein 84a | *Galleria mellonella* | XP_026755199.1 | 49.64 | 5e-37 | 3 |
| PflaOBP43 | 348 | 115 | No | No | 6 | odorant binding protein 11 | *Grapholita molesta* | ALC79591.1 | 80.00 | 8e-63 | 3 |
| PflaGOBP1 | 504 | 167 | Yes | 23 | 7 | general odorant-binding protein 1 | *Galleria mellonella* | XP_026755845.1 | 73.05 | 3e-81 | 2 |
| PflaGOBP2 | 495 | 164 | Yes | 23 | 7 | general odorant-binding protein 2 | *Vanessa tameamea* | XP_026490959.1 | 77.44 | 1e-93 | 2 |
| PflaGOBP3 | 321 | 106 | No | 23 | 3 | general odorant-binding protein 1 | *Galleria mellonella* | XP_026755845.1 | 71.70 | 8e-42 | 3 |
| PflaGOBP5 | 453 | 150 | Yes | 23 | 6 | general odorant-binding protein 1 | *Streltzoviella insularis* | QLI62030.1 | 50.00 | 5e-34 | 3 |
| PflaGOBP6 | 348 | 115 | No | No | 6 | general odorant-binding protein 1 | *Aricia agestis* | XP_041984827.1 | 80.70 | 9e-65 | 3 |
| PflaGOBP7 | 369 | 122 | No | 23 | 3 | general odorant-binding protein 1 | *Galleria mellonella* | XP_026755845.1 | 70.75 | 1e-40 | 3 |
| PflaGOBP8 | 411 | 136 | Yes | No | 6 | general odorant binding protein 1 | *Cydia pomonella* | AFP66957.1 | 81.65 | 5e-60 | 3 |
| PflaGOBP10 | 321 | 106 | No | 23 | 3 | general odorant-binding protein 2 | *Galleria mellonella* | QEI46781.1 | 77.36 | 9e-54 | 3 |
| PflaGOBP11 | 321 | 106 | No | 23 | 3 | general odorant-binding protein 2 | *Vanessa tameamea* | XP_026490959.1 | 73.58 | 6e-54 | 3 |
| PflaGOBP12 | 762 | 253 | Yes | 23 | 10 | general odorant binding protein 1 | *Corcyra cephalonica* | UDM59724.1 | 74.03 | 4e-72 | 3 |
| PflaGOBP14 | 504 | 167 | Yes | 23 | 7 | general odorant-binding protein 1 | *Galleria mellonella* | XP_026755845.1 | 73.57 | 4e-64 | 3 |
| PflaGOBP15 | 339 | 112 | No | No | 6 | general odorant binding protein 2 | *Maruca vitrata* | AIN41151.1 | 82.14 | 2e-65 | 3 |
| PflaGOBP20 | 504 | 167 | Yes | 23 | 7 | general odorant-binding protein 1 | *Galleria mellonella* | XP_026755845.1 | 71.86 | 1e-86 | 3 |
| PflaPBP1 | 495 | 164 | Yes | 22 | 6 | pheromone-binding protein | *Dioryctria abietella* | AZK90260.1 | 76.76 | 2e-75 | 2 |
| PflaPBP2 | 492 | 163 | Yes | 21 | 6 | pheromone-binding protein 3 | *Grapholita molesta* | AHZ89399.1 | 60.87 | 4e-72 | 2 |

Note: Transcriptome source 2 or 3 means the antennal unigene transcriptome or the antennal full-length transcriptome.

**TABLE 4 |** Protein names and gene accession used in phylogenetic tree of OBPs

| **Name** | **ID** | **Name** | **ID** | **Name** | **ID** | **Name** | **ID** |
| --- | --- | --- | --- | --- | --- | --- | --- |
| BmorOBP1 | XP_037873010.1 | HarmPBP3 | AAO16091.1 | OfurGOBP1 | BAV56786.1 | SlituOBP27 | ALD65901 |
| BmorOBP2 | BAI22689.1 | GpylOBP1 | QIJ45745.1 | OfurGOBP2 | ABG66419.2 | SlituOBP29 | ALD65903.1 |
| BmorOBP3 | BAI22690.1 | GpylOBP2 | QIJ45744.1 | PxylOBP3 | ANC60174.1 | SlituOBP33 | ALD65907.1 |
| BmorOBP4 | XP_012547441.1 | GpylOBP3 | QIJ45757.1 | PxylOBP6 | ANG08530.1 | SlituGOBP1 | XP_022816701.1 |
| BmorOBP5 | BAI44700.1 | GpylOBP4 | QIJ45756.1 | PxylOBP9 | ANC60175.1 | SlituGOBP2 | XP_022817877.1 |
| BmorOBP6 | XP_012547442.1 | GpylOBP5 | QIJ45755.1 | PxylOBP12 | ANG08531.1 | SlituGOBP28a | XP_022826771.1 |
| BmorOBP7 | BAI44701.1 | GpylOBP6 | QIJ45752.1 | PxylOBP13 | ANG08532.1 | SlituGOBP69a | XP_022827633.1 |
| BmorOBP8 | BAH36759.1 | GpylOBP7 | QIJ45759.1 | PxylOBP18 | AMR99730.1 | SlituPBP1 | AIS72935.1 |
| BmorOBP9 | BAH36762.1 | GpylOBP8 | QIJ45758.1 | PxylOBP19 | ANC60176.1 | SlituPBP2 | AIS72933.1 |
| BmorOBP10 | BAH36763.1 | GpylOBP9 | QIJ45751.1 | PxylOBP23 | ANG08533.1 | SlituPBP3 | AIS72934.1 |
| BmorOBP11 | BAH79158.1 | GpylOBP10 | QIJ45749.1 | PxylOBP24 | AMR99731.1 | SlituPBP4 | AXO77502.1 |
| BmorOBP12 | BAH79159.1 | GpylOBP11 | QIJ45748.1 | PxylOBP28 | AMR99728.1 | SexiOBP1 | AGH70097.1 |
| BmorOBP13 | BAH36761.1 | GpylOBP12 | QIJ45747.1 | PxylOBP31 | ANG08529.1 | SexiOBP2 | AGH70098.1 |
| BmorGOBP1 | CAA64444.1 | GpylOBP13 | QIJ45743.1 | PxylOBP32 | ANG08535.1 | SexiOBP3 | AGH70099.1 |
| BmorGOBP2 | CAA64445.1 | GpylOBP14 | QIJ45742.1 | PxylOBP34 | ANG08534.1 | SexiOBP4 | AGH70100.1 |
| BmorPBP1 | AGR44745.1 | GpylOBP15 | QIJ45741.1 | PxylOBP35 | AMR99729.1 | SexiOBP5 | AGH70101.1 |
| BmorPBP3 | CAL47309.1 | GpylOBP16 | QIJ45740.1 | PxylOBP36 | AMR99732.1 | SexiOBP6 | AGH70102.1 |
| HarmOBP1 | AEB54580.1 | GpylOBP17 | QIJ45739.1 | PxylGOBP1 | ABY71034.1 | SexiOBP7 | AGH70103.1 |
| HarmOBP2 | AEB54586.1 | GpylOBP18 | QIJ45738.1 | PxylGOBP2 | ACE78188.1 | SexiOBP8 | AGH70104.1 |
| HarmOBP3 | AEB54582.1 | GpylOBP19 | QIJ45735.1 | PxylPBP1 | ACI28451.1 | SexiOBP9 | AGH70105.1 |
| HarmOBP4 | AEB54584.1 | GpylGOBP1 | QIJ45730.1 | PxylPBP3 | XP_011550154.2 | SexiOBP10 | AGH70106.1 |
| HarmOBP5 | AEB54581.1 | GpylGOBP2 | QIJ45727.1 | PxylPBP2 | AGH13203.1 | SexiOBP11 | AGH70107.1 |
| HarmOBP6 | AEB54587.1 | GpylGOBP3 | QIJ45728.1 | SlituOBP1 | AKI87962.1 | SexiOBP17 | AKT26495.1 |
| HarmOBP7 | AEB54591.1 | GpylPBP1 | QIJ45734.1 | SlituOBP4 | AKI87965.1 | SexiOBP18 | AKT26496.1 |
| HarmOBP8 | AEB54589.1 | GpylPBP2 | QIJ45732.1 | SlituOBP5 | AKI87966.1 | SexiOBP19 | AKT26497.1 |
| HarmOBP9 | AEB54592.1 | GpylPBP3 | QIJ45731.1 | SlituOBP6 | AKI87967.1 | SexiOBP20 | AKT26498.1 |
| HarmOBP13 | AEB54588.1 | OfurOBP1 | BAV56788.1 | SlituOBP7 | AKI87968.1 | SexiOBP22 | AKT26499.1 |
| HarmOBP15 | AEB54590.1 | OfurOBP4 | BAV56791.1 | SlituOBP8 | AKI87969.1 | SexiOBP23 | AKT26500.1 |
| HarmOBP16 | AFI57165.1 | OfurOBP5 | BAV56792.1 | SlituOBP9 | ALD65883.1 | SexiOBP24 | AKT26501.1 |
| HarmOBP17 | AFI57166.1 | OfurOBP6 | BAV56793.1 | SlituOBP10 | ALD65884.1 | SexiOBP25 | AKT26502.1 |
| HarmOBP18 | AFI57167.1 | OfurOBP7 | BAV56794.1 | SlituOBP11 | ALD65885.1 | SexiOBP26 | AKT26503.1 |
| HarmOBP19 | AFM93773.1 | OfurOBP8 | BAV56795.1 | SlituOBP12 | ALD65886.1 | SexiOBP27 | ADY17882.1 |
| HarmOBP31 | ASA40067.1 | OfurOBP9 | BAV56796.1 | SlituOBP13 | ALD65887.1 | SexiOBP28 | ADY17883.1 |
| HarmOBP35 | ASA40068.1 | OfurOBP10 | BAV56797.1 | SlituOBP15 | ALD65889.1 | SexiOBP29 | ADY17884.1 |
| HarmOBP36 | ASA40069.1 | OfurOBP11 | BAV56798.1 | SlituOBP16 | ALD65890.1 | SexiOBP30 | ADY17885.1 |
| HarmGOBP1 | XP_021192665.1 | OfurOBP12 | BAV56799.1 | SlituOBP17 | ALD65891.1 | SexiOBP31 | ADY17886.1 |
| HarmGOBP2 | XP_021192653.1 | OfurOBP13 | BAV56800.1 | SlituOBP18 | ALD65892.1 | SexiGOBP1 | ACY78412.1 |
| HarmGOBP56a | XP_021196568.1 | OfurOBP14 | BAV56801.1 | SlituOBP19 | ALD65893.1 | SexiGOBP2 | CAC12832.1 |
| HarmGOBP69a | XP_021194654.1 | OfurOBP15 | BAV56802.1 | SlituOBP20 | ALD65894.1 | SexiPBP1 | AAS46620.1 |
| HarmPBP1 | AEB54585.1 | OfurOBP16 | BAV56803.1 | SlituOBP21 | ALD65895.1 | SexiPBP2 | AAU95537.1 |
| HarmPBP2 | AEB54583.1 | OfurOBP17 | BAV56804 | SlituOBP25 | ALD65899.1 | SexiPBP3 | ACY78413.1 |

**TABLE 5 |** Blastx matches for genes encoding candidate CSPs in *P. flammans*

| Gene  Name | Gene  length  (bp) | ORF  (aa) | Complete  ORF | Signal  Peptide  (aa) | Best blastx match | | | | | Transcriptome  Source  (No.) |
| --- | --- | --- | --- | --- | --- | --- | --- | --- | --- | --- |
|  |  |  |  |  | Gene description | species | Accession  number | Identity  % | E-value |  |
| PflaCSP1 | 546 | 181 | Yes | 19 | chemosensory protein 9 | *Bombyx mori* | NP_001037069.1 | 69.18 | 1e-70 | 3 |
| PflaCSP2 | 378 | 125 | Yes | 18 | chemosensory protein 18 | *Lobesia botrana* | AXF48714.1 | 60.00 | 3e-50 | 3 |
| PflaCSP3 | 402 | 133 | Yes | 19 | chemosensory protein 17 | *Ectropis obliqua* | ALS03842.1 | 70.87 | 1e-48 | 3 |
| PflaCSP4 | 390 | 129 | Yes | 18 | chemosensory protein 6 | *Agrotis ipsilon* | AGR39576.1 | 68.25 | 5e-60 | 3 |
| PflaCSP5 | 369 | 122 | Yes | 18 | chemosensory protein 7 | *Conogethes pinicolalis* | QFR36134.1 | 70.87 | 2e-48 | 3 |
| PflaCSP6 | 378 | 125 | Yes | 18 | chemosensory protein 18 | *Lobesia botrana* | AXF48714.1 | 60.80 | 4e-51 | 3 |
| PflaCSP7 | 369 | 122 | Yes | 18 | chemosensory protein 7 | *Conogethes pinicolalis* | QFR36134.1 | 70.25 | 2e-55 | 3 |
| PflaCSP8 | 369 | 122 | Yes | 18 | chemosensory protein 3 | *Cnaphalocrocis medinalis* | AGI37365.1 | 70.87 | 8e-49 | 3 |
| PflaCSP9 | 360 | 119 | Yes | 15 | chemosensory protein 8.1 | *Lobesia botrana* | AXF48705.1 | 57.26 | 2e-43 | 3 |
| PflaCSP10 | 312 | 103 | Yes | 18 | chemosensory protein 6 | *Carposina sasakii* | AYD42210.1 | 36.00 | 1e-14 | 3 |
| PflaCSP11 | 359 | 118 | No | 18 | chemosensory protein 7 | *Grapholita molesta* | QEV81552.1 | 60.20 | 1e-36 | 2 |
| PflaCSP12 | 369 | 122 | Yes | 16 | chemosensory protein | *Dendrolimus kikuchii* | AII01037.1 | 39.34 | 6e-20 | 2 |
| PflaCSP13 | 411 | 136 | Yes | 19 | chemosensory protein | *Sesamia inferens* | AGY49270.1 | 70.91 | 5e-51 | 2 |
| PflaCSP14 | 360 | 119 | Yes | 16 | Chemosensory protein 18 | *Dendrolimus punctatus* | ARO70322.1 | 62.18 | 1e-45 | 2 |
| PflaCSP15 | 369 | 122 | Yes | 18 | chemosensory protein 3 | *Cnaphalocrocis medinalis* | AGI37365.1 | 70.87 | 2e-48 | 2 |
| PflaCSP16 | 312 | 103 | Yes | 18 | chemosensory protein 1 | *Cnaphalocrocis medinalis* | AGI37361.1 | 38.38 | 7e-14 | 2 |
| PflaCSP17 | 333 | 110 | Yes | 17 | chemosensory protein | *Helicoverpa armigera* | AIW65101.1 | 81.98 | 5e-56 | 2 |
| PflaCSP18 | 366 | 121 | Yes | 19 | chemosensory protein 13 | *Cydia pomonella* | ATD12156.1 | 51.46 | 1e-31 | 2 |
| PflaCSP19 | 369 | 122 | Yes | 17 | chemosensory protein 7 | *Bombyx mori* | NP_001037068.1 | 100.00 | 6e-83 | 2 |

**TABLE 6 |** Protein names and gene accession used in phylogenetic tree of CSPs

| **Name** | **ID** | **Name** | **ID** | **Name** | **ID** | **Name** | **ID** |
| --- | --- | --- | --- | --- | --- | --- | --- |
| AdisCSP1 | AND82443.1 | EoblCSP4 | ALS03829.1 | GmelCSP4 | QEI46802.1 | OfurCSP15 | BAV56819.1 |
| AdisCSP2 | AND82444.1 | EoblCSP5 | ALS03830.1 | GmelCSP5 | QEI46803.1 | OfurCSP16 | BAV56820.1 |
| AdisCSP3 | AND82445.1 | EoblCSP6 | ALS03831.1 | GmelCSP6 | QEI46804.1 | OfurCSP17 | BAV56821.1 |
| AdisCSP4 | AND82446.1 | EoblCSP7 | ALS03832.1 | GmelCSP7 | QEI46805.1 | OfurCSP18 | BAV56822.1 |
| AdisCSP5 | AND82447.1 | EoblCSP8 | ALS03833.1 | GmelCSP8 | QEI46806.1 | OfurCSP19 | BAV56823.1 |
| AdisCSP6 | AND82448.1 | EoblCSP9 | ALS03834.1 | GmelCSP9 | QEI46807.1 | SlitCSP1 | ALJ30212.1 |
| AdisCSP7 | AND82449.1 | EoblCSP10 | ALS03835.1 | GmelCSP10 | QEI46808.1 | SlitCSP2 | ALJ30213.1 |
| AdisCSP8 | AND82450.1 | EoblCSP11 | ALS03836.1 | GmelCSP11 | QEI46809.1 | SlitCSP3 | ALJ30214.1 |
| AdisCSP9 | AND82451.1 | EoblCSP12 | ALS03837.1 | GmelCSP12 | QEI46810.1 | SlitCSP4 | ALJ30215.1 |
| AdisCSP10 | AND82452.1 | EoblCSP13 | ALS03838.1 | GmelCSP13 | QEI46811.1 | SlitCSP5 | ALJ30216.1 |
| BmorCSP1 | XP_012549255.1 | EoblCSP14 | ALS03839.1 | GmelCSP14 | QEI46812.1 | SlitCSP6 | ALJ30217.1 |
| BmorCSP2 | AAM34275.1 | EoblCSP15 | ALS03840.1 | GmelCSP15 | QEI46813.1 | SlitCSP7 | ALJ30218.1 |
| BmorCSP3 | NP_001037063.1 | EoblCSP16 | ALS03841.1 | GmelCSP16 | QEI46814.1 | SlitCSP8 | ALJ30219.1 |
| BmorCSP4 | NP_001037052.1 | EoblCSP17 | ALS03842.1 | GmelCSP17 | QEI46815.1 | SlitCSP9 | ALJ30220.1 |
| BmorCSP5 | NP_001037062.1 | EoblCSP18 | ALS03843.1 | GmelCSP18 | QEI46816.1 | SlitCSP10 | ALJ30221.1 |
| BmorCSP6 | XP_037873394.1 | EoblCSP19 | ALS03844.1 | MsepCSP1 | AWT22249.1 | SlitCSP12 | ALJ30223.1 |
| BmorCSP7 | NP_001037068.1 | EoblCSP20 | ALS03845.1 | MsepCSP2 | JAV45879.1 | SlitCSP13 | ALJ30224.1 |
| BmorCSP8 | NP_001037067.1 | EoblCSP21 | ALS03846.1 | MsepCSP3 | JAV45878.1 | SexiCSP1 | AKF42444.1 |
| BmorCSP9 | NP_001037069.1 | HassCSP18 | AGH20056.1 | MsepCSP4 | JAV45877.1 | SexiCSP2 | AKF42443.1 |
| BmorCSP10 | NP_001037064.1 | HassCSP20 | ASA40081.1 | MsepCSP5 | JAV45876.1 | SexiCSP3 | AKF42442.1 |
| BmorCSP11 | NP_001091779.1 | HassCSP21 | ASA40082.1 | MsepCSP6 | JAV45875.1 | SexiCSP4 | AKT26481.1 |
| BmorCSP12 | XP_021206019.2 | HassCSP22 | ASA40083.1 | MsepCSP7 | JAV45874.1 | SexiCSP5 | AKT26482.1 |
| BmorCSP13 | NP_001037180.1 | HassCSP23 | ASA40084.1 | MsepCSP8 | JAV45873.1 | SexiCSP6 | AKT26483.1 |
| BmorCSP14 | NP_001037192.1 | HassCSP24 | ASA40085.1 | MsepCSP9 | JAV45872.1 | SexiCSP7 | AKT26484.1 |
| BmorCSP15 | NP_001091781.1 | HassCSP25 | ASA40086.1 | MsepCSP10 | JAV45871.1 | SexiCSP8 | AKT26485.1 |
| BmorCSP16 | NP_001091782.1 | HarmCSP8 | AFR92092.1 | MsepCSP11 | JAV45870.1 | SexiCSP10 | AKT26486.1 |
| CmedCSP1 | AGI37361.1 | HarmCSP9 | AFR92093.1 | MsepCSP12 | JAV45869.1 | SexiCSP11 | AKT26487.1 |
| CmedCSP2 | AGI37363.1 | HarmCSP10 | AFR92094.1 | MsepCSP13 | JAV45868.1 | SexiCSP12 | AKT26488.1 |
| CmedCSP3 | AGI37365.1 | HarmCSP11 | AFR92095.1 | MsepCSP14 | JAV45867.1 | SexiCSP13 | AKT26489.1 |
| CmedCSP23 | ALT31605.1 | HarmCSP12 | AFR92096.1 | MsepCSP15 | JAV45866.1 | SexiCSP14 | AKT26490.1 |
| CmedCSP24 | ALT31606.1 | HarmCSP13 | AFR92097.1 | MsepCSP16 | JAV45865.1 | SexiCSP16 | AKT26491.1 |
| CmedCSP25 | ALT31607.1 | HarmCSP14 | AFR92098.1 | OfurCSP1 | BAV56805.1 | SexiCSP18 | AKT26492.1 |
| CmedCSP26 | ALT31608.1 | HarmCSP15 | AGH20053.1 | OfurCSP2 | BAV56806.1 | SexiCSP19 | AKT26493.1 |
| CmedCSP27 | ALT31609.1 | HarmCSP16 | AGH20054.1 | OfurCSP3 | BAV56807.1 | SexiCSP20 | AKT26494.1 |
| CmedCSP28 | ALT31610.1 | HarmCSP17 | AGH20055.1 | OfurCSP4 | BAV56808.1 | SinsCSP1 | QLI62032.1 |
| CmedCSP29 | ALT31611.1 | HarmCSP20 | ASA40076.1 | OfurCSP5 | BAV56809.1 | SinsCSP2 | QLI62033.1 |
| CmedCSP30 | ALT31612.1 | HarmCSP21 | ASA40077.1 | OfurCSP6 | BAV56810.1 | SinsCSP3 | QLI62034.1 |
| CmedCSP31 | ALT31613.1 | HarmCSP22 | ASA40078.1 | OfurCSP7 | BAV56811.1 | SinsCSP4 | QLI62035.1 |
| CmedCSP32 | ALT31614.1 | HarmCSP23 | ASA40079.1 | OfurCSP8 | BAV56812.1 | SinsCSP5 | QLI62036.1 |
| CmedCSP33 | ALT31615.1 | HarmCSP24 | ASA40080.1 | OfurCSP9 | BAV56813.1 | SinsCSP6 | QLI62037.1 |
| CmedCSP34 | ALT31616.1 | HarmCSP25 | ASA40087.1 | OfurCSP10 | BAV56814.1 | SinsCSP7 | QLI62038.1 |
| CmedCSP35 | ALT31617.1 | HarmCSP26 | ASA40088.1 | OfurCSP11 | BAV56815.1 | SinsCSP8 | QLI62039.1 |
| CmedCSP36 | APY22695.1 | GmelCSP1 | QEI46799.1 | OfurCSP12 | BAV56816.1 | SinsCSP9 | QLI62040.1 |
| EoblCSP1 | ALS03827.1 | GmelCSP2 | QEI46800.1 | OfurCSP13 | BAV56817.1 | SinsCSP10 | QLI62041.1 |
| EoblCSP3 | ALS03828.1 | GmelCSP3 | QEI46801.1 | OfurCSP14 | BAV56818.1 | SinsCSP11 | QLI62042.1 |

**TABLE 7 |** Blastx matches for genes encoding candidate ORs in *P. flammans*

| Gene Name | Gene  Length  (bp) | ORF (aa) | Complete  ORF | TMD  (No.) | Best blastx match | | | | | Transcriptome  source  (No.) |
| --- | --- | --- | --- | --- | --- | --- | --- | --- | --- | --- |
|  |  |  |  |  | Gene description | species | Accession number | Identity  % | E-value |  |
| PflaOrco | 1425 | 474 | Yes | 7 | odorant receptor | *Ostrinia furnacalis* | AGG91643.1 | 90.51 | 0 | 2 |
| PflaOR3 | 816 | 271 | No | 5 | odorant receptor | *Histia rhodope* | QMS80346.1 | 76.01 | 1e-147 | 2 |
| PflaOR4 | 1290 | 429 | Yes | 7 | odorant receptor | *Eogystia hippophaecolus* | AOG12933.1 | 55.04 | 3e-150 | 2 |
| PflaOR5 | 1176 | 391 | Yes | 6 | olfactory receptor 11 | *Ctenopseustis herana* | AIT69876.1 | 47.98 | 8e-113 | 2 |
| PflaOR6 | 1179 | 392 | Yes | 7 | odorant receptor 94a | *Galleria mellonella* | XP_026759878.1 | 32.82 | 8e-75 | 2 |
| PflaOR7 | 1227 | 408 | Yes | 7 | odorant receptor 1 | *Helicoverpa armigera* | XP_021196261.1 | 54.36 | 1e-155 | 2 |
| PflaOR8 | 1092 | 363 | Yes | 6 | odorant receptor 50 | *Streltzoviella insularis* | QLI62093.1 | 60.00 | 7e-155 | 2 |
| PflaOR9 | 744 | 247 | No | 5 | odorant receptor | *Histia rhodope* | QMS80321.1 | 69.64 | 2e-126 | 2 |
| PflaOR10 | 1218 | 405 | Yes | 7 | odorant receptor 19 | *Achelura yunnanensis* | QZH55115.1 | 63.95 | 1e-178 | 2 |
| PflaOR11 | 1179 | 392 | Yes | 7 | odorant receptor | *Eogystia hippophaecolus* | AOG12927.1 | 66.16 | 2e-173 | 2 |
| PflaOR12 | 1170 | 389 | Yes | 7 | odorant receptor 43a | *Pararge aegeria* | XP_039758114.1 | 59.13 | 1e-165 | 2 |
| PflaOR13 | 1188 | 395 | Yes | 7 | odorant receptor 59a | *Manduca sexta* | XP_037298859.1 | 51.65 | 2e-112 | 2 |
| PflaOR14 | 1059 | 352 | Yes | 6 | odorant receptor | *Histia rhodope* | QMS80326.1 | 40.00 | 2e-68 | 2 |
| PflaOR15 | 1248 | 415 | Yes | 7 | odorant receptor 67a | *Plutella xylostella* | XP_037961479.1 | 59.28 | 2e-180 | 2 |
| PflaOR16 | 1173 | 391 | Yes | 7 | odorant receptor | *Semiothisa cinerearia* | QRF70985.1 | 64.27 | 0 | 2 |
| PflaOR17 | 1065 | 353 | Yes | 7 | odorant receptor 26 | *Cydia fagiglandana* | AST36313.1 | 31.28 | 2e-55 | 2 |
| PflaOR18 | 1365 | 436 | Yes | 6 | olfactory receptor 27 | *Mythimna separata* | QNS36223.1 | 44.39 | 1e-98 | 2 |
| PflaOR19 | 1224 | 407 | Yes | 6 | odorant receptor 85c | *Vanessa tameamea* | XP_026493935.1 | 37.25 | 6e-80 | 2 |
| PflaOR20 | 1362 | 454 | Yes | 6 | olfactory receptor 71 | *Ctenopseustis herana* | AIT69911.1 | 62.16 | 0.0 | 2 |
| PflaOR21 | 1176 | 391 | Yes | 7 | odorant receptor | *Helicoverpa armigera* | QPX50365.1 | 36.90 | 4e-69 | 2 |
| PflaOR23 | 1173 | 390 | Yes | 7 | olfactory receptor 56 | *Bombyx mori* | NP_001166617.1 | 53.67 | 1e-151 | 2 |
| PflaOR24 | 1119 | 372 | Yes | 6 | odorant receptor 85c | *Vanessa tameamea* | XP_026493935.1 | 30.65 | 3e-45 | 2 |
| PflaOR25 | 1224 | 407 | Yes | 7 | odorant receptor 47a | *Bombyx mandarina* | XP_028030655.1 | 65.04 | 0 | 2 |
| PflaOR26 | 1281 | 427 | Yes | 3 | odorant receptor 55 | *Streltzoviella insularis* | QLI62098.1 | 47.54 | 2e-72 | 2 |
| PflaOR27 | 720 | 239 | No | 4 | olfactory receptor 1 | *Antheraea pernyi* | CBH19583.1 | 53.66 | 4e-63 | 2 |
| PflaOR28 | 1272 | 423 | Yes | 6 | olfactory receptor 74 | *Mythimna separata* | QNS36250.1 | 44.92 | 2e-132 | 2 |
| PflaOR29 | 1230 | 410 | Yes | 7 | odorant receptor | *Eogystia hippophaecolus* | AOG12917.1 | 41.63 | 1e-109 | 2 |
| PflaOR30 | 1200 | 399 | Yes | 6 | odorant receptors 66 | *Lobesia botrana* | AXF48805.1 | 42.52 | 2e-106 | 2 |
| PflaOR33 | 1173 | 390 | Yes | 6 | odorant receptor | *Dendrolimus houi* | AII01058.1 | 52.12 | 1e-132 | 2 |
| PflaOR34 | 1194 | 397 | Yes | 7 | olfactory receptor 50 | *Carposina sasakii* | AYD42268.1 | 52.26 | 5e-144 | 2 |
| PflaOR35 | 1182 | 393 | Yes | 7 | odorant receptor | *Eogystia hippophaecolus* | AOG12934.1 | 41.52 | 1e-104 | 2 |
| PflaOR36 | 1218 | 405 | Yes | 0 | odorant receptor, partial | *Eogystia hippophaecolus* | AOG12922.1 | 56.57 | 2e-55 | 2 |
| PflaOR37 | 1440 | 479 | Yes | 8 | odorant receptor 15 | *Streltzoviella insularis* | QLI62058.1 | 38.73 | 4e-86 | 2 |
| PflaOR38 | 1038 | 345 | Yes | 5 | Odorant Receptor 13 | *Dendrolimus punctatus* | ARO70225.1 | 55.03 | 2e-117 | 2 |
| PflaOR40 | 834 | 277 | No | 4 | odorant receptor | *Helicoverpa armigera* | AIG51894.1 | 32.97 | 1e-47 | 2 |
| PflaOR41 | 1134 | 377 | Yes | 7 | odorant receptor | *Dendrolimus kikuchii* | AII01092.1 | 44.68 | 7e-108 | 2 |
| PflaOR42 | 1185 | 394 | Yes | 7 | odorant receptor | *Eogystia hippophaecolus* | AOG12934.1 | 38.89 | 3e-89 | 2 |
| PflaOR43 | 1215 | 404 | Yes | 6 | odorant receptor 18 | *Achelura yunnanensis* | QZH55114.1 | 44.91 | 4e-118 | 2 |
| PflaOR44 | 1212 | 404 | Yes | 7 | odorant receptor | *Eogystia hippophaecolus* | AOG12954.1 | 51.96 | 1e-143 | 2 |
| PflaOR45 | 1239 | 412 | Yes | 6 | odorant receptor 34 | *Plutella xylostella* | QZA75630.1 | 39.20 | 2e-97 | 2 |
| PflaOR46 | 1245 | 414 | Yes | 6 | odorant receptor 34 | *Plutella xylostella* | QZA75630.1 | 37.56 | 6e-89 | 2 |
| PflaOR47 | 1182 | 393 | Yes | 7 | odorant receptor 22 | *Streltzoviella insularis* | QLI62065.1 | 56.99 | 2e-167 | 2 |
| PflaOR48 | 795 | 264 | No | 4 | olfactory receptor 7 | *Helicoverpa armigera* | AGK90001.1 | 32.52 | 3e-12 | 2 |
| PflaOR49 | 1236 | 412 | Yes | 7 | odorant receptor 5 | *Ostrinia nubilalis* | ADB89182.1 | 35.06 | 3e-84 | 2 |
| PflaOR50 | 1191 | 397 | Yes | 7 | olfactory receptor 4 | *olfactory receptor 4* | ACF32962.1 | 73.68 | 0 | 2 |
| PflaOR51 | 1236 | 412 | Yes | 7 | odorant receptor 3 | *Cydia pomonella* | AFC91713.2 | 36.94 | 1e-85 | 2 |
| PflaOR52 | 1221 | 406 | Yes | 6 | olfactory receptor | *Glyphodes pyloalis* | QIJ45817.1 | 40.52 | 3e-85 | 2 |
| PflaOR53 | 687 | 228 | No | 4 | olfactory receptor 20 | *Heortia vitessoides* | AZB49434.1 | 76.83 | 7e-84 | 2 |
| PflaOR54 | 1218 | 405 | NO | 7 | olfactory receptor 4 | *Helicoverpa armigera* | ACF32962.1 | 32.85 | 2e-45 | 2 |
| PflaOR55 | 1209 | 402 | Yes | 7 | odorant receptor 4 | *Papilio polytes* | XP_013144580.1 | 69.15 | 0 | 2 |
| PflaOR56 | 1227 | 408 | Yes | 7 | odorant receptor | *Eogystia hippophaecolus* | AOG12913.1 | 36.23 | 9e-74 | 2 |
| PflaOR57 | 1221 | 406 | Yes | 6 | odorant receptor 21 | *Achelura yunnanensis* | QZH55117.1 | 47.52 | 1e-135 | 2 |
| PflaOR58 | 1191 | 396 | Yes | 7 | odorant receptor | *Eogystia hippophaecolus* | AOG12915.1 | 61.11 | 4e-161 | 2 |
| PflaOR59 | 1101 | 366 | Yes | 5 | odorant receptor 85e | *Helicoverpa armigera* | XP_021183770.1 | 65.13 | 3e-152 | 3 |
| PflaOR60 | 1215 | 404 | Yes | 7 | odorant receptor 10 | *Streltzoviella insularis* | QLI62053.1 | 51.19 | 3e-146 | 3 |
| PflaOR61 | 1224 | 407 | Yes | 7 | odorant receptor 3 | *Cydia pomonella* | AFC91713.2 | 36.77 | 1e-74 | 3 |
| PflaOR62 | 1266 | 421 | Yes | 7 | odorant receptor | *Eogystia hippophaecolus* | AOG12954.1 | 51.20 | 5e-145 | 3 |
| PflaOR63 | 1239 | 412 | Yes | 7 | odorant receptor 5 | *Ostrinia nubilalis* | ADB89182.1 | 35.29 | 9e-73 | 3 |
| PflaOR64 | 762 | 244 | NO | 3 | odorant receptor | *Dendrolimus kikuchii* | AII01102.1 | 60.82 | 2e-100 | 3 |

**TABLE 8 |** Protein names and gene accession used in phylogenetic tree of ORs

| **Name** | **ID** | **Name** | **ID** | **Name** | **ID** | **Name** | **ID** |
| --- | --- | --- | --- | --- | --- | --- | --- |
| CpomOR1 | AFC91714.1 | DpunOR21 | ARO70233.1 | EposOR6 | JAI18060.1 | EsemOR18 | (Yuvaraj et al., 2017) |
| CpomOR2a | AFC91715.2 | DpunOR22 | ARO70234.1 | EposOR7 | JAI18059.1 | EsemOR19 | (Yuvaraj et al., 2017) |
| CpomOR2b | JAP38462.1 | DpunOR23 | ARO70235.1 | EposOR8 | (Corcoran et al., 2015) | EsemOR20 | (Yuvaraj et al., 2017) |
| CpomOR2c | JAP38461.1 | DpunOR24 | ARO70236.1 | EposOR9 | (Corcoran et al., 2015) | EsemOR21 | (Yuvaraj et al., 2017) |
| CpomOR3 | AFC91713.2 | DpunOR25 | ARO70237.1 | EposOR10 | JAI18058.1 | EsemOR22 | (Yuvaraj et al., 2017) |
| CpomOR4 | AFC91716.2 | DpunOR26 | ARO70238.1 | EposOR12 | JAI18057.1 | EsemOR23 | (Yuvaraj et al., 2017) |
| CpomOR5 | JAP38459.1 | DpunOR27 | ARO70239.1 | EposOR13 | (Corcoran et al., 2015) | EsemOR24 | (Yuvaraj et al., 2017) |
| CpomOR6a | AFC91711.2 | DpunOR29 | ARO70241.1 | EposOR14 | JAI18056.1 | EsemOR25 | (Yuvaraj et al., 2017) |
| CpomOR6b | JAP38458.1 | DpunOR30 | ARO70245.1 | EposOR15 | JAI18055.1 | EsemOR26 | (Yuvaraj et al., 2017) |
| CpomOR7 | JAP38457.1 | DpunOR31 | ARO70243.1 | EposOR16 | (Corcoran et al., 2015) | EsemOR27 | (Yuvaraj et al., 2017) |
| CpomOR8 | JAP38456.1 | DpunOR32 | ARO70244.1 | EposOR18 | JAI18053.1 | EsemOR28 | (Yuvaraj et al., 2017) |
| CpomOR9 | JAP38455.1 | DpunOR34 | ARO70246.1 | EposOR19 | JAI18052.1 | EsemOR29 | (Yuvaraj et al., 2017) |
| CpomOR10 | AFC91736.1 | DpunOR39 | ARO70514.1 | EposOR20 | (Corcoran et al., 2015) | EsemOR30 | (Yuvaraj et al., 2017) |
| CpomOR11 | AFC91720.2 | DpunOR40 | ARO70252.1 | EposOR21 | JAI18051.1 | EsemOR31 | (Yuvaraj et al., 2017) |
| CpomOR12 | JAP38454.1 | DpunOR41 | ARO70253.1 | EposOR22 | JAI18050.1 | EsemOR32 | (Yuvaraj et al., 2017) |
| CpomOR13 | JAP38453.1 | DpunOR44 | ARO70256.1 | EposOR23 | (Corcoran et al., 2015) | EsemOR35 | (Yuvaraj et al., 2017) |
| CpomOR14 | AFC91722.1 | DpunOR45 | ARO70516.1 | EposOR24 | JAI18049.1 | EsemORCO | ATV96621.1 |
| CpomOR15 | AFC91728.1 | DpunOR46 | ARO70517.1 | EposOR25 | JAI18048.1 | SlitOR1 | (de Fouchier et al., 2017) |
| CpomOR16 | JAP38452.1 | DpunOR47 | ARO70518.1 | EposOR26 | JAI18047.1 | SlitOR3 | (de Fouchier et al., 2017) |
| CpomOR18 | AFC91719.1 | DpunOR49 | ARO70261.1 | EposOR27 | JAI18046.1 | SlitOR4 | (de Fouchier et al., 2017) |
| CpomOR19 | AFC91727.1 | DpunOR50 | ARO70262.1 | EposOR28 | JAI18045.1 | SlitOR5 | (de Fouchier et al., 2017) |
| CpomOR20 | AFC91726.1 | DpunOR51 | ARO70263.1 | EposOR29 | JAI18044.1 | SlitOR6 | (de Fouchier et al., 2017) |
| CpomOR21 | JAP38451.1 | DpunOR54 | ARO70520.1 | EposOR30 | JAI18043.1 | SlitOR7 | (de Fouchier et al., 2017) |
| CpomOR22 | AFC91723.2 | DpunOR58 | ARO70521.1 | EposOR31 | JAI18042.1 | SlitOR8 | (de Fouchier et al., 2017) |
| CpomOR25 | AFC91729.1 | DpunOR59 | ARO70522.1 | EposOR32 | JAI18041.1 | SlitOR9 | (de Fouchier et al., 2017) |
| CpomOR26 | JAP38450.1 | DpunOR61 | ARO70523.1 | EposOR33 | JAI18040.1 | SlitOR10 | (de Fouchier et al., 2017) |
| CpomOR27 | AFC91735.2 | DpunOR62 | ARO70524.1 | EposOR34 | JAI18039.1 | SlitOR11 | (de Fouchier et al., 2017) |
| CpomOR28 | AFC91734.2 | DpunOR65 | ARO70527.1 | EposOR35 | JAI18038.1 | SlitOR12 | (de Fouchier et al., 2017) |
| CpomOR29 | JAP38449.1 | DpunOR66 | ARO70528.1 | EposOR36 | JAI18037.1 | SlitOR13 | (de Fouchier et al., 2017) |
| CpomOR30 | AFC91738.2 | DpunOR67 | ARO70529.1 | EposOR37 | JAI18036.1 | SlitOR14 | (de Fouchier et al., 2017) |
| CpomOR31 | JAP38448.1 | DpunOR68 | ARO70530.1 | EposOR38 | JAI18035.1 | SlitOR15 | (de Fouchier et al., 2017) |
| CpomOR32 | JAP38447.1 | DpunOrco | ARO70214.1 | EposOR39 | JAI18034.1 | SlitOR16 | (de Fouchier et al., 2017) |
| CpomOR35 | AFC91743.2 | EgriOR2 | (Li et al., 2017) | EposOR40 | JAI18033.1 | SlitOR17 | (de Fouchier et al., 2017) |
| CpomOR37 | AFC91744.1 | EgriOR3 | (Li et al., 2017) | EposOR41 | JAI18032.1 | SlitOR18 | (de Fouchier et al., 2017) |
| CpomOR38 | JAP38446.1 | EgriOR4 | (Li et al., 2017) | EposOR42 | JAI18031.1 | SlitOR19 | (de Fouchier et al., 2017) |
| CpomOR39 | AFC91746.1 | EgriOR5 | (Li et al., 2017) | EposOR43 | JAI18030.1 | SlitOR20 | (de Fouchier et al., 2017) |
| CpomOR40 | AFC91741.2 | EgriOR6 | (Li et al., 2017) | EposOR44 | JAI18029.1 | SlitOR21 | (de Fouchier et al., 2017) |
| CpomOR41 | JAP38445.1 | EgriOR7 | (Li et al., 2017) | EposOR45 | JAI18028.1 | SlitOR22 | (de Fouchier et al., 2017) |
| CpomOR42 | AFC91750.2 | EgriOR8 | (Li et al., 2017) | EposOR46 | JAI18028.1 | SlitOR23 | (de Fouchier et al., 2017) |
| CpomOR44 | JAP38444.1 | EgriOR9 | (Li et al., 2017) | EposOR47 | JAI18026.1 | SlitOR24 | (de Fouchier et al., 2017) |
| CpomOR46 | AFC91724.1 | EgriOR10 | (Li et al., 2017) | EposOR48 | JAI18025.1 | SlitOR25 | (de Fouchier et al., 2017) |
| CpomOR47 | JAP38443.1 | EgriOR11 | (Li et al., 2017) | EposOR49 | JAI18024.1 | SlitOR26 | (de Fouchier et al., 2017) |
| CpomOR49 | AFC91746.1 | EgriOR12 | (Li et al., 2017) | EposOR50 | (Corcoran et al., 2015) | SlitOR27 | (de Fouchier et al., 2017) |
| CpomOR53 | AFC91718.2 | EgriOR13 | (Li et al., 2017) | EposOR51 | JAI18023.1 | SlitOR28 | (de Fouchier et al., 2017) |
| CpomOR54 | AFC91717.2 | EgriOR14 | (Li et al., 2017) | EposOR52 | JAI18022.1 | SlitOR29 | (de Fouchier et al., 2017) |
| CpomOR56 | AFC91745.2 | EgriOR15 | (Li et al., 2017) | EposOR53 | JAI18021.1 | SlitOR30 | (de Fouchier et al., 2017) |
| CpomOR57 | AFC91739.1 | EgriOR16 | (Li et al., 2017) | EposOR54 | JAI18020.1 | SlitOR31 | (de Fouchier et al., 2017) |
| CpomOR58 | AFC91742.1 | EgriOR17 | (Li et al., 2017) | EposOR55 | JAI18019.1 | SlitOR32 | (de Fouchier et al., 2017) |
| CpomOR59 | AFC91721.1 | EgriOR18 | (Li et al., 2017) | EposOR56 | (Corcoran et al., 2015) | SlitOR33 | (de Fouchier et al., 2017) |
| CpomOR60 | AFC91742.1 | EgriOR19 | (Li et al., 2017) | EposOR57 | JAI18018.1 | SlitOR34 | (de Fouchier et al., 2017) |
| CpomOR61 | AFC91725.2 | EgriOR20 | (Li et al., 2017) | EposOR58 | JAI18017.1 | SlitOR35 | (de Fouchier et al., 2017) |
| CpomOR62 | JAP38440.1 | EgriOR21 | (Li et al., 2017) | EposOR59 | JAI18016.1 | SlitOR36 | (de Fouchier et al., 2017) |
| CpomOR63 | AFC91731.2 | EgriOR22 | (Li et al., 2017) | EposOR60 | JAI18015.1 | SlitOR37 | (de Fouchier et al., 2017) |
| CpomOR64 | AFC91732.1 | EgriOR23 | (Li et al., 2017) | EposOR61 | JAI18014.1 | SlitOR38 | (de Fouchier et al., 2017) |
| CpomOR65 | AFC91730.1 | EgriOR24 | (Li et al., 2017) | EposOR62 | JAI18013.1 | SlitOR39 | (de Fouchier et al., 2017) |
| CpomOR66 | AFC91740.2 | EgriOR25 | (Li et al., 2017) | EposOR63 | (Corcoran et al., 2015) | SlitOR40 | (de Fouchier et al., 2017) |
| CpomOR67 | JAP38439.1 | EgriOR26 | (Li et al., 2017) | EposOR64 | JAI18012.1 | SlitOR41 | (de Fouchier et al., 2017) |
| CpomOR68 | JAP38438.1 | EgriOR27 | (Li et al., 2017) | EposOR65 | JAI18011.1 | SlitOR42 | (de Fouchier et al., 2017) |
| CpomOR71 | JAP38437.1 | EgriOR28 | (Li et al., 2017) | EposOR66 | JAI18010.1 | SlitOR43 | (de Fouchier et al., 2017) |
| CpomOR72 | AFC91748.2 | EgriOR29 | (Li et al., 2017) | EposOR67 | JAI18009.1 | SlitOR44 | (de Fouchier et al., 2017) |
| CpomOrco | AFC91712.1 | EgriOR30 | (Li et al., 2017) | EposOR68 | JAI18008.1 | SlitOR45 | (de Fouchier et al., 2017) |
| DpunOR1 | ARO70500.1 | EgriOR31 | (Li et al., 2017) | EposOR69 | (Corcoran et al., 2015) | SlitOR46 | (de Fouchier et al., 2017) |
| DpunOR3 | ARO70215.1 | EgriOR32 | (Li et al., 2017) | EposOR70 | (Corcoran et al., 2015) | SlitOR47 | (de Fouchier et al., 2017) |
| DpunOR4 | ARO70216.1 | EgriOR33 | (Li et al., 2017) | EposOrco | ACJ12928.2 | SlitOR48 | (de Fouchier et al., 2017) |
| DpunOR5 | ARO70217.1 | EgriOR34 | (Li et al., 2017) | EsemOR1 | ATV96622.1 | SlitOR49 | (de Fouchier et al., 2017) |
| DpunOR6 | ARO70218.1 | EgriOR35 | (Li et al., 2017) | EsemOR3 | ATV96623.1 | SlitOR50 | (de Fouchier et al., 2017) |
| DpunOR7 | ARO70219.1 | EgriOR36 | (Li et al., 2017) | EsemOR4 | ATV96624.1 | SlitOR51 | (de Fouchier et al., 2017) |
| DpunOR8 | ARO70220.1 | EgriOR37 | (Li et al., 2017) | EsemOR5 | ATV96625.1 | SlitOR52 | (de Fouchier et al., 2017) |
| DpunOR9 | ARO70221.1 | EgriOR38 | (Li et al., 2017) | EsemOR6 | ATV96626.1 | SlitOR53 | (de Fouchier et al., 2017) |
| DpunOR10 | ARO70222.1 | EgriOR39 | (Li et al., 2017) | EsemOR7 | (Yuvaraj et al., 2017) | SlitOR54 | (de Fouchier et al., 2017) |
| DpunOR11 | ARO70223.1 | EgriOR40 | (Li et al., 2017) | EsemOR8 | (Yuvaraj et al., 2017) | SlitOR55 | (de Fouchier et al., 2017) |
| DpunOR12 | ARO70224.1 | EgriOR41 | (Li et al., 2017) | EsemOR9 | (Yuvaraj et al., 2017) | SlitOR56 | (de Fouchier et al., 2017) |
| DpunOR13 | ARO70225.1 | EgriOR42 | (Li et al., 2017) | EsemOR10 | (Yuvaraj et al., 2017) | SlitOR57 | (de Fouchier et al., 2017) |
| DpunOR14 | ARO70226.1 | EgriOR43 | (Li et al., 2017) | EsemOR11 | (Yuvaraj et al., 2017) | SlitOR58 | (de Fouchier et al., 2017) |
| DpunOR15 | ARO70227.1 | EgriOR44 | (Li et al., 2017) | EsemOR12 | (Yuvaraj et al., 2017) | SlitOR59 | (de Fouchier et al., 2017) |
| DpunOR16 | ARO70228.1 | EgriOrco | (Li et al., 2017) | EsemOR13 | (Yuvaraj et al., 2017) | SlitOR60 | (de Fouchier et al., 2017) |
| DpunOR17 | ARO70229.1 | EposOR1 | ACJ12927.2 | EsemOR14 | (Yuvaraj et al., 2017) | SlitORco | (de Fouchier et al., 2017) |
| DpunOR18 | ARO70230.1 | EposOR3 | ACJ12929.2 | EsemOR15 | (Yuvaraj et al., 2017) |  |  |
| DpunOR19 | ARO70231.1 | EposOR4 | JAI18062.1 | EsemOR16 | (Yuvaraj et al., 2017) |  |  |
| DpunOR20 | ARO70232.1 | EposOR5 | JAI18061.1 | EsemOR17 | (Yuvaraj et al., 2017) |  |  |

Note: odorant receptors of *Ectropis grisescens* came from “Chemosensory gene families in *Ectropis grisescens* and candidates for detection of Type-II sex pheromones”; odorant receptors of *Epiphyas postvittana* came from “The peripheral olfactory repertoire of the lightbrown apple moth, *Epiphyas postvittana*”; odorant receptors of *Eriocrania semipurpurella* came from “Characterization of Odorant Receptors from a Non-ditrysian Moth, *Eriocrania semipurpurella* Sheds Light on the Origin of Sex Pheromone Receptors in Lepidoptera”; odorant receptors of *Spodoptera littoralis* came from “Functional evolution of Lepidoptera olfactory receptors revealed by deorphanization of a moth repertoire”.

**TABLE 9 |** Blastx matches for genes encoding candidate IRs in *P. flammans*

| Gene Name | Gene  length (bp) | ORF (aa) | Complete ORF | TMD  (No.) | Best blastx match | | | | | Transcriptome source  (No.) |
| --- | --- | --- | --- | --- | --- | --- | --- | --- | --- | --- |
|  |  |  |  |  | Gene description | species | Accession number | Identity% | E-value |  |
| PflaIR8a | 2700 | 899 | Yes | 4 | ionotropic receptor | *Eogystia hippophaecolus* | AOG12845.1 | 74.60 | 0 | 3 |
| PflaIR85a | 1869 | 622 | Yes | 3 | ionotropic receptor 85a | *Heliconius melpomene* rosina | AMM70659.1 | 51.62 | 0 | 3 |
| PflaIR60a | 2007 | 668 | Yes | 5 | ionotropic receptor 60a | *Peridroma saucia* | QHB15321.1 | 52.89 | 0 | 3 |
| PflaIR25a | 2790 | 929 | Yes | 3 | ionotropic receptor 25a | *Hedya nubiferana* | AST36228.1 | 90.55 | 0 | 3 |
| PflaIR76b | 1653 | 550 | Yes | 3 | ionotropic receptor 76b | *Streltzoviella insularis* | QLI62110.1 | 64.05 | 0 | 3 |
| PflaIR75p | 1959 | 652 | Yes | 3 | ionotropic receptor 75p | *Cydia pomonella* | AFC91755.2 | 64.38 | 0 | 3 |
| PflaIR2 | 1803 | 600 | Yes | 4 | ionotropic receptor | *Eogystia hippophaecolus* | AOG12849.1 | 53.11 | 0 | 3 |
| PflaIR75q | 1890 | 629 | Yes | 3 | ionotropic receptor 3 | *Conopomorpha sinensis* | AXY83444.1 | 44.90 | 1e-172 | 3 |
| PflaIR21a | 2502 | 833 | Yes | 3 | ionotropic receptor 21a | *Ostrinia furnacalis* | XP_028175661.1 | 68.84 | 0 | 2 |
| PflaIR40a | 1116 | 371 | Yes | 1 | ionotropic receptor | *Histia rhodope* | QMS80367.1 | 84.59 | 0 | 2 |
| PflaIR41a | 1812 | 603 | Yes | 2 | ionotropic receptor | *Eogystia hippophaecolus* | AOG12846.1 | 61.62 | 0 | 2 |
| PflaIR93a | 2600 | 866 | Yes | 3 | ionotropic receptor 93a | *Peridroma saucia* | QHB15334.1 | 72.66 | 0 | 2 |
| PflaIR75c | 1887 | 628 | Yes | 1 | ionotropic receptor 75a | *Manduca sexta* | XP_037298953.1 | 47.62 | 9e-171 | 2 |
| PflaIR87a | 1956 | 651 | Yes | 3 | ionotropic receptor | *Glyphodes pyloalis* | QIJ45773.1 | 67.67 | 0 | 2 |
| PflaIR75d | 1848 | 615 | Yes | 2 | ionotropic receptor 75a | *Hyposmocoma kahamanoa* | XP_026314114.1 | 56.16 | 0 | 2 |
| PflaGluR1 | 1383 | 460 | Yes | 1 | ionotropic receptor | *Ostrinia furnacalis* | BAR64816.1 | 69.06 | 0 | 2 |

**TABLE 10 |** Protein names and gene accession used in phylogenetic tree of IRs

| **Name** | **ID** | **Name** | **ID** | **Name** | **ID** | **Name** | **ID** |
| --- | --- | --- | --- | --- | --- | --- | --- |
| BmorIR21a | XP_037866853.1 | CmedIR75p | ALT31628.1 | HassIR7d | AJD81625.1 | PsauIR4 | QHB15337.1 |
| BmorIR25a | XP_021207886.1 | CmedIR75q | ALT31629.1 | HassIR21a | AJD81627.1 | PsauIR6 | QHB15338.1 |
| BmorIR40a | XP_021202684.1 | CmedIR87a | ALT31631.1 | HassIR25a | AJD81628.1 | PsauIR7 | QHB15339.1 |
| BmorIR75a | XP_021205850.1 | CmedIR93a | ALT31632.1 | HassIR75b | AJD81640.1 | PsauIR8a | QHB15316.1 |
| BmorIR93a | XP_037875093.1 | CpomIR1 | AFC91754.2 | HassIR75p | AJD81636.1 | PsauIR25a | QHB15318.1 |
| CfagIR2 | AST36354.1 | CpomIR2 | AFC91762.2 | HassIR75q | AJD81639.1 | PsauIR64a | QHB15323.1 |
| CfagIR3 | AST36355.1 | CpomIR3 | AFC91767.2 | HassIR75d | AJD81642.1 | PsauIR68a | QHB15324.1 |
| CfagIR8a | AST36356.1 | CpomIR4 | AFC91763.2 | HassIR93a | AJD81643.1 | PsauIR75d | QHB15325.1 |
| CfagIR21a | AST36357.1 | CpomIR7d | AFC91766.2 | GmelIR8a | QEI46862.1 | PsauIR75p | QHB15328.1 |
| CfagIR25a | AST36358.1 | CpomIR8a | AFC91764.2 | GmelIR25a | QEI46864.1 | PsauIR75q | QHB15329.1 |
| CfagIR41a | AST36359.1 | CpomIR21a | AFC91761.2 | GmelIR75p | QEI46872.1 | PsauIR76b | QHB15331.1 |
| CfagIR60a | AST36360.1 | CpomIR25a | CpomIR25a | GmelIR75q | QEI46874.1 | PsauIR85a | QHB15332.1 |
| CfagIR64a | AST36361.1 | CpomIR41a | JAP38476.1 | GmelIR76b | QEI46875.1 | PsauIR87a | QHB15333.1 |
| CfagIR75q | AST36363.1 | CpomIR60a | JAP38474.1 | GmelIR87a | QEI46876.1 | PsauIR93a | QHB15334.1 |
| CfagIR75p | AST36362.1 | CpomIR64a | JAP38473.1 | GmelIR93a | QEI46877.1 | SlitIR1 | ADR64688.1 |
| CfagIR76b | AST36365.1 | CpomIR75d | JAP38472.1 | MsepIR1 | QNS36181.1 | SlitIR21a | ADR64678.1 |
| CfagIR87a | AST36366.1 | CpomIR75p | AFC91755.2 | MsepIR2 | QNS36182.1 | SlitIR25a | ADR64679.1 |
| CfagIR93a | AST36367.1 | CpomIR75q | AFC91756.2 | MsepIR5 | JAV45791.1 | SlitIR41a | ADR64681.1 |
| CpunIR1 | ARO76464.1 | CpomIR76b | AFC91765.1 | MsepIR7d | QNS36183.1 | SlitIR68a | ADR64682.1 |
| CpunIR2 | ARO76465.1 | CpomIR87a | AFC91760.2 | MsepIR10a | QNS36184.1 | SlitIR75d | ADR64683.1 |
| CpunIR3 | ARO76466.1 | CpomIR93a | AFC91753.2 | MsepIR21a | QNS36185.1 | SlitIR75p | ADR64684.1 |
| CpunIR4 | ARO76467.1 | HnubIR7d | AST36225.1 | MsepIR25a | QNS36186.1 | SlitIR75q | ADR64686.1 |
| CpunIR6 | ARO76469.1 | HnubIR8a | AST36226.1 | MsepIR40a | QNS36187.1 | SlitIR76b | ADR64687.1 |
| CpunIR7 | ARO76470.1 | HnubIR21a | AST36227.1 | MsepIR41a | QNS36188.1 | SlitIR87a | ADR64689.1 |
| CpunIR25a | ARO76463.1 | HnubIR25a | AST36228.1 | MsepIR75a | QNS36189.1 | SinsIR21a | QLI62108.1 |
| CmedIR1 | ALT31619.1 | HnubIR41a | AST36229.1 | MsepIR75c | QNS36190.1 | SinsIR68a | QLI62101.1 |
| CmedIR2 | ALT31620.1 | HnubIR60a | AST36231.1 | MsepIR75d | QNS36191.1 | SinsIR41a | QLI62106.1 |
| CmedIR21a | CmedIR21a | HnubIR64a | AST36232.1 | MsepIR75p | QNS36192.1 | SinsIR75a | QLI62109.1 |
| CmedIR25a | APY22696.1 | HnubIR75p | AST36234.1 | MsepIR76b | QNS36193.1 | SinsIR75p | QLI62103.1 |
| CmedIR40a | APY22697.1 | HnubIR75q | AST36236.1 | MsepIR87a | QNS36194.1 | SinsIR75q | QLI62102.1 |
| CmedIR60a | APY22698.1 | HnubIR76b | AST36237.1 | MsepIR93a | QNS36195.1 | SinsIR76b | QLI62110.1 |
| CmedIR64a | APY22699.1 | HnubIR87a | AST36238.1 | PsauIR2 | QHB15335.1 | SinsIR93a | QLI62100.1 |
| CmedIR75 | ALT31627.1 | HassIR2 | AJD81622.1 | PsauIR3 | QHB15336.1 | HnubGluR2 | AST36240.1 |
| HnubGluR4 | AST36241.1 | HassGluR2 | AJD81613.1 | HassGluR3 | AJD81614.1 | HassGluR4 | AJD81615.1 |
| HassGluR5 | AJD81616.1 | HassGluR6 | AJD81617.1 | HassGluR7 | AJD81618.1 | HassGluR8 | AJD81619.1 |
| PsauIR1.1 | (Sun et al., 2020b) | PsauIR1.2 | (Sun et al., 2020b) | PsauIR7d.1 | (Sun et al., 2020b) | PsauIR7d.3 | (Sun et al., 2020b) |
| PsauiGluR2 | (Sun et al., 2020b) | PsauiGluR3 | (Sun et al., 2020b) | PsauiGluR4 | (Sun et al., 2020b) | PsauiGluR6 | (Sun et al., 2020b) |
| PsauiGluR7 | (Sun et al., 2020b) | PsauiGluR8 | (Sun et al., 2020b) |  |  |  |  |

**TABLE 11 |** Blastx matches for genes encoding candidate GRs in *P. flammans*

| Gene Name | Gene  length  (bp) | ORF (aa) | Complete  ORF | TMD  (No.) | Best blastx match | | | | | Transcriptome source  (No.) |
| --- | --- | --- | --- | --- | --- | --- | --- | --- | --- | --- |
|  |  |  |  |  | Gene description | species | Accession number | Identity  % | E-value |  |
| PflaGR1 | 1275 | 424 | Yes | 6 | gustatory receptor 13 | *Achelura yunnanensis* | QZH55046.1 | 42.12 | 8e-93 | 3 |
| PflaGR2 | 1227 | 408 | Yes | 7 | gustatory receptor 13 | *Achelura yunnanensis* | QZH55046.1 | 27.98 | 8e-21 | 3 |
| PflaGR3 | 1227 | 408 | Yes | 6 | gustatory receptor 7 | *Peridroma saucia* | QHB15307.1 | 27.87 | 1e-19 | 3 |
| PflaGR4 | 1116 | 371 | No | 6 | gustatory receptor 13 | *Achelura yunnanensis* | QZH55046.1 | 42.82 | 42.82 | 3 |
| PflaGR5 | 1275 | 424 | Yes | 7 | gustatory receptor 13 | *Achelura yunnanensis* | QZH55046.1 | 42.12 | 8e-94 | 3 |
| PflaGR6 | 1227 | 408 | Yes | 7 | gustatory receptor 13 | *Achelura yunnanensis* | QZH55046.1 | 27.98 | 4e-21 | 3 |
| PflaGR7 | 981 | 326 | No | 5 | gustatory receptor 16 | *Achelura yunnanensis* | QZH55049.1 | 44.73 | 2e-88 | 2 |
| PflaGR8 | 1193 | 396 | No | 7 | gustatory receptor | *Helicoverpa armigera* | AGA04648.1 | 73.68 | 0 | 2 |
| PflaGR9 | 678 | 225 | No | 4 | gustatory receptor 2 | *Helicoverpa assulta* | AJD81595.1 | 90.86 | 3e-99 | 2 |
| PflaGR10 | 897 | 298 | No | 2 | gustatory receptor | *Eogystia hippophaecolus* | AOG12966.1 | 40.24 | 2e-11 | 2 |
| PflaGR11 | 699 | 232 | No | 0 | gustatory receptor 2 | *Athetis dissimilis* | ALM26252.1 | 42.49 | 1e-53 | 2 |
| PflaGR12 | 1219 | 405 | No | 7 | gustatory receptor 43a | *Danaus plexippus* | XP_032526693.1 | 32.54 | 2e-47 | 2 |
| PflaGR13 | 621 | 206 | No | 3 | gustatory receptor 11 | *Bombyx mori* | DAA06375.1 | 43.75 | 2e-12 | 2 |
| PflaGR14 | 708 | 236 | No | 4 | gustatory receptor 13 | *Achelura yunnanensis* | QZH55046.1 | 40.95 | 7e-52 | 2 |

**TABLE 12 |** Protein names and gene accession used in phylogenetic tree of GRs

| **Name** | **ID** | **Name** | **ID** | **Name** | **ID** | **Name** | **ID** |
| --- | --- | --- | --- | --- | --- | --- | --- |
| AtraGR28b | XP_013183192.1 | BmorGR61 | DAA06393.1 | HnubGR6 | AST36214.1 | GmelGR68a | XP_031766124.1 |
| AtraGR43a | XP_013188294.1 | BmorGR62 | DAA06394.1 | HnubGR55 | AST36215.1 | OfurGR5a | XP_028179059.1 |
| AtraGR64f | XP_013189983.1 | BmorGR63 | DAA06395.1 | HnubGR58 | AST36216.1 | OfurGR28a | XP_028163542.1 |
| AtraGR64e | XP_013189985.1 | BmorGR64 | DAA06396.1 | HnubGR60 | AST36217.1 | OfurGR43a | XP_028175090.1 |
| AtraGR64a | XP_013196848.1 | BmorGR64a | XP_037871940.1 | HnubGR63 | AST36218.1 | OfurGR64a | XP_028179080.1 |
| AtraGR5a | XP_013197699.1 | BmorGR64f | XP_037871621.1 | HnubGR68 | AST36221.1 | OfurGR64e | XP_028179060.1 |
| BmorGR7 | DAA06374.1 | BmorGR66 | BAK52798.1 | HassGR1 | AGK90023.1 | OfurGR64f | XP_028178841.1 |
| BmorGR8 | NP_001124344.1 | BmorGR67 | BAK52799.1 | HassGR2 | AJD81595.1 | OfurGR68a | XP_028173528.1 |
| BmorGR9 | XP_021207986.1 | BmorGR68 | BAK52800.1 | HassGR3 | AJD81596.1 | PsauGR1 | QHB15301.1 |
| BmorGR10 | BAS18817.1 | CpunGR1 | ARO76472.1 | HassGR4 | AGK90024.1 | PsauGR2 | QHB15302.1 |
| BmorGR11 | DAA06375.1 | CpunGR2 | ARO76473.1 | HassGR5 | AGK90025.1 | PsauGR3 | QHB15303.1 |
| BmorGR13 | DAA06376.1 | CpunGR3 | ARO76474.1 | HassGR6 | AJD81599.1 | PsauGR4 | QHB15304.1 |
| BmorGR14 | DAA06377.1 | CpunGR4 | ARO76475.1 | HassGR7 | AJD81600.1 | PsauGR5 | QHB15305.1 |
| BmorGR15 | DAA06378.1 | CpunGR5 | ARO76476.1 | HassGR10 | AJD81603.1 | PsauGR6 | QHB15306.1 |
| BmorGR16 | DAA06379.1 | CpunGR10 | ARO76489.1 | HassGR11 | AJD81604.1 | PsauGR7 | QHB15307.1 |
| BmorGR17 | DAA06380.1 | CpomGR1 | JAP38495.1 | HassGR12 | AJD81605.1 | PsauGR8 | QHB15308.1 |
| BmorGR18 | DAA06381.1 | CpomGR2 | JAP38494.1 | HassGR13 | AJD81606.1 | PsauGR9 | QHB15309.1 |
| BmorGR26 | DAA06382.1 | CpomGR3 | JAP38493.1 | HassGR14 | AJD81607.1 | PsauGR10 | QHB15310.1 |
| BmorGR27 | DAA06383.1 | CpomGR4 | JAP38492.1 | HassGR15 | AJD81608.1 | PxylGR2a | XP_011561224.2 |
| BmorGR28 | BAW33746.1 | CpomGR6 | JAP38491.1 | HassGR16 | AJD81609.1 | PxylGR28a | XP_037970631.1 |
| BmorGR28a | XP_012546109.1 | CpomGR8 | AFC91733.2 | HassGR17 | AJD81610.1 | PxylGR28b | XP_011565113.2 |
| BmorGR29 | DAA06384.1 | CpomGR9 | JAP38490.1 | HassGR18 | AJD81611.1 | PxylGR43a | XP_037974720.1 |
| BmorGR30 | DAA06385.1 | CpomGR10 | JAP38489.1 | HarmGR1 | AGK90010.1 | PxylGR64a | XP_037963809.1 |
| BmorGR31 | BAW33756.1 | CpomGR29 | JAP38488.1 | HarmGR4 | AGK90011.1 | PxylGR64e | PxylGR64e |
| BmorGR33 | DAA06386.1 | CpomGR30 | JAP38487.1 | HarmGR5 | QJR83062.1 | PxylGR64f | XP_037963803.1 |
| BmorGR45 | NP_001124346.1 | CpomGR55 | JAP38486.1 | HarmGR6 | QJR83063.1 | PxylGR68a | XP_037969597.1 |
| BmorGR47 | ACD85126.1 | CpomGR58 | JAP38485.1 | HarmGR7 | QJR83064.1 | SlitGR5a | XP_022818462.1 |
| BmorGR58 | DAA06392.1 | CpomGR60 | JAP38484.1 | HarmGR8 | QJR83065.1 | SlitGR28a | XP_022816229.1 |
| BmorGR50 | DAA06387.1 | CpomGR61 | JAP38483.1 | GmelGR28b | XP_031766287.1 | SlitGR43a | XP_022831987.1 |
| BmorGR51 | DAA06388.1 | CpomGR63 | JAP38482.1 | GmelGR43a | XP_031764556.1 | SlitGR64a | XP_022826921.1 |
| BmorGR53 | DAA06389.1 | CpomGR68 | JAP38480.1 | GmelGR64a | XP_031769969.1 | SlitGR64e | XP_022826944.1 |
| BmorGR56 | DAA06390.1 | HnubGR2 | AST36211.1 | GmelGR64b | XP_026764494.1 | SlitGR64f | XP_022826955.1 |
| BmorGR57 | DAA06391.1 | HnubGR4 | AST36212.1 | GmelGR64e | XP_026764713.1 |  |  |
| BmorGR60 | NP_001124347.1 | HnubGR5 | AST36213.1 | GmelGR64f | XP_026764722.1 |  |  |

**TABLE 13 |** Gene information for SNMPs of *P . flammans*

| Gene Name | Gene  length (bp) | ORF (aa) | Complete ORF | TMD  (No.) | Best blastx match | | | | | Transcriptome  source  (No.) |
| --- | --- | --- | --- | --- | --- | --- | --- | --- | --- | --- |
|  |  |  |  |  | Gene description | species | Accession number | Identity% | E-value |  |
| PflaSNMP1 | 1563 | 520 | Yes | 2 | sensory neuron membrane protein 1 | *Hyposmocoma kahamanoa* | XP_026331738.1 | 77.78 | 0 | 3 |
| PflaSNMP2 | 1563 | 520 | Yes | 2 | sensory neuron membrane protein 2 | *Ostrinia nubilalis* | ADQ73889.1 | 66.48 | 0 | 3 |

**TABLE 14 |** Protein names and gene accession used in phylogenetic tree of SNMPs

| **Name** | **ID** | **Name** | **ID** | **Name** | **ID** | **Name** | **ID** |
| --- | --- | --- | --- | --- | --- | --- | --- |
| BmorSNMP1 | XP_037873020.1 | EoblSNMP2 | ANA75033.1 | MsexSNMP1 | AAG49366.1 | SlitSNMP2 | XP_022829056.1 |
| BmorSNMP2 | XP_037870755.1 | HarmSNMP | AAO15604.1 | MsexSNMP2 | XP_037302081.1 | SlitSNMP3 | AKT26506.1 |
| CsupSNMP1 | AFS50073.1 | HarmSNMP2 | XP_021182700.1 | OfurSNMP1 | ADQ73894.1 | SexiSNMP1 | AGN52676.1 |
| CsupSNMP2 | AFS50074.1 | HvirSNMP1 | CAB65739.1 | OfurSNMP2 | XP_028176441.1 | SexiSNMP2 | SexiSNMP2 |
| CpomSNMP1 | ATD12153.1 | HvirSNMP2 | CAP19028.1 | PxylSNMP1 | ADK66278.1 | SexiSNMP3 | AKT26505.1 |
| CpomSNMP2 | ANE31722.1 | GmelSNMP1 | QEI46778.1 | PxylSNMP2 | KAG7305165.1 |  |  |
| EoblSNMP1 | AZB86632.1 | GmelSNMP2 | QEI46779.1 | SlitSNMP1 | AGN48098.1 |  |  |
